# Beneficial microbial consortium improves winter rye performance by modulating bacterial communities in the rhizosphere and enhancing plant nutrient acquisition

**DOI:** 10.3389/fpls.2023.1232288

**Published:** 2023-08-28

**Authors:** Jan Helge Behr, Ioannis D. Kampouris, Doreen Babin, Loreen Sommermann, Davide Francioli, Theresa Kuhl-Nagel, Soumitra Paul Chowdhury, Joerg Geistlinger, Kornelia Smalla, Günter Neumann, Rita Grosch

**Affiliations:** ^1^ Leibniz Institute of Vegetable and Ornamental Crops (IGZ), Plant-Microbe Systems, Großbeeren, Germany; ^2^ Julius Kühn Institute (JKI) - Federal Research Centre for Cultivated Plants, Institute for Epidemiology and Pathogen Diagnostics, Braunschweig, Germany; ^3^ Department of Agriculture, Ecotrophology and Landscape Development, Institute of Bioanalytical Sciences (IBAS), Anhalt University of Applied Sciences, Bernburg, Germany; ^4^ Department of Nutritional Crop Physiology, Institute of Crop Science, University of Hohenheim, Stuttgart, Germany; ^5^ Department of Soil Science and Plant Nutrition, Hochschule Geisenheim University, Geisenheim, Germany; ^6^ Institute for Network Biology, Helmholtz Zentrum München – German Research Center for Environmental Health, Neuherberg, Germany

**Keywords:** organic farming, conventional farming, 16S rRNA gene amplicon sequencing, *Bacillus*, *Trichoderma*, *Pseudomonas*

## Abstract

The beneficial effect of microbial consortium application on plants is strongly affected by soil conditions, which are influenced by farming practices. The establishment of microbial inoculants in the rhizosphere is a prerequisite for successful plant-microorganism interactions. This study investigated whether a consortium of beneficial microorganisms establishes in the rhizosphere of a winter crop during the vegetation period, including the winter growing season. In addition, we aimed for a better understanding of its effect on plant performance under different farming practices. Winter rye plants grown in a long-time field trial under conventional or organic farming practices were inoculated after plant emergence in autumn with a microbial consortium containing *Pseudomonas* sp. (RU47), *Bacillus atrophaeus* (ABi03) and *Trichoderma harzianum* (OMG16). The density of the microbial inoculants in the rhizosphere and root-associated soil was quantified in autumn and the following spring. Furthermore, the influence of the consortium on plant performance and on the rhizosphere bacterial community assembly was investigated using a multidisciplinary approach. Selective plating showed a high colonization density of individual microorganisms of the consortium in the rhizosphere and root-associated soil of winter rye throughout its early growth cycle. 16S rRNA gene amplicon sequencing showed that the farming practice affected mainly the rhizosphere bacterial communities in autumn and spring. However, the microbial consortium inoculated altered also the bacterial community composition at each sampling time point, especially at the beginning of the new growing season in spring. Inoculation of winter rye with the microbial consortium significantly improved the plant nutrient status and performance especially under organic farming. In summary, the microbial consortium showed sufficient efficacy throughout vegetation dormancy when inoculated in autumn and contributed to better plant performance, indicating the potential of microbe-based solutions in organic farming where nutrient availability is limited.

## Introduction

Soil microorganisms play a central role in almost all soil processes, as they do not only contribute to soil formation (Watteau and Villemin, 2018) but also have a significant impact on important ecosystem functions such as nutrient cycling and plant performance (Latz et al., 2016; Lori et al., 2017). Therefore, managing the soil microbial community is essential to maintain soil health and productivity. Microbial diversity has been positively associated with the resilience of microbial communities and soil fertility (van Elsas et al., 2012; Usero et al., 2021). Crops benefit from diverse soil microbial communities as they recruit and enrich beneficial microorganisms (BM) via root exudates in the rhizosphere (Berg and Smalla, 2009; Liu et al., 2021). BM can stimulate plant growth through various mechanisms, such as modulating phytohormone levels (Zubair et al., 2019) and increasing nutrient availability (Jacoby et al., 2017). Furthermore, beneficial rhizosphere microorganisms can trigger induced systemic resistance and protect the plant against below- and aboveground pathogens (Wei et al., 2020; Vlot et al., 2021). Indeed, plant pathogens can be controlled by BM either directly by producing lytic enzymes and bioactive compounds (Fira et al., 2018; Ali et al., 2020) or indirectly by competing for resources in the same ecological niche like the rhizosphere (Rai et al., 2016).

The inoculation of microorganisms with known beneficial functions for plants is a practical approach that is able to modulate the composition of indigenous microbial communities, particularly in the rhizosphere (Deng et al., 2019; Berg et al., 2021). Although many BM show promising functions on plant growth and protection under controlled conditions *in vitro* and *in vivo* (Mendes et al., 2013; Mazzola and Freilich, 2017), their efficacy in the field is often limited due to insufficient colonization of the host rhizosphere and/or unfavorable conditions (Parnell et al., 2016; Batista and Singh, 2021). To successfully colonize the rhizosphere, inoculated BM have to bypass the barrier of the indigenous soil microbial community, adapt to variable environmental conditions, and interact with the host plant using mainly root exudates as a chemoattractant and substrate (Finkel et al., 2017; Berg et al., 2021). Different plant developmental stages and changing environmental conditions shift the root exudation pattern and thereby also the composition of the microbial community in the rhizosphere (Chaparro et al., 2014; Windisch et al., 2021), which may affect the rhizosphere competence of BM. Thus, even if inoculated BM successfully establish in the rhizosphere at early plant development, persistence over an extended period is not guaranteed. Inoculation with multi-strain consortia containing two or more BM has shown increased efficacy compared to single BM species (Sun et al., 2022). Consortia of BM, comprising members with diverse functions, can occupy different ecological niches, making them more resilient to variable environments (Bradáčová et al., 2019; Tosi et al., 2020). Furthermore, the interaction among consortium members increases their ecological fitness, and complementary beneficial functions generate synergies that promote their interaction with the plant host (Moradtalab et al., 2020; Pascale et al., 2020).

Different farming practices, such as conventional and organic farming, drastically affect the structure of microbial communities (Francioli et al., 2016; Windisch et al., 2021). Chowdhury et al. (2019) showed that conventional farming using synthetic pesticides and mineral fertilizers shaped the rhizosphere microbial community composition differently from organic farming, which was associated with varying states of plant health. There is currently a limited understanding of how the farming practice impacts the efficacy of a beneficial microorganism consortium (BMc). For instance, crops can benefit more from increased nutrient bioavailability through BMc in nutrient-deficient soils (Eltlbany et al., 2019), while in soils with high nutrient level the rhizosphere colonization by the inoculated BMc might be reduced (Lopes et al., 2021).

The main goal of this study was to assess the rhizosphere competence of the inoculated BMc members in winter rye (*Secale cereale* cv. Conduct) during the growing season in dependence on different farming practices. Winter cereal crops are exposed to extreme environmental changes during the growing season, raising the question of whether the members of a BMc inoculated in autumn can establish in the rhizosphere of winter rye and maintain a high colonization density over the winter period. Furthermore, the impact of the applied BMc on plant performance may be defined by the physicochemical properties of the soil and the farming practice dependent soil microbial communities. In this context, we hypothesized that (i) early inoculation of winter rye enables sufficient colonization of each BMc member at early plant developmental stage, supporting its persistence in the rhizosphere throughout the vegetation period; (ii) the application of BMc shapes the composition of the rhizosphere bacterial community depending on the farming practice and thus differentially affects the plant performance.

To test our hypotheses, we first characterized *in vitro* the plant growth-promoting traits and the ability of each BMc member to inhibit the growth of soil-borne pathogens. Then, we used a long-time field experiment to evaluate the rhizosphere competence and efficacy of the tested BMc on plant performance under field conditions with different farming practices (organic vs. conventional farming practice). Winter rye plants were inoculated shortly after emergence and BMc colonization and plant performance were evaluated in autumn and spring. The dynamics of the bacterial community throughout vegetation dormancy were characterized by 16S rRNA amplicon sequencing.

## Materials and methods

### Microbial consortium used in the study

The microbial members of the consortium [*Pseudomonas* sp. (RU47; strain collection of the Julius Kühn Institute, Braunschweig, Germany), *Bacillus atrophaeus* (ABi03; strain provided by ABiTEP, Berlin, Germany), and *Trichoderma harzianum* (OMG16; strain collection of Anhalt University of Applied Sciences, Bernburg, Germany)] were selected as each strain showed beneficial effects on plant performance in previous experiments and trials (Schreiter et al., 2014; Mpanga et al., 2018; Schreiter et al., 2018; Mpanga et al., 2019; Moradtalab et al., 2020; Hafiz et al., 2022).

### *In vitro* characterization of consortium members

To provide a more comprehensive characterization of the plant-beneficial traits associated with each consortium member (*Pseudomonas* sp. RU47, *B. atrophaeus* ABi03, and *T. harzianum* OMG16) a series of *in vitro* tests were conducted to assess their antagonistic capabilities and their capacity for plant growth promotion. The modulation of plant growth hormones by indole-3-acetic acid (IAA) production and 1-aminocyclopropane-1-carboxylate (ACC) deaminase activity (Koo et al., 2010), as well as the increased nutrient availability by siderophore production (Schwyn and Neilands, 1987) was tested for each strain. In addition, the solubilization of potassium feldspar (KAlSi_3_O_8_) (Breitkreuz et al., 2021), calcium phosphate (Ca_3_(PO_4_)_2_) (Nautiyal, 1999), zinc oxide (ZnO) (Mumtaz et al., 2017), manganese dioxide (MnO_2_) (Sanket et al., 2017) and ammonia production (Cappuccino and Sherman, 2014) by the individual strains was analyzed. For nutrient solubilization assays, the medium was modified for OMG16 using Waksman-agar (Huber et al., 1987).

Cellulase (Berg et al., 2005), chitinase (Berg et al., 2001), β-1,3-glucanase (Weinert et al., 2010), protease (Berg et al., 2005), and hydrogen cyanide (HCN) (Donate-Correa et al., 2005) formation were tested as antagonistic functions. *In vitro* tests for enzyme secretion with antagonistic functions were not feasible for OMG16 due to cultivation incompatibilities. A dual culture assay modified after Lin and Ho (2021) was used to evaluate the ability of each strain to inhibit pathogen growth *in vitro*. For the two bacterial strains RU47 and ABi03, 20 µl of bacterial suspension (10^5^ colony forming units (CFU) mL^-1^) was dispensed in a centered line of a nutrient-agar I plate (Sifin diagnostics, Berlin, Germany). Freshly grown mycelia plugs (ø 6 mm) of *Fusarium culmorum* (Isolate F247), *F. graminearum* (Isolate FG66), and *Rhizoctonia solani* Isolate AG3 (Isolate Ben3) were placed at a distance of 2.5 cm on both sides of the line. For the fungal strain OMG16, precultured mycelia plugs of OMG16 and the pathogen were placed on opposite sides of a potato dextrose agar (PDA, Carl Roth, Karlsruhe, Germany) plate. Dual culture assays were cultivated in the dark at 25°C for 10 days. Antagonistic properties, i.e. formation of an inhibition zone, of the individual BMc strains were checked every second day (**Supplementary Figure 1**).

### Field site description

The experiment was conducted from 2020-2021 in an experimental field with plots under conventional and organic farming practices as part of the long-time field trial in Thyrow (Thy_ABS; 52°15’ N, 13°14’ E, 44 m a.s.l.), which was established in 2005 at the Agricultural Research Institute of the Humboldt University of Berlin. Pallid brown earth (Luvisols) of sand over deep loam with low humus contents is the predominant soil type (83% sand, 14% silt, and only 3% clay) at this site. The average annual temperature (1981-2020) is 9.2°C with a mean annual precipitation of 509.8 mm.

Winter rapeseed (*Brassica napus* ssp. *napus*), winter wheat (*Triticum aestivum*), and winter rye (*Secale cereale*) were rotated in conventional farming and lucerne (*Medicago sativa*), lucerne, potato (*Solanum tuberosum*), triticale (*Triticale*), forage pea (*Pisum sativum* ssp. *arvense*), spring barley (*Hordeum vulgare*), and winter rye (*Secale cereale*) in organic farming. Mineral fertilization was used in conventional farming practice according to the agricultural standard of the region (N = 120 kg ha^−1^, P = 21 kg ha^−1^, K = 120 kg ha^−1^, Mg = 12 kg ha^−1^, S = 14 kg ha^−1^). Nitrogen was applied as calcium ammonium nitrate (KAS) and potassium, magnesium and sulfur as equal ratios of Patentkali^®^ (K + S Minerals and Agriculture, Kassel, Germany) and Korn-Kali^®^ (K + S Minerals and Agriculture). Triple superphosphate (46% P_2_O_5_) was used for phosphate fertilization. Before growing winter rye and triticale, cattle manure was incorporated into the soil in organic farming (15,000 kg ha^-1^), supported by the cultivation of lucerne and forage peas. Plant protection was conducted following the agricultural standards of conventional cultivation [herbicides: 2.0 L ha^-1^ Picona (BASF, Ludwigshafen, Germany), 0.25 L ha^-1^ Cadou SC (Bayer, Leverkusen, Germany); fungicides: 2.0 L ha^-1^ Ceriax (BASF)]. In organic farming, no pesticides were used, and emerging weeds were removed with a mechanical cultivator. Before sowing of winter rye (sowing density: 200 seeds per m^2^ in conventional farming and 300 seeds per m^2^ in organic farming), the soil was tilled, including stubble clearing with a disc harrow, plowing to a depth of about 20-25 cm and seedbed preparation with a reciprocating harrow.

### Preparation of the microbial consortium and its field application

The *T. harzianum* OMG16 strain was cultivated on PDA medium (Carl Roth) supplemented with 100 mg L^-1^ penicillin (Carl Roth), 50 mg L^-1^ streptomycin sulfate (Sigma-Aldrich, St. Louis, USA), and 10 mg L^-1^ tetracycline hydrochloride (AppliChem, Darmstadt, Germany). To prepare the field inoculum, millet grains (*Panicum miliaceum*) were soaked overnight in tap water. The grains were washed intensively with cold tap water afterwards. Clean grains (500 g per bag) were transferred to bags designed for fungal culture (sun bag; Sigma-Aldrich) and autoclaved on three subsequent days. A disc with freshly grown OMG16 mycelium was added to the grains and incubated for 30 days in the dark at room temperature. After OMG16 entirely colonized the millet grains, the inoculum was freeze-dried and ground. Shortly before winter rye sowing, the powder (100 mg m^-2^) was incorporated into the soil to a depth of 20 cm. Freeze-dried millet grain powder without OMG16 was used as a control.

The *B. atrophaeu*s ABi03 and *Pseudomonas* sp. RU47 strains were used as spontaneous rifampicin-resistant mutants provided as a prepared inoculum by ABiTEP (Schreiter et al., 2018). Two weeks after winter rye emergence, each plot was drenched with 4 L of the bacterial ABi03 and RU47 suspension, mixed with tap water (each 7.5 x 10^7^ CFU mL^-1^). Control plots were drenched with tap water. Each treatment included four replicates (1.5 x 2 m) arranged in a randomized block design for both farming practices.

### Sampling and verification of rhizosphere competence

Only plants from the inner plot core (0.5 x 1m) of each replicate were considered for the sampling, which was performed in autumn at seven weeks post inoculation (WPI) at EC 21, and in spring of the following year (22 WPI) at EC 25-29. A total of 20 winter rye plants were sampled per plot and treated as one sample per treatment replicate. After plant excavation, the shoots were separated from the roots. To obtain root-associated soil, the soil fraction loosely adhering to the root system was shaken off. Roots, shoots, and root-associated soil were stored immediately at 4°C. After shoot fresh mass determination, shoots were dried by lyophilization, and shoot dry mass (SDM) was measured.

Roots with the remaining tightly attached soil were washed with sterile tap water and cut into 1 cm pieces for rhizosphere sampling. After pooling the root fragments, five grams of root material were transferred into a sterile stomacher bag containing 15 mL of sterile 0.3% NaCl. The rhizosphere samples were obtained by a Stomacher 400 Circulator (Seward Ltd., Worthing, UK) for 60 s at 300 rpm. The supernatant was transferred into a falcon tube, and the Stomacher blending steps were repeated twice. The combined supernatants was used for determining bacterial BMc counts by immediately plating serial dilutions onto nutrient agar I plates (Sifin diagnostics) containing 75 µg mL^-1^ rifampicin (Serva Electrophoresis, Heidelberg, Germany) and 100 µg mL^-1^ cycloheximide (Serva Electrophoresis) and incubated in the dark for 48 h at 28°C. Given the distinct colony morphology of ABi03 and RU47, the CFU were counted and CFU per g root dry mass, used for stomacher processing, were calculated. The remaining Stomacher supernatant was further processed as described by Schreiter et al. (2014) for bacterial rhizosphere community analysis based on total community DNA extraction.

Root-associated soil was used to determine OMG16 counts. Therefore, 5 g of root-associated soil were extracted in a total volume of 50 mL NaCl (0.3%) by shaking for 30 min at room temperature on an orbital shaker (HSM-10; Hettich, Tuttlingen, Germany) at 70 rpm. Subsequently, the supernatant was immediately plated on *Trichoderma* selection medium as suggested by Williams et al. (2003) containing 250 mg L^-1^ chloramphenicol (Carl Roth), 90 mg L^-1^ streptomycin (Sigma-Aldrich), 200 mg L^-1^ quintozene (Sigma-Aldrich), 926 mg L^-1^ propamocarb (ProPlant; Arysta LifeScience, Paris, France) and 150 mg L^-1^ rose bengal (AppliChem). Following an incubation of OMG16 at 28°C in the dark for 10 days, the CFU were quantified per gram soil dry mass. Soil dry mass was obtained by drying five grams of fresh soil at 110°C until a constant weight was reached.

Winter rye ears of the whole plot were sampled at full ripeness. After counting the ears, the grain was threshed and the weight was determined. Yield per hectare was extrapolated for better comparison with literature data.

### Nutrient analysis

The nutrient analysis of shoot material was conducted according to the certified protocols of the Association of German Agricultural Analytic and Research Institutes, VDLUFA, Germany (Verband Deutscher Landwirtschaftlicher Untersuchungs- und Forschungsanstalten, 2011). 200-500 mg of dry plant material were solubilized in 5 mL HNO_3_ (65%) and 3 mL H_2_O_2_ (30%) by microwave digestion at 210°C for 25 min. The extract was filtered and diluted to a final volume of 100 mL. K, P, Mg, S, Ca, Mn, Cu, and Zn concentrations were determined via inductively coupled plasma optical emission spectrometry (ICP-OES, Thermo Fisher Scientific, Dreieich, Germany); total C and N were determined via elemental analysis (Elementary Vario El cube; Langenselbold, Germany). The content of macro- and micronutrients in the root-associated soil were analyzed according to the certified protocols of VDLUFA by AGROLAB (Leinefelde-Worbis, Germany).

### DNA extraction and high-throughput amplicon sequencing of the 16S rRNA gene

The total community DNA was extracted from total rhizosphere pellets using the FastPrep-24 bead-beating system and FastDNA Spin Kit for Soil (MP Biomedicals, Santa Ana, USA) following the manufacturer’s recommendations. Samples were purified with the GeneClean Spin Kit (MP Biomedicals). Library construction and sequencing of the 16S rRNA gene was carried out by Novogene (Cambridge, UK) on NovaSeq PE250 using the primers Uni341F (5’-CCTAYGGGRBGCASCAG-3’) and Uni806R (5’- GGACTACNNGGGTATCTAAT-3’) (Sundberg et al., 2013). Primers and adapters were removed with the software Cutadapt (Martin, 2011). Paired-end reads were processed using the DADA2 pipeline (Callahan et al., 2016). For taxonomic assignment of the obtained amplicon sequence variants (ASVs), the representative sequences were taxonomically classified down to the lowest possible taxonomic level in a Galaxy workflow (Cock et al., 2013) with an e-value cut-off of 0.001 and a percent identity cut-off of 80% against the SILVA 138.1 SSU Ref NR99 database (v.138.1, Quast et al., 2012; v138.1). Sequences identified as plastids (chloroplast or mitochondria) and sequences with less than five reads were removed. No ASV classified as archaeal taxa was present in our dataset. Rarefaction curves were generated to estimate the read coverage, and sequencing depth sufficiently covered the diversity of each sample. Amplicon sequence data has been deposited at the Sequence Read Archive (https://www.ncbi.nlm.nih.gov/sra) under the BioProject accession number PRJNA975889.

### Data analysis

Statistical analysis of winter rye growth, yield, nutrient concentration, and BMc abundance was performed using R (v.4.2.2, R Core Team, 2022). The main and interaction effects between long-time farming practices and BMc use were analyzed by two-way ANOVA for SDM and shoot nutrients. Differences in the BMc abundance in the rhizosphere and root-associated soil were tested by two-way ANOVA analyzing the main and interaction effects of the sampling time and different long-time farming practices. All analyses were inspected visually for homoscedasticity and normal distribution of residuals using the Durbin-Watson test from the “car” package (v.3.1.1, Fox and Weisberg, 2019) and normal Q-Q plots. If ANOVA assumptions failed, data were transformed using the R package “rcompanion” (v.2.4.26, Mangiafico, 2023) according to Tukey’s ladder of power approach. The statistical evaluation was conducted using the Sidak-test algorithm and the pairwise comparison combined with a compact letter display of the packages “emmeans” (v.1.8.3, Lenth, 2022), “multcomp” (v.1.4, Hothorn et al., 2008) and “multcompView” (v.0.1.8, Graves et al., 2019). Data were visualized using the “ggplot2” (v.3.4.1, Wickham, 2016) and “ggpattern” packages (v.1.0.1, FC and Davis, 2022). Comparisons with *p*-values below 0.05 were considered statistically significant.

All data handling and statistical analyses for ASV data were performed with the R packages “tidyverse” (v.1.3.1, Wickham et al., 2019), “ggplot2”, “dplyr” (v.1.1.1, Wickham et al., 2023), “tibble” (v.3.1.8, Müller and Wickham, 2022), “reshape2” (v.1.4.4, Wickham, 2007), “vegan” (v.2.6.1, Oksanen et al., 2022) and “phyloseq” (v.3.16, McMurdie and Holmes, 2013). To account for uneven sequencing depth, we repeatedly performed 1000 rarefactions to our dataset’s lowest number of sequences (49,206). Average ASV abundances were calculated based on the 1000 rarefactions. This procedure enables the representation of all observed sequences in equal proportionality and accounts for the random variation introduced by rarefaction (Cameron et al., 2021). Bray-Curtis distance was used for estimating the β-diversity by normalizing the rarefied datasets to relative abundance (%) and applying log_10_ transformation with pseudo-count addition. PERMANOVA tests were applied to evaluate how farming practice and BMc inoculation affected the bacterial community composition. A pairwise PERMANOVA test with Benjamini-Hochberg correction was performed after combining the two treatments into four groups using the “pairwiseadonis” package (v.0.4, Martinez, 2022). Significant differences in α-diversity (ASV Richness, Simpson, Shannon Index, and Evenness) were estimated with two-way ANOVA tests. If the data failed to fulfill the normality criteria based on the Shapiro test, the non-parametric aligned-rank ANOVA was performed (“ARTool” package, v.0.11.1, Kay et al., 2021).

For investigating the effect of farming practice type and BMc inoculation on the abundance of ASVs, ANCOM-BC2 (v2.1.2, Lin and Peddada, 2020) was performed as a differential abundance test with *p-*value correction via Benjamini-Hochberg method. Following the differential abundance test, we performed multiple logistic regressions with Benjamini-Hochberg correction as *post hoc* tests. Logistic regression was selected because of its similar predictive power to machine learning algorithms (e.g., random forest) and its inherent interpretability (Topçuoğlu et al., 2020). We used a logistic regression equation to predict the probability of the treatment to occur (i.e., Organic-BMc, Organic-Control, Conventional-BMc, or Conventional-Control) based on the relative abundance of the ASVs. Consequently, the logistic regression tests enabled us to evaluate whether the differentially abundant ASVs responded to a specific treatment (ASV responders). For each particular treatment, the fitted model’s positive or negative model coefficient indicated higher or lower relative abundance, respectively. ASVs were assigned as positive or negative responders to each treatment based on the model coefficient.

To evaluate whether the biomass yield of rye was associated with higher proportions of the inoculated bacterial strains in comparison to the rest of the bacterial community members in the different treatments, ASV responder sequences classified as *Pseudomonas* spp. or *Bacillus* spp. were aligned to herein constructed databases. For *Pseudomonas* spp. we created a database that contained the 16S rRNA gene sequences from the six 16S rRNA gene copies of the inoculant RU47 (Kuzmanović et al., 2018). For the strain ABi03, we performed cloning and sequencing of the complete 16S rRNA region using primers U8-27 and R1494-1514 (Heuer et al., 2009), where we identified six different 16S rRNA genes. In addition, we retrieved sequences of *Pseudomonas* spp. or *Bacillus* spp. from GenBank 16S rRNA gene restricted to the type strain collection (Federhen, 2015). The V3-V4 region of the 16S rRNA gene, used for Illumina amplicon sequencing, was extracted from the database sequences using the Cutadapt software (Martin, 2011). Pairwise alignment was performed for *Bacillus* spp. and *Pseudomonas* spp., using the ASVs and the constructed databases with the package “msa” (v.1.30.1, Bodenhofer et al., 2015). A distance matrix of the alignment was calculated via the package “seqinr” (v.4.2.23, Charif and Lobry, 2007). A phylogenetic distance was calculated with the neighbor-joining algorithm (package “ape”, v.5.7, Paradis and Schliep, 2019). The phylogenetic distance was visualized as principal coordinates analysis.

## Results

### *In vitro* traits of the microbial consortium members

*In vitro*, the individual members of the used microbial consortium were tested for antagonistic and growth-promoting functions (**Table 1**). ABi03 showed cellulase, chitinase, β-1,3-glucanase, and protease activity but no HCN production. In dual culture assays, ABi03 reduced the growth of all tested pathogens by inhibiting the mycelium growth (**Supplementary Figure 1**). Despite the inability of RU47 to inhibit the growth of *F. culmorum* or *F. graminearum*, it is noteworthy that the RU47 colonies were not overgrown by these pathogens. Additionally, we could confirm a minor inhibition of *R. solani* growth by RU47 as well as protease and HCN production. OMG16 also reduced the growth of *F. culmorum* and *F. graminearum* by spreading faster than the pathogen on PDA. No lytic degradation of the mycelium was observed in the border region between OMG16 and the tested pathogens (**Supplementary Figure 1**).

**Table 1:** *In vitro* characterization of beneficial functions of the inoculated consortium members.

	RU47	ABi03	OMG16
Dual culture assay			
*F. culmorum*	–	+	+
*F. graminearum*	–	+	+
*R. solani*	+^*^	+	+/-
Antagonistic functions			
Cellulase	-^*^	+	NA
Chitinase	-^*^	+	NA
β-1,3-Glucanase	-^*^	+	NA
Protease	+^*^	+	–
HCN	+^**^	–	–
Nutrient availability			
Ammonia	–	+	NA
K-feldspar	+	–	–
Ca_3_(PO_4_)_2_	+^**^	–	–
ZnO	–	–	+
MnO_2_	–	–	NA
Siderophores	+^*^	+	+/-
Plant hormone modulation			
IAA	+^**^	–	+
ACC deaminase	–	–	–

*(Adesina et al., 2009).

**(Kuzmanović et al., 2018).

BMc members were characterized separately for beneficial functions [positive (+), negative (-), inconclusive (+/-), not applicable (N/A)]. Results marked with asterisks were taken from the indicated literature.

RU47 dissolved K-feldspar and Ca_3_(PO_4_)_2_ *in vitro* in nutrient availability assays and showed siderophore as well as IAA production (**Table 1**). For ABi03, ammonia and siderophores were detected, but no increase in solubility of the tested nutrients or plant hormone modulation was observed. Furthermore, IAA, low siderophore production, and ZnO solubility could be shown for OMG16.

### Density of the consortium members in the rhizosphere and soil

**Figure 1:**
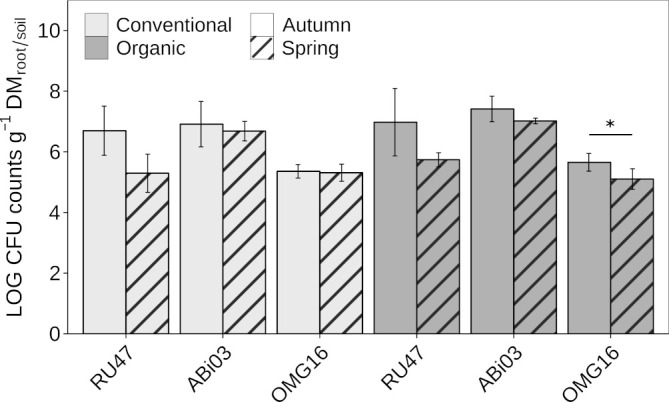
Abundance of beneficial microorganisms in winter rye (*cv.* Conduct) cultivated under different long-time farming practices (conventional vs. organic) in autumn and spring of the same growing season. Bars represent means ± standard deviation of four replicates. A two-way ANOVA was conducted to test the effect of the farming practice and the sampling time on the rhizosphere competence of each member of the consortium (**Supplementary Table 1**). Asterisk indicates a significant difference of the CFU between the sampling time points for each consortium member separately (^∗^ *p* ≤ 0.05).

To evaluate the persistence of the individual BM in the rhizosphere and root-associated soil of winter rye at field scale, we estimated their density after inoculation with two subsequent samplings in autumn and spring of the following year. Both bacterial strains ABi03 and RU47 sufficiently colonized the rhizosphere of winter rye with a density of more than six Log_10_ CFU per gram root dry mass seven WPI at both farming practices (**Figure 1**). The fungal strain OMG16 also showed a sufficient density in the root-associated soil of more than five Log_10_ CFU per gram soil dry mass. No differences in the density of the individual BM in the respective habitat depending on farming practice were observed. At the second sampling in spring (22 WPI), no changes in the density of ABi03 in the rhizosphere of winter rye were found independent of farming practice (**Figure 1**, **Supplementary Table 1**). RU47 density decreased to approximately five Log_10_ CFU per gram of root dry mass at both farming practices compared to the first sampling in autumn. Plants grown under organic farming practice showed a significantly reduced density of OMG16 in the root-associated soil compared to the first sampling in autumn. The two different farming practices had no significant impact on the ability of the individual BM to establish in the rye rhizosphere or root-associated soil at both sampling time points.

### Plant performance

**Figure 2:**
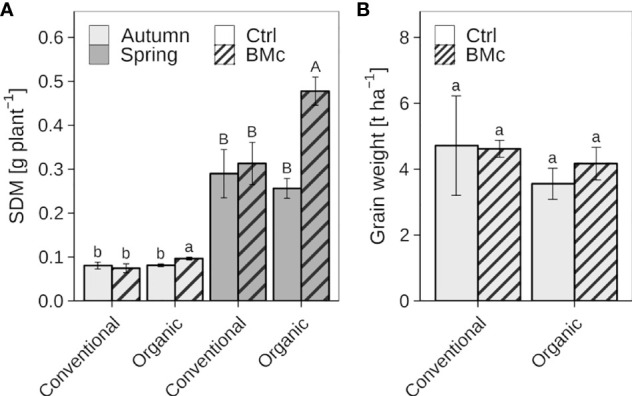
Effect of beneficial microorganisms consortium (BMc) on autumn and spring shoot dry mass (SDM) **(A)** and yield **(B)** of winter rye (*cv.* Conduct) cultivated under conventional and organic long-time farming practices. Bars represent means ± standard deviation of four replicates (**Supplementary Table 2**). Different letters indicate significant differences calculated by the Sidak-test (*p* ≤ 0.05). The type of letter indicates a separate statistical analysis (lowercase: autumn, uppercase: spring).

The mean SDM of winter rye plants at the first sampling (EC 21) in autumn ranged from 0.07 to 0.10 g plant^-1^, while the mean SDM in the second sampling (EC 25-29) in spring was between 0.26 and 0.48 g plant^-1^ (**Figure 2A**, **Supplementary Table 2A**). Control plants without BMc inoculation showed no significant difference in SDM in response to farming practice (conventional vs. organic) at both sampling time points. Organically cultivated winter rye plants treated with the BMc showed a significant increase in SDM compared to those without BMc application in autumn (7 WPI, +20%) and particularly in spring (22 WPI, +45%). In contrast to organic farming, the BMc treatment did not affect the SDM of plants grown under conventional farming at both sampling time points. Two-way ANOVA further revealed a significant interaction between farming practice and BMc inoculation on SDM at both sampling time points (**Supplementary Table 2B**) indicating farming practice-dependent effects of BMc on SDM. At harvest, there were no significant differences in yield between organic and conventional farming, although organically grown plants had slightly lower grain weight per hectare (**Figure 2B**). The BMc treatment had no significant effect on the yield in either conventional or organic farming. However, a slight tendency towards higher yield per unit area was observed in organic farming after the BMc treatment.

To determine whether the nutrient status of winter rye was associated with farming practice and BMc inoculation, the concentrations of several micro- and macronutrients were measured in plants grown across all studied plots and sampling time points (**Table 2**). At the first sampling time in autumn, most nutrients were at the same level for both farming practices, regardless of BMc inoculation. Two-way ANOVA revealed an overall significant effect (*p* ≤ 0.021) of BMc inoculation on the nitrogen concentration (**Supplementary Table 3**), which was slightly increased with BMc inoculation for both farming practices. However, a pairwise comparison could not verify a significant difference between the four treatments. In contrast to the autumn sampling, the concentrations of almost all nutrients except carbon (C), copper (Cu), calcium (Ca), and manganese (Mn) were reduced in organic farming practice (**Table 2**) at the second sampling in spring of the following year. Based on a two-way ANOVA, the BMc treatment showed a significant effect on the majority of nutrients in both farming practices, with the exception of zinc (Zn) (**Supplementary Table 3**). However, a pairwise comparison across all four treatments revealed that the BMc treatment significantly increased only the phosphorus (P) concentration in organically grown plants and the sulphur (S) concentration in conventionally grown plants, in comparison to the control (**Table 2**). The soil properties were not affected by BMc treatment, but were mainly influenced by the farming practice (**Supplementary Table 4**).

**Table 2:** Nutrient status of winter rye (*cv.* Conduct) grown under different long-time farming practices and use of beneficial microorganisms (control vs. BMc) in autumn and spring of the same growing season.

		Autumn									Spring							
		Conventional					Organic				Conventional				Organic			
	DT^#^	Ctrl			BMc		Ctrl		BMc		Ctrl		BMc		Ctrl		BMc	
Macro-nutrients [g kg^-1^ shoot DM]																		
C		411		a	407	a	400	a	390	a	331	B	367	AB	358	AB	391	A
N	25	27.0		b	30.7	ab	31.9	a	33.8	a	34.2	A	40.5	A	20.3	B	25.0	B
P	3.0	5.73		a	5.56	a	5.74	a	5.69	a	4.07	A	4.61	A	3.34	B	4.04	A
K	28	28.3		a	28.0	a	29.8	a	30.1	a	20.9	AB	23.9	A	16.4	B	20.7	AB
Mg	1.5	1.91		a	2.03	a	1.79	a	1.83	a	1.96	A	2.19	A	1.30	B	1.45	B
Ca	3.5	2.92		a	3.09	a	2.86	a	2.87	a	2.82	AB	3.30	A	2.76	B	3.00	AB
S	1.0	2.26		a	2.41	a	2.48	a	2.54	a	2.58	B	3.18	A	1.63	C	1.92	C
Micro-nutrients [mg kg^-1^ shoot DM]																		
Cu	6	6.80		a	6.84	a	6.72	a	6.88	a	5.48	A	6.04	A	5.42	A	5.97	A
Mn	25	122		ab	128	a	100	b	113	ab	209	A	180	AB	180	AB	141	B
Zn	20	29.7		a	31.6	a	26.4	a	30.4	a	34.5	A	34.2	A	20.4	B	20.4	B

^#^Deficiency threshold (DT) of macro- and micro-nutrients after Bergmann (1988).

Different letters indicate significant differences calculated by the Sidak-test (*p* ≤ 0.05). The type of letter indicates a separate statistical analysis (lowercase: autumn, uppercase: spring) (BMc, Beneficial Microorganism Consortium; Ctrl, Control; DM, Dry Mass).

### Bacterial community in the rhizosphere

**Figure 3:**
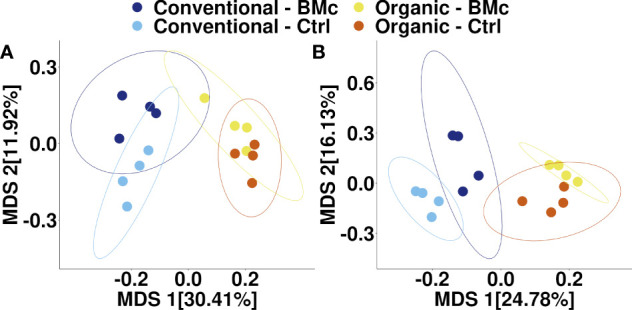
Multidimensional scaling (MDS) of bacterial community composition associated with winter rye rhizosphere under different farming practices and BMc inoculation treatment sampled in autumn **(A)** and spring **(B)**. The ellipses represent a 95% confidence interval. (BMc, Beneficial Microorganism Consortium; Ctrl, Control).

To elucidate whether the choice of farming practice and BMc inoculation influenced the composition of the winter rye rhizosphere bacterial communities, we performed 16S rRNA gene amplicon sequencing. The farming practice strongly influenced the composition of rhizosphere bacterial communities in the autumn (PERMANOVA, R^2^ = 0.29, *p* < 0.001, **Figure 3A**) and in the spring sampling (PERMANOVA, R^2^ = 0.23, *p* < 0.001, **Figure 3B**). Similarly, BMc inoculation significantly influenced the bacterial community composition of the rhizosphere in both samplings (PERMANOVA, R^2^ = 0.10-0.13, *p* < 0.001, **Figure 3**). However, we did not detect any interaction effect between the choice of farming practice and the BMc inoculation (PERMANOVA, R^2^ = 0.04-0.05). Combining the two variables and performing pairwise PERMANOVA tests showed that all treatment combinations significantly differed in the autumn sampling (**Table 3**). Similarly, most groups significantly differed in the spring sampling, except for the Organic-BMc and Organic-Control combination (**Table 3**). While both farming practices and BMc inoculation influenced bacterial community composition, they did not consistently affect α-diversity. Specifically, farming practice significantly affected ASV richness, Shannon index, and Evenness in the spring but not in the autumn sampling (**Supplementary Figure 2**, **Supplementary Table 5**). Irrespective of the sampling time, BMc inoculation did not influence bacterial α-diversity and no interaction effect between the two experimental factors was detected (**Supplementary Figure 2**).

**Table 3:** Pairwise PERMANOVA tests of the combined treatments (farming practices: conventional or organic, use of beneficial microorganisms: BMc or Control) with Benjamini-Hochberg correction (n=4).

Treatment Combination	R^2^	Adjusted *p-*value
Autumn		
Conventional-Control vs. Conventional-BMc	0.23	0.029
Conventional-Control vs. Organic-Control	0.39	0.029
Conventional-Control vs. Organic-BMc	0.40	0.029
Conventional-BMc vs. Organic-Control	0.42	0.029
Conventional-BMc vs. Organic-BMc	0.36	0.029
Organic-Control vs. Organic-BMc	0.19	0.029
Spring		
Conventional-Control vs. Conventional-BMc	0.23	0.034
Conventional-Control vs. Organic-Control	0.34	0.034
Conventional-Control vs. Organic-BMc	0.44	0.034
Conventional-BMc vs. Organic-Control	0.31	0.034
Conventional-BMc vs. Organic-BMc	0.29	0.034
Organic-Control vs. Organic-BMc	0.20	0.057

**Figure 4:**
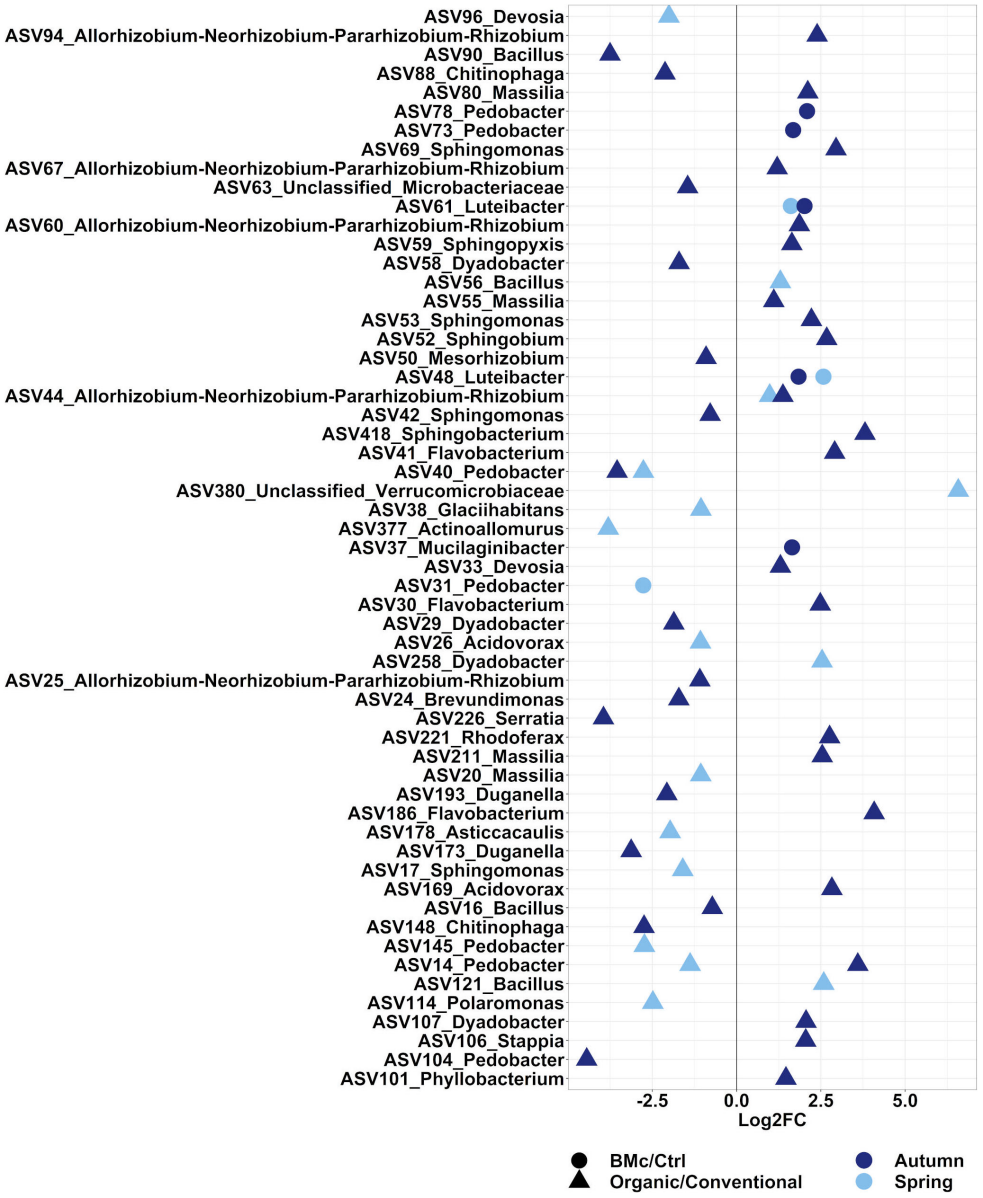
Log_2_ fold-change (FC) of ASVs that significantly differed and occurred in relative abundance higher than 0.5%. The comparisons were performed between farming practices (Organic *vs.* Conventional) or BMc inoculation (BMc *vs.* Ctrl) in the autumn or the spring sampling campaign during the growth of winter rye plants. Differential abundance testing was performed on rarefied datasets via ANCOM-BC2 with Benjamini-Hochberg correction (adjusted *p-*value<0.05).

We aimed to evaluate the ASVs that were affected by either the choice of the farming practice and/or the BMc inoculation. In total, 371 ASVs in the autumn and 273 ASVs in the spring sampling significantly differed between the two farming practices (**Supplementary Table 6**, ANCOM-BC2). Out of these differential abundant ASVs, 38 ASVs in the autumn and 16 ASVs in the spring sampling were representative of the bacterial communities investigated as they showed a relative abundance above 0.5% (**Figure 4**). Dominant discriminant ASVs with the highest differential abundance due to choices of farming practice included ASVs taxonomically related to the *Rhizobium group* (ASV44), *Sphingobacterium* spp. (ASV418), *Sphingomonas* spp. (ASV17, ASV42, ASV53 and ASV69) and *Pedobacter* spp. (ASV14, ASV40, ASV104, ASV140 and ASV145). Two dominant ASVs belonging to *Pedobacter* spp. (ASV14 and ASV40) and one dominant ASV belonging to *Rhizobium* spp. (ASV44) differed between farming practices independent of the sampling time (**Supplementary Table 6**). Specifically, the relative abundance of ASV44 (*Rhizobium* spp.) increased, while the relative abundance of ASV40 (*Pedobacter* spp.) decreased due to organic farming practice (**Figure 4**). In contrast, the ASV14 (*Pedobacter* spp.) showed opposite trends between the two samplings under organic farming, with an increase in abundance in autumn and a decline in spring. The BMc inoculation altered the relative abundance of 21 ASVs in the autumn and 14 ASVs in the spring sampling (**Supplementary Table 6**), with five ASVs in the autumn and three ASVs in the spring accounting for more than 0.5% of relative abundance (**Figure 4**). Two ASVs classified as *Luteibacter* spp. (ASV48 and ASV61) consistently occurred in higher relative abundance in the BMc inoculated samples. Other ASVs that increased due to BMc inoculation belonged to *Mucilaginibacter* spp. (ASV37) and *Pedobacter* spp. (ASV78, ASV73). Moreover, one dominant ASVs, which belonged to *Pedobacter* spp. (ASV31) was negatively affected by BMc inoculation in the spring sampling.

We were then interested to elucidate ASVs that significantly differed between each treatment combination. In total, 304 and 236 ASVs were found significantly different between the treatment combinations in the autumn and spring samplings, respectively (**Supplementary Table 7**), with 43 ASVs significantly differing independent from the sampling time (**Figure 4**, **Supplementary Table 8**). The logistic regression models revealed 277 (autumn sampling) and 210 (spring sampling) ASVs as representative predictors for each specific treatment combination (**Supplementary Table 9**). Of these ASVs, the relative abundance of 30 and 46 ASVs increased in the BMc-inoculated samples under organic fertilization in autumn and spring, respectively (**Supplementary Table 9**), where significantly higher SDM was detected (**Figure 2A**). In contrast, in the organic farming, BMc inoculation resulted in a decreased relative abundance of 57 ASVs at the autumn sampling and 19 ASVs at the spring sampling (**Supplementary Table 9**). Furthermore, three ASVs consistently responded to the BMc-inoculation in organic treatment in each sampling campaign (**Supplementary Table 10**).

**Figure 5:**
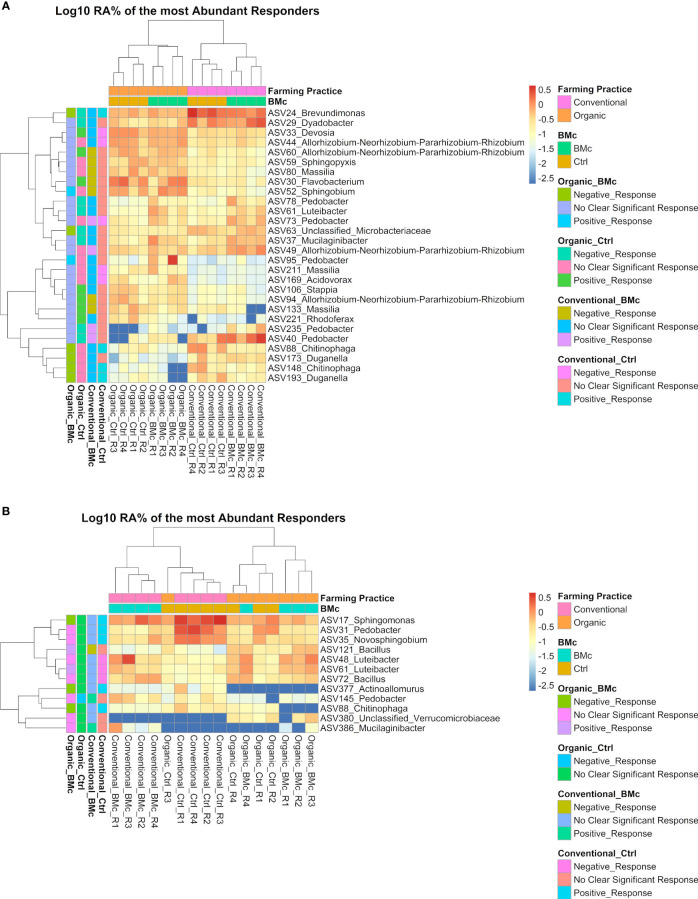
Heatmaps with the log_10_ transformed mean relative abundance of differential abundant ASVs in the autumn **(A)** and spring **(B)** sampling. The presented ASVs occurred in relative abundance higher than 0.5% in at least one sample. Significantly different ASVs were identified via ANCOM-BC2 (adjusted *p*-value via Benjamini-Hochberg <0.05, n=4). The colored legend (left side) shows whether these ASVs responded positively or negatively to a particular treatment combination (Organic-Ctrl, Organic-BMc, Conventional-Ctrl, Conventional-BMc). The responder ASVs were identified via logistic regression (adjusted *p*-value via Benjamini-Hochberg < 0.05). RA, Relative abundance.

Out of these differentially abundant ASV between the four treatment combinations, 28 and 12 ASVs exceeded the relative abundance of 0.5% in at least one sample in the autumn (**Figure 5A**) and the spring samplings, respectively (**Figure 5B**). From these dominant discriminant ASVs, 13 ASVs associated either positively or negatively with the BMc inoculation in the organic treatment. Negative responder ASVs were affiliated to *Chitinophaga* spp. (ASV148 and ASV88), *Duganella* spp. (ASV173 and ASV193), *Brevundimonas* spp. (ASV24), *Microbacteriaceae* spp. (ASV63), *Sphingomonas* spp. (ASV17), and *Actinoallomurus* spp. (ASV377), while positive responders were affiliated with *Sphingobium* spp. (ASV52), *Bacillus* spp. (ASV121), *Pedobacter* spp. (ASV95) and *Luteibacter* spp. (ASV61; only spring). Most notably, ASV88 (*Chitinophaga* sp.) was the single top abundant, significantly different ASV that negatively responded to BMc inoculation in the organic treatment at both sampling time points (**Figure 5**). Some of these ASVs were also associated with other treatments, especially in the control of conventional farming. For example, the ASV88 (*Chitinophaga* spp.) was enriched in the control samples under conventional farming and depleted with BMc inoculation in the organic treatment at both sampling time points. However, in the spring sampling, ASV61 (*Luteibacter* spp.) responded positively to BMc inoculation in the organic treatment and negatively to control in conventional farming (**Figure 5**).

**Figure 6:**
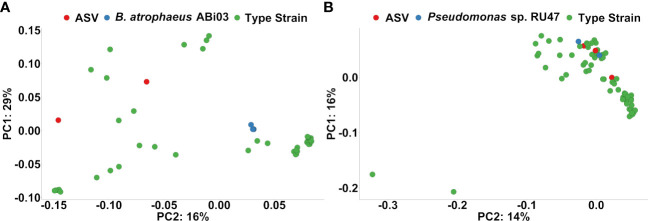
**(A)** Ordination plots based on phylogenetic distance following pairwise local alignment of 16S rRNA gene sequence from the ASV responders to Organic-BMc that were classified as *Bacillus* spp., 16S rRNA genes from *Bacillus atrophaeus* ABi03 and *Bacillus* spp. from the type strain collection of Genbank (Red: ASV responders, Blue: *Bacillus atrophaeus* ABi03, Green: *Bacillus* spp. type strains). The plot shows no indication that these ASV responders classified as *Bacillus* spp. could belong to *Bacillus* ABi03. **(B)** Ordination plots based on phylogenetic distance following pairwise local alignment of 16S rRNA gene sequence from the ASV responders to Organic-BMc that were classified as *Pseudomonas* spp., the strain *Pseudomonas* sp. RU47 (six 16S rRNA genes) and *Pseudomonas* spp. from the type strain collection of Genbank (Red: ASV responders, Blue: *Pseudomonas* sp. RU47, Green: *Pseudomonas* spp. type strains). The plot indicates that two ASV responders (ASV1123 and ASV1576) probably belong to the inoculum strain *Pseudomonas* sp. RU47, since they cluster with the 16S rRNA genes of the inoculum strain *Pseudomonas* sp. RU47.

Finally, we performed a phylogenetic clustering analysis to estimate whether ASV responders to the BMc inoculation in the organic treatment were associated phylogenetically with the inoculated BM strains (**Figure 6**). ASV responders classified as *Bacillus* spp. clustered more closely to other *Bacillus* spp. than the 16S rRNA genes of *B. atrophaeus* ABi03 (**Figure 6A**). In contrast, two out of the three ASVs classified as *Pseudomonas* spp. (ASV1123 and ASV1576), were clustered together with the six 16S rRNA gene copies of *Pseudomonas* sp. RU47 (**Figure 6B**). These two ASVs were positively associated with the BMc inoculation in the organic treatment in the autumn sampling but not in the spring sampling (**Supplementary Figure 3**).

## Discussion

### Successful establishment of the microbial consortium throughout winter dormancy

In this study, we hypothesized that each consortium member would colonize the rhizosphere of winter rye plants and maintain a high density over the winter dormancy independent of farming practices (organic vs. conventional). The three consortium members were selected based on their previously observed positive effects on plant performance. *Pseudomonas* sp. RU47 strain enhanced growth of tomato and maize plants in P-deficient soils (Eltlbany et al., 2019) and suppressed bottom rot (*Rhizoctonia solani*) in lettuce very efficiently under greenhouse and field conditions, although no strong antagonistic properties were found in *in vitro* assays (Adesina et al., 2009; Schreiter et al., 2018). We assumed that the disease suppression effect was mainly based on the production of HCN (Kuzmanović et al., 2018). In contrast, the *Bacillus atrophaeus* ABi03 strain showed various antagonistic functions *in vitro*. Plant growth promotion was also observed in consortia containing the *Trichoderma harzianum* OMG16 strain in tomato (Mpanga et al., 2018) and maize (Mpanga et al., 2019; Moradtalab et al., 2020). Furthermore, OMG16 reduced root infection by *Verticillium longisporum* in rapeseed as well (Hafiz et al., 2022). In this study, the strains with different modes of action were applied as a consortium in the field. Several studies have shown that using a consortium of two or more microorganisms can have a stronger and more sustainable effect on plant health and performance than a single beneficial microorganism (Sharma et al., 2020; Hafiz et al., 2022). Additive or synergistic effects can, in part, be attributed to separate habitats such as the rhizosphere, the root-associated soil, and the root cortex but also to different beneficial functions such as improved nutrient availability, phytohormone production, antagonistic functions against plant pathogens and (a)biotic stress mitigation (Jha and Saraf, 2012; De Vrieze et al., 2018; Gu et al., 2020; Santoyo et al., 2021).

A long-term coexistence between the members of a BMc in the rhizosphere or root-associated soil is crucial to ensure beneficial effects on plants in short and long periods. Seven weeks after inoculation (autumn sampling), all members of the consortium colonized the winter rye roots in a similar density compared to previous studies (Schreiter et al., 2018; Jamil et al., 2021), indicating that the combined application of the beneficial microorganisms as a consortium did not impair their rhizosphere competence. The bacterial strains ABi03 and RU47 were mainly detected in the rhizosphere and did not inhibit each other as observed in dual culture (data not shown), whereas OMG16 inhabits especially the root-associated soil but was also previously reported as an endophyte in rapeseed (Hafiz et al., 2022). Because of this spatial separation, potential antagonistic functions demonstrated *in vitro* for RU47 and ABi03 (**Table 1**) can likely not act against OMG16. Besides direct competition, the establishment of applied microorganisms is highly dependent on the composition and structure of the indigenous microbial community, as demonstrated by previous studies that linked a low rhizosphere competence of applied microorganisms to a high microbial diversity, which acts as a barrier against external invaders (Schierstaedt et al., 2020; Mawarda et al., 2022). The successful establishment of inoculated microorganisms within the rhizosphere has been linked to rapid nutrient utilization, biofilm formation, but also antagonistic capabilities (Adam et al., 2016; Kumawat et al., 2019). *In vitro* demonstrated antagonistic functions of all consortium members may facilitate their selective occupation of niches within the rhizosphere, thus promoting their integration into the microbial community (Berg et al., 2021).

Although our results (**Figure 3**) confirm that farming practice drives the composition of microbial communities in the soil and rhizosphere (Schrama et al., 2018; Babin et al., 2019; Bziuk et al., 2021; Fernandez-Gnecco et al., 2022), the BMc rhizosphere competence was not affected, indicating a robust competence of the consortium members at different environmental conditions. Since only a few responder ASVs consistently differed between the farming practices at both sampling time points, we presume that the assembly of rhizosphere microbiota was a stochastic process influenced by environmental conditions and plant developmental stages. The continuous time-dependent stochastic assembly of bacterial communities in the rhizosphere could have contributed to the high rhizosphere competence of the consortium members independent of farming practices. In spring, the colonization density of RU47 in the rhizosphere decreased overall by a factor of 10, whereas the densities of ABi03 and OMG16 (root-associated soil) remained almost the same compared to the autumn sampling. The higher resilience of ABi03 and OMG16 can be attributed to their potential for sporulation, which allows higher survival under adverse winter conditions compared to RU47 (Fernández-Sandoval et al., 2012; Sella et al., 2014). Nevertheless, all three microorganisms colonized the rhizosphere of winter rye sufficiently, even 22 weeks after inoculation. This indicates a strong positive feedback between the plant and the members of the consortium since the persistence of a high colonization density depends on the one side on the root exudation profile of the plant and on the other side on the chemotaxis of each consortium member towards these metabolites and their use as a substrate (Malgioglio et al., 2022).

### Consortium improved plant performance and nutrient acquisition under organic farming

Shoot growth of winter rye was promoted by BMc inoculation in autumn (+20%), but especially during regrowth after winter (+45%) exclusively under organic farming. These findings are in line with the robust rhizosphere competence shown by the investigated inoculants, which remained unaffected even during unfavorable winter conditions. Moreover, under long-time organic farming practice, BMc inoculation was associated with the enrichment of bacterial taxa with documented plant growth promoting properties. As a consequence, organic farming practice may support soil biological processes that allow more complex taxonomic and functional communities (Bender et al., 2016). Numerous studies have consistently reported positive effects on beneficial plant-microbe interactions when using manure-based fertilizers alongside inoculants such as *Bacillus*, *Pseudomonas*, and *Trichoderma* (Thonar et al., 2017; Mpanga et al., 2018; Bradáčová et al., 2019). A beneficial plant-microbe interaction under organic farming might improve the carbon supply of fast-growing copiotrophic inoculants as well as indigenous plant growth-promoting microorganisms, thereby supporting the establishment of a beneficial microbial community. This effect may be of particular importance especially on light sandy soils characterized by low organic carbon content, which were observed in this study even after long-time organic farming practice (**Supplementary Table 4**). Accordingly, a meta-analysis reported that responsiveness to microbial inoculants decreases with increasing soil organic carbon content (Schütz et al., 2018). Furthermore, the high availability of N and P, which is characteristic of manure-based fertilizers, can serve as a starter fertilization for the host plant, facilitating root growth and the establishment of microbial inoculants in the rhizosphere (Bittman et al., 2006; Chekanai et al., 2018).

The beneficial effects of inoculants and their impact on the rhizosphere microbiome can in part be attributed to improved plant nutrient supply. During autumn, no significant differences in shoot nutrient concentrations were initially observed among the treatments reflecting a comparable nutritional status (**Table 2**). However, ANOVA revealed a general impact of the BMc inoculation on the N concentration of the shoots (**Supplementary Table 3**). Nutrient deficiencies were only apparent for Ca (Bergmann, 1988) and may be attributed to low Ca levels of the light sandy soil at the field site (**Supplementary Table 4**). In contrast, during regrowth after winter, critical nutrient concentrations were recorded, particularly under organic fertilization, including N, P, K, Mg, Ca, Cu and Zn. BMc inoculation significantly improved the P nutritional status, which is a critical nutrient in many organic farming systems (Cooper et al., 2018). However, similar trends were also recorded for all remaining nutrients except Zn and Mn (**Table 2**). An improved plant nutritional status due to BMc inoculation is also supported by ANOVA, which shows a general effect of BMc inoculation on the concentration of these nutrients independent of farming practice (**Supplementary Table 3**). As a consequence, nutrient concentrations of BMc inoculated plants reached or even exceeded the sufficiency thresholds particularly for N and P. These findings suggest a general positive effect of BMc on the nutrient acquisition, possibly mediated through the stimulation of root growth, which has been well-documented for the inoculant strains (Mpanga et al., 2018; Eltlbany et al., 2019; Moradtalab et al., 2020). However, improved plant nutrient acquisition through direct nutrient mobilizing by the BMc is a less likely scenario, although *in vitro* tests did detect solubilization of P and K. Analysis of the root-associated nutrient pools in the soil did not show any changes following the BMc inoculation, suggesting no specific effects on solubilization of available soil nutrients in spring (**Supplementary Table 4**). According to current literature, P-solubilizing microorganisms contribute to the host plant nutrition primarily through their long-term impact on nutrient cycling, rather than direct nutrient solubilization (Raymond et al., 2021). Interestingly, similar trends of an improved nutrient status after BMc inoculation were also shown for plants grown under conventional farming. However, compared to organic farming, conventional farming practice generally resulted in a higher nutritional status of the plants, reaching sufficiency levels for N, P, and Mg, even in non-inoculated plants. This may explain the absence of additional growth responses by BMc inoculation under conventional farming.

Although there were no significant differences among the treatments, the final yield data (**Figure 2B**) corresponded with the effects of the shoot biomass during spring vegetative growth (**Figure 2A**). The grain yield under long-time conventional farming practices was slightly higher than the 2015-2020 average rye yields (4.1 t ha^-1^) in the district of Brandenburg (Germany). Organic farming practice tended to decrease yield by approx. 22%, similar to the reported average yield losses determined by meta-studies (de Ponti et al., 2012; Seufert et al., 2012). Although not significant, our results indicate that this yield decline by organic farming may partially be compensated by BMc application.

### Inoculation-dependent modulation of rhizosphere bacterial community persists over winter dormancy

We hypothesized that the application of the BMc would affect the bacterial community composition in the rhizosphere depending on farming practice. Consequently, we anticipated that these variations would exert differential influences on the performance of winter rye. An alteration of the bacterial community composition in the rhizosphere of winter rye was found in the BMc inoculated treatments, regardless of farming practices. This effect persisted until spring, indicating a prolonged impact of the BMc (**Figure 3**). Similar results were observed in a study by Deng et al. (2019), where BMc inoculation affected the bacterial community composition of strawberry roots over various time points, irrespective of farming practices. This indicates that repeated application of the inoculants might not be necessary, once their effects have been established. However, further time-series experiments are needed to clarify whether the effects of BMc on the rhizosphere microbiota and plant performance persist throughout the growing season at the field scale.

In addition to BMc inoculation, we observed an effect of the farming practices on the soil microbiota, similar to our previous studies (Bziuk et al., 2021; Windisch et al., 2021; Fernandez-Gnecco et al., 2022; Sommermann et al., 2022). However, this is the first time we report that this effect persists across different plant developmental stages. While both farming practices and BMc inoculation significantly affected bacterial community composition, only a few ASVs exhibited consistent differential abundance patterns over time (**Figure 4**). This indicates a) that the recruitment of microorganisms in the rhizosphere differed over the developmental stages of the plant and b) that the BMc inoculation and the farming practices influenced this recruitment. A similar influence of farming practices on the recruitment of microorganisms in the rhizosphere has been demonstrated previously (Windisch et al., 2021; Sommermann et al., 2022). In summary, the BMc inoculation influence on rhizosphere microbiota persisted over the winter dormancy, indicating specific strategies such as spore formation by the consortium members to survive during winter time. In addition, changes in the root exudation during spring could have further activated dormant BMs (Windisch et al., 2017).

According to ecological theories on plant-microbe interactions, the mutualistic selection of plant beneficial microorganisms often emerges under nutrient limitations (Sánchez-Cañizares et al., 2017). Since chemical fertilizers provide a high availability of nutrients to plant and rhizosphere microbiota, they could interfere with the plant’s selection processes for beneficial microorganisms (Sánchez-Cañizares et al., 2017). Interestingly, we observed increased growth performance of plants inoculated with the BMc only under organic farming, where nutrient availability was low (**Supplementary Table 4**). We believe that this additive effect could be associated with a higher selection pressure on both the plants and soil microorganisms due to nutrient limitations, in contrast to conventional farming with high nutrient availability. However, CFU data indicated that the farming practices negligibly altered the density of the applied BMc in the soil (**Figure 1**). Herein, the differential abundance testing revealed an enrichment of ASVs closely related to *Pseudomonas* spp. or *Bacillus* spp., which may correspond to our BMc inoculants. By applying phylogenetic clustering, we identified two *Pseudomonas* ASVs closely related to *Pseudomonas* sp. RU47 (**Figure 6**). The higher proportions (relative abundance) of *Pseudomonas* sp. RU47 under organic farming and BMc inoculation, might indicate less competition from other bacterial taxa inhabiting the rhizosphere in organic farming. The interaction effect between farming practices and BMc inoculation on the rhizosphere bacterial community profiles was weak. Thus, the two farming practices only slightly influenced the ability of BMc to modify the rhizosphere bacterial community. Nevertheless, we attempted to identify ASVs associated with BMc inoculation under organic farming to determine which fraction of bacterial taxa are associated with the inoculated BMc. Several ASV responders were detected, but most had a relative abundance below 0.5%.

Among the dominant responders (>0.5% relative abundance), several ASVs closely classified as known potential plant-beneficial bacteria increased in relative abundance in the rhizosphere of winter rye (**Figure 5**). For instance, members of the genus *Luteibacter* (which belong to γ-*Proteobacteria*) are known for their plant-growth-promoting traits (Guglielmetti et al., 2013; Hoffman et al., 2013) and were enriched in the rhizosphere of the organic treatment. Interestingly, *Luteibacter* spp. can act as symbionts to fungal plant endophytes and trigger the production of IAA, which promotes plant growth. Similarly, one ASV classified as *Pedobacter* spp. (ASV95), a genus that includes several plant-beneficial species, increased in relative abundance due to BMc inoculation under organic farming (Morais et al., 2019). While the combination of BMc inoculation and organic farming might not substantially affect the overall profile of the bacterial community, it increased the relative abundance of bacterial taxa that potentially act as plant-beneficial microorganisms promoting plant growth. However, further field studies are needed to confirm whether the microbial responders due to BMc inoculation also contributed to increased plant growth.

Among the dominant responders with decreasing relative abundance due to BMc inoculation, primarily in organic farming, we identified an ASV classified as *Chitinophaga* sp. or *Duganella* sp. These ASVs have been previously associated with the onset of plant diseases (Carrión et al., 2019; Li et al., 2021). Organic farming practice and the BMc combination probably reduced bacterial taxa associated with plant diseases and stressful conditions. In contrast, some of these ASVs had higher relative abundance in conventional farming practice without BMc inoculation, indicating that the high input of nutrients through chemical fertilization might have led to the accumulation of bacterial taxa associated with plant diseases. Alternatively, plants often recruit several closely taxonomically-related bacteria in response to a pathogen (e.g., *Duganella* spp.) (Haack et al., 2016), so direct association of these taxa with plant pathogenicity is not possible, especially since no plant disease phenotype was detected.

## Conclusion

This study illustrated the effects of a beneficial microorganism consortium (BMc) on the performance of a winter rye depending on different farming strategies, i.e. conventional and organic farming. The main goal of our study was to assess the ability of the consortium members to survive in a sufficient density in the rhizosphere/root-associated soil of winter rye as prerequisite for successful plant-microorganism interaction. Our findings demonstrated that the consortium members persisted in the rye rhizosphere over the vegetation period, maintaining a high density even after winter dormancy independent of the farming practice. As expected, the BMc had a positive effect on the rye performance, increasing shoot dry biomass and plant nutritional status especially under organic fertilization. These findings further confirmed the *in vitro* tested plant-promoting features that characterized the microbial members inoculated in the rhizosphere of winter rye under field conditions. Moreover, the inoculated BMc had a significant effect on the bacterial community dynamics as large shifts in rhizosphere bacterial assembly were observed after inoculation in both organic and conventional treatments, and such community shifts were also detected across different plant developmental stages. Interestingly, under organic farming we observed that in the rye rhizosphere, treated with BMc, several bacterial taxa previously associated with plant diseases were depleted, while several putative plant beneficial bacterial taxa were enriched in their relative abundance, thus further highlighting the positive effects of the BMc on the plant health by modulating the rye rhizosphere microbiome. It is noteworthy that although our field experiment indicated a positive effect of BMc on rye performance, especially under organic fertilization, the beneficial impact on rye yield was only marginal. Nevertheless, our results indicate that BMc inoculation might have the potential to compensate yield losses caused by nutrient limitation in organic farming practice. Furthermore, BMc treatment may contribute to a better yield stability by improving plant nutrient status and promoting beneficial microbiota, thus representing a sustainable approach especially in combination with organic farming. Our research emphasizes the importance of conducting additional field experiments to understand the efficacy of microbial inoculants in various cropping systems, promoting the development of efficient microbe-based solutions for sustainable agriculture.

## Data availability statement

The datasets presented in this study can be found in online repositories. The names of the repository/repositories and accession number(s) can be found below: https://www.ncbi.nlm.nih.gov/bioproject/PRJNA975889.

## Author contributions

Conceptualization: JB, RG, GN, JG, KS. Experiment conduct: JB. Methodology: JB, IK, DB. Validation: JB, IK. Formal analysis: JB, IK. Data curation: JB, IK. Visualization: JB, IK. Writing—original draft preparation: JB, IK. Writing—review and editing: RG, DB, LS, DF, TK-N, SC, JG, GN, KS. Supervision: RG. Project administration: RG. All authors have read and agreed to the published version of the manuscript. All authors contributed to the article and approved the submitted version.

## References

[B1] Adam E. Groenenboom A. E. Kurm V. Rajewska M. Schmidt R. Tyc O.. (2016). Controlling the microbiome: microhabitat adjustments for successful biocontrol strategies in soil and human gut. Front. Microbiol. 7. doi: 10.3389/fmicb.2016.01079 PMC4942455 27468279

[B2] Adesina M. F. Grosch R. Lembke A. Vatchev T. D. Smalla K. (2009). *In vitro* antagonists of *Rhizoctonia solani* tested on lettuce: rhizosphere competence, biocontrol efficiency and rhizosphere microbial community response. FEMS Microbiol. Ecol. 69, 62–74. doi: 10.1111/j.1574-6941.2009.00685.x 19486156

[B3] Ali S. Hameed S. Shahid M. Iqbal M. Lazarovits G. Imran A. (2020). Functional characterization of potential PGPR exhibiting broad-spectrum antifungal activity. Microbiol. Res. 232, 126389. doi: 10.1016/j.micres.2019.126389 31821969

[B4] Babin D. Deubel A. Jacquiod S. Sørensen S. J. Geistlinger J. Grosch R.. (2019). Impact of long-term agricultural management practices on soil prokaryotic communities. Soil Biol. Biochem. 129, 17–28. doi: 10.1016/j.soilbio.2018.11.002

[B5] Batista B. D. Singh B. K. (2021). Realities and hopes in the application of microbial tools in agriculture. Microb. Biotechnol. 14, 1258–1268. doi: 10.1111/1751-7915.13866 34156754 PMC8313292

[B6] Bender S. F. Wagg C. van der Heijden M. G. A. (2016). An underground revolution: biodiversity and soil ecological engineering for agricultural sustainability. Trends Ecol. Evol. 31, 440–452. doi: 10.1016/j.tree.2016.02.016 26993667

[B7] Berg G. Fritze A. Roskot N. Smalla K. (2001). Evaluation of potential biocontrol rhizobacteria from different host plants of *Verticillium dahliae* Kleb. J. Appl. Microbiol. 91, 963–971. doi: 10.1046/j.1365-2672.2001.01462.x 11851803

[B8] Berg G. Krechel A. Ditz M. Sikora R. A. Ulrich A. Hallmann J. (2005). Endophytic and ectophytic potato-associated bacterial communities differ in structure and antagonistic function against plant pathogenic fungi. FEMS Microbiol. Ecol. 51, 215–229. doi: 10.1016/j.femsec.2004.08.006 16329870

[B9] Berg G. Kusstatscher P. Abdelfattah A. Cernava T. Smalla K. (2021). Microbiome modulation—Toward a better understanding of plant microbiome response to microbial inoculants. Front. Microbiol. 12. doi: 10.3389/fmicb.2021.650610 PMC8060476 33897663

[B10] Berg G. Smalla K. (2009). Plant species and soil type cooperatively shape the structure and function of microbial communities in the rhizosphere. FEMS Microbiol. Ecol. 68, 1–13. doi: 10.1111/j.1574-6941.2009.00654.x 19243436

[B11] Bergmann W. (Ed.) (1988). Ernährungsstörungen bei Kulturpflanzen (Stuttgart: G. Fischer).

[B12] Bittman S. Kowalenko C. G. Hunt D. E. Forge T. A. Wu X. (2006). Starter phosphorus and broadcast nutrients on corn with contrasting colonization by mycorrhizae. Agron. J. 98, 394–401. doi: 10.2134/agronj2005.0093

[B13] Bodenhofer U. Bonatesta E. Horejš-Kainrath C. Hochreiter S. (2015). msa: an R package for multiple sequence alignment. Bioinformatics 31, btv494. doi: 10.1093/bioinformatics/btv494 26315911

[B14] Bradáčová K. Florea A. Bar-Tal A. Minz D. Yermiyahu U. Shawahna R.. (2019). Microbial consortia versus single-strain inoculants: an advantage in PGPM-assisted tomato production? Agronomy 9, 105. doi: 10.3390/agronomy9020105

[B15] Breitkreuz C. Reitz T. Schulz E. Tarkka M. (2021). Drought and plant community composition affect the metabolic and genotypic diversity of pseudomonas strains in grassland soils. Microorganisms 9, 1677. doi: 10.3390/microorganisms9081677 34442756 PMC8399733

[B16] Bziuk N. Maccario L. Douchkov D. Lueck S. Babin D. Sørensen S. J.. (2021). Tillage shapes the soil and rhizosphere microbiome of barley—but not its susceptibility towards *Blumeria graminis* f. sp. *hordei* . FEMS Microbiol. Ecol. 97, fiab018. doi: 10.1093/femsec/fiab018 33544837

[B17] Callahan B. J. McMurdie P. J. Rosen M. J. Han A. W. Johnson A. J. A. Holmes S. P. (2016). DADA2: High-resolution sample inference from Illumina amplicon data. Nat. Methods 13, 581–583. doi: 10.1038/nmeth.3869 27214047 PMC4927377

[B18] Cameron E. S. Schmidt P. J. Tremblay B. J.-M. Emelko M. B. Müller K. M. (2021). Enhancing diversity analysis by repeatedly rarefying next generation sequencing data describing microbial communities. Sci. Rep. 11, 22302. doi: 10.1038/s41598-021-01636-1 34785722 PMC8595385

[B19] Cappuccino J. G. Sherman N. (2014). Microbiology: A Laboratory Manual. 10th ed (Boston: Pearson).

[B20] Carrión V. J. Perez-Jaramillo J. Cordovez V. Tracanna V. de Hollander M. Ruiz-Buck D.. (2019). Pathogen-induced activation of disease-suppressive functions in the endophytic root microbiome. Science 366, 606–612. doi: 10.1126/science.aaw9285 31672892

[B21] Chaparro J. M. Badri D. V. Vivanco J. M. (2014). Rhizosphere microbiome assemblage is affected by plant development. ISME J. 8, 790–803. doi: 10.1038/ismej.2013.196 24196324 PMC3960538

[B22] Charif D. Lobry J. R. (2007). “SeqinR 1.0-2: A contributed package to the R project for statistical computing devoted to biological sequences retrieval and analysis,” in Structural Approaches to Sequence Evolution Biological and Medical Physics, Biomedical Engineering. Eds. Bastolla U. Porto M. ROman H. E. Vendruscolo M. (Berlin, Heidelberg: Springer Berlin Heidelberg), 207–232. doi: 10.1007/978-3-540-35306-5_10

[B23] Chekanai V. Chikowo R. Vanlauwe B. (2018). Response of common bean (*Phaseolus vulgaris* L.) to nitrogen, phosphorus and rhizobia inoculation across variable soils in Zimbabwe. Agric. Ecosyst. Environ. 266, 167–173. doi: 10.1016/j.agee.2018.08.010 30393414 PMC6142818

[B24] Chowdhury S. P. Babin D. Sandmann M. Jacquiod S. Sommermann L. Sørensen S. J.. (2019). Effect of long-term organic and mineral fertilization strategies on rhizosphere microbiota assemblage and performance of lettuce. Environ. Microbiol. 21, 2426–2439. doi: 10.1111/1462-2920.14631 30990945 PMC6849853

[B25] Cock P. J. A. Grüning B. A. Paszkiewicz K. Pritchard L. (2013). Galaxy tools and workflows for sequence analysis with applications in molecular plant pathology. PeerJ 1, e167. doi: 10.7717/peerj.167 24109552 PMC3792188

[B26] Cooper J. Reed E. Y. Hörtenhuber S. Lindenthal T. Løes A.-K. Mäder P.. (2018). Phosphorus availability on many organically managed farms in Europe. Nutr. Cycling Agroecosyst. 110, 227–239. doi: 10.1007/s10705-017-9894-2

[B27] Deng S. Wipf H. M.-L. Pierroz G. Raab T. K. Khanna R. Coleman-Derr D. (2019). A plant growth-promoting microbial soil amendment dynamically alters the strawberry root bacterial microbiome. Sci. Rep. 9, 17677. doi: 10.1038/s41598-019-53623-2 31776356 PMC6881409

[B28] de Ponti T. Rijk B. van Ittersum M. K. (2012). The crop yield gap between organic and conventional agriculture. Agric. Syst. 108, 1–9. doi: 10.1016/j.agsy.2011.12.004

[B29] De Vrieze M. Germanier F. Vuille N. Weisskopf L. (2018). Combining different potato-associated pseudomonas strains for improved biocontrol of *phytophthora infestans* . Front. Microbiol. 9. doi: 10.3389/fmicb.2018.02573 PMC6215842 30420845

[B30] Donate-Correa J. León-Barrios M. Pérez-Galdona R. (2005). Screening for plant growth-promoting rhizobacteria in *Chamaecytisus proliferus* (tagasaste), a forage tree-shrub legume endemic to the Canary Islands. Plant Soil 266, 261–272. doi: 10.1007/s11104-005-0754-5

[B31] Eltlbany N. Baklawa M. Ding G.-C. Nassal D. Weber N. Kandeler E.. (2019). Enhanced tomato plant growth in soil under reduced P supply through microbial inoculants and microbiome shifts. FEMS Microbiol. Ecol. 95, fiz124. doi: 10.1093/femsec/fiz124 31386159

[B32] FC M. Davis T. (2022). ggpattern: “ggplot2” Pattern geoms. Available at: https://CRAN.R-project.org/package=ggpattern.

[B33] Federhen S. (2015). Type material in the NCBI taxonomy database. Nucleic Acids Res. 43, D1086–D1098. doi: 10.1093/nar/gku1127 25398905 PMC4383940

[B34] Fernandez-Gnecco G. Covacevich F. Consolo V. F. Behr J. H. Sommermann L. Moradtalab N.. (2022). Effect of long-term agricultural management on the soil microbiota influenced by the time of soil sampling. Front. Soil Sci. 2. doi: 10.3389/fsoil.2022.837508

[B35] Fernández-Sandoval M. T. Ortiz-García M. Galindo E. Serrano-Carreón L. (2012). Cellular damage during drying and storage of *Trichoderma harzianum* spores. Process Biochem. 47, 186–194. doi: 10.1016/j.procbio.2011.10.006

[B36] Finkel O. M. Castrillo G. Herrera Paredes S. Salas González I. Dangl J. L. (2017). Understanding and exploiting plant beneficial microbes. Curr. Opin. Plant Biol. 38, 155–163. doi: 10.1016/j.pbi.2017.04.018 28622659 PMC5561662

[B37] Fira D. Dimkić I. Berić T. Lozo J. Stanković S. (2018). Biological control of plant pathogens by *Bacillus* species. J. Biotechnol. 285, 44–55. doi: 10.1016/j.jbiotec.2018.07.044 30172784

[B38] Fox J. Weisberg S. (2019). An R companion to applied regression. 3rd ed (Thousand Oaks, CA: Sage). Available at: https://socialsciences.mcmaster.ca/jfox/Books/Companion/.

[B39] Francioli D. Schulz E. Lentendu G. Wubet T. Buscot F. Reitz T. (2016). Mineral vs. Organic amendments: microbial community structure, activity and abundance of agriculturally relevant microbes are driven by long-term fertilization strategies. Front. Microbiol. 7. doi: 10.3389/fmicb.2016.01446 PMC5022044 27683576

[B40] Graves S. Piepho H.-P. Selzer L. (2019). multcompView: visualizations of paired comparisons. Available at: https://CRAN.R-project.org/package=multcompView.

[B41] Gu S. Yang T. Shao Z. Wang T. Cao K. Jousset A.. (2020). Siderophore-mediated interactions determine the disease suppressiveness of microbial consortia. mSystems 5, e00811–e00819. doi: 10.1128/mSystems.00811-19 32606030 PMC7329327

[B42] Guglielmetti S. Basilico R. Taverniti V. Arioli S. Piagnani C. Bernacchi A. (2013). *Luteibacter rhizovicinus* MIMR1 promotes root development in barley (*Hordeum vulgare* L.) under laboratory conditions. World J. Microbiol. Biotechnol. 29, 2025–2032. doi: 10.1007/s11274-013-1365-6 23653264

[B43] Haack F. S. Poehlein A. Kröger C. Voigt C. A. Piepenbring M. Bode H. B.. (2016). Molecular Keys to the *Janthinobacterium* and Duganella spp. Interaction with the Plant Pathogen *Fusarium graminearum* . Front. Microbiol. 7, 7. doi: 10.3389/fmicb.2016.01668 27833590 PMC5080296

[B44] Hafiz F. B. Moradtalab N. Goertz S. Rietz S. Dietel K. Rozhon W.. (2022). Synergistic effects of a root-endophytic *trichoderma* fungus and *bacillus* on early root colonization and defense activation against *verticillium longisporum* in rapeseed. MPMI 35, MPMI–11-21-0274-R. doi: 10.1094/MPMI-11-21-0274-R 35147443

[B45] Heuer H. Kopmann C. Binh C. T. T. Top E. M. Smalla K. (2009). Spreading antibiotic resistance through spread manure: characteristics of a novel plasmid type with low %G+C content. Environ. Microbiol. 11, 937–949. doi: 10.1111/j.1462-2920.2008.01819.x 19055690

[B46] Hoffman M. T. Gunatilaka M. K. Wijeratne K. Gunatilaka L. Arnold A. E. (2013). Endohyphal bacterium enhances production of indole-3-acetic acid by a foliar fungal endophyte. PloS One 8, e73132. doi: 10.1371/journal.pone.0073132 24086270 PMC3782478

[B47] Hothorn T. Bretz F. Westfall P. (2008). Simultaneous inference in general parametric models. Biom. J. 50, 346–363. doi: 10.1002/bimj.200810425 18481363

[B48] Huber J. Bochow H. Junge H. (1987). Selektion und biotechnische Herstellung von Kulturlösungen mikrobieller Antagonisten zur Unterdrückung phytopathogener Bodenpilze. J. Basic Microbiol. 27, 497–503. doi: 10.1002/jobm.3620270907

[B49] Jacoby R. Peukert M. Succurro A. Koprivova A. Kopriva S. (2017). The role of soil microorganisms in plant mineral nutrition—Current knowledge and future directions. Front. Plant Sci. 8. doi: 10.3389/fpls.2017.01617 PMC5610682 28974956

[B50] Jamil A. Musheer N. Ashraf S. (2021). Antagonistic potential of *Trichoderma harzianum* and *Azadirachta indica* against *Fusarium oxysporum* f. sp. *capsici* for the management of chilli wilt. J. Plant Dis. Prot. 128, 161–172. doi: 10.1007/s41348-020-00383-1

[B51] Jha C. K. Saraf M. (2012). Evaluation of multispecies plant-growth-promoting consortia for the growth promotion of *jatropha curcas* L. J. Plant Growth Regul. 31, 588–598. doi: 10.1007/s00344-012-9269-5

[B52] Kay M. Elkin L. A. Higgins J. J. Wobbrock J. O. (2021). mjskay/ARTool: ARTool 0.11.0 (Zenodo). doi: 10.5281/ZENODO.594511

[B53] Koo S.-Y. Hong S. H. Ryu H. W. Cho K. (2010). Plant growth-promoting trait of rhizobacteria isolated from soil contaminated with petroleum and heavy metals. J. Microbiol. Biotechnol. 20, 587–593. doi: 10.4014/jmb.0907.07017 20372032

[B54] Kumawat K. C. Sharma P. Sirari A. Singh I. Gill B. S. Singh U.. (2019). Synergism of *Pseudomonas aeruginosa* (LSE-2) nodule endophyte with Bradyrhizobium sp. (LSBR-3) for improving plant growth, nutrient acquisition and soil health in soybean. World J. Microbiol. Biotechnol. 35, 35. doi: 10.1007/s11274-019-2622-0 30834977

[B55] Kuzmanović N. Eltlbany N. Ding G. Baklawa M. Min L. Wei L.. (2018). Analysis of the genome sequence of plant beneficial strain Pseudomonas sp. RU47. J. Biotechnol. 281, 281. doi: 10.1016/j.jbiotec.2018.07.023 30031092

[B56] Latz E. Eisenhauer N. Rall B. C. Scheu S. Jousset A. (2016). Unravelling linkages between plant community composition and the pathogen-suppressive potential of soils. Sci. Rep. 6, 23584. doi: 10.1038/srep23584 27021053 PMC4810420

[B57] Lenth R. (2022). emmeans: Estimated Marginal Means, aka Least-Squares Means. Available at: https://CRAN.R-project.org/package=emmeans.

[B58] Li H. Song F. Wu X. Deng C. Xu Q. Peng S.. (2021). Microbiome and metagenome analysis reveals huanglongbing affects the abundance of citrus rhizosphere bacteria associated with resistance and energy metabolism. Horticulturae 7, 151. doi: 10.3390/horticulturae7060151

[B59] Lin C.-P. Ho Y.-C. (2021). Beneficial microbes and basal fertilization in antagonism of banana fusarium wilt. Agronomy 11, 2043. doi: 10.3390/agronomy11102043

[B60] Lin H. Peddada S. D. (2020). Analysis of compositions of microbiomes with bias correction. Nat. Commun. 11, 3514. doi: 10.1038/s41467-020-17041-7 32665548 PMC7360769

[B61] Liu H. Li J. Carvalhais L. C. Percy C. D. Prakash Verma J. Schenk P. M.. (2021). Evidence for the plant recruitment of beneficial microbes to suppress soil-borne pathogens. New Phytol. 229, 2873–2885. doi: 10.1111/nph.17057 33131088

[B62] Lopes M. J. dos S. Dias-Filho M. B. Gurgel E. S. C. (2021). Successful plant growth-promoting microbes: inoculation methods and abiotic factors. Front. Sustain. Food Syst. 5. doi: 10.3389/fsufs.2021.606454

[B63] Lori M. Symnaczik S. Mäder P. De Deyn G. Gattinger A. (2017). Organic farming enhances soil microbial abundance and activity—A meta-analysis and meta-regression. PloS One 12, e0180442. doi: 10.1371/journal.pone.0180442 28700609 PMC5507504

[B64] Malgioglio G. Rizzo G. F. Nigro S. Lefebvre du Prey V. Herforth-Rahmé J. Catara V.. (2022). Plant-microbe interaction in sustainable agriculture: the factors that may influence the efficacy of PGPM application. Sustainability 14, 2253. doi: 10.3390/su14042253

[B65] Mangiafico S. S. (2023). rcompanion: Functions to Support Extension Education Program Evaluation. Available at: https://CRAN.R-project.org/package=rcompanion/.

[B66] Martin M. (2011). Cutadapt removes adapter sequences from high-throughput sequencing reads. EMBnet J. 17, 10. doi: 10.14806/ej.17.1.200

[B67] Martinez A. P. (2022). pairwiseAdonis: Pairwise Multilevel Comparison Using Adonis. Available at: https://github.com/pmartinezarbizu/pairwiseAdonis.

[B68] Mawarda P. C. Lakke S. L. Dirk van Elsas J. Salles J. F. (2022). Temporal dynamics of the soil bacterial community following Bacillus invasion. iScience 25, 104185. doi: 10.1016/j.isci.2022.104185 35479409 PMC9035691

[B69] Mazzola M. Freilich S. (2017). Prospects for biological soilborne disease control: application of indigenous versus synthetic microbiomes. Phytopathology® 107, 256–263. doi: 10.1094/PHYTO-09-16-0330-RVW 27898265

[B70] McMurdie P. J. Holmes S. (2013). phyloseq: an R package for reproducible interactive analysis and graphics of microbiome census data. PloS One 8, e61217. doi: 10.1371/journal.pone.0061217 23630581 PMC3632530

[B71] Mendes R. Garbeva P. Raaijmakers J. M. (2013). The rhizosphere microbiome: significance of plant beneficial, plant pathogenic, and human pathogenic microorganisms. FEMS Microbiol. Rev. 37, 634–663. doi: 10.1111/1574-6976.12028 23790204

[B72] Moradtalab N. Ahmed A. Geistlinger J. Walker F. Höglinger B. Ludewig U.. (2020). Synergisms of microbial consortia, N forms, and micronutrients alleviate oxidative damage and stimulate hormonal cold stress adaptations in maize. Front. Plant Sci. 11. doi: 10.3389/fpls.2020.00396 PMC7193188 32391028

[B73] Morais M. C. Mucha Â. Ferreira H. Gonçalves B. Bacelar E. Marques G. (2019). Comparative study of plant growth-promoting bacteria on the physiology, growth and fruit quality of strawberry. J. Sci. Food Agric. 99, 5341–5349. doi: 10.1002/jsfa.9773 31058322

[B74] Mpanga I. Dapaah H. Geistlinger J. Ludewig U. Neumann G. (2018). Soil type-dependent interactions of P-solubilizing microorganisms with organic and inorganic fertilizers mediate plant growth promotion in tomato. Agronomy 8, 213. doi: 10.3390/agronomy8100213

[B75] Mpanga I. Nkebiwe P. Kuhlmann M. Cozzolino V. Piccolo A. Geistlinger J.. (2019). The form of N supply determines plant growth promotion by P-solubilizing microorganisms in maize. Microorganisms 7, 38. doi: 10.3390/microorganisms7020038 30699936 PMC6406690

[B76] Müller M. Wickham H. (2022). tibble: Simple Data Frames. Available at: https://CRAN.R-project.org/package=tibble.

[B77] Mumtaz M. Z. Ahmad M. Jamil M. Hussain T. (2017). Zinc solubilizing *Bacillus* spp. potential candidates for biofortification in maize. Microbiol. Res. 202, 51–60. doi: 10.1016/j.micres.2017.06.001 28647123

[B78] Nautiyal C. S. (1999). An efficient microbiological growth medium for screening phosphate solubilizing microorganisms. FEMS Microbiol. Lett. 170, 265–270. doi: 10.1111/j.1574-6968.1999.tb13383.x 9919677

[B79] Oksanen J. Simpson G. L. Blanchet F. G. Kindt R. Legendre P. Minchin P. R.. (2022). vegan: Community Ecology Package. Available at: https://CRAN.R-project.org/package=vegan.

[B80] Paradis E. Schliep K. (2019). ape 5.0: an environment for modern phylogenetics and evolutionary analyses in R. Bioinformatics 35, 526–528. doi: 10.1093/bioinformatics/bty633 30016406

[B81] Parnell J. J. Berka R. Young H. A. Sturino J. M. Kang Y. Barnhart D. M.. (2016). From the lab to the farm: an industrial perspective of plant beneficial microorganisms. Front. Plant Sci. 7. doi: 10.3389/fpls.2016.01110 PMC4973397 27540383

[B82] Pascale A. Proietti S. Pantelides I. S. Stringlis I. A. (2020). Modulation of the root microbiome by plant molecules: the basis for targeted disease suppression and plant growth promotion. Front. Plant Sci. 10. doi: 10.3389/fpls.2019.01741 PMC6992662 32038698

[B83] Quast C. Pruesse E. Yilmaz P. Gerken J. Schweer T. Yarza P.. (2012). The SILVA ribosomal RNA gene database project: improved data processing and web-based tools. Nucleic Acids Res. 41, D590–D596. doi: 10.1093/nar/gks1219 23193283 PMC3531112

[B84] Rai S. Kashyap P. L. Kumar S. Srivastava A. K. Ramteke P. W. (2016). Identification, characterization and phylogenetic analysis of antifungal *Trichoderma* from tomato rhizosphere. SpringerPlus 5, 1939. doi: 10.1186/s40064-016-3657-4 27917337 PMC5102998

[B85] Raymond N. S. Gómez-Muñoz B. van der Bom F. J. T. Nybroe O. Jensen L. S. Müller-Stöver D. S.. (2021). Phosphate-solubilising microorganisms for improved crop productivity: a critical assessment. New Phytol. 229, 1268–1277. doi: 10.1111/nph.16924 32929739

[B86] R Core Team (2022). R: A Language and Environment for Statistical Computing. Available at: https://www.R-project.org/.

[B87] Sánchez-Cañizares C. Jorrín B. Poole P. S. Tkacz A. (2017). Understanding the holobiont: the interdependence of plants and their microbiome. Curr. Opin. Microbiol. 38, 188–196. doi: 10.1016/j.mib.2017.07.001 28732267

[B88] Sanket A. S. Ghosh S. Sahoo R. Nayak S. Das A. P. (2017). Molecular identification of acidophilic manganese (Mn)-solubilizing bacteria from mining effluents and their application in mineral beneficiation. Geomicrobiol. J. 34, 71–80. doi: 10.1080/01490451.2016.1141340

[B89] Santoyo G. Guzmán-Guzmán P. Parra-Cota F. I. de los Santos-Villalobos S. del Orozco-Mosqueda, Ma. C. Glick B. R. (2021). Plant growth stimulation by microbial consortia. Agronomy 11, 219. doi: 10.3390/agronomy11020219

[B90] Schierstaedt J. Jechalke S. Nesme J. Neuhaus K. Sørensen S. J. Grosch R.. (2020). *Salmonella* persistence in soil depends on reciprocal interactions with indigenous microorganisms. Environ. Microbiol. 22, 2639–2652. doi: 10.1111/1462-2920.14972 32128943

[B91] Schrama M. de Haan J. J. Kroonen M. Verstegen H. van der Putten W. H. (2018). Crop yield gap and stability in organic and conventional farming systems. Agric. Ecosyst. Environ. 256, 123–130. doi: 10.1016/j.agee.2017.12.023

[B92] Schreiter S. Babin D. Smalla K. Grosch R. (2018). Rhizosphere Competence and Biocontrol Effect of *Pseudomonas* sp. RU47 Independent from Plant Species and Soil Type at the Field Scale. Front. Microbiol. 9. doi: 10.3389/fmicb.2018.00097 PMC5799239 29449832

[B93] Schreiter S. Sandmann M. Smalla K. Grosch R. (2014). Soil type dependent rhizosphere competence and biocontrol of two bacterial inoculant strains and their effects on the rhizosphere microbial community of field-grown lettuce. PloS One 9, e103726. doi: 10.1371/journal.pone.0103726 25099168 PMC4123886

[B94] Schütz L. Gattinger A. Meier M. Müller A. Boller T. Mäder P.. (2018). Improving crop yield and nutrient use efficiency via biofertilization—A global meta-analysis. Front. Plant Sci. 8. doi: 10.3389/fpls.2017.02204 PMC5770357 29375594

[B95] Schwyn B. Neilands J. B. (1987). Universal chemical assay for the detection and determination of siderophores. Anal. Biochem. 160, 47–56. doi: 10.1016/0003-2697(87)90612-9 2952030

[B96] Sella S. R. B. R. Vandenberghe L. P. S. Soccol C. R. (2014). Life cycle and spore resistance of spore-forming *Bacillus atrophaeus* . Microbiol. Res. 169, 931–939. doi: 10.1016/j.micres.2014.05.001 24880805

[B97] Seufert V. Ramankutty N. Foley J. A. (2012). Comparing the yields of organic and conventional agriculture. Nature 485, 229–232. doi: 10.1038/nature11069 22535250

[B98] Sharma S. Compant S. Ballhausen M.-B. Ruppel S. Franken P. (2020). The interaction between *Rhizoglomus irregulare* and hyphae attached phosphate solubilizing bacteria increases plant biomass of *Solanum lycopersicum* . Microbiol. Res. 240, 126556. doi: 10.1016/j.micres.2020.126556 32683279

[B99] Sommermann L. Babin D. Behr J. H. Chowdhury S. P. Sandmann M. Windisch S.. (2022). Long-term fertilization strategy impacts *Rhizoctonia solani*–microbe interactions in soil and rhizosphere and defense responses in lettuce. Microorganisms 10, 1717. doi: 10.3390/microorganisms10091717 36144319 PMC9501836

[B100] Sun X. Xu Z. Xie J. Hesselberg-Thomsen V. Tan T. Zheng D.. (2022). *Bacillus velezensis* stimulates resident rhizosphere *Pseudomonas stutzeri* for plant health through metabolic interactions. ISME J. 16, 774–787. doi: 10.1038/s41396-021-01125-3 34593997 PMC8483172

[B101] Sundberg C. Al-Soud W. A. Larsson M. Alm E. Yekta S. S. Svensson B. H.. (2013). 454 pyrosequencing analyses of bacterial and archaeal richness in 21 full-scale biogas digesters. FEMS Microbiol. Ecol. 85, 612–626. doi: 10.1111/1574-6941.12148 23678985

[B102] Thonar C. Lekfeldt J. D. S. Cozzolino V. Kundel D. Kulhánek M. Mosimann C.. (2017). Potential of three microbial bio-effectors to promote maize growth and nutrient acquisition from alternative phosphorous fertilizers in contrasting soils. Chem. Biol. Technol. Agric. 4, 7. doi: 10.1186/s40538-017-0088-6

[B103] Topçuoğlu B. D. Lesniak N. A. Ruffin M. T. Wiens J. Schloss P. D. (2020). A framework for effective application of machine learning to microbiome-based classification problems. mBio 11, e00434–e00420. doi: 10.1128/mBio.00434-20 32518182 PMC7373189

[B104] Tosi M. Mitter E. K. Gaiero J. Dunfield K. (2020). It takes three to tango: the importance of microbes, host plant, and soil management to elucidate manipulation strategies for the plant microbiome. Can. J. Microbiol. 66, 413–433. doi: 10.1139/cjm-2020-0085 32396748

[B105] Usero F. M. Armas C. Morillo J. A. Gallardo M. Thompson R. B. Pugnaire F. I. (2021). Effects of soil microbial communities associated to different soil fertilization practices on tomato growth in intensive greenhouse agriculture. Appl. Soil Ecol. 162, 103896. doi: 10.1016/j.apsoil.2021.103896

[B106] van Elsas J. D. Chiurazzi M. Mallon C. A. Elhottovā D. Krištůfek V. Salles J. F. (2012). Microbial diversity determines the invasion of soil by a bacterial pathogen. Proc. Natl. Acad. Sci. U.S.A. 109, 1159–1164. doi: 10.1073/pnas.1109326109 22232669 PMC3268289

[B107] Verband Deutscher Landwirtschaftlicher Untersuchungs- und Forschungsanstalten (2011). Umweltanalytik. 4th ed. Ed. Janßen E. (Darmstadt: VDLUFA-Verlag).

[B108] Vlot A. C. Sales J. H. Lenk M. Bauer K. Brambilla A. Sommer A.. (2021). Systemic propagation of immunity in plants. New Phytol. 229, 1234–1250. doi: 10.1111/nph.16953 32978988

[B109] Watteau F. Villemin G. (2018). Soil microstructures examined through transmission electron microscopy reveal soil-microorganisms interactions. Front. Environ. Sci. 6. doi: 10.3389/fenvs.2018.00106

[B110] Wei Z. Friman V.-P. Pommier T. Geisen S. Jousset A. Shen Q. (2020). Rhizosphere immunity: targeting the underground for sustainable plant health management. Front. Agr. Sci. Eng. 7, 317. doi: 10.15302/J-FASE-2020346

[B111] Weinert N. Meincke R. Gottwald C. Heuer H. Schloter M. Berg G.. (2010). Bacterial diversity on the surface of potato tubers in soil and the influence of the plant genotype. FEMS Microbiol. Ecol. 74, 114–123. doi: 10.1111/j.1574-6941.2010.00936.x 20698886

[B112] Wickham H. (2007). Reshaping data with the reshape package. J. Stat. Software 21, 1–20. doi: 10.18637/jss.v021.i12

[B113] Wickham H. (2016). ggplot2: Elegant Graphics for Data Analysis. 2nd ed (Cham: Springer International Publishing: Imprint: Springer). doi: 10.1007/978-3-319-24277-4

[B114] Wickham H. Averick M. Bryan J. Chang W. McGowan L. François R.. (2019). Welcome to the tidyverse. JOSS 4, 1686. doi: 10.21105/joss.01686

[B115] Wickham H. François R. Henry L. Müller K. Vaughan D. (2023). dplyr: A Grammar of Data Manipulation. Available at: https://dplyr.tidyverse.org https://github.com/tidyverse/dplyr.

[B116] Williams J. Clarkson J. M. Mills P. R. Cooper R. M. (2003). A Selective Medium for Quantitative Reisolation of *Trichoderma harzianum* from *Agaricus bisporus* Compost. Appl. Environ. Microbiol. 69, 4190–4191. doi: 10.1128/AEM.69.7.4190-4191.2003 12839798 PMC165142

[B117] Windisch S. Bott S. Ohler M.-A. Mock H.-P. Lippmann R. Grosch R.. (2017). *Rhizoctonia solani* and bacterial inoculants stimulate root exudation of antifungal compounds in lettuce in a soil-type specific manner. Agronomy 7, 44. doi: 10.3390/agronomy7020044

[B118] Windisch S. Sommermann L. Babin D. Chowdhury S. P. Grosch R. Moradtalab N.. (2021). Impact of long-term organic and mineral fertilization on rhizosphere metabolites, root–microbial interactions and plant health of lettuce. Front. Microbiol. 11. doi: 10.3389/fmicb.2020.597745 PMC7838544 33519736

[B119] Zubair M. Hanif A. Farzand A. Sheikh T. M. M. Khan A. R. Suleman M.. (2019). Genetic screening and expression analysis of psychrophilic bacillus spp. Reveal their potential to alleviate cold stress and modulate phytohormones in wheat. Microorganisms 7, 337. doi: 10.3390/microorganisms7090337 31510075 PMC6780275

