# Supplementary material for: Beneficial microbial consortium improves winter rye performance by modulating bacterial communities in the rhizosphere and enhancing plant nutrient acquisition
Source: Front Plant Sci. 2023 Aug 28;14:1232288. doi: 10.3389/fpls.2023.1232288 (PMC10498285; doi:10.3389/fpls.2023.1232288)
Supplement: Supplementary file 1 [file DataSheet_1.docx]

**Supplementary figures**


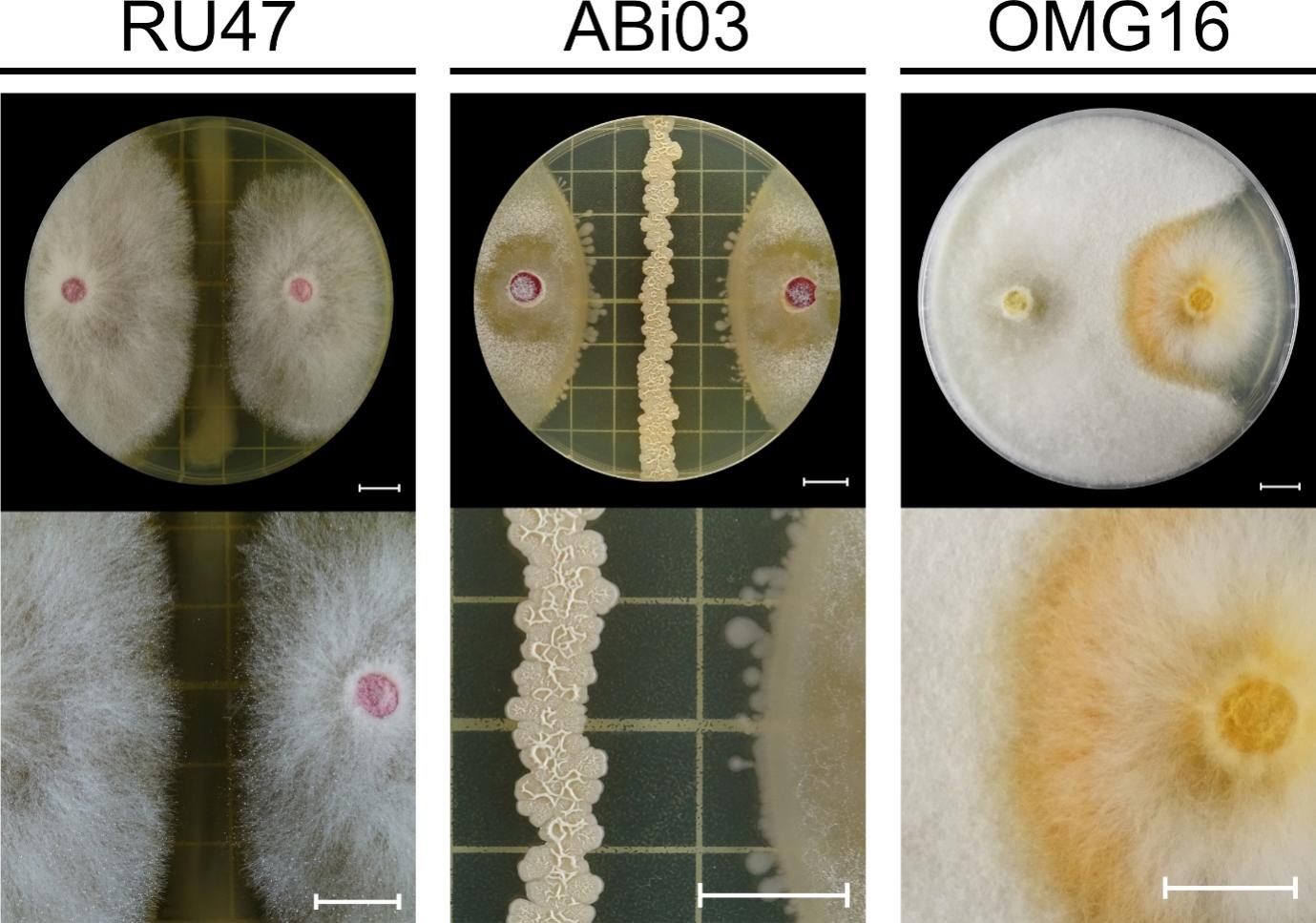


**Supplementary figure 1 |** *In vitro* dual culture assay between *Fusarium graminearum* and each beneficial microorganism. The two bacterial strains RU47 and ABi03 were placed in the center of the plate in a straight line. On both sides of the line a disc with freshly grown *Fusarium graminearum* was placed. For the fungal strain, a disc with OMG16 (left) and *Fusarium graminearum* (right) were placed on opposite sides of the plate. The plates were incubated for 10 days at 28°C in the dark. Lower images show border region in detail. Scale bar = 1cm. Figure was created by Hans Tietze.

**
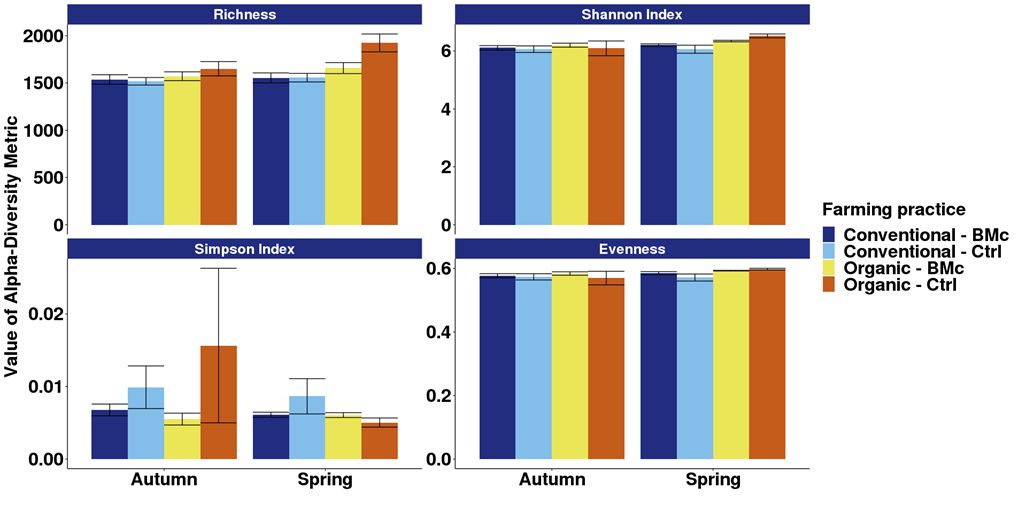
**

**Supplementary figure 2 |** Metrics of α-diversity among the different treatments (farming practice and inoculation of beneficial microorganisms, n=4). BMc: beneficial microorganisms consortium, Ctrl: Control. The error-bars represent standard deviation.


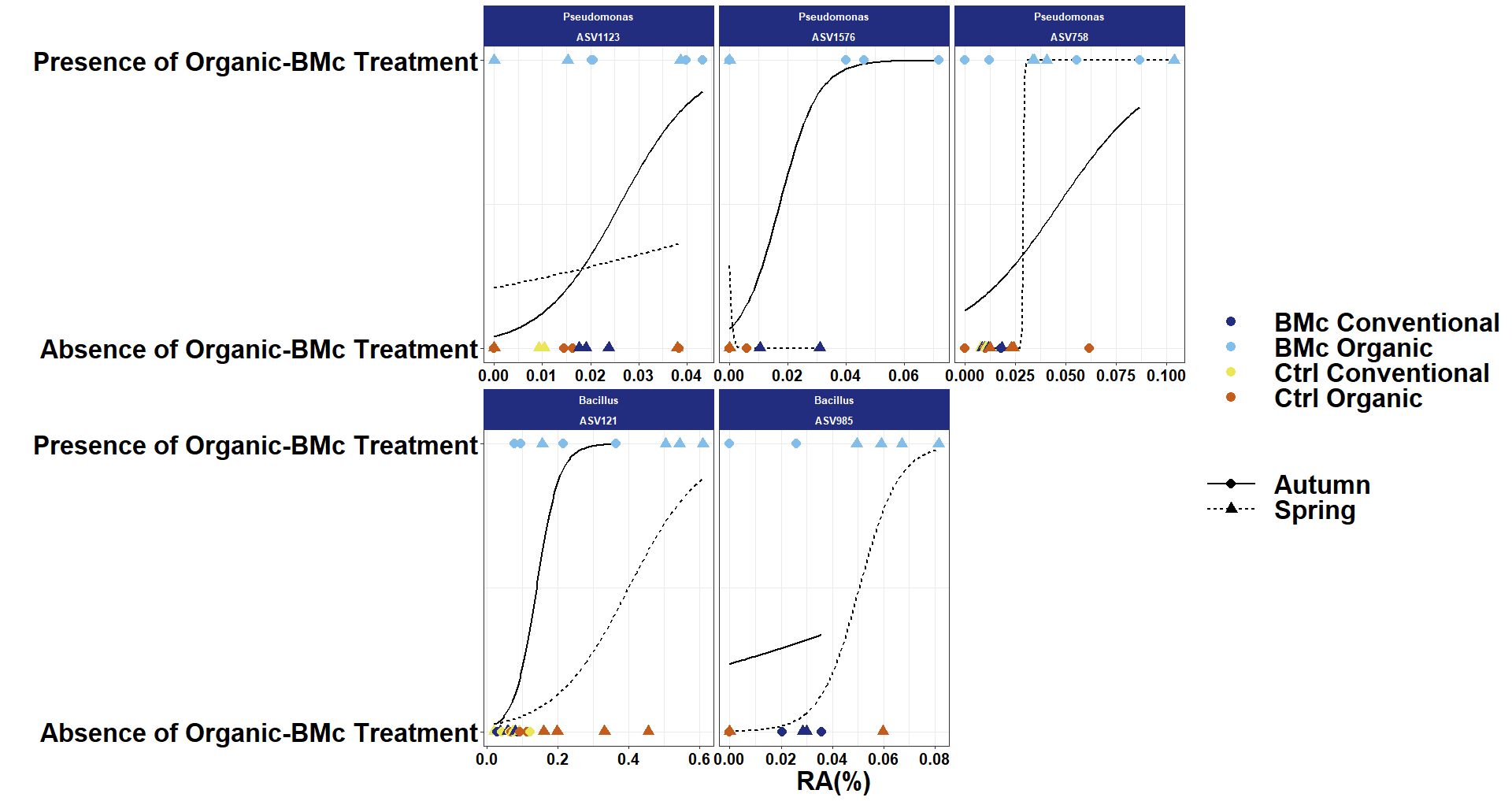


**Supplementary figure 3 |** Logistic regression models for the Organic-BMc treatment with the relative abundance of the *Bacillus* (A) and *Pseudomonas* (B) responders. The logistic regression model was fit for autumn and spring samplings separately.
