# Supplementary material for: Beneficial microbial consortium improves winter rye performance by modulating bacterial communities in the rhizosphere and enhancing plant nutrient acquisition
Source: Front Plant Sci. 2023 Aug 28;14:1232288. doi: 10.3389/fpls.2023.1232288 (PMC10498285; doi:10.3389/fpls.2023.1232288)
Supplement: Supplementary file 2 [file Table_1.docx]

**Supplementary table 1 |** Influence of the sampling time and long-time farming practice on the rhizosphere competence of the beneficial microorganisms consortium (BMc). A) Abundance of BMc in the rhizosphere of winter rye cultivated with different long-term farming practices (conventional, organic) in autumn and spring of the same growing season. Values show means ± standard deviation of four replicates. Means with different letters indicate significant differences (p ≤ 0.05). Different letter types indicate a separate statistical analysis. B) Test of main effect and interactions between different sampling time points (ST) and different long-term farming practices (MGMT) via two-way ANOVA. *P*-values below the significance threshold of *p* < 0.05 are highlighted in bold.

| A) | | **RU47*** | | |  | **ABi03** | | |  | **OMG16** | | |
| --- | --- | --- | --- | --- | --- | --- | --- | --- | --- | --- | --- | --- |
|  |  | [LOG CFU g^-1^ DM_root_] | | |  | [LOG CFU g^-1^ DM_root_] | | |  | [LOG CFU g^-1^ DM_soil_] | | |
|  |  |  |  |  |  |  |  |  |  |  |  |  |
| **Conventional** | |  |  |  |  |  |  |  |  |  |  |  |
|  | Autumn | 6.70 | ±0.81 | ab |  | 6.91 | ±0.75 | A |  | 5.36 | ±0.22 | *ab* |
|  | Spring | 5.30 | ±0.63 | b |  | 6.68 | ±0.32 | A |  | 5.31 | ±0.28 | *ab* |
|  |  |  |  |  |  |  |  |  |  |  |  |  |
| **Organic** | |  |  |  |  |  |  |  |  |  |  |  |
|  | Autumn | 6.98 | ±1.11 | a |  | 7.41 | ±0.42 | A |  | 5.65 | ±0.29 | *a* |
|  | Spring | 5.74 | ±0.23 | ab |  | 7.02 | ±0.10 | A |  | 5.10 | ±0.34 | *b* |
|  |  |  |  |  |  |  |  |  |  |  |  |  |

| B) | **RU47*** | |  | **ABi03** | |  | **OMG16** | |
| --- | --- | --- | --- | --- | --- | --- | --- | --- |
|  |  | |  |  | |  |  | |
|  | **F-value** | ***p*-value** |  | **F-value** | ***p*-value** |  | **F-value** | ***p*-value** |
| ST | 9.61 | **0.015** |  | 1.82 | 0.210 |  | 6.64 | **0.030** |
| MGMT | 0.73 | 0.417 |  | 3.25 | 0.105 |  | 0.14 | 0.717 |
| ST x MGMT | 0.19 | 0.675 |  | 0.13 | 0.729 |  | 4.72 | 0.058 |

* Analysis with transformed data
