# Supplementary material for: Beneficial microbial consortium improves winter rye performance by modulating bacterial communities in the rhizosphere and enhancing plant nutrient acquisition
Source: Front Plant Sci. 2023 Aug 28;14:1232288. doi: 10.3389/fpls.2023.1232288 (PMC10498285; doi:10.3389/fpls.2023.1232288)
Supplement: Supplementary file 3 [file Table_2.docx]

**Supplementary table 2 |** Impact of the beneficial microorganism consortium (BMc) on the biomass of winter rye plants grown at different long-time farming practices. A) Shoot dry mass (SDM) of winter rye plants sampled in autumn and spring of the same growing season. Values show means ± standard deviation of four replicates. Means not sharing any letter are significantly different by the Sidak-test (*p* ≤ 0.05). B) Test of main effect and interactions between different long-term farming practices (MGMT; conventional *vs.* organic) and the use of a consortium of three beneficial microorganisms (control *vs.* BMc) on the shoot dry mass of winter rye in autumn and spring of the same growing season via two-way ANOVA. *P*-values below the significance threshold of *p* < 0.05 are highlighted in bold.

| A) |  | **SDM** [g plant^-1^] | | | | | | |
| --- | --- | --- | --- | --- | --- | --- | --- | --- |
|  |  | **Autumn** | | |  | **Spring** | | |
| Conventional | Ctrl | 0.08 | ±0.008 | b |  | 0.29 | ±0.055 | B |
|  | BMc | 0.07 | ±0.010 | b |  | 0.31 | ±0.048 | B |
|  |  |  |  |  |  |  |  |  |
| Organic | Ctrl | 0.08 | ±0.003 | b |  | 0.26 | ±0.023 | B |
|  | BMc | 0.10 | ±0.003 | a |  | 0.48 | ±0.032 | A |

| B) | **Autumn** | |  | **Spring** | | |  |
| --- | --- | --- | --- | --- | --- | --- | --- |
|  |  |  |  |  | |  |  |
| **Factor** | **F-value** | ***p*-value** |  | **F-value** | ***p*-value** | | |
| MGMT | 12.80 | **0.005** |  | 10.10 | **0.011** | | |
| BMc | 2.15 | 0.177 |  | 35.19 | **<0.001** | | |
| MGMT x BMc | 11.45 | **0.008** |  | 23.02 | **<0.001** | | |
