# Supplementary material for: Beneficial microbial consortium improves winter rye performance by modulating bacterial communities in the rhizosphere and enhancing plant nutrient acquisition
Source: Front Plant Sci. 2023 Aug 28;14:1232288. doi: 10.3389/fpls.2023.1232288 (PMC10498285; doi:10.3389/fpls.2023.1232288)
Supplement: Supplementary file 4 [file Table_3.docx]

**Supplementary table 3 |** P-values of the two-way ANOVA showing the main and interactions effects between different long-time farming practices (MGMT; conventional *vs.* organic) and use of beneficial microorganisms consortium (BMc; control *vs.* BMc) on the nutrient status of winter rye (*cv.* Conduct) at two separate time points (autumn and spring). Statistical significance was indicated by marking values with a significance threshold of *p* < 0.05 in bold.

|  | **Autumn** | | |  | **Spring** | | |
| --- | --- | --- | --- | --- | --- | --- | --- |
|  |  | | |  |  | | |
|  | **MGMT** | **BMc** | **MGMT x BMc** |  | **MGMT** | **BMc** | **MGMT x BMc** |
| C_total_ | **0.043** | 0.255 | 0.597 |  | 0.084 | **0.029** | 0.907 |
| N_total_ | **0.003** | **0.021** | 0.409 |  | **< 0.001** | **0.011** | 0.668 |
| P | 0.663 | 0.504 | 0.698 |  | **0.002** | **0.003** | 0.597 |
| K | 0.058 | 1.000 | 0.726 |  | **0.008** | **0.011** | 0.584 |
| Mg | **0.049** | 0.292 | 0.612 |  | **< 0.001** | **0.008** | 0.476 |
| Ca | 0.348* | 0.435* | 0.578* |  | 0.167 | **0.014** | 0.337 |
| S | 0.061 | 0.226 | 0.571 |  | **< 0.001** | **0.009** | 0.274 |
| Cu | 0.957 | 0.689 | 0.816 |  | 0.792 | **0.050** | 0.988 |
| Mn | **0.016** | 0.148 | 0.593 |  | **0.006** | **0.005** | 0.608 |
| Zn | 0.208 | 0.109 | 0.536 |  | **< 0.001*** | 0.873* | 0.875* |

* Analysis with transformed data

#
