# Supplementary material for: Beneficial microbial consortium improves winter rye performance by modulating bacterial communities in the rhizosphere and enhancing plant nutrient acquisition
Source: Front Plant Sci. 2023 Aug 28;14:1232288. doi: 10.3389/fpls.2023.1232288 (PMC10498285; doi:10.3389/fpls.2023.1232288)
Supplement: Supplementary file 5 [file Table_4.docx]

**Supplementary table 4 |** Effect of BMc inoculation on the chemical properties of soils with different long-time farming practices. A) Nutrient composition of soils with different long-time farming practice (conventional and organic) 22 WPI. In spring, root-associated soil of control (Ctrl) and inoculated plants (BMc) were sampled in a depth of 0-30 cm. Abbreviations: total carbon (TC), total nitrogen (TN), dry matter (DM), organic matter (OM). Values represent means ± standard deviation of four replicates. Means not sharing any letter are significantly different by the Sidak-test (*p* ≤ 0.05). B) Test of the main effect and interactions between different long-term farming practices (MGMT; conventional *vs.* organic) and the use of beneficial microorganisms (BMc; control *vs.* BMc) on the chemical properties of root-associated soil via two-way ANOVA. *P*-values below the significance threshold of *p* < 0.05 are highlighted in bold.

| A) | | **Conventional** | | | | | | | | | |  | **Organic** | | | | | | |  |
| --- | --- | --- | --- | --- | --- | --- | --- | --- | --- | --- | --- | --- | --- | --- | --- | --- | --- | --- | --- | --- |
|  | |  | | | | | | | | | |  |  | | | | | | |  |
|  | | **Ctrl** | | | | |  | **BMc** | | | |  | **Ctrl** | | |  | **BMc** | | | |
|  | pH | 5.5 | | | ±0 | c |  | 5.7 | ±0.3 | bc | |  | 6.1 | ±0.2 | ab |  | 6.2 | ±0.3 | a | |
|  | C/N | 12.5 | | | ±0.58 | a |  | 11.5 | ±0.58 | | a |  | 12.5 | ±0.58 | a |  | 12.5 | ±1.29 | a | |
|  |  |  | | |  |  |  |  |  | |  |  |  |  |  |  |  |  |  | |
| **[%]** | | | | |  |  |  |  |  | |  |  |  |  |  |  |  |  |  | |
|  | TC | 0.80 | | | ±0.06 | a |  | 0.78 | ±0.04 | | a |  | 0.78 | ±0.05 | a |  | 0.82 | ±0.05 | a | |
|  | TN^#^ | 0.07 | | | ±0.01 | - |  | 0.07 | ±0 | | - |  | 0.06 | ±0.01 | - |  | 0.07 | ±0.01 | - | |
|  | DM | 90.3 | | | ±0.1 | ab |  | 90.3 | ±0.3 | | b |  | 90.6 | ±0.1 | ab |  | 90.9 | ±0.4 | a | |
|  | OM | | | 1.35 | ±0.06 | a |  | 1.35 | ±0.06 | | a |  | 1.35 | ±0.13 | a |  | 1.35 | ±0.06 | a | |
|  |  |  | | |  |  |  |  |  | |  |  |  |  |  |  |  |  |  | |
| **[mg 100 g^-1^ soil]** | | | | |  |  |  |  |  | |  |  |  |  |  |  |  |  |  | |
|  | NO_3_-N^#^ | | 0.65 | | ±0.17 | - |  | 1.00 | ±0.07 | | - |  | 0.08 | ±0.01 | - |  | 0.07 | ±0.01 | - | |
|  | NH_4_-N^#^ | | 1.70 | | ±0.62 | - |  | 1.75 | ±0.34 | | - |  | 0.12 | ±0.03 | - |  | 0.13 | ±0.04 | - | |
|  | K | | 20.6 | | ±2.2 | a |  | 20.9 | ±2.8 | | a |  | 8.5 | ±1.2 | b |  | 8.9 | ±2.1 | b | |
|  | Mg^*^ | | 29.7 | | ±14.3 | a |  | 50.6 | ±4.4 | | a |  | 26.8 | ±15.9 | a |  | 35.5 | ±23.0 | a | |
|  | P | | 6.53 | | ±0.50 | a |  | 6.78 | ±0.52 | | a |  | 7.15 | ±0.58 | a |  | 7.08 | ±0.79 | a | |
|  | SO_4_-S^#^ | | 1.80 | | ±0.94 | - |  | 2.29 | ±0.65 | | - |  | 0.09 | ±0.02 | - |  | 0.07 | ±0.01 | - | |
|  | Ca | | 22.3 | | ±3.29 | b |  | 23.5 | ±1.79 | | b |  | 39.1 | ±4.97 | a |  | 37.3 | ±4.25 | a | |
|  | Fe | | 7.43 | | ±0.62 | a |  | 7.38 | ±0.35 | | a |  | 6.88 | ±0.46 | a |  | 6.68 | ±0.22 | a | |
|  |  | |  | |  |  |  |  |  | |  |  |  |  |  |  |  |  |  | |
| **[mg kg^-1^ soil]** | | | | |  |  |  |  |  | |  |  |  |  |  |  |  |  |  | |
|  | Cu^#^ | 1.18 | | | ±0.10 | - |  | 1.20 | - | | - |  | 1.40 | ±0.08 | - |  | 1.40 | - | - | |
|  | Mn | 46.0 | | | ±5.9 | a |  | 45.5 | ±1.3 | | a |  | 34.3 | ±3.6 | b |  | 33.5 | ±1.3 | b | |
|  | Na | 4.75 | | | ±0.96 | a |  | 5.50 | ±0.58 | | a |  | 2.75 | ±0.50 | b |  | 2.50 | ±0.58 | b | |
|  | B^#^ | < 0.10 | | | - | - |  | < 0.10 | - | | - |  | 0.15 | ±0.01 | - |  | 0.15 | ±0.02 | - | |
|  | Zn | 2.25 | | | ±0.25 | a |  | 2.30 | ±0.18 | | a |  | 2.48 | ±0.36 | a |  | 2.33 | ±0.19 | a | |

* Analysis with transformed data

# Statistical analysis not feasible

| B) | **MGMT** | |  | **BMc** | |  | **MGMT x BMc** | |
| --- | --- | --- | --- | --- | --- | --- | --- | --- |
|  |  |  |  |  |  |  |  |  |
|  | **F-value** | ***p*-value** |  | **F-value** | ***p*-value** |  | **F-value** | ***p*-value** |
| pH | 31.45 | **< 0.001** |  | 3.16 | 0.109 |  | 0.92 | 0.363 |
| C/N | 1.29 | 0.286 |  | 1.29 | 0.286 |  | 1.29 | 0.286 |
| TC | 0.16 | 0.697 |  | 0.29 | 0.603 |  | 1.34 | 0.278 |
| DM | 10.84 | **0.009** |  | 0.72 | 0.419 |  | 1.50 | 0.252 |
| OM | 0.16 | 0.700 |  | 0.16 | 0.700 |  | 0.16 | 0.700 |
| TN^#^ | - | - |  | - | - |  | - | - |
| NO_3_-N^#^ | - | - |  | - | - |  | - | - |
| NH_4_-N^#^ | - | - |  | - | - |  | - | - |
| K | 128.7 | **< 0.001** |  | 0.12 | 0.740 |  | 0.00 | 0.973 |
| Mg^*^ | 1.12 | 0.317 |  | 3.28 | 0.104 |  | 0.53 | 0.484 |
| P | 4.16 | 0.072 |  | 0.15 | 0.709 |  | 0.51 | 0.492 |
| SO_4_-S^#^ | - | - |  | - | - |  | - | - |
| Ca | 78.62 | **< 0.001** |  | 0.03 | 0.876 |  | 0.79 | 0.398 |
| Fe | 11.01 | **0.009** |  | 0.44 | 0.524 |  | 0.16 | 0.700 |
| Cu^#^ | - | - |  | - | - |  | - | - |
| Mn | 75.14 | **< 0.001** |  | 0.21 | 0.659 |  | 0.01 | 0.929 |
| Na | 52.94 | **< 0.001** |  | 0.53 | 0.485 |  | 2.12 | 0.180 |
| B^#^ | - | - |  | - | - |  | - | - |
| Zn | 1.19 | 0.304 |  | 0.19 | 0.673 |  | 0.76 | 0.405 |

* Analysis with transformed data

# Statistical analysis not feasible
