# Supplementary material for: Beneficial microbial consortium improves winter rye performance by modulating bacterial communities in the rhizosphere and enhancing plant nutrient acquisition
Source: Front Plant Sci. 2023 Aug 28;14:1232288. doi: 10.3389/fpls.2023.1232288 (PMC10498285; doi:10.3389/fpls.2023.1232288)
Supplement: Supplementary file 6 [file Table_5.docx]

**Supplementary table 5 |** Two-way ANOVA (in case Shapiro test *p-*value > 0.05) and two-way Aligned-Ranked ANOVA (in case Shapiro test *p*-value ≤ 0.05) for the bacterial alpha-diversity in the rhizosphere of rye in autumn and spring sampling. *P*-values below the significance threshold of *p* < 0.05 are highlighted in bold.

| **Sampling** | **Metric** | ***p*-value MGMT** | ***p*-value BMc** | ***p*-value**  **MGMT X BMc** | **Shapiro test** |
| --- | --- | --- | --- | --- | --- |
| **Autumn** | Evenness | 0.910 | 0.491 | 0.668 | 0.059 |
|  | Richness | 0.153 | 0.593 | 0.383 | 0.710 |
|  | Shannon Index | 0.696 | 0.610 | 0.831 | 0.163 |
|  | Simpson Index | 1.000 | 0.509 | 0.752 | 0.05 |
|  |  |  |  |  |  |
| **Spring** | Evenness | 0.018 | 0.484 | 0.157 | 0.054 |
|  | Richness | **0.004** | 0.062 | 0.066 | 0.696 |
|  | Shannon Index | **0.003** | 0.828 | 0.069 | 0.403 |
|  | Simpson Index | 0.21 | 1.00 | 0.20 | **<0.05** |
