# Supplementary material for: Beneficial microbial consortium improves winter rye performance by modulating bacterial communities in the rhizosphere and enhancing plant nutrient acquisition
Source: Front Plant Sci. 2023 Aug 28;14:1232288. doi: 10.3389/fpls.2023.1232288 (PMC10498285; doi:10.3389/fpls.2023.1232288)
Supplement: Supplementary file 7 [file Table_6.docx]

**Supplementary table 6.** ASVs that significantly differed between Control (Ctrl) vs BMc inoculation and between Organic and Conventional farming in the autumn or the spring sampling. Differential abundance testing via ANCOM-BC2 and Benjamini-Hochberg correction. FC: Fold Change, RA: Relative Abundance.

| **log10FC** | **ASV** | **Sampling** | **Tested Condition** | **Max RA%** |
| --- | --- | --- | --- | --- |
| -2.84889 | ASV1152_Aureimonas | Spring | BMc/Ctrl | 0.070445 |
| -2.54443 | ASV148_Chitinophaga | Spring | BMc/Ctrl | 0.366722 |
| 2.075248 | ASV191_Paenibacillus | Spring | BMc/Ctrl | 0.462966 |
| 1.813993 | ASV276_Burkholderia-Caballeronia-Paraburkholderia | Spring | BMc/Ctrl | 0.204245 |
| -2.76612 | ASV31_Pedobacter | Spring | BMc/Ctrl | 3.233982 |
| -4.74022 | ASV331_Microbacterium | Spring | BMc/Ctrl | 0.094948 |
| 3.508067 | ASV396_Sphingomonas | Spring | BMc/Ctrl | 0.20415 |
| 5.370905 | ASV453_Pseudarthrobacter | Spring | BMc/Ctrl | 0.142107 |
| 2.632386 | ASV465_Luteolibacter | Spring | BMc/Ctrl | 0.132793 |
| 5.228569 | ASV466_Sphingopyxis | Spring | BMc/Ctrl | 0.149512 |
| 2.577889 | ASV48_Luteibacter | Spring | BMc/Ctrl | 3.087764 |
| 1.740903 | ASV593_Kitasatospora | Spring | BMc/Ctrl | 0.07505 |
| 1.611901 | ASV61_Luteibacter | Spring | BMc/Ctrl | 1.010956 |
| -2.19788 | ASV775_Rhodoferax | Spring | BMc/Ctrl | 0.11211 |
| 2.084655 | ASV1063_Candidatus Alysiosphaera | Autumn | BMc/Ctrl | 0.039355 |
| 2.673951 | ASV167_Chitinophaga | Autumn | BMc/Ctrl | 0.449652 |
| 2.312302 | ASV2402_Chitinophaga | Autumn | BMc/Ctrl | 0.014425 |
| 3.221064 | ASV260_Mycobacterium | Autumn | BMc/Ctrl | 0.179151 |
| 4.622017 | ASV300_Chitinophaga | Autumn | BMc/Ctrl | 0.342092 |
| 2.918993 | ASV312_Taibaiella | Autumn | BMc/Ctrl | 0.135215 |
| 1.812755 | ASV329_Burkholderia-Caballeronia-Paraburkholderia | Autumn | BMc/Ctrl | 0.196212 |
| 1.647009 | ASV37_Mucilaginibacter | Autumn | BMc/Ctrl | 1.140017 |
| 2.888648 | ASV409_Burkholderia-Caballeronia-Paraburkholderia | Autumn | BMc/Ctrl | 0.126241 |
| 2.755003 | ASV437_Mucilaginibacter | Autumn | BMc/Ctrl | 0.11321 |
| 4.874256 | ASV465_Luteolibacter | Autumn | BMc/Ctrl | 0.199512 |
| 5.115028 | ASV466_Sphingopyxis | Autumn | BMc/Ctrl | 0.117291 |
| 1.840386 | ASV48_Luteibacter | Autumn | BMc/Ctrl | 1.191666 |
| 2.425127 | ASV555_Sphingomonas | Autumn | BMc/Ctrl | 0.053735 |
| 2.339302 | ASV571_Luteolibacter | Autumn | BMc/Ctrl | 0.038284 |
| 5.283848 | ASV605_Pedobacter | Autumn | BMc/Ctrl | 0.230695 |
| 2.017726 | ASV61_Luteibacter | Autumn | BMc/Ctrl | 0.858001 |
| 1.297843 | ASV72_Bacillus | Autumn | BMc/Ctrl | 0.413106 |
| 1.679289 | ASV73_Pedobacter | Autumn | BMc/Ctrl | 0.790357 |
| 2.091462 | ASV78_Pedobacter | Autumn | BMc/Ctrl | 0.851866 |
| 4.015861 | ASV892_Nocardioides | Autumn | BMc/Ctrl | 0.052742 |
| 2.932363 | ASV1003_Unclassified_11-24 | Spring | Organic/Integrated | 0.063433 |
| 2.740728 | ASV1029_Larkinella | Spring | Organic/Integrated | 0.065033 |
| 1.917879 | ASV103_Sphingomonas | Spring | Organic/Integrated | 0.428809 |
| -2.53475 | ASV1040_Unclassified_B12-WMSP1 | Spring | Organic/Integrated | 0.041017 |
| 1.26727 | ASV106_Stappia | Spring | Organic/Integrated | 0.364118 |
| 3.878725 | ASV1060_Gaiella | Spring | Organic/Integrated | 0.0601 |
| -2.32866 | ASV1062_Mycobacterium | Spring | Organic/Integrated | 0.042684 |
| 2.806631 | ASV1065_Rhodoplanes | Spring | Organic/Integrated | 0.050488 |
| 2.045089 | ASV1069_Paenibacillus | Spring | Organic/Integrated | 0.053315 |
| 3.2976 | ASV1078_JGI 0001001-H03 | Spring | Organic/Integrated | 0.073225 |
| 4.438601 | ASV1079_Mycobacterium | Spring | Organic/Integrated | 0.102327 |
| -2.20606 | ASV1081_Unclassified_Gaiellales | Spring | Organic/Integrated | 0.049337 |
| 2.959537 | ASV1091_Candidatus Udaeobacter | Spring | Organic/Integrated | 0.071569 |
| 2.56327 | ASV1095_Unclassified_Steroidobacteraceae | Spring | Organic/Integrated | 0.05365 |
| -2.97205 | ASV1100_Pedobacter | Spring | Organic/Integrated | 0.029157 |
| -2.14502 | ASV1104_Unclassified_Chitinophagaceae | Spring | Organic/Integrated | 0.072034 |
| 1.179815 | ASV111_Streptomyces | Spring | Organic/Integrated | 0.39082 |
| 2.802869 | ASV1117_Unclassified_Acidobacteriales | Spring | Organic/Integrated | 0.057769 |
| -2.4837 | ASV114_Polaromonas | Spring | Organic/Integrated | 0.549124 |
| 3.698371 | ASV1143_Devosia | Spring | Organic/Integrated | 0.059464 |
| 3.734303 | ASV1165_Unclassified_Blastocatellaceae | Spring | Organic/Integrated | 0.050478 |
| -3.17552 | ASV1169_Unclassified_Chitinophagaceae | Spring | Organic/Integrated | 0.034242 |
| 3.256988 | ASV1172_Candidatus Ovatusbacter | Spring | Organic/Integrated | 0.114964 |
| 4.201967 | ASV1178_Unclassified_Gemmatimonadaceae | Spring | Organic/Integrated | 0.082502 |
| 1.153019 | ASV118_Paenarthrobacter | Spring | Organic/Integrated | 0.368981 |
| 2.840337 | ASV1180_Lysobacter | Spring | Organic/Integrated | 0.02642 |
| 2.862304 | ASV1186_Rhodococcus | Spring | Organic/Integrated | 0.051559 |
| 3.709497 | ASV1191_Paenibacillus | Spring | Organic/Integrated | 0.049563 |
| 2.584677 | ASV121_Bacillus | Spring | Organic/Integrated | 0.609682 |
| 3.486183 | ASV1217_Asanoa | Spring | Organic/Integrated | 0.04486 |
| -2.64223 | ASV1220_Arenimonas | Spring | Organic/Integrated | 0.046799 |
| 3.126208 | ASV1222_Chthoniobacter | Spring | Organic/Integrated | 0.087388 |
| 3.426694 | ASV1228_Sphingomonas | Spring | Organic/Integrated | 0.05556 |
| 4.273889 | ASV1250_Edaphobaculum | Spring | Organic/Integrated | 0.0825 |
| -3.70823 | ASV1273_Microvirga | Spring | Organic/Integrated | 0.044377 |
| -3.23904 | ASV1275_Unclassified_mle1-27 | Spring | Organic/Integrated | 0.045458 |
| 1.163721 | ASV128_Luteolibacter | Spring | Organic/Integrated | 0.400758 |
| -2.43772 | ASV1282_Unclassified_SC-I-84 | Spring | Organic/Integrated | 0.032285 |
| 3.892682 | ASV1287_Unclassified_Gemmatimonadaceae | Spring | Organic/Integrated | 0.073383 |
| 2.551358 | ASV1302_Unclassified_Comamonadaceae | Spring | Organic/Integrated | 0.064703 |
| 4.072417 | ASV1312_Unclassified_Xanthobacteraceae | Spring | Organic/Integrated | 0.065419 |
| 3.777801 | ASV1324_Unclassified_11-24 | Spring | Organic/Integrated | 0.096588 |
| 1.676495 | ASV1329_Unclassified_Xanthobacteraceae | Spring | Organic/Integrated | 0.035709 |
| 3.783915 | ASV1331_Unclassified_Gemmatimonadaceae | Spring | Organic/Integrated | 0.051983 |
| 3.634709 | ASV1335_Dongia | Spring | Organic/Integrated | 0.062639 |
| 3.646047 | ASV1353_Unclassified_Chitinophagaceae | Spring | Organic/Integrated | 0.07529 |
| 2.263977 | ASV1356_Unclassified_SC-I-84 | Spring | Organic/Integrated | 0.042279 |
| 3.900299 | ASV1366_Unclassified_Acidobacteriales | Spring | Organic/Integrated | 0.095592 |
| 3.819834 | ASV1377_Unclassified_Blastocatellaceae | Spring | Organic/Integrated | 0.055284 |
| -3.94167 | ASV1380_Unclassified_JG30-KF-CM45 | Spring | Organic/Integrated | 0.111923 |
| -1.20753 | ASV139_Nakamurella | Spring | Organic/Integrated | 0.317524 |
| -1.37753 | ASV14_Pedobacter | Spring | Organic/Integrated | 2.268217 |
| -3.69088 | ASV1400_Jatrophihabitans | Spring | Organic/Integrated | 0.051128 |
| 3.142822 | ASV1420_Unclassified_Gemmatimonadaceae | Spring | Organic/Integrated | 0.032657 |
| 1.141226 | ASV143_Phyllobacterium | Spring | Organic/Integrated | 0.353615 |
| -1.98417 | ASV1433_Chthoniobacter | Spring | Organic/Integrated | 0.038302 |
| 3.230895 | ASV1441_JGI 0001001-H03 | Spring | Organic/Integrated | 0.045342 |
| -2.73414 | ASV145_Pedobacter | Spring | Organic/Integrated | 0.665943 |
| 2.646759 | ASV1455_Unclassified_Alphaproteobacteria | Spring | Organic/Integrated | 0.040897 |
| 3.458152 | ASV1458_RB41 | Spring | Organic/Integrated | 0.055225 |
| -3.14086 | ASV1486_Unclassified_67-14 | Spring | Organic/Integrated | 0.038908 |
| -2.69622 | ASV1487_Unclassified_C0119 | Spring | Organic/Integrated | 0.036101 |
| -2.76249 | ASV1488_Nitrolancea | Spring | Organic/Integrated | 0.039359 |
| 3.729281 | ASV1500_Afipia | Spring | Organic/Integrated | 0.040857 |
| -2.91657 | ASV1504_Edaphobaculum | Spring | Organic/Integrated | 0.031866 |
| -2.89212 | ASV1506_Pseudolabrys | Spring | Organic/Integrated | 0.035896 |
| -2.32718 | ASV1519_Rhodanobacter | Spring | Organic/Integrated | 0.012826 |
| -3.39551 | ASV1542_Unclassified_Diplorickettsiaceae | Spring | Organic/Integrated | 0.040765 |
| 1.071608 | ASV155_Unclassified_Gemmatimonadaceae | Spring | Organic/Integrated | 0.214868 |
| 2.950748 | ASV1563_Unclassified_JG30-KF-CM45 | Spring | Organic/Integrated | 0.02642 |
| 2.949211 | ASV1581_Unclassified_Intrasporangiaceae | Spring | Organic/Integrated | 0.044342 |
| 3.812485 | ASV1591_RB41 | Spring | Organic/Integrated | 0.0535 |
| 3.244297 | ASV1592_Mycobacterium | Spring | Organic/Integrated | 0.030848 |
| 3.422586 | ASV1599_Unclassified_Chitinophagaceae | Spring | Organic/Integrated | 0.047171 |
| -3.67333 | ASV1606_Unclassified_Acetobacteraceae | Spring | Organic/Integrated | 0.079791 |
| 2.393509 | ASV1609_Unclassified_Subgroup 17 | Spring | Organic/Integrated | 0.042972 |
| -1.06751 | ASV161_Rhodoferax | Spring | Organic/Integrated | 0.115203 |
| 2.836429 | ASV1612_Unclassified_SC-I-84 | Spring | Organic/Integrated | 0.03235 |
| -2.41042 | ASV1626_Unclassified_JG30-KF-CM45 | Spring | Organic/Integrated | 0.02684 |
| -3.01676 | ASV1648_Sphaerisporangium | Spring | Organic/Integrated | 0.025749 |
| 2.341657 | ASV1665_Unclassified_UA11 | Spring | Organic/Integrated | 0.040971 |
| -3.22597 | ASV1692_Flexivirga | Spring | Organic/Integrated | 0.042836 |
| -1.59652 | ASV17_Sphingomonas | Spring | Organic/Integrated | 4.618868 |
| 2.421087 | ASV1743_Unclassified_WPS-2 | Spring | Organic/Integrated | 0.025743 |
| 3.19521 | ASV1744_Unclassified_Vicinamibacteraceae | Spring | Organic/Integrated | 0.04371 |
| 3.235976 | ASV1745_Unclassified_MB-A2-108 | Spring | Organic/Integrated | 0.024786 |
| -3.42062 | ASV1752_Unclassified_Xanthomonadaceae | Spring | Organic/Integrated | 0.06159 |
| 3.597516 | ASV1753_Nocardioides | Spring | Organic/Integrated | 0.067488 |
| 3.679072 | ASV1754_Unclassified_Sporichthyaceae | Spring | Organic/Integrated | 0.047582 |
| 3.058425 | ASV1759_Rhodoplanes | Spring | Organic/Integrated | 0.025891 |
| 3.630842 | ASV1763_Candidatus Udaeobacter | Spring | Organic/Integrated | 0.054467 |
| -1.9711 | ASV178_Asticcacaulis | Spring | Organic/Integrated | 0.509968 |
| 3.633693 | ASV1793_Reyranella | Spring | Organic/Integrated | 0.079259 |
| -2.7049 | ASV1806_Nocardioides | Spring | Organic/Integrated | 0.025804 |
| 1.72354 | ASV182_Unclassified_Acidobacteriales | Spring | Organic/Integrated | 0.201756 |
| -1.78476 | ASV183_Asticcacaulis | Spring | Organic/Integrated | 0.189217 |
| 3.165229 | ASV1846_RB41 | Spring | Organic/Integrated | 0.039672 |
| 1.927236 | ASV1883_Unclassified_Verrucomicrobiaceae | Spring | Organic/Integrated | 0.029765 |
| 3.065185 | ASV1896_Candidatus Solibacter | Spring | Organic/Integrated | 0.024184 |
| 1.878823 | ASV1905_Bdellovibrio | Spring | Organic/Integrated | 0.01829 |
| 2.572071 | ASV1917_Candidatus Udaeobacter | Spring | Organic/Integrated | 0.022355 |
| 3.28307 | ASV1942_Luteolibacter | Spring | Organic/Integrated | 0.030637 |
| -1.06273 | ASV20_Massilia | Spring | Organic/Integrated | 0.599565 |
| 1.445694 | ASV202_Unclassified_Rhizobiales Incertae Sedis | Spring | Organic/Integrated | 0.210704 |
| 3.233677 | ASV2065_Paenibacillus | Spring | Organic/Integrated | 0.0456 |
| 2.753364 | ASV2099_Subgroup 10 | Spring | Organic/Integrated | 0.024312 |
| 2.620743 | ASV2106_Unclassified_Subgroup 5 | Spring | Organic/Integrated | 0.034921 |
| 2.892148 | ASV2131_Arcticibacter | Spring | Organic/Integrated | 0.027757 |
| -1.75386 | ASV215_Gemmatimonas | Spring | Organic/Integrated | 0.219305 |
| 2.813422 | ASV2156_Unclassified_Micromonosporaceae | Spring | Organic/Integrated | 0.036278 |
| -1.75736 | ASV219_Mycobacterium | Spring | Organic/Integrated | 0.176562 |
| 2.376151 | ASV2196_Mycobacterium | Spring | Organic/Integrated | 0.019859 |
| 2.57764 | ASV2233_Lysobacter | Spring | Organic/Integrated | 0.020323 |
| 2.613564 | ASV2250_Unclassified_Chitinophagaceae | Spring | Organic/Integrated | 0.030441 |
| -1.82722 | ASV230_Sphingobium | Spring | Organic/Integrated | 0.257922 |
| 2.370653 | ASV2302_Sphingoaurantiacus | Spring | Organic/Integrated | 0.015506 |
| 2.943968 | ASV2369_Unclassified_JG30-KF-CM45 | Spring | Organic/Integrated | 0.035681 |
| -1.29634 | ASV244_Pseudomonas | Spring | Organic/Integrated | 0.090904 |
| 2.704131 | ASV249_Dyadobacter | Spring | Organic/Integrated | 0.31185 |
| 1.351119 | ASV250_Arenimonas | Spring | Organic/Integrated | 0.164311 |
| -1.7103 | ASV254_Rhodoferax | Spring | Organic/Integrated | 0.364256 |
| 2.934776 | ASV2564_Legionella | Spring | Organic/Integrated | 0.038705 |
| 2.534601 | ASV258_Dyadobacter | Spring | Organic/Integrated | 0.574975 |
| -1.07223 | ASV26_Acidovorax | Spring | Organic/Integrated | 1.371227 |
| 2.606353 | ASV2618_Acidibacter | Spring | Organic/Integrated | 0.023843 |
| -2.80392 | ASV2623_Unclassified_Pedosphaeraceae | Spring | Organic/Integrated | 0.028425 |
| 1.592106 | ASV263_Unclassified_Gemmatimonadaceae | Spring | Organic/Integrated | 0.192983 |
| -2.58418 | ASV265_Acidipila-Silvibacterium | Spring | Organic/Integrated | 0.162891 |
| 1.580545 | ASV269_Pseudaminobacter | Spring | Organic/Integrated | 0.131232 |
| -2.73254 | ASV2694_Anaeromyxobacter | Spring | Organic/Integrated | 0.035333 |
| 2.794702 | ASV2697_Nocardia | Spring | Organic/Integrated | 0.024534 |
| -2.79761 | ASV271_Bradyrhizobium | Spring | Organic/Integrated | 0.230094 |
| 2.809697 | ASV2782_Chthoniobacter | Spring | Organic/Integrated | 0.025739 |
| -1.94988 | ASV283_Mucilaginibacter | Spring | Organic/Integrated | 0.185927 |
| -2.0563 | ASV284_Flavobacterium | Spring | Organic/Integrated | 0.209794 |
| 2.183685 | ASV2902_Sphingomonas | Spring | Organic/Integrated | 0.015577 |
| -1.69687 | ASV297_Gemmatimonas | Spring | Organic/Integrated | 0.118475 |
| -2.20774 | ASV298_Sphingomonas | Spring | Organic/Integrated | 0.212557 |
| -1.85417 | ASV299_Bacillus | Spring | Organic/Integrated | 0.183476 |
| -3.05575 | ASV303_Nakamurella | Spring | Organic/Integrated | 0.398807 |
| 2.092934 | ASV306_Lysobacter | Spring | Organic/Integrated | 0.233033 |
| -2.39011 | ASV308_Unclassified_SC-I-84 | Spring | Organic/Integrated | 0.198992 |
| 1.993363 | ASV309_CL500-29 marine group | Spring | Organic/Integrated | 0.197454 |
| 1.495337 | ASV310_Afipia | Spring | Organic/Integrated | 0.144163 |
| 2.275516 | ASV321_Unclassified_Xanthobacteraceae | Spring | Organic/Integrated | 0.131468 |
| 2.411171 | ASV323_Unclassified_Blastocatellaceae | Spring | Organic/Integrated | 0.154235 |
| -1.47621 | ASV327_Acidiphilium | Spring | Organic/Integrated | 0.106211 |
| 1.626386 | ASV338_Dyadobacter | Spring | Organic/Integrated | 0.29849 |
| -2.32675 | ASV3383_Gemmatirosa | Spring | Organic/Integrated | 0.014722 |
| -5.97578 | ASV339_Pseudarthrobacter | Spring | Organic/Integrated | 0.273377 |
| 1.837309 | ASV345_Pseudolabrys | Spring | Organic/Integrated | 0.105707 |
| 2.539005 | ASV349_Unclassified_Saprospiraceae | Spring | Organic/Integrated | 0.214299 |
| 1.323606 | ASV375_Glycomyces | Spring | Organic/Integrated | 0.128049 |
| -3.80602 | ASV377_Actinoallomurus | Spring | Organic/Integrated | 0.569798 |
| -1.05776 | ASV38_Glaciihabitans | Spring | Organic/Integrated | 1.523834 |
| 6.574648 | ASV380_Unclassified_Verrucomicrobiaceae | Spring | Organic/Integrated | 0.5101 |
| 1.743815 | ASV381_Nordella | Spring | Organic/Integrated | 0.102494 |
| -2.90418 | ASV383_Devosia | Spring | Organic/Integrated | 0.252662 |
| -2.21914 | ASV392_Acidipila-Silvibacterium | Spring | Organic/Integrated | 0.125182 |
| 2.860404 | ASV395_Nocardioides | Spring | Organic/Integrated | 0.128946 |
| -2.76455 | ASV40_Pedobacter | Spring | Organic/Integrated | 1.184173 |
| 3.266894 | ASV400_Pedobacter | Spring | Organic/Integrated | 0.253811 |
| 2.228766 | ASV407_Pedomicrobium | Spring | Organic/Integrated | 0.097917 |
| -2.9551 | ASV420_Rhodopseudomonas | Spring | Organic/Integrated | 0.136693 |
| 1.172151 | ASV426_Unclassified_IMCC26256 | Spring | Organic/Integrated | 0.10228 |
| 1.796165 | ASV430_Rhizobacter | Spring | Organic/Integrated | 0.120229 |
| 0.981259 | ASV44_Allorhizobium-Neorhizobium-Pararhizobium-Rhizobium | Spring | Organic/Integrated | 0.793352 |
| 1.317945 | ASV440_agricultural soil bacterium SC-I-84 | Spring | Organic/Integrated | 0.081216 |
| 2.595741 | ASV446_Mycobacterium | Spring | Organic/Integrated | 0.108597 |
| 5.701618 | ASV448_Phyllobacterium | Spring | Organic/Integrated | 0.20526 |
| 3.491805 | ASV449_Verrucomicrobium | Spring | Organic/Integrated | 0.228523 |
| -2.6231 | ASV452_Hyphomicrobium | Spring | Organic/Integrated | 0.106383 |
| 1.997602 | ASV467_Nordella | Spring | Organic/Integrated | 0.110003 |
| 1.49344 | ASV468_Terrimonas | Spring | Organic/Integrated | 0.077226 |
| -2.89716 | ASV482_Unclassified_Chitinophagaceae | Spring | Organic/Integrated | 0.144617 |
| 3.851945 | ASV491_Ferruginibacter | Spring | Organic/Integrated | 0.401707 |
| 3.059182 | ASV498_Unclassified_Blastocatellaceae | Spring | Organic/Integrated | 0.117618 |
| 3.018099 | ASV511_Paeniglutamicibacter | Spring | Organic/Integrated | 0.146752 |
| 5.059615 | ASV514_Leifsonia | Spring | Organic/Integrated | 0.192428 |
| 2.892855 | ASV518_Catellatospora | Spring | Organic/Integrated | 0.12242 |
| 2.802083 | ASV522_Unclassified_KD4-96 | Spring | Organic/Integrated | 0.095314 |
| 3.751818 | ASV523_Streptomyces | Spring | Organic/Integrated | 0.153105 |
| 2.583433 | ASV526_Sphingomonas | Spring | Organic/Integrated | 0.109743 |
| -1.92266 | ASV532_Rhodopseudomonas | Spring | Organic/Integrated | 0.063167 |
| 2.890258 | ASV535_Solirubrobacter | Spring | Organic/Integrated | 0.158517 |
| -2.47854 | ASV536_Mesorhizobium | Spring | Organic/Integrated | 0.083656 |
| 3.619227 | ASV554_Rhizobacter | Spring | Organic/Integrated | 0.163718 |
| 1.297689 | ASV56_Bacillus | Spring | Organic/Integrated | 1.212352 |
| -2.31461 | ASV575_Unclassified_Acetobacteraceae | Spring | Organic/Integrated | 0.090873 |
| -1.99595 | ASV576_Micromonospora | Spring | Organic/Integrated | 0.150982 |
| 5.179999 | ASV582_Mycobacterium | Spring | Organic/Integrated | 0.10758 |
| 2.969344 | ASV588_Hyphomicrobium | Spring | Organic/Integrated | 0.081035 |
| 1.707309 | ASV598_Tahibacter | Spring | Organic/Integrated | 0.058306 |
| -1.49984 | ASV599_Dokdonella | Spring | Organic/Integrated | 0.067589 |
| 2.076834 | ASV601_Unclassified_Gitt-GS-136 | Spring | Organic/Integrated | 0.100173 |
| 2.041465 | ASV607_Sphingomonas | Spring | Organic/Integrated | 0.116756 |
| -3.11339 | ASV609_Tahibacter | Spring | Organic/Integrated | 0.077702 |
| 1.795652 | ASV610_Unclassified_Gemmatimonadaceae | Spring | Organic/Integrated | 0.077836 |
| 3.141967 | ASV613_Marmoricola | Spring | Organic/Integrated | 0.12079 |
| -1.39882 | ASV614_Methylotenera | Spring | Organic/Integrated | 0.05553 |
| -5.04174 | ASV623_Dokdonella | Spring | Organic/Integrated | 0.098838 |
| 4.643892 | ASV628_Candidatus Solibacter | Spring | Organic/Integrated | 0.095078 |
| -1.20256 | ASV63_Unclassified_Microbacteriaceae | Spring | Organic/Integrated | 0.473034 |
| -3.0573 | ASV637_Unclassified_SC-I-84 | Spring | Organic/Integrated | 0.098447 |
| 2.40257 | ASV639_Flavisolibacter | Spring | Organic/Integrated | 0.082823 |
| -4.51438 | ASV652_Bryobacter | Spring | Organic/Integrated | 0.098663 |
| 1.580986 | ASV654_Chitinophaga | Spring | Organic/Integrated | 0.083323 |
| 2.803672 | ASV656_Pseudolabrys | Spring | Organic/Integrated | 0.073326 |
| -1.62171 | ASV661_Unclassified_Micavibrionales | Spring | Organic/Integrated | 0.05175 |
| -2.65455 | ASV668_Mizugakiibacter | Spring | Organic/Integrated | 0.101041 |
| 2.873386 | ASV675_Unclassified_KD4-96 | Spring | Organic/Integrated | 0.095866 |
| 3.138349 | ASV682_Pseudomonas | Spring | Organic/Integrated | 0.35061 |
| 4.049978 | ASV686_Luteolibacter | Spring | Organic/Integrated | 0.12936 |
| -3.02521 | ASV691_Rhodanobacter | Spring | Organic/Integrated | 0.049831 |
| 2.715875 | ASV694_Unclassified_Chitinophagaceae | Spring | Organic/Integrated | 0.069845 |
| 3.667152 | ASV696_Ferruginibacter | Spring | Organic/Integrated | 0.083459 |
| 4.330047 | ASV701_Solirubrobacter | Spring | Organic/Integrated | 0.08942 |
| 3.260999 | ASV706_Unclassified_Blastocatellaceae | Spring | Organic/Integrated | 0.082043 |
| 4.62252 | ASV709_Unclassified_Blastocatellaceae | Spring | Organic/Integrated | 0.078344 |
| 1.771567 | ASV711_Pseudoxanthomonas | Spring | Organic/Integrated | 0.09134 |
| 2.814769 | ASV715_Pseudoxanthomonas | Spring | Organic/Integrated | 0.261938 |
| -2.17269 | ASV721_Nocardia | Spring | Organic/Integrated | 0.083998 |
| 2.380451 | ASV722_Lysinimonas | Spring | Organic/Integrated | 0.103428 |
| 1.922305 | ASV724_Chthoniobacter | Spring | Organic/Integrated | 0.081878 |
| 2.419812 | ASV726_Unclassified_Chitinophagaceae | Spring | Organic/Integrated | 0.08844 |
| 2.110535 | ASV754_RB41 | Spring | Organic/Integrated | 0.064728 |
| 1.545736 | ASV758_Pseudomonas | Spring | Organic/Integrated | 0.103646 |
| 4.674517 | ASV760_Unclassified_Rhizobiales Incertae Sedis | Spring | Organic/Integrated | 0.117904 |
| 4.000614 | ASV767_Actimicrobium | Spring | Organic/Integrated | 0.126903 |
| 4.466807 | ASV771_RB41 | Spring | Organic/Integrated | 0.09696 |
| -3.56262 | ASV788_Flavobacterium | Spring | Organic/Integrated | 0.109413 |
| 2.707681 | ASV792_Chthoniobacter | Spring | Organic/Integrated | 0.11307 |
| 2.717793 | ASV802_Unclassified_A21b | Spring | Organic/Integrated | 0.053835 |
| -3.9258 | ASV804_Microbispora | Spring | Organic/Integrated | 0.065136 |
| 2.878333 | ASV806_Bradyrhizobium | Spring | Organic/Integrated | 0.048945 |
| 3.393104 | ASV815_Gaiella | Spring | Organic/Integrated | 0.082409 |
| 4.611416 | ASV822_Rhodoferax | Spring | Organic/Integrated | 0.093249 |
| 3.344642 | ASV823_Streptomyces | Spring | Organic/Integrated | 0.075015 |
| -2.48397 | ASV827_Patulibacter | Spring | Organic/Integrated | 0.14985 |
| -2.18558 | ASV828_Burkholderia-Caballeronia-Paraburkholderia | Spring | Organic/Integrated | 0.058771 |
| 1.680555 | ASV829_Flavitalea | Spring | Organic/Integrated | 0.062704 |
| -4.43178 | ASV839_Flavobacterium | Spring | Organic/Integrated | 0.095005 |
| 2.367627 | ASV840_Unclassified_Xanthobacteraceae | Spring | Organic/Integrated | 0.056904 |
| 4.350736 | ASV842_Candidatus Udaeobacter | Spring | Organic/Integrated | 0.090284 |
| 2.173554 | ASV845_Bacillus | Spring | Organic/Integrated | 0.06338 |
| 2.921254 | ASV852_Unclassified_Micropepsaceae | Spring | Organic/Integrated | 0.081291 |
| 2.941831 | ASV853_Unclassified_Azospirillales | Spring | Organic/Integrated | 0.071416 |
| 2.243538 | ASV855_Unclassified_Xanthobacteraceae | Spring | Organic/Integrated | 0.079639 |
| 4.237764 | ASV876_Unclassified_SC-I-84 | Spring | Organic/Integrated | 0.058948 |
| 4.51741 | ASV881_Luteolibacter | Spring | Organic/Integrated | 0.093287 |
| -3.65927 | ASV891_Acidothermus | Spring | Organic/Integrated | 0.088056 |
| 2.81212 | ASV897_Nordella | Spring | Organic/Integrated | 0.053589 |
| 2.913133 | ASV911_Nordella | Spring | Organic/Integrated | 0.055182 |
| 2.59689 | ASV919_Luteolibacter | Spring | Organic/Integrated | 0.073162 |
| -3.04219 | ASV921_Mucilaginibacter | Spring | Organic/Integrated | 0.076375 |
| 2.732197 | ASV925_Nordella | Spring | Organic/Integrated | 0.048726 |
| 1.860161 | ASV926_Unclassified_Microtrichales | Spring | Organic/Integrated | 0.042351 |
| 1.228713 | ASV93_Mesorhizobium | Spring | Organic/Integrated | 0.360476 |
| -4.19149 | ASV933_Mycobacterium | Spring | Organic/Integrated | 0.072341 |
| 1.604264 | ASV94_Allorhizobium-Neorhizobium-Pararhizobium-Rhizobium | Spring | Organic/Integrated | 0.485012 |
| -4.07539 | ASV940_Reyranella | Spring | Organic/Integrated | 0.070323 |
| -4.40312 | ASV946_Unclassified_Caulobacteraceae | Spring | Organic/Integrated | 0.100461 |
| -1.76007 | ASV95_Pedobacter | Spring | Organic/Integrated | 0.199085 |
| 3.858329 | ASV952_Unclassified_PLTA13 | Spring | Organic/Integrated | 0.057495 |
| -2.00711 | ASV96_Devosia | Spring | Organic/Integrated | 0.540341 |
| 2.509371 | ASV966_Dokdonella | Spring | Organic/Integrated | 0.053857 |
| -1.74173 | ASV968_Unclassified_Obscuribacteraceae | Spring | Organic/Integrated | 0.079791 |
| 3.014737 | ASV973_Unclassified_Xanthobacteraceae | Spring | Organic/Integrated | 0.065559 |
| 1.933895 | ASV974_Unclassified_Gaiellales | Spring | Organic/Integrated | 0.043714 |
| 4.19252 | ASV975_Nocardioides | Spring | Organic/Integrated | 0.097665 |
| 3.678911 | ASV976_Bradyrhizobium | Spring | Organic/Integrated | 0.08297 |
| 0.9611 | ASV98_Streptomyces | Spring | Organic/Integrated | 0.392409 |
| -2.86179 | ASV991_Streptacidiphilus | Spring | Organic/Integrated | 0.053126 |
| -0.79721 | ASV100_Ralstonia | Autumn | Organic/Integrated | 0.300734 |
| 1.46391 | ASV101_Phyllobacterium | Autumn | Organic/Integrated | 0.687532 |
| -3.82357 | ASV1010_Massilia | Autumn | Organic/Integrated | 0.092544 |
| -3.23718 | ASV1011_Gemmatimonas | Autumn | Organic/Integrated | 0.041332 |
| 4.166736 | ASV1017_Aminobacter | Autumn | Organic/Integrated | 0.071501 |
| -2.15349 | ASV1020_JGI 0001001-H03 | Autumn | Organic/Integrated | 0.072755 |
| -1.21733 | ASV1028_Unclassified_Micropepsaceae | Autumn | Organic/Integrated | 0.033681 |
| 1.882096 | ASV103_Sphingomonas | Autumn | Organic/Integrated | 0.457271 |
| -1.98729 | ASV1034_Unclassified_Roseiflexaceae | Autumn | Organic/Integrated | 0.049488 |
| -4.44562 | ASV104_Pedobacter | Autumn | Organic/Integrated | 3.747102 |
| -3.3109 | ASV1040_Unclassified_B12-WMSP1 | Autumn | Organic/Integrated | 0.054743 |
| 3.833346 | ASV1049_Unclassified_SC-I-84 | Autumn | Organic/Integrated | 0.056717 |
| 3.825353 | ASV1059_Flavobacterium | Autumn | Organic/Integrated | 0.090485 |
| 2.051057 | ASV106_Stappia | Autumn | Organic/Integrated | 0.531175 |
| -3.54586 | ASV1061_Unclassified_Acidobacteriales | Autumn | Organic/Integrated | 0.04393 |
| -2.00294 | ASV1062_Mycobacterium | Autumn | Organic/Integrated | 0.035898 |
| 2.059414 | ASV107_Dyadobacter | Autumn | Organic/Integrated | 0.76955 |
| -4.50836 | ASV1077_Rhodoferax | Autumn | Organic/Integrated | 0.097401 |
| 4.499591 | ASV1082_Rhizobacter | Autumn | Organic/Integrated | 0.139522 |
| 3.593145 | ASV1085_Acidibacter | Autumn | Organic/Integrated | 0.083419 |
| 4.392068 | ASV1094_Pedobacter | Autumn | Organic/Integrated | 0.098962 |
| 3.561036 | ASV1095_Unclassified_Steroidobacteraceae | Autumn | Organic/Integrated | 0.049929 |
| -4.03566 | ASV1102_Unclassified_Micropepsaceae | Autumn | Organic/Integrated | 0.052742 |
| -4.20391 | ASV1105_Unclassified_Acidobacteriales | Autumn | Organic/Integrated | 0.057481 |
| 2.278308 | ASV1116_Larkinella | Autumn | Organic/Integrated | 0.042113 |
| 3.687047 | ASV1123_Pseudomonas | Autumn | Organic/Integrated | 0.043344 |
| -3.45978 | ASV1128_Unclassified_Acidobacteriales | Autumn | Organic/Integrated | 0.055696 |
| -3.86887 | ASV1137_Unclassified_Acetobacteraceae | Autumn | Organic/Integrated | 0.061714 |
| 3.550377 | ASV1140_Ferruginibacter | Autumn | Organic/Integrated | 0.060718 |
| 3.564637 | ASV1144_Lysobacter | Autumn | Organic/Integrated | 0.040507 |
| 3.528087 | ASV1159_Stenotrophomonas | Autumn | Organic/Integrated | 0.044247 |
| 1.662535 | ASV116_Pseudoxanthomonas | Autumn | Organic/Integrated | 0.406934 |
| -3.62992 | ASV1169_Unclassified_Chitinophagaceae | Autumn | Organic/Integrated | 0.039609 |
| 2.557671 | ASV1180_Lysobacter | Autumn | Organic/Integrated | 0.050085 |
| -3.81891 | ASV1185_Lysobacter | Autumn | Organic/Integrated | 0.050305 |
| 3.876922 | ASV1201_Herpetosiphon | Autumn | Organic/Integrated | 0.054947 |
| -3.95593 | ASV1218_Unclassified_SC-I-84 | Autumn | Organic/Integrated | 0.068022 |
| -2.33135 | ASV1224_Unclassified_Gaiellales | Autumn | Organic/Integrated | 0.037402 |
| -4.2404 | ASV1231_Unclassified_Oxalobacteraceae | Autumn | Organic/Integrated | 0.084642 |
| -2.88318 | ASV1240_Massilia | Autumn | Organic/Integrated | 0.060088 |
| 3.20081 | ASV1251_Reyranella | Autumn | Organic/Integrated | 0.041765 |
| -4.10613 | ASV1266_Mucilaginibacter | Autumn | Organic/Integrated | 0.063354 |
| -2.22779 | ASV1274_Asticcacaulis | Autumn | Organic/Integrated | 0.050323 |
| -2.66172 | ASV1279_Unclassified_Caulobacteraceae | Autumn | Organic/Integrated | 0.041253 |
| 1.221012 | ASV128_Luteolibacter | Autumn | Organic/Integrated | 0.169585 |
| 3.430018 | ASV1286_Sphingopyxis | Autumn | Organic/Integrated | 0.050333 |
| 3.296384 | ASV1289_Unclassified_Fimbriimonadaceae | Autumn | Organic/Integrated | 0.041895 |
| -2.23012 | ASV1291_Unclassified_Chitinophagaceae | Autumn | Organic/Integrated | 0.066411 |
| -3.29195 | ASV1300_Unclassified_Xanthobacteraceae | Autumn | Organic/Integrated | 0.059338 |
| -3.01336 | ASV1305_Mucilaginibacter | Autumn | Organic/Integrated | 0.081008 |
| 3.218936 | ASV1316_Pseudoflavitalea | Autumn | Organic/Integrated | 0.038455 |
| -3.37985 | ASV1320_Mucilaginibacter | Autumn | Organic/Integrated | 0.049266 |
| -1.88418 | ASV1342_Chthoniobacter | Autumn | Organic/Integrated | 0.061633 |
| 4.137965 | ASV1344_Massilia | Autumn | Organic/Integrated | 0.109668 |
| -3.87197 | ASV1346_Mucilaginibacter | Autumn | Organic/Integrated | 0.070977 |
| -3.3977 | ASV1351_Unclassified_Chitinophagaceae | Autumn | Organic/Integrated | 0.04206 |
| -1.61375 | ASV1352_Taibaiella | Autumn | Organic/Integrated | 0.030541 |
| -3.07086 | ASV1364_Unclassified_Acidobacteriales | Autumn | Organic/Integrated | 0.030466 |
| -3.94025 | ASV1372_Unclassified_Oxalobacteraceae | Autumn | Organic/Integrated | 0.055201 |
| -3.78394 | ASV1394_Candidatus Ovatusbacter | Autumn | Organic/Integrated | 0.050126 |
| -2.75491 | ASV1399_Bauldia | Autumn | Organic/Integrated | 0.041229 |
| 3.595033 | ASV14_Pedobacter | Autumn | Organic/Integrated | 20.81788 |
| 3.27551 | ASV1414_Flavobacterium | Autumn | Organic/Integrated | 0.04032 |
| 3.065766 | ASV1420_Unclassified_Gemmatimonadaceae | Autumn | Organic/Integrated | 0.027543 |
| 3.380885 | ASV1421_Sphingomonas | Autumn | Organic/Integrated | 0.046183 |
| -2.59395 | ASV1434_Unclassified_C0119 | Autumn | Organic/Integrated | 0.027206 |
| 3.460923 | ASV1457_Mucilaginibacter | Autumn | Organic/Integrated | 0.047643 |
| -1.12453 | ASV146_Paenibacillus | Autumn | Organic/Integrated | 0.384608 |
| 3.891687 | ASV1465_Unclassified_Comamonadaceae | Autumn | Organic/Integrated | 0.043399 |
| 3.026335 | ASV1466_Pseudonocardia | Autumn | Organic/Integrated | 0.030803 |
| 2.68364 | ASV1478_Pedobacter | Autumn | Organic/Integrated | 0.026023 |
| -2.74465 | ASV148_Chitinophaga | Autumn | Organic/Integrated | 0.822723 |
| -2.60558 | ASV1484_Unclassified_Subgroup 7 | Autumn | Organic/Integrated | 0.071217 |
| 3.741831 | ASV1490_Unclassified_BIrii41 | Autumn | Organic/Integrated | 0.063537 |
| 3.059642 | ASV1497_Unclassified_C0119 | Autumn | Organic/Integrated | 0.043452 |
| 3.476382 | ASV1498_Microvirga | Autumn | Organic/Integrated | 0.038524 |
| -2.54493 | ASV1504_Edaphobaculum | Autumn | Organic/Integrated | 0.030474 |
| 1.15998 | ASV151_Paenarthrobacter | Autumn | Organic/Integrated | 0.282988 |
| -3.61921 | ASV1515_Bryobacter | Autumn | Organic/Integrated | 0.065525 |
| -2.77115 | ASV1519_Rhodanobacter | Autumn | Organic/Integrated | 0.027706 |
| -3.36122 | ASV1522_Massilia | Autumn | Organic/Integrated | 0.055814 |
| 2.992655 | ASV1537_Unclassified_Gemmatimonadaceae | Autumn | Organic/Integrated | 0.031771 |
| -1.67657 | ASV156_Dyadobacter | Autumn | Organic/Integrated | 0.457119 |
| 2.915688 | ASV1563_Unclassified_JG30-KF-CM45 | Autumn | Organic/Integrated | 0.034969 |
| -3.13185 | ASV1569_Massilia | Autumn | Organic/Integrated | 0.0457 |
| 2.094764 | ASV157_Limnohabitans | Autumn | Organic/Integrated | 0.337995 |
| -0.72086 | ASV16_Bacillus | Autumn | Organic/Integrated | 2.025473 |
| 2.565353 | ASV1608_Methylobacterium-Methylorubrum | Autumn | Organic/Integrated | 0.025105 |
| 2.532972 | ASV1612_Unclassified_SC-I-84 | Autumn | Organic/Integrated | 0.023345 |
| 2.683416 | ASV1618_Taibaiella | Autumn | Organic/Integrated | 0.038944 |
| 2.321726 | ASV163_Devosia | Autumn | Organic/Integrated | 0.421503 |
| -2.87106 | ASV1635_Unclassified_Acetobacteraceae | Autumn | Organic/Integrated | 0.027606 |
| -3.37565 | ASV1639_Unclassified_Microscillaceae | Autumn | Organic/Integrated | 0.055249 |
| -1.94752 | ASV1646_Unclassified_Microscillaceae | Autumn | Organic/Integrated | 0.041436 |
| 3.676521 | ASV1656_Corynebacterium | Autumn | Organic/Integrated | 0.084831 |
| -2.83721 | ASV1659_Unclassified_JG30-KF-CM66 | Autumn | Organic/Integrated | 0.02895 |
| 3.631235 | ASV1672_Actimicrobium | Autumn | Organic/Integrated | 0.050065 |
| -3.0817 | ASV1686_Unclassified_Vicinamibacteraceae | Autumn | Organic/Integrated | 0.029309 |
| 2.826691 | ASV169_Acidovorax | Autumn | Organic/Integrated | 0.619662 |
| 1.496734 | ASV1695_SWB02 | Autumn | Organic/Integrated | 0.022058 |
| 2.40591 | ASV170_Dyadobacter | Autumn | Organic/Integrated | 0.386926 |
| 2.868094 | ASV1702_Unclassified_TRA3-20 | Autumn | Organic/Integrated | 0.026168 |
| -3.13922 | ASV1704_Unclassified_Xanthobacteraceae | Autumn | Organic/Integrated | 0.040904 |
| -2.02658 | ASV1708_Parafilimonas | Autumn | Organic/Integrated | 0.023877 |
| 2.977028 | ASV1712_Pseudomonas | Autumn | Organic/Integrated | 0.02582 |
| -2.81745 | ASV1723_Unclassified_Candidatus Jidaibacter | Autumn | Organic/Integrated | 0.025605 |
| -3.12684 | ASV173_Duganella | Autumn | Organic/Integrated | 0.68299 |
| -1.8136 | ASV178_Asticcacaulis | Autumn | Organic/Integrated | 0.209316 |
| -2.51913 | ASV1781_Candidatus Udaeobacter | Autumn | Organic/Integrated | 0.022881 |
| 3.159407 | ASV1787_Cellvibrio | Autumn | Organic/Integrated | 0.04851 |
| 3.394343 | ASV1818_Dyadobacter | Autumn | Organic/Integrated | 0.038418 |
| 3.50911 | ASV1819_Unclassified_Roseiflexaceae | Autumn | Organic/Integrated | 0.050161 |
| -1.84531 | ASV183_Asticcacaulis | Autumn | Organic/Integrated | 0.29342 |
| 3.102763 | ASV1831_Unclassified_JG30-KF-CM45 | Autumn | Organic/Integrated | 0.052128 |
| -1.05382 | ASV184_Crossiella | Autumn | Organic/Integrated | 0.178145 |
| -3.13595 | ASV1851_Unclassified_Rhodanobacteraceae | Autumn | Organic/Integrated | 0.039406 |
| 4.080319 | ASV186_Flavobacterium | Autumn | Organic/Integrated | 0.619792 |
| -2.7486 | ASV1863_Rhodanobacter | Autumn | Organic/Integrated | 0.042997 |
| -1.90896 | ASV187_Unclassified_Acidobacteriales | Autumn | Organic/Integrated | 0.349876 |
| 2.555156 | ASV1890_IS-44 | Autumn | Organic/Integrated | 0.058182 |
| 0.791455 | ASV190_Bryobacter | Autumn | Organic/Integrated | 0.148224 |
| -2.27143 | ASV1915_Lysobacter | Autumn | Organic/Integrated | 0.023123 |
| -2.06856 | ASV193_Duganella | Autumn | Organic/Integrated | 0.690408 |
| -2.80648 | ASV1945_Jatrophihabitans | Autumn | Organic/Integrated | 0.027245 |
| 2.962225 | ASV1955_Burkholderia-Caballeronia-Paraburkholderia | Autumn | Organic/Integrated | 0.053827 |
| 2.49811 | ASV197_Allorhizobium-Neorhizobium-Pararhizobium-Rhizobium | Autumn | Organic/Integrated | 0.308662 |
| -3.34005 | ASV1973_Unclassified_Oxalobacteraceae | Autumn | Organic/Integrated | 0.051963 |
| 2.789286 | ASV1985_Microbacterium | Autumn | Organic/Integrated | 0.026107 |
| -1.13988 | ASV200_Polaromonas | Autumn | Organic/Integrated | 0.177076 |
| -1.97342 | ASV2014_Massilia | Autumn | Organic/Integrated | 0.019914 |
| -2.85444 | ASV2019_Unclassified_Chitinophagaceae | Autumn | Organic/Integrated | 0.024389 |
| 1.55854 | ASV202_Unclassified_Rhizobiales Incertae Sedis | Autumn | Organic/Integrated | 0.200461 |
| 1.841585 | ASV205_Mucilaginibacter | Autumn | Organic/Integrated | 0.285128 |
| 1.751214 | ASV2059_Unclassified_Chitinophagaceae | Autumn | Organic/Integrated | 0.024428 |
| -2.49565 | ASV2069_Massilia | Autumn | Organic/Integrated | 0.023851 |
| 2.169925 | ASV2075_Lysobacter | Autumn | Organic/Integrated | 0.02055 |
| 2.689215 | ASV2083_Burkholderia-Caballeronia-Paraburkholderia | Autumn | Organic/Integrated | 0.030508 |
| -2.07238 | ASV209_Massilia | Autumn | Organic/Integrated | 0.326082 |
| 2.540407 | ASV211_Massilia | Autumn | Organic/Integrated | 0.639995 |
| -2.51012 | ASV215_Gemmatimonas | Autumn | Organic/Integrated | 0.237146 |
| -3.10167 | ASV2173_Massilia | Autumn | Organic/Integrated | 0.049508 |
| 2.759519 | ASV221_Rhodoferax | Autumn | Organic/Integrated | 0.590666 |
| 3.058923 | ASV2214_Duganella | Autumn | Organic/Integrated | 0.034114 |
| 1.893314 | ASV2233_Lysobacter | Autumn | Organic/Integrated | 0.012958 |
| 2.451112 | ASV2234_Dokdonella | Autumn | Organic/Integrated | 0.018758 |
| -1.69463 | ASV225_Occallatibacter | Autumn | Organic/Integrated | 0.165124 |
| -3.94883 | ASV226_Serratia | Autumn | Organic/Integrated | 1.240225 |
| -1.67048 | ASV227_Unclassified_Gaiellales | Autumn | Organic/Integrated | 0.186611 |
| -2.02163 | ASV228_Duganella | Autumn | Organic/Integrated | 0.48601 |
| 2.4266 | ASV2303_Dokdonella | Autumn | Organic/Integrated | 0.017532 |
| -2.15921 | ASV2309_Massilia | Autumn | Organic/Integrated | 0.029242 |
| -1.91153 | ASV2331_Conexibacter | Autumn | Organic/Integrated | 0.017701 |
| -1.69463 | ASV2375_Unclassified_BIrii41 | Autumn | Organic/Integrated | 0.017577 |
| -2.84162 | ASV2377_Unclassified_Acidobacteriales | Autumn | Organic/Integrated | 0.04308 |
| -2.45513 | ASV2380_Massilia | Autumn | Organic/Integrated | 0.023861 |
| 2.606679 | ASV2386_Aquabacterium | Autumn | Organic/Integrated | 0.025221 |
| -1.71344 | ASV24_Brevundimonas | Autumn | Organic/Integrated | 4.317163 |
| -2.11829 | ASV240_Massilia | Autumn | Organic/Integrated | 0.379068 |
| 1.843709 | ASV243_Ferruginibacter | Autumn | Organic/Integrated | 0.188597 |
| 1.765638 | ASV246_Rhodoferax | Autumn | Organic/Integrated | 0.23034 |
| -1.09092 | ASV25_Allorhizobium-Neorhizobium-Pararhizobium-Rhizobium | Autumn | Organic/Integrated | 2.149403 |
| 2.23775 | ASV250_Arenimonas | Autumn | Organic/Integrated | 0.271804 |
| 2.743973 | ASV2578_Unclassified_Actinomarinales | Autumn | Organic/Integrated | 0.036611 |
| 2.856538 | ASV2579_Limnohabitans | Autumn | Organic/Integrated | 0.032667 |
| 1.974743 | ASV259_Devosia | Autumn | Organic/Integrated | 0.384581 |
| -2.7292 | ASV2600_Conexibacter | Autumn | Organic/Integrated | 0.029196 |
| -2.36746 | ASV265_Acidipila-Silvibacterium | Autumn | Organic/Integrated | 0.164504 |
| 1.517148 | ASV269_Pseudaminobacter | Autumn | Organic/Integrated | 0.121262 |
| -5.99731 | ASV271_Bradyrhizobium | Autumn | Organic/Integrated | 0.172546 |
| 2.045225 | ASV2715_Massilia | Autumn | Organic/Integrated | 0.013496 |
| 3.909748 | ASV280_Frigoribacterium | Autumn | Organic/Integrated | 0.087351 |
| 2.274411 | ASV2813_Unclassified_Sericytochromatia | Autumn | Organic/Integrated | 0.01715 |
| -2.49773 | ASV283_Mucilaginibacter | Autumn | Organic/Integrated | 0.149305 |
| -1.86146 | ASV29_Dyadobacter | Autumn | Organic/Integrated | 2.459375 |
| -2.07999 | ASV290_Luteolibacter | Autumn | Organic/Integrated | 0.05946 |
| 1.624695 | ASV291_Legionella | Autumn | Organic/Integrated | 0.104331 |
| 1.988358 | ASV295_Duganella | Autumn | Organic/Integrated | 0.378998 |
| -1.83383 | ASV297_Gemmatimonas | Autumn | Organic/Integrated | 0.150912 |
| -1.66238 | ASV298_Sphingomonas | Autumn | Organic/Integrated | 0.145559 |
| -4.38406 | ASV299_Bacillus | Autumn | Organic/Integrated | 0.124298 |
| 2.48151 | ASV30_Flavobacterium | Autumn | Organic/Integrated | 1.86689 |
| 2.320412 | ASV3022_Flavobacterium | Autumn | Organic/Integrated | 0.021219 |
| -1.33533 | ASV308_Unclassified_SC-I-84 | Autumn | Organic/Integrated | 0.086158 |
| 1.849487 | ASV309_CL500-29 marine group | Autumn | Organic/Integrated | 0.096271 |
| 2.416925 | ASV310_Afipia | Autumn | Organic/Integrated | 0.136554 |
| -3.38144 | ASV315_Pseudarcobacter | Autumn | Organic/Integrated | 0.117856 |
| 2.330128 | ASV3154_Aquibacter | Autumn | Organic/Integrated | 0.01925 |
| 6.043295 | ASV317_Sphingomonas | Autumn | Organic/Integrated | 0.238069 |
| 2.097706 | ASV323_Unclassified_Blastocatellaceae | Autumn | Organic/Integrated | 0.146013 |
| 2.155223 | ASV3249_Flavobacterium | Autumn | Organic/Integrated | 0.017303 |
| 5.513317 | ASV325_Sphingomonas | Autumn | Organic/Integrated | 0.19964 |
| -1.62576 | ASV327_Acidiphilium | Autumn | Organic/Integrated | 0.203548 |
| -1.79867 | ASV328_Unclassified_Subgroup 2 | Autumn | Organic/Integrated | 0.120989 |
| 1.300174 | ASV33_Devosia | Autumn | Organic/Integrated | 1.0797 |
| 1.534627 | ASV331_Microbacterium | Autumn | Organic/Integrated | 0.197665 |
| 2.109478 | ASV332_Limnohabitans | Autumn | Organic/Integrated | 0.190249 |
| 2.491893 | ASV333_Rhizobacter | Autumn | Organic/Integrated | 0.338782 |
| -2.43129 | ASV335_Mucilaginibacter | Autumn | Organic/Integrated | 0.273239 |
| -1.77996 | ASV341_Bacillus | Autumn | Organic/Integrated | 0.103174 |
| 4.59525 | ASV350_Sphingobium | Autumn | Organic/Integrated | 0.110527 |
| -2.07737 | ASV3516_Parafilimonas | Autumn | Organic/Integrated | 0.013935 |
| -3.32038 | ASV359_Chthoniobacter | Autumn | Organic/Integrated | 0.168439 |
| -3.38073 | ASV365_Unclassified_Acidobacteriales | Autumn | Organic/Integrated | 0.172253 |
| 1.235364 | ASV371_Sphingomonas | Autumn | Organic/Integrated | 0.174219 |
| -1.88896 | ASV379_Reyranella | Autumn | Organic/Integrated | 0.093438 |
| -2.45095 | ASV382_Mucilaginibacter | Autumn | Organic/Integrated | 0.262366 |
| -2.71898 | ASV383_Devosia | Autumn | Organic/Integrated | 0.171428 |
| -4.80394 | ASV384_Massilia | Autumn | Organic/Integrated | 0.281671 |
| 1.070493 | ASV386_Mucilaginibacter | Autumn | Organic/Integrated | 0.088469 |
| 1.26396 | ASV387_Ferruginibacter | Autumn | Organic/Integrated | 0.086674 |
| 2.053557 | ASV388_Dyadobacter | Autumn | Organic/Integrated | 0.313872 |
| -2.57315 | ASV392_Acidipila-Silvibacterium | Autumn | Organic/Integrated | 0.143907 |
| 5.644404 | ASV394_Dyadobacter | Autumn | Organic/Integrated | 0.329466 |
| 1.802316 | ASV395_Nocardioides | Autumn | Organic/Integrated | 0.108137 |
| -3.54418 | ASV40_Pedobacter | Autumn | Organic/Integrated | 3.19976 |
| 2.760711 | ASV400_Pedobacter | Autumn | Organic/Integrated | 0.146437 |
| -3.59986 | ASV403_Sphingomonas | Autumn | Organic/Integrated | 0.255054 |
| 1.658705 | ASV407_Pedomicrobium | Autumn | Organic/Integrated | 0.078972 |
| 2.908859 | ASV41_Flavobacterium | Autumn | Organic/Integrated | 1.796444 |
| -4.05431 | ASV415_Massilia | Autumn | Organic/Integrated | 0.292838 |
| 3.808569 | ASV418_Sphingobacterium | Autumn | Organic/Integrated | 0.820693 |
| -0.78413 | ASV42_Sphingomonas | Autumn | Organic/Integrated | 0.821211 |
| -2.23239 | ASV420_Rhodopseudomonas | Autumn | Organic/Integrated | 0.129336 |
| 2.60801 | ASV423_Pseudoxanthomonas | Autumn | Organic/Integrated | 0.132067 |
| -2.57077 | ASV425_Herbaspirillum | Autumn | Organic/Integrated | 0.351681 |
| 2.357041 | ASV428_Duganella | Autumn | Organic/Integrated | 0.321701 |
| -3.48543 | ASV433_Granulicella | Autumn | Organic/Integrated | 0.180405 |
| 2.594266 | ASV436_Pseudomonas | Autumn | Organic/Integrated | 0.2097 |
| 1.37461 | ASV44_Allorhizobium-Neorhizobium-Pararhizobium-Rhizobium | Autumn | Organic/Integrated | 0.929074 |
| 1.741123 | ASV440_agricultural soil bacterium SC-I-84 | Autumn | Organic/Integrated | 0.074623 |
| -2.18977 | ASV442_Mucilaginibacter | Autumn | Organic/Integrated | 0.210397 |
| 1.222667 | ASV446_Mycobacterium | Autumn | Organic/Integrated | 0.075379 |
| 4.62402 | ASV448_Phyllobacterium | Autumn | Organic/Integrated | 0.080911 |
| -2.78324 | ASV452_Hyphomicrobium | Autumn | Organic/Integrated | 0.113374 |
| 2.295757 | ASV459_Luteibacter | Autumn | Organic/Integrated | 0.043934 |
| -2.65457 | ASV462_Occallatibacter | Autumn | Organic/Integrated | 0.137611 |
| 5.191714 | ASV463_Pseudomonas | Autumn | Organic/Integrated | 0.408251 |
| 2.217715 | ASV467_Nordella | Autumn | Organic/Integrated | 0.09699 |
| 2.738532 | ASV468_Terrimonas | Autumn | Organic/Integrated | 0.126641 |
| -1.65179 | ASV480_Prosthecobacter | Autumn | Organic/Integrated | 0.107597 |
| -1.9405 | ASV482_Unclassified_Chitinophagaceae | Autumn | Organic/Integrated | 0.068327 |
| 4.043222 | ASV486_Devosia | Autumn | Organic/Integrated | 0.173219 |
| -3.93595 | ASV489_Pedobacter | Autumn | Organic/Integrated | 0.330153 |
| -1.72762 | ASV490_Prosthecobacter | Autumn | Organic/Integrated | 0.104233 |
| -5.21166 | ASV496_Unclassified_SC-I-84 | Autumn | Organic/Integrated | 0.120865 |
| 3.379495 | ASV498_Unclassified_Blastocatellaceae | Autumn | Organic/Integrated | 0.082821 |
| -0.90474 | ASV50_Mesorhizobium | Autumn | Organic/Integrated | 0.824212 |
| -2.16431 | ASV510_Jatrophihabitans | Autumn | Organic/Integrated | 0.080466 |
| 4.659696 | ASV511_Paeniglutamicibacter | Autumn | Organic/Integrated | 0.092143 |
| 2.922579 | ASV512_Unclassified_Sphingomonadaceae | Autumn | Organic/Integrated | 0.057899 |
| -1.40995 | ASV517_Arthrobacter | Autumn | Organic/Integrated | 0.057619 |
| -1.81656 | ASV519_Bryobacter | Autumn | Organic/Integrated | 0.080639 |
| 2.672772 | ASV52_Sphingobium | Autumn | Organic/Integrated | 1.193192 |
| 3.779981 | ASV529_Bacillus | Autumn | Organic/Integrated | 0.288786 |
| 2.219433 | ASV53_Sphingomonas | Autumn | Organic/Integrated | 1.002979 |
| -2.2902 | ASV532_Rhodopseudomonas | Autumn | Organic/Integrated | 0.102406 |
| 1.707469 | ASV533_Duganella | Autumn | Organic/Integrated | 0.176273 |
| -3.02683 | ASV536_Mesorhizobium | Autumn | Organic/Integrated | 0.112216 |
| 2.64158 | ASV539_Taibaiella | Autumn | Organic/Integrated | 0.115455 |
| 2.515149 | ASV541_Dyadobacter | Autumn | Organic/Integrated | 0.134795 |
| 1.102997 | ASV55_Massilia | Autumn | Organic/Integrated | 1.17862 |
| -5.27923 | ASV552_Lysinimonas | Autumn | Organic/Integrated | 0.138798 |
| 3.478261 | ASV556_Devosia | Autumn | Organic/Integrated | 0.331027 |
| 3.689271 | ASV561_Pseudochrobactrum | Autumn | Organic/Integrated | 0.038619 |
| 2.948414 | ASV562_Unclassified_MB-A2-108 | Autumn | Organic/Integrated | 0.041212 |
| -5.4117 | ASV565_Taibaiella | Autumn | Organic/Integrated | 0.499437 |
| -2.42395 | ASV567_Unclassified_Armatimonadales | Autumn | Organic/Integrated | 0.185199 |
| -4.67079 | ASV570_Rhodopseudomonas | Autumn | Organic/Integrated | 0.08084 |
| 2.421439 | ASV573_Pedococcus-Phycicoccus | Autumn | Organic/Integrated | 0.051971 |
| -2.55623 | ASV575_Unclassified_Acetobacteraceae | Autumn | Organic/Integrated | 0.105896 |
| -4.05457 | ASV577_Unclassified_Acidobacteriaceae (Subgroup 1) | Autumn | Organic/Integrated | 0.129566 |
| -1.70573 | ASV58_Dyadobacter | Autumn | Organic/Integrated | 1.17786 |
| -4.75418 | ASV584_Unclassified_Gemmatimonadaceae | Autumn | Organic/Integrated | 0.097149 |
| -2.06065 | ASV585_Edaphobacter | Autumn | Organic/Integrated | 0.144056 |
| 1.638726 | ASV59_Sphingopyxis | Autumn | Organic/Integrated | 0.720636 |
| 2.322243 | ASV591_Bosea | Autumn | Organic/Integrated | 0.081336 |
| -1.83388 | ASV595_Bryobacter | Autumn | Organic/Integrated | 0.055942 |
| -1.70945 | ASV596_Sphingomonas | Autumn | Organic/Integrated | 0.093539 |
| -2.91903 | ASV597_Unclassified_Gemmatimonadaceae | Autumn | Organic/Integrated | 0.07354 |
| 3.362662 | ASV598_Tahibacter | Autumn | Organic/Integrated | 0.09519 |
| -2.70015 | ASV599_Dokdonella | Autumn | Organic/Integrated | 0.060499 |
| 1.861422 | ASV60_Allorhizobium-Neorhizobium-Pararhizobium-Rhizobium | Autumn | Organic/Integrated | 0.841231 |
| 3.375901 | ASV607_Sphingomonas | Autumn | Organic/Integrated | 0.074609 |
| -2.45964 | ASV609_Tahibacter | Autumn | Organic/Integrated | 0.047828 |
| 3.967526 | ASV610_Unclassified_Gemmatimonadaceae | Autumn | Organic/Integrated | 0.076889 |
| -4.33495 | ASV627_Gemmatimonas | Autumn | Organic/Integrated | 0.058989 |
| -1.45209 | ASV63_Unclassified_Microbacteriaceae | Autumn | Organic/Integrated | 0.671424 |
| 3.522197 | ASV631_Massilia | Autumn | Organic/Integrated | 0.225401 |
| -1.52534 | ASV632_Lysobacter | Autumn | Organic/Integrated | 0.051707 |
| 5.09604 | ASV640_Rhizobacter | Autumn | Organic/Integrated | 0.097204 |
| -3.95428 | ASV644_Massilia | Autumn | Organic/Integrated | 0.155953 |
| 2.558259 | ASV654_Chitinophaga | Autumn | Organic/Integrated | 0.104014 |
| 2.810458 | ASV656_Pseudolabrys | Autumn | Organic/Integrated | 0.049537 |
| 4.244782 | ASV658_Bosea | Autumn | Organic/Integrated | 0.061466 |
| 1.76259 | ASV659_Gemmatimonas | Autumn | Organic/Integrated | 0.064764 |
| 4.330505 | ASV660_Sphingomonas | Autumn | Organic/Integrated | 0.081075 |
| -2.21972 | ASV661_Unclassified_Micavibrionales | Autumn | Organic/Integrated | 0.073528 |
| -5.2027 | ASV663_Unclassified_Oxalobacteraceae | Autumn | Organic/Integrated | 0.181555 |
| -2.18309 | ASV667_Unclassified_Acetobacteraceae | Autumn | Organic/Integrated | 0.060556 |
| -2.34954 | ASV668_Mizugakiibacter | Autumn | Organic/Integrated | 0.06901 |
| 1.20355 | ASV67_Allorhizobium-Neorhizobium-Pararhizobium-Rhizobium | Autumn | Organic/Integrated | 1.156162 |
| -3.29195 | ASV670_Mucilaginibacter | Autumn | Organic/Integrated | 0.148106 |
| 2.106692 | ASV672_Pelomonas | Autumn | Organic/Integrated | 0.102252 |
| -4.55802 | ASV678_Candidatus Udaeobacter | Autumn | Organic/Integrated | 0.059229 |
| 4.591208 | ASV683_Unclassified_Roseiflexaceae | Autumn | Organic/Integrated | 0.078885 |
| -2.29481 | ASV687_Unclassified_BIrii41 | Autumn | Organic/Integrated | 0.104091 |
| 2.948636 | ASV69_Sphingomonas | Autumn | Organic/Integrated | 0.966474 |
| -4.76076 | ASV690_Mucilaginibacter | Autumn | Organic/Integrated | 0.134213 |
| 3.802969 | ASV696_Ferruginibacter | Autumn | Organic/Integrated | 0.068006 |
| -2.17181 | ASV697_Unclassified_WPS-2 | Autumn | Organic/Integrated | 0.07508 |
| 1.660084 | ASV702_Unclassified_Chitinophagaceae | Autumn | Organic/Integrated | 0.070552 |
| 2.282909 | ASV705_Phenylobacterium | Autumn | Organic/Integrated | 0.069168 |
| 3.711399 | ASV706_Unclassified_Blastocatellaceae | Autumn | Organic/Integrated | 0.047752 |
| 3.076235 | ASV710_Methylotenera | Autumn | Organic/Integrated | 0.15617 |
| 3.29737 | ASV711_Pseudoxanthomonas | Autumn | Organic/Integrated | 0.098608 |
| -1.70337 | ASV717_Unclassified_Caulobacteraceae | Autumn | Organic/Integrated | 0.094763 |
| -2.73405 | ASV721_Nocardia | Autumn | Organic/Integrated | 0.088888 |
| 4.105898 | ASV722_Lysinimonas | Autumn | Organic/Integrated | 0.055902 |
| 1.326033 | ASV723_Unclassified_BIrii41 | Autumn | Organic/Integrated | 0.074891 |
| 4.877683 | ASV730_Rhizobacter | Autumn | Organic/Integrated | 0.124696 |
| 1.7383 | ASV749_hgcI clade | Autumn | Organic/Integrated | 0.055837 |
| -3.12462 | ASV756_Unclassified_Holosporaceae | Autumn | Organic/Integrated | 0.101471 |
| -4.66407 | ASV773_Devosia | Autumn | Organic/Integrated | 0.102609 |
| 1.46152 | ASV776_Luteimonas | Autumn | Organic/Integrated | 0.04422 |
| -2.69843 | ASV777_Acidipila-Silvibacterium | Autumn | Organic/Integrated | 0.108855 |
| -3.15356 | ASV779_Mucilaginibacter | Autumn | Organic/Integrated | 0.068906 |
| 2.850331 | ASV788_Flavobacterium | Autumn | Organic/Integrated | 0.032368 |
| -2.97121 | ASV793_Phenylobacterium | Autumn | Organic/Integrated | 0.086168 |
| -3.16671 | ASV795_Hyphomicrobium | Autumn | Organic/Integrated | 0.070859 |
| -1.96246 | ASV799_JGI 0001001-H03 | Autumn | Organic/Integrated | 0.056158 |
| 2.110898 | ASV80_Massilia | Autumn | Organic/Integrated | 0.959546 |
| -3.05321 | ASV803_Mucilaginibacter | Autumn | Organic/Integrated | 0.087656 |
| 2.598594 | ASV808_Bradyrhizobium | Autumn | Organic/Integrated | 0.041796 |
| 4.70454 | ASV811_Variovorax | Autumn | Organic/Integrated | 0.211651 |
| 4.568313 | ASV818_Unclassified_Roseiflexaceae | Autumn | Organic/Integrated | 0.113984 |
| 4.970083 | ASV819_Rhizobacter | Autumn | Organic/Integrated | 0.099065 |
| -3.49098 | ASV820_Ferruginibacter | Autumn | Organic/Integrated | 0.164943 |
| 3.408576 | ASV822_Rhodoferax | Autumn | Organic/Integrated | 0.066211 |
| -3.73852 | ASV826_Spirillospora | Autumn | Organic/Integrated | 0.043728 |
| -1.63382 | ASV828_Burkholderia-Caballeronia-Paraburkholderia | Autumn | Organic/Integrated | 0.070825 |
| -4.0964 | ASV843_Luteolibacter | Autumn | Organic/Integrated | 0.103682 |
| 4.081467 | ASV849_Unclassified_Xanthobacteraceae | Autumn | Organic/Integrated | 0.073182 |
| 3.670685 | ASV852_Unclassified_Micropepsaceae | Autumn | Organic/Integrated | 0.045047 |
| 3.249123 | ASV853_Unclassified_Azospirillales | Autumn | Organic/Integrated | 0.027444 |
| -4.4462 | ASV859_Bradyrhizobium | Autumn | Organic/Integrated | 0.081378 |
| -3.98609 | ASV868_Unclassified_Blastocatellaceae | Autumn | Organic/Integrated | 0.089243 |
| 3.740213 | ASV876_Unclassified_SC-I-84 | Autumn | Organic/Integrated | 0.051961 |
| -3.82944 | ASV878_Unclassified_Ktedonobacteraceae | Autumn | Organic/Integrated | 0.058477 |
| -2.12181 | ASV88_Chitinophaga | Autumn | Organic/Integrated | 1.166228 |
| 4.578901 | ASV882_Pseudomonas | Autumn | Organic/Integrated | 0.177541 |
| -3.04151 | ASV891_Acidothermus | Autumn | Organic/Integrated | 0.030655 |
| 2.601358 | ASV897_Nordella | Autumn | Organic/Integrated | 0.045354 |
| 3.46798 | ASV898_Reyranella | Autumn | Organic/Integrated | 0.034595 |
| -3.75365 | ASV90_Bacillus | Autumn | Organic/Integrated | 3.096958 |
| -3.38292 | ASV905_Unclassified_Oxalobacteraceae | Autumn | Organic/Integrated | 0.059928 |
| 3.680268 | ASV909_Unclassified_Rhizobiales | Autumn | Organic/Integrated | 0.037991 |
| -3.21254 | ASV923_Bradyrhizobium | Autumn | Organic/Integrated | 0.04486 |
| 4.253611 | ASV924_Citricoccus | Autumn | Organic/Integrated | 0.118498 |
| 2.189834 | ASV926_Unclassified_Microtrichales | Autumn | Organic/Integrated | 0.058015 |
| -3.12475 | ASV927_Unclassified_Intrasporangiaceae | Autumn | Organic/Integrated | 0.066545 |
| 3.868304 | ASV928_Pseudomonas | Autumn | Organic/Integrated | 0.061338 |
| -3.12298 | ASV935_Labrys | Autumn | Organic/Integrated | 0.085652 |
| 2.388781 | ASV94_Allorhizobium-Neorhizobium-Pararhizobium-Rhizobium | Autumn | Organic/Integrated | 0.763982 |
| -3.63142 | ASV940_Reyranella | Autumn | Organic/Integrated | 0.060865 |
| 2.145175 | ASV952_Unclassified_PLTA13 | Autumn | Organic/Integrated | 0.053552 |
| 1.882362 | ASV958_Arenimonas | Autumn | Organic/Integrated | 0.06169 |
| 3.084064 | ASV959_Bdellovibrio | Autumn | Organic/Integrated | 0.057818 |
| -1.48784 | ASV96_Devosia | Autumn | Organic/Integrated | 0.441166 |
| -2.3296 | ASV961_Unclassified_C0119 | Autumn | Organic/Integrated | 0.02431 |
| -2.77344 | ASV965_Roseiarcus | Autumn | Organic/Integrated | 0.043306 |
| 4.706282 | ASV994_Unclassified_Roseiflexaceae | Autumn | Organic/Integrated | 0.097848 |
