# Supplementary material for: Beneficial microbial consortium improves winter rye performance by modulating bacterial communities in the rhizosphere and enhancing plant nutrient acquisition
Source: Front Plant Sci. 2023 Aug 28;14:1232288. doi: 10.3389/fpls.2023.1232288 (PMC10498285; doi:10.3389/fpls.2023.1232288)
Supplement: Supplementary file 8 [file Table_7.docx]

**Supplementary table 7.** ASVs that significantly differed in the rhizosphere of Control (Ctrl) or BMc inoculated Maize plants, under Organic or Conventional farming, in the autumn or the spring sampling. Differential abundance testing was performed via an ANOVA-like test implement in ANCOM-BC2 with Benjamini-Hochberg correction for each of the two sampling periods.

| **ASV** | **Adjusted p-value** | **Sampling** | **Phylum** | **Class** | **Order** | **Family** | **Genus** |
| --- | --- | --- | --- | --- | --- | --- | --- |
| ASV1011 | 8.75E-04 | Autumn | *Gemmatimonadota* | *Gemmatimonadetes* | *Gemmatimonadales* | *Gemmatimonadaceae* | *Gemmatimonas* |
| ASV1017 | 0.039715493 | Autumn | *Proteobacteria* | *Alphaproteobacteria* | *Rhizobiales* | *Rhizobiaceae* | *Aminobacter* |
| ASV103 | 0.007321997 | Autumn | *Proteobacteria* | *Alphaproteobacteria* | *Sphingomonadales* | *Sphingomonadaceae* | *Sphingomonas* |
| ASV1030 | 6.08E-06 | Autumn | *Actinobacteriota* | *Actinobacteria* | *Frankiales* | *Geodermatophilaceae* | *Blastococcus* |
| ASV1034 | 0.020128048 | Autumn | *Chloroflexi* | *Chloroflexia* | *Chloroflexales* | *Roseiflexaceae* | *Unclassified_Roseiflexaceae* |
| ASV1040 | 2.22E-13 | Autumn | *Chloroflexi* | *Ktedonobacteria* | *B12-WMSP1* | *Unclassified* | *Unclassified_B12-WMSP1* |
| ASV106 | 0.012213973 | Autumn | *Proteobacteria* | *Alphaproteobacteria* | *Rhizobiales* | *Stappiaceae* | *Stappia* |
| ASV1063 | 0.001872252 | Autumn | *Proteobacteria* | *Alphaproteobacteria* | *Tistrellales* | *Geminicoccaceae* | *Candidatus Alysiosphaera* |
| ASV1070 | 0.03109952 | Autumn | *Actinobacteriota* | *Actinobacteria* | *Corynebacteriales* | *Mycobacteriaceae* | *Mycobacterium* |
| ASV1082 | 0.040301554 | Autumn | *Proteobacteria* | *Gammaproteobacteria* | *Burkholderiales* | *Comamonadaceae* | *Rhizobacter* |
| ASV1094 | 0.041935316 | Autumn | *Bacteroidota* | *Bacteroidia* | *Sphingobacteriales* | *Sphingobacteriaceae* | *Pedobacter* |
| ASV1102 | 0.033457024 | Autumn | *Proteobacteria* | *Alphaproteobacteria* | *Micropepsales* | *Micropepsaceae* | *Unclassified_Micropepsaceae* |
| ASV1105 | 6.11E-39 | Autumn | *Acidobacteriota* | *Acidobacteriae* | *Acidobacteriales* | *Unclassified* | *Unclassified_Acidobacteriales* |
| ASV1118 | 0.003845376 | Autumn | *Bacteroidota* | *Bacteroidia* | *Flavobacteriales* | *Flavobacteriaceae* | *Flavobacterium* |
| ASV1123 | 6.79E-06 | Autumn | *Proteobacteria* | *Gammaproteobacteria* | *Pseudomonadales* | *Pseudomonadaceae* | *Pseudomonas* |
| ASV1144 | 1.05E-05 | Autumn | *Proteobacteria* | *Gammaproteobacteria* | *Xanthomonadales* | *Xanthomonadaceae* | *Lysobacter* |
| ASV1152 | 0.004951524 | Autumn | *Proteobacteria* | *Alphaproteobacteria* | *Rhizobiales* | *Rhizobiaceae* | *Aureimonas* |
| ASV1159 | 6.52E-04 | Autumn | *Proteobacteria* | *Gammaproteobacteria* | *Xanthomonadales* | *Xanthomonadaceae* | *Stenotrophomonas* |
| ASV1169 | 8.92E-06 | Autumn | *Bacteroidota* | *Bacteroidia* | *Chitinophagales* | *Chitinophagaceae* | *Unclassified_Chitinophagaceae* |
| ASV1180 | 3.99E-07 | Autumn | *Proteobacteria* | *Gammaproteobacteria* | *Xanthomonadales* | *Xanthomonadaceae* | *Lysobacter* |
| ASV1183 | 0.002090261 | Autumn | *Proteobacteria* | *Alphaproteobacteria* | *Rhizobiales* | *Xanthobacteraceae* | *Unclassified_Xanthobacteraceae* |
| ASV1185 | 4.46E-07 | Autumn | *Proteobacteria* | *Gammaproteobacteria* | *Xanthomonadales* | *Xanthomonadaceae* | *Lysobacter* |
| ASV11877 | 0.021275957 | Autumn | *Proteobacteria* | *Gammaproteobacteria* | *Steroidobacterales* | *Steroidobacteraceae* | *Steroidobacter* |
| ASV1201 | 2.17E-07 | Autumn | *Chloroflexi* | *Chloroflexia* | *Chloroflexales* | *Herpetosiphonaceae* | *Herpetosiphon* |
| ASV1206 | 0.001231226 | Autumn | *Bacteroidota* | *Bacteroidia* | *Sphingobacteriales* | *Sphingobacteriaceae* | *Pedobacter* |
| ASV1213 | 4.08E-06 | Autumn | *Proteobacteria* | *Gammaproteobacteria* | *Burkholderiales* | *Oxalobacteraceae* | *Herbaspirillum* |
| ASV1218 | 8.23E-04 | Autumn | *Proteobacteria* | *Gammaproteobacteria* | *Burkholderiales* | *SC-I-84* | *Unclassified_SC-I-84* |
| ASV1231 | 1.85E-05 | Autumn | *Proteobacteria* | *Gammaproteobacteria* | *Burkholderiales* | *Oxalobacteraceae* | *Unclassified_Oxalobacteraceae* |
| ASV1274 | 2.68E-04 | Autumn | *Proteobacteria* | *Alphaproteobacteria* | *Caulobacterales* | *Caulobacteraceae* | *Asticcacaulis* |
| ASV128 | 0.026299585 | Autumn | *Verrucomicrobiota* | *Verrucomicrobiae* | *Verrucomicrobiales* | *Rubritaleaceae* | *Luteolibacter* |
| ASV1286 | 0.012213973 | Autumn | *Proteobacteria* | *Alphaproteobacteria* | *Sphingomonadales* | *Sphingomonadaceae* | *Sphingopyxis* |
| ASV1315 | 0.001042712 | Autumn | *Proteobacteria* | *Gammaproteobacteria* | *Burkholderiales* | *Burkholderiaceae* | *Burkholderia-Caballeronia-Paraburkholderia* |
| ASV1316 | 0.011572328 | Autumn | *Bacteroidota* | *Bacteroidia* | *Chitinophagales* | *Chitinophagaceae* | *Pseudoflavitalea* |
| ASV1319 | 0.013470252 | Autumn | *Actinobacteriota* | *Thermoleophilia* | *Gaiellales* | *Unclassified* | *Unclassified_Gaiellales* |
| ASV1320 | 8.61E-04 | Autumn | *Bacteroidota* | *Bacteroidia* | *Sphingobacteriales* | *Sphingobacteriaceae* | *Mucilaginibacter* |
| ASV133 | 0.034832964 | Autumn | *Proteobacteria* | *Gammaproteobacteria* | *Burkholderiales* | *Oxalobacteraceae* | *Massilia* |
| ASV1344 | 0.004906117 | Autumn | *Proteobacteria* | *Gammaproteobacteria* | *Burkholderiales* | *Oxalobacteraceae* | *Massilia* |
| ASV1360 | 0.002946098 | Autumn | *Bacteroidota* | *Bacteroidia* | *Chitinophagales* | *Chitinophagaceae* | *Taibaiella* |
| ASV1372 | 7.64E-05 | Autumn | *Proteobacteria* | *Gammaproteobacteria* | *Burkholderiales* | *Oxalobacteraceae* | *Unclassified_Oxalobacteraceae* |
| ASV1378 | 0.020132307 | Autumn | *Proteobacteria* | *Alphaproteobacteria* | *Elsterales* | *Unclassified* | *Unclassified_Elsterales* |
| ASV1381 | 1.55E-08 | Autumn | *Chloroflexi* | *Ktedonobacteria* | *C0119* | *Unclassified* | *Unclassified_C0119* |
| ASV1399 | 0.006815767 | Autumn | *Proteobacteria* | *Alphaproteobacteria* | *Rhizobiales* | *Rhizobiales Incertae Sedis* | *Bauldia* |
| ASV1402 | 0.02225271 | Autumn | *Gemmatimonadota* | *Gemmatimonadetes* | *Gemmatimonadales* | *Gemmatimonadaceae* | *Unclassified_Gemmatimonadaceae* |
| ASV1421 | 5.20E-05 | Autumn | *Proteobacteria* | *Alphaproteobacteria* | *Sphingomonadales* | *Sphingomonadaceae* | *Sphingomonas* |
| ASV1434 | 1.21E-04 | Autumn | *Chloroflexi* | *Ktedonobacteria* | *C0119* | *Unclassified* | *Unclassified_C0119* |
| ASV1457 | 1.88E-12 | Autumn | *Bacteroidota* | *Bacteroidia* | *Sphingobacteriales* | *Sphingobacteriaceae* | *Mucilaginibacter* |
| ASV146 | 0.019309615 | Autumn | *Firmicutes* | *Bacilli* | *Paenibacillales* | *Paenibacillaceae* | *Paenibacillus* |
| ASV1465 | 4.67E-26 | Autumn | *Proteobacteria* | *Gammaproteobacteria* | *Burkholderiales* | *Comamonadaceae* | *Unclassified_Comamonadaceae* |
| ASV1466 | 1.93E-04 | Autumn | *Actinobacteriota* | *Actinobacteria* | *Pseudonocardiales* | *Pseudonocardiaceae* | *Pseudonocardia* |
| ASV1476 | 0.022020071 | Autumn | *Proteobacteria* | *Gammaproteobacteria* | *Burkholderiales* | *Oxalobacteraceae* | *Massilia* |
| ASV1478 | 0.022797656 | Autumn | *Bacteroidota* | *Bacteroidia* | *Sphingobacteriales* | *Sphingobacteriaceae* | *Pedobacter* |
| ASV148 | 0.003496689 | Autumn | *Bacteroidota* | *Bacteroidia* | *Chitinophagales* | *Chitinophagaceae* | *Chitinophaga* |
| ASV1497 | 2.52E-04 | Autumn | *Chloroflexi* | *Ktedonobacteria* | *C0119* | *Unclassified* | *Unclassified_C0119* |
| ASV1498 | 0.045471771 | Autumn | *Proteobacteria* | *Alphaproteobacteria* | *Rhizobiales* | *Beijerinckiaceae* | *Microvirga* |
| ASV1519 | 6.08E-10 | Autumn | *Proteobacteria* | *Gammaproteobacteria* | *Xanthomonadales* | *Rhodanobacteraceae* | *Rhodanobacter* |
| ASV1558 | 0.046907305 | Autumn | *Proteobacteria* | *Alphaproteobacteria* | *Acetobacterales* | *Acetobacteraceae* | *Unclassified_Acetobacteraceae* |
| ASV156 | 0.007669718 | Autumn | *Bacteroidota* | *Bacteroidia* | *Cytophagales* | *Spirosomaceae* | *Dyadobacter* |
| ASV1568 | 0.043078276 | Autumn | *Bacteroidota* | *Bacteroidia* | *Sphingobacteriales* | *KD3-93* | *Unclassified_KD3-93* |
| ASV157 | 0.008414735 | Autumn | *Proteobacteria* | *Gammaproteobacteria* | *Burkholderiales* | *Comamonadaceae* | *Limnohabitans* |
| ASV1576 | 0.021666798 | Autumn | *Proteobacteria* | *Gammaproteobacteria* | *Pseudomonadales* | *Pseudomonadaceae* | *Pseudomonas* |
| ASV159 | 0.012345518 | Autumn | *Actinobacteriota* | *Actinobacteria* | *Streptomycetales* | *Streptomycetaceae* | *Kitasatospora* |
| ASV163 | 0.043224676 | Autumn | *Proteobacteria* | *Alphaproteobacteria* | *Rhizobiales* | *Devosiaceae* | *Devosia* |
| ASV1646 | 0.039122191 | Autumn | *Bacteroidota* | *Bacteroidia* | *Cytophagales* | *Microscillaceae* | *Unclassified_Microscillaceae* |
| ASV1659 | 1.58E-06 | Autumn | *Chloroflexi* | *JG30-KF-CM66* | *Unclassified* | *Unclassified* | *Unclassified_JG30-KF-CM66* |
| ASV167 | 0.022656142 | Autumn | *Bacteroidota* | *Bacteroidia* | *Chitinophagales* | *Chitinophagaceae* | *Chitinophaga* |
| ASV1672 | 0.003133973 | Autumn | *Proteobacteria* | *Gammaproteobacteria* | *Burkholderiales* | *Oxalobacteraceae* | *Actimicrobium* |
| ASV169 | 0.005157918 | Autumn | *Proteobacteria* | *Gammaproteobacteria* | *Burkholderiales* | *Comamonadaceae* | *Acidovorax* |
| ASV1698 | 0.005732663 | Autumn | *Proteobacteria* | *Gammaproteobacteria* | *Burkholderiales* | *Oxalobacteraceae* | *Massilia* |
| ASV170 | 5.89E-05 | Autumn | *Bacteroidota* | *Bacteroidia* | *Cytophagales* | *Spirosomaceae* | *Dyadobacter* |
| ASV1702 | 0.012334215 | Autumn | *Proteobacteria* | *Gammaproteobacteria* | *Burkholderiales* | *TRA3-20* | *Unclassified_TRA3-20* |
| ASV1711 | 0.013988924 | Autumn | *Chloroflexi* | *Chloroflexia* | *Thermomicrobiales* | *JG30-KF-CM45* | *Unclassified_JG30-KF-CM45* |
| ASV1723 | 6.47E-05 | Autumn | *Proteobacteria* | *Alphaproteobacteria* | *Rickettsiales* | *Candidatus Jidaibacter* | *Unclassified_Candidatus Jidaibacter* |
| ASV173 | 1.26E-04 | Autumn | *Proteobacteria* | *Gammaproteobacteria* | *Burkholderiales* | *Oxalobacteraceae* | *Duganella* |
| ASV178 | 2.55E-04 | Autumn | *Proteobacteria* | *Alphaproteobacteria* | *Caulobacterales* | *Caulobacteraceae* | *Asticcacaulis* |
| ASV1794 | 0.029130277 | Autumn | *Proteobacteria* | *Gammaproteobacteria* | *Burkholderiales* | *Oxalobacteraceae* | *[Aquaspirillum] arcticum group* |
| ASV1803 | 0.004922254 | Autumn | *Proteobacteria* | *Gammaproteobacteria* | *Burkholderiales* | *Comamonadaceae* | *Giesbergeria* |
| ASV1818 | 5.82E-05 | Autumn | *Bacteroidota* | *Bacteroidia* | *Cytophagales* | *Spirosomaceae* | *Dyadobacter* |
| ASV1851 | 0.039715493 | Autumn | *Proteobacteria* | *Gammaproteobacteria* | *Xanthomonadales* | *Rhodanobacteraceae* | *Unclassified_Rhodanobacteraceae* |
| ASV187 | 1.07E-04 | Autumn | *Acidobacteriota* | *Acidobacteriae* | *Acidobacteriales* | *Unclassified* | *Unclassified_Acidobacteriales* |
| ASV191 | 0.024619661 | Autumn | *Firmicutes* | *Bacilli* | *Paenibacillales* | *Paenibacillaceae* | *Paenibacillus* |
| ASV1915 | 0.007856402 | Autumn | *Proteobacteria* | *Gammaproteobacteria* | *Xanthomonadales* | *Xanthomonadaceae* | *Lysobacter* |
| ASV193 | 0.029151731 | Autumn | *Proteobacteria* | *Gammaproteobacteria* | *Burkholderiales* | *Oxalobacteraceae* | *Duganella* |
| ASV1985 | 2.55E-04 | Autumn | *Actinobacteriota* | *Actinobacteria* | *Micrococcales* | *Microbacteriaceae* | *Microbacterium* |
| ASV205 | 6.89E-04 | Autumn | *Bacteroidota* | *Bacteroidia* | *Sphingobacteriales* | *Sphingobacteriaceae* | *Mucilaginibacter* |
| ASV2097 | 0.004596231 | Autumn | *Nitrospirota* | *Nitrospiria* | *Nitrospirales* | *Nitrospiraceae* | *Nitrospira* |
| ASV211 | 6.62E-04 | Autumn | *Proteobacteria* | *Gammaproteobacteria* | *Burkholderiales* | *Oxalobacteraceae* | *Massilia* |
| ASV215 | 0.012213973 | Autumn | *Gemmatimonadota* | *Gemmatimonadetes* | *Gemmatimonadales* | *Gemmatimonadaceae* | *Gemmatimonas* |
| ASV2173 | 3.57E-07 | Autumn | *Proteobacteria* | *Gammaproteobacteria* | *Burkholderiales* | *Oxalobacteraceae* | *Massilia* |
| ASV218 | 0.020208921 | Autumn | *Bacteroidota* | *Bacteroidia* | *Sphingobacteriales* | *Sphingobacteriaceae* | *Pedobacter* |
| ASV2194 | 0.008414735 | Autumn | *Proteobacteria* | *Gammaproteobacteria* | *Burkholderiales* | *Comamonadaceae* | *Variovorax* |
| ASV220 | 0.001322495 | Autumn | *Bacteroidota* | *Bacteroidia* | *Cytophagales* | *Spirosomaceae* | *Dyadobacter* |
| ASV2204 | 0.006794001 | Autumn | *Actinobacteriota* | *Actinobacteria* | *Streptomycetales* | *Streptomycetaceae* | *Streptomyces* |
| ASV221 | 0.03946884 | Autumn | *Proteobacteria* | *Gammaproteobacteria* | *Burkholderiales* | *Comamonadaceae* | *Rhodoferax* |
| ASV2234 | 0.013988924 | Autumn | *Proteobacteria* | *Gammaproteobacteria* | *Xanthomonadales* | *Rhodanobacteraceae* | *Dokdonella* |
| ASV2256 | 0.010986203 | Autumn | *Bacteroidota* | *Bacteroidia* | *Cytophagales* | *Microscillaceae* | *Unclassified_Microscillaceae* |
| ASV2276 | 0.027466773 | Autumn | *Abditibacteriota* | *Abditibacteria* | *Abditibacteriales* | *Abditibacteriaceae* | *Abditibacterium* |
| ASV228 | 0.022656142 | Autumn | *Proteobacteria* | *Gammaproteobacteria* | *Burkholderiales* | *Oxalobacteraceae* | *Duganella* |
| ASV2328 | 0.022482671 | Autumn | *Myxococcota* | *Myxococcia* | *Myxococcales* | *Myxococcaceae* | *Cystobacter* |
| ASV2331 | 0.049745322 | Autumn | *Actinobacteriota* | *Thermoleophilia* | *Solirubrobacterales* | *Solirubrobacteraceae* | *Conexibacter* |
| ASV235 | 2.24E-04 | Autumn | *Bacteroidota* | *Bacteroidia* | *Sphingobacteriales* | *Sphingobacteriaceae* | *Pedobacter* |
| ASV2380 | 0.002007388 | Autumn | *Proteobacteria* | *Gammaproteobacteria* | *Burkholderiales* | *Oxalobacteraceae* | *Massilia* |
| ASV239 | 0.010162555 | Autumn | *Proteobacteria* | *Gammaproteobacteria* | *Pseudomonadales* | *Pseudomonadaceae* | *Pseudomonas* |
| ASV24 | 0.002322032 | Autumn | *Proteobacteria* | *Alphaproteobacteria* | *Caulobacterales* | *Caulobacteraceae* | *Brevundimonas* |
| ASV243 | 1.08E-05 | Autumn | *Bacteroidota* | *Bacteroidia* | *Chitinophagales* | *Chitinophagaceae* | *Ferruginibacter* |
| ASV2447 | 0.009633038 | Autumn | *Proteobacteria* | *Gammaproteobacteria* | *Burkholderiales* | *Oxalobacteraceae* | *Massilia* |
| ASV250 | 0.004474346 | Autumn | *Proteobacteria* | *Gammaproteobacteria* | *Xanthomonadales* | *Xanthomonadaceae* | *Arenimonas* |
| ASV259 | 0.037433776 | Autumn | *Proteobacteria* | *Alphaproteobacteria* | *Rhizobiales* | *Devosiaceae* | *Devosia* |
| ASV2600 | 1.21E-04 | Autumn | *Actinobacteriota* | *Thermoleophilia* | *Solirubrobacterales* | *Solirubrobacteraceae* | *Conexibacter* |
| ASV265 | 5.50E-05 | Autumn | *Acidobacteriota* | *Acidobacteriae* | *Acidobacteriales* | *Acidobacteriaceae (Subgroup 1)* | *Acidipila-Silvibacterium* |
| ASV2672 | 0.019004817 | Autumn | *Desulfobacterota* | *Desulfuromonadia* | *Bradymonadales* | *Unclassified* | *Unclassified_Bradymonadales* |
| ASV2702 | 0.016888948 | Autumn | *Bacteroidota* | *Bacteroidia* | *Flavobacteriales* | *Flavobacteriaceae* | *Gaetbulibacter* |
| ASV271 | 1.47E-102 | Autumn | *Proteobacteria* | *Alphaproteobacteria* | *Rhizobiales* | *Xanthobacteraceae* | *Bradyrhizobium* |
| ASV2715 | 0.004026814 | Autumn | *Proteobacteria* | *Gammaproteobacteria* | *Burkholderiales* | *Oxalobacteraceae* | *Massilia* |
| ASV2722 | 0.011793046 | Autumn | *Bacteroidota* | *Bacteroidia* | *Sphingobacteriales* | *Sphingobacteriaceae* | *Pedobacter* |
| ASV280 | 3.87E-12 | Autumn | *Actinobacteriota* | *Actinobacteria* | *Micrococcales* | *Microbacteriaceae* | *Frigoribacterium* |
| ASV2801 | 0.023910327 | Autumn | *Actinobacteriota* | *Actinobacteria* | *Propionibacteriales* | *Propionibacteriaceae* | *Friedmanniella* |
| ASV2813 | 0.02225271 | Autumn | *Cyanobacteria* | *Sericytochromatia* | *Unclassified* | *Unclassified* | *Unclassified_Sericytochromatia* |
| ASV2817 | 0.046907305 | Autumn | *Proteobacteria* | *Gammaproteobacteria* | *Burkholderiales* | *Oxalobacteraceae* | *Noviherbaspirillum* |
| ASV283 | 0.042296505 | Autumn | *Bacteroidota* | *Bacteroidia* | *Sphingobacteriales* | *Sphingobacteriaceae* | *Mucilaginibacter* |
| ASV287 | 0.022020071 | Autumn | *Proteobacteria* | *Gammaproteobacteria* | *Burkholderiales* | *Oxalobacteraceae* | *Massilia* |
| ASV29 | 0.021010452 | Autumn | *Bacteroidota* | *Bacteroidia* | *Cytophagales* | *Spirosomaceae* | *Dyadobacter* |
| ASV290 | 0.028812767 | Autumn | *Verrucomicrobiota* | *Verrucomicrobiae* | *Verrucomicrobiales* | *Rubritaleaceae* | *Luteolibacter* |
| ASV291 | 0.03545524 | Autumn | *Proteobacteria* | *Gammaproteobacteria* | *Legionellales* | *Legionellaceae* | *Legionella* |
| ASV298 | 5.80E-04 | Autumn | *Proteobacteria* | *Alphaproteobacteria* | *Sphingomonadales* | *Sphingomonadaceae* | *Sphingomonas* |
| ASV299 | 4.12E-19 | Autumn | *Firmicutes* | *Bacilli* | *Bacillales* | *Bacillaceae* | *Bacillus* |
| ASV30 | 0.005605691 | Autumn | *Bacteroidota* | *Bacteroidia* | *Flavobacteriales* | *Flavobacteriaceae* | *Flavobacterium* |
| ASV300 | 8.14E-04 | Autumn | *Bacteroidota* | *Bacteroidia* | *Chitinophagales* | *Chitinophagaceae* | *Chitinophaga* |
| ASV304 | 0.032427658 | Autumn | *Bacteroidota* | *Bacteroidia* | *Sphingobacteriales* | *Sphingobacteriaceae* | *Pedobacter* |
| ASV3047 | 0.016371764 | Autumn | *Acidobacteriota* | *Acidobacteriae* | *Acidobacteriales* | *Unclassified* | *Unclassified_Acidobacteriales* |
| ASV310 | 0.012213973 | Autumn | *Proteobacteria* | *Alphaproteobacteria* | *Rhizobiales* | *Xanthobacteraceae* | *Afipia* |
| ASV312 | 8.55E-06 | Autumn | *Bacteroidota* | *Bacteroidia* | *Chitinophagales* | *Chitinophagaceae* | *Taibaiella* |
| ASV3146 | 0.002979602 | Autumn | *Proteobacteria* | *Gammaproteobacteria* | *Burkholderiales* | *Burkholderiaceae* | *Burkholderia-Caballeronia-Paraburkholderia* |
| ASV317 | 2.09E-05 | Autumn | *Proteobacteria* | *Alphaproteobacteria* | *Sphingomonadales* | *Sphingomonadaceae* | *Sphingomonas* |
| ASV3182 | 0.017011004 | Autumn | *Proteobacteria* | *Gammaproteobacteria* | *Burkholderiales* | *Burkholderiaceae* | *Burkholderia-Caballeronia-Paraburkholderia* |
| ASV323 | 0.017772807 | Autumn | *Acidobacteriota* | *Blastocatellia* | *Blastocatellales* | *Blastocatellaceae* | *Unclassified_Blastocatellaceae* |
| ASV325 | 1.39E-05 | Autumn | *Proteobacteria* | *Alphaproteobacteria* | *Sphingomonadales* | *Sphingomonadaceae* | *Sphingomonas* |
| ASV327 | 0.034832964 | Autumn | *Proteobacteria* | *Alphaproteobacteria* | *Acetobacterales* | *Acetobacteraceae* | *Acidiphilium* |
| ASV328 | 0.047739814 | Autumn | *Acidobacteriota* | *Acidobacteriae* | *Subgroup 2* | *Unclassified* | *Unclassified_Subgroup 2* |
| ASV329 | 0.02225271 | Autumn | *Proteobacteria* | *Gammaproteobacteria* | *Burkholderiales* | *Burkholderiaceae* | *Burkholderia-Caballeronia-Paraburkholderia* |
| ASV33 | 0.039411263 | Autumn | *Proteobacteria* | *Alphaproteobacteria* | *Rhizobiales* | *Devosiaceae* | *Devosia* |
| ASV333 | 0.003663851 | Autumn | *Proteobacteria* | *Gammaproteobacteria* | *Burkholderiales* | *Comamonadaceae* | *Rhizobacter* |
| ASV335 | 8.25E-05 | Autumn | *Bacteroidota* | *Bacteroidia* | *Sphingobacteriales* | *Sphingobacteriaceae* | *Mucilaginibacter* |
| ASV3414 | 0.008301425 | Autumn | *Proteobacteria* | *Gammaproteobacteria* | *Xanthomonadales* | *Xanthomonadaceae* | *Thermomonas* |
| ASV350 | 7.50E-10 | Autumn | *Proteobacteria* | *Alphaproteobacteria* | *Sphingomonadales* | *Sphingomonadaceae* | *Sphingobium* |
| ASV354 | 0.019572566 | Autumn | *Proteobacteria* | *Alphaproteobacteria* | *Sphingomonadales* | *Sphingomonadaceae* | *Sphingopyxis* |
| ASV359 | 1.80E-05 | Autumn | *Verrucomicrobiota* | *Verrucomicrobiae* | *Chthoniobacterales* | *Chthoniobacteraceae* | *Chthoniobacter* |
| ASV365 | 0.001010785 | Autumn | *Acidobacteriota* | *Acidobacteriae* | *Acidobacteriales* | *Unclassified* | *Unclassified_Acidobacteriales* |
| ASV366 | 0.040301554 | Autumn | *Actinobacteriota* | *Actinobacteria* | *Micrococcales* | *Microbacteriaceae* | *Schumannella* |
| ASV3661 | 0.024185921 | Autumn | *Bacteroidota* | *Bacteroidia* | *Flavobacteriales* | *Flavobacteriaceae* | *Flavobacterium* |
| ASV37 | 0.020875971 | Autumn | *Bacteroidota* | *Bacteroidia* | *Sphingobacteriales* | *Sphingobacteriaceae* | *Mucilaginibacter* |
| ASV3716 | 0.039799043 | Autumn | *Chloroflexi* | *Ktedonobacteria* | *Ktedonobacterales* | *Ktedonobacteraceae* | *Unclassified_Ktedonobacteraceae* |
| ASV3755 | 0.041250531 | Autumn | *Proteobacteria* | *Alphaproteobacteria* | *Sphingomonadales* | *Sphingomonadaceae* | *Sphingopyxis* |
| ASV379 | 0.017478787 | Autumn | *Proteobacteria* | *Alphaproteobacteria* | *Reyranellales* | *Reyranellaceae* | *Reyranella* |
| ASV3811 | 0.004563456 | Autumn | *Proteobacteria* | *Gammaproteobacteria* | *Burkholderiales* | *Comamonadaceae* | *Unclassified_Comamonadaceae* |
| ASV382 | 0.033457024 | Autumn | *Bacteroidota* | *Bacteroidia* | *Sphingobacteriales* | *Sphingobacteriaceae* | *Mucilaginibacter* |
| ASV384 | 4.04E-24 | Autumn | *Proteobacteria* | *Gammaproteobacteria* | *Burkholderiales* | *Oxalobacteraceae* | *Massilia* |
| ASV387 | 4.37E-04 | Autumn | *Bacteroidota* | *Bacteroidia* | *Chitinophagales* | *Chitinophagaceae* | *Ferruginibacter* |
| ASV3903 | 0.048822028 | Autumn | *Bacteroidota* | *Bacteroidia* | *Cytophagales* | *Spirosomaceae* | *Dyadobacter* |
| ASV392 | 0.008781156 | Autumn | *Acidobacteriota* | *Acidobacteriae* | *Acidobacteriales* | *Acidobacteriaceae (Subgroup 1)* | *Acidipila-Silvibacterium* |
| ASV3934 | 0.039799043 | Autumn | *Bacteroidota* | *Bacteroidia* | *Flavobacteriales* | *Flavobacteriaceae* | *Flavobacterium* |
| ASV394 | 0.043078276 | Autumn | *Bacteroidota* | *Bacteroidia* | *Cytophagales* | *Spirosomaceae* | *Dyadobacter* |
| ASV40 | 0.016659714 | Autumn | *Bacteroidota* | *Bacteroidia* | *Sphingobacteriales* | *Sphingobacteriaceae* | *Pedobacter* |
| ASV400 | 0.01469433 | Autumn | *Bacteroidota* | *Bacteroidia* | *Sphingobacteriales* | *Sphingobacteriaceae* | *Pedobacter* |
| ASV403 | 1.99E-06 | Autumn | *Proteobacteria* | *Alphaproteobacteria* | *Sphingomonadales* | *Sphingomonadaceae* | *Sphingomonas* |
| ASV409 | 0.007488159 | Autumn | *Proteobacteria* | *Gammaproteobacteria* | *Burkholderiales* | *Burkholderiaceae* | *Burkholderia-Caballeronia-Paraburkholderia* |
| ASV4095 | 0.034832964 | Autumn | *Bacteroidota* | *Bacteroidia* | *Sphingobacteriales* | *Sphingobacteriaceae* | *Pedobacter* |
| ASV415 | 0.00405231 | Autumn | *Proteobacteria* | *Gammaproteobacteria* | *Burkholderiales* | *Oxalobacteraceae* | *Massilia* |
| ASV4172 | 0.010986203 | Autumn | *Proteobacteria* | *Gammaproteobacteria* | *Xanthomonadales* | *Rhodanobacteraceae* | *Luteibacter* |
| ASV420 | 0.011994869 | Autumn | *Proteobacteria* | *Alphaproteobacteria* | *Rhizobiales* | *Xanthobacteraceae* | *Rhodopseudomonas* |
| ASV423 | 0.006000162 | Autumn | *Proteobacteria* | *Gammaproteobacteria* | *Xanthomonadales* | *Xanthomonadaceae* | *Pseudoxanthomonas* |
| ASV425 | 0.019938051 | Autumn | *Proteobacteria* | *Gammaproteobacteria* | *Burkholderiales* | *Oxalobacteraceae* | *Herbaspirillum* |
| ASV431 | 1.99E-06 | Autumn | *Proteobacteria* | *Alphaproteobacteria* | *Rhizobiales* | *Labraceae* | *Labrys* |
| ASV433 | 1.23E-06 | Autumn | *Acidobacteriota* | *Acidobacteriae* | *Acidobacteriales* | *Acidobacteriaceae (Subgroup 1)* | *Granulicella* |
| ASV436 | 0.034624545 | Autumn | *Proteobacteria* | *Gammaproteobacteria* | *Pseudomonadales* | *Pseudomonadaceae* | *Pseudomonas* |
| ASV437 | 1.75E-04 | Autumn | *Bacteroidota* | *Bacteroidia* | *Sphingobacteriales* | *Sphingobacteriaceae* | *Mucilaginibacter* |
| ASV44 | 0.041448025 | Autumn | *Proteobacteria* | *Alphaproteobacteria* | *Rhizobiales* | *Rhizobiaceae* | *Allorhizobium-Neorhizobium-Pararhizobium-Rhizobium* |
| ASV442 | 0.016888948 | Autumn | *Bacteroidota* | *Bacteroidia* | *Sphingobacteriales* | *Sphingobacteriaceae* | *Mucilaginibacter* |
| ASV448 | 5.15E-04 | Autumn | *Proteobacteria* | *Alphaproteobacteria* | *Rhizobiales* | *Rhizobiaceae* | *Phyllobacterium* |
| ASV451 | 0.001920602 | Autumn | *Verrucomicrobiota* | *Verrucomicrobiae* | *Chthoniobacterales* | *Terrimicrobiaceae* | *Terrimicrobium* |
| ASV452 | 0.021119458 | Autumn | *Proteobacteria* | *Alphaproteobacteria* | *Rhizobiales* | *Hyphomicrobiaceae* | *Hyphomicrobium* |
| ASV453 | 1.93E-08 | Autumn | *Actinobacteriota* | *Actinobacteria* | *Micrococcales* | *Micrococcaceae* | *Pseudarthrobacter* |
| ASV463 | 0.010162555 | Autumn | *Proteobacteria* | *Gammaproteobacteria* | *Pseudomonadales* | *Pseudomonadaceae* | *Pseudomonas* |
| ASV465 | 0.010136328 | Autumn | *Verrucomicrobiota* | *Verrucomicrobiae* | *Verrucomicrobiales* | *Rubritaleaceae* | *Luteolibacter* |
| ASV466 | 1.22E-06 | Autumn | *Proteobacteria* | *Alphaproteobacteria* | *Sphingomonadales* | *Sphingomonadaceae* | *Sphingopyxis* |
| ASV468 | 8.92E-06 | Autumn | *Bacteroidota* | *Bacteroidia* | *Chitinophagales* | *Chitinophagaceae* | *Terrimonas* |
| ASV478 | 1.07E-64 | Autumn | *Bacteroidota* | *Bacteroidia* | *Sphingobacteriales* | *Sphingobacteriaceae* | *Pedobacter* |
| ASV482 | 0.045108288 | Autumn | *Bacteroidota* | *Bacteroidia* | *Chitinophagales* | *Chitinophagaceae* | *Unclassified_Chitinophagaceae* |
| ASV486 | 0.013988924 | Autumn | *Proteobacteria* | *Alphaproteobacteria* | *Rhizobiales* | *Devosiaceae* | *Devosia* |
| ASV489 | 0.003702314 | Autumn | *Bacteroidota* | *Bacteroidia* | *Sphingobacteriales* | *Sphingobacteriaceae* | *Pedobacter* |
| ASV49 | 0.048068051 | Autumn | *Proteobacteria* | *Alphaproteobacteria* | *Rhizobiales* | *Rhizobiaceae* | *Allorhizobium-Neorhizobium-Pararhizobium-Rhizobium* |
| ASV496 | 0.026081634 | Autumn | *Proteobacteria* | *Gammaproteobacteria* | *Burkholderiales* | *SC-I-84* | *Unclassified_SC-I-84* |
| ASV498 | 0.012614695 | Autumn | *Acidobacteriota* | *Blastocatellia* | *Blastocatellales* | *Blastocatellaceae* | *Unclassified_Blastocatellaceae* |
| ASV511 | 3.23E-31 | Autumn | *Actinobacteriota* | *Actinobacteria* | *Micrococcales* | *Micrococcaceae* | *Paeniglutamicibacter* |
| ASV512 | 0.015626904 | Autumn | *Proteobacteria* | *Alphaproteobacteria* | *Sphingomonadales* | *Sphingomonadaceae* | *Unclassified_Sphingomonadaceae* |
| ASV517 | 0.002756624 | Autumn | *Actinobacteriota* | *Actinobacteria* | *Micrococcales* | *Micrococcaceae* | *Arthrobacter* |
| ASV52 | 8.26E-05 | Autumn | *Proteobacteria* | *Alphaproteobacteria* | *Sphingomonadales* | *Sphingomonadaceae* | *Sphingobium* |
| ASV523 | 0.004762999 | Autumn | *Actinobacteriota* | *Actinobacteria* | *Streptomycetales* | *Streptomycetaceae* | *Streptomyces* |
| ASV532 | 0.006794001 | Autumn | *Proteobacteria* | *Alphaproteobacteria* | *Rhizobiales* | *Xanthobacteraceae* | *Rhodopseudomonas* |
| ASV5349 | 0.034832964 | Autumn | *Proteobacteria* | *Gammaproteobacteria* | *Burkholderiales* | *Oxalobacteraceae* | *Massilia* |
| ASV5363 | 0.005732663 | Autumn | *Bacteroidota* | *Bacteroidia* | *Sphingobacteriales* | *Sphingobacteriaceae* | *Mucilaginibacter* |
| ASV539 | 0.007927218 | Autumn | *Bacteroidota* | *Bacteroidia* | *Chitinophagales* | *Chitinophagaceae* | *Taibaiella* |
| ASV541 | 0.025141474 | Autumn | *Bacteroidota* | *Bacteroidia* | *Cytophagales* | *Spirosomaceae* | *Dyadobacter* |
| ASV552 | 4.18E-07 | Autumn | *Actinobacteriota* | *Actinobacteria* | *Micrococcales* | *Microbacteriaceae* | *Lysinimonas* |
| ASV554 | 8.99E-04 | Autumn | *Proteobacteria* | *Gammaproteobacteria* | *Burkholderiales* | *Comamonadaceae* | *Rhizobacter* |
| ASV555 | 0.013871733 | Autumn | *Proteobacteria* | *Alphaproteobacteria* | *Sphingomonadales* | *Sphingomonadaceae* | *Sphingomonas* |
| ASV561 | 9.03E-05 | Autumn | *Proteobacteria* | *Alphaproteobacteria* | *Rhizobiales* | *Rhizobiaceae* | *Pseudochrobactrum* |
| ASV562 | 0.003854971 | Autumn | *Actinobacteriota* | *MB-A2-108* | *Unclassified* | *Unclassified* | *Unclassified_MB-A2-108* |
| ASV565 | 0.048822028 | Autumn | *Bacteroidota* | *Bacteroidia* | *Chitinophagales* | *Chitinophagaceae* | *Taibaiella* |
| ASV567 | 0.02225271 | Autumn | *Armatimonadota* | *Armatimonadia* | *Armatimonadales* | *Unclassified* | *Unclassified_Armatimonadales* |
| ASV570 | 1.63E-07 | Autumn | *Proteobacteria* | *Alphaproteobacteria* | *Rhizobiales* | *Xanthobacteraceae* | *Rhodopseudomonas* |
| ASV571 | 0.001760511 | Autumn | *Verrucomicrobiota* | *Verrucomicrobiae* | *Verrucomicrobiales* | *Rubritaleaceae* | *Luteolibacter* |
| ASV575 | 0.008781156 | Autumn | *Proteobacteria* | *Alphaproteobacteria* | *Acetobacterales* | *Acetobacteraceae* | *Unclassified_Acetobacteraceae* |
| ASV577 | 6.62E-04 | Autumn | *Acidobacteriota* | *Acidobacteriae* | *Acidobacteriales* | *Acidobacteriaceae (Subgroup 1)* | *Unclassified_Acidobacteriaceae (Subgroup 1)* |
| ASV5772 | 0.008747026 | Autumn | *Actinobacteriota* | *Actinobacteria* | *Bifidobacteriales* | *Bifidobacteriaceae* | *Bifidobacterium* |
| ASV584 | 0.028529357 | Autumn | *Gemmatimonadota* | *Gemmatimonadetes* | *Gemmatimonadales* | *Gemmatimonadaceae* | *Unclassified_Gemmatimonadaceae* |
| ASV59 | 0.005267318 | Autumn | *Proteobacteria* | *Alphaproteobacteria* | *Sphingomonadales* | *Sphingomonadaceae* | *Sphingopyxis* |
| ASV591 | 6.87E-04 | Autumn | *Proteobacteria* | *Alphaproteobacteria* | *Rhizobiales* | *Beijerinckiaceae* | *Bosea* |
| ASV593 | 0.002410207 | Autumn | *Actinobacteriota* | *Actinobacteria* | *Streptomycetales* | *Streptomycetaceae* | *Kitasatospora* |
| ASV595 | 0.016888948 | Autumn | *Acidobacteriota* | *Acidobacteriae* | *Bryobacterales* | *Bryobacteraceae* | *Bryobacter* |
| ASV597 | 0.012614695 | Autumn | *Gemmatimonadota* | *Gemmatimonadetes* | *Gemmatimonadales* | *Gemmatimonadaceae* | *Unclassified_Gemmatimonadaceae* |
| ASV598 | 7.83E-11 | Autumn | *Proteobacteria* | *Gammaproteobacteria* | *Xanthomonadales* | *Rhodanobacteraceae* | *Tahibacter* |
| ASV599 | 4.69E-05 | Autumn | *Proteobacteria* | *Gammaproteobacteria* | *Xanthomonadales* | *Rhodanobacteraceae* | *Dokdonella* |
| ASV60 | 9.71E-04 | Autumn | *Proteobacteria* | *Alphaproteobacteria* | *Rhizobiales* | *Rhizobiaceae* | *Allorhizobium-Neorhizobium-Pararhizobium-Rhizobium* |
| ASV605 | 0.036690223 | Autumn | *Bacteroidota* | *Bacteroidia* | *Sphingobacteriales* | *Sphingobacteriaceae* | *Pedobacter* |
| ASV606 | 0.008781156 | Autumn | *Bacteroidota* | *Bacteroidia* | *Flavobacteriales* | *Weeksellaceae* | *Chryseobacterium* |
| ASV607 | 0.022482671 | Autumn | *Proteobacteria* | *Alphaproteobacteria* | *Sphingomonadales* | *Sphingomonadaceae* | *Sphingomonas* |
| ASV609 | 3.69E-04 | Autumn | *Proteobacteria* | *Gammaproteobacteria* | *Xanthomonadales* | *Rhodanobacteraceae* | *Tahibacter* |
| ASV61 | 0.005926663 | Autumn | *Proteobacteria* | *Gammaproteobacteria* | *Xanthomonadales* | *Rhodanobacteraceae* | *Luteibacter* |
| ASV627 | 7.06E-51 | Autumn | *Gemmatimonadota* | *Gemmatimonadetes* | *Gemmatimonadales* | *Gemmatimonadaceae* | *Gemmatimonas* |
| ASV63 | 0.003496689 | Autumn | *Actinobacteriota* | *Actinobacteria* | *Micrococcales* | *Microbacteriaceae* | *Unclassified_Microbacteriaceae* |
| ASV631 | 0.034858096 | Autumn | *Proteobacteria* | *Gammaproteobacteria* | *Burkholderiales* | *Oxalobacteraceae* | *Massilia* |
| ASV640 | 4.71E-109 | Autumn | *Proteobacteria* | *Gammaproteobacteria* | *Burkholderiales* | *Comamonadaceae* | *Rhizobacter* |
| ASV644 | 0.016659714 | Autumn | *Proteobacteria* | *Gammaproteobacteria* | *Burkholderiales* | *Oxalobacteraceae* | *Massilia* |
| ASV656 | 0.002007388 | Autumn | *Proteobacteria* | *Alphaproteobacteria* | *Rhizobiales* | *Xanthobacteraceae* | *Pseudolabrys* |
| ASV657 | 0.02753122 | Autumn | *Actinobacteriota* | *Actinobacteria* | *Propionibacteriales* | *Propionibacteriaceae* | *Microlunatus* |
| ASV658 | 2.55E-04 | Autumn | *Proteobacteria* | *Alphaproteobacteria* | *Rhizobiales* | *Beijerinckiaceae* | *Bosea* |
| ASV661 | 0.017927401 | Autumn | *Proteobacteria* | *Alphaproteobacteria* | *Micavibrionales* | *Unclassified* | *Unclassified_Micavibrionales* |
| ASV663 | 4.92E-05 | Autumn | *Proteobacteria* | *Gammaproteobacteria* | *Burkholderiales* | *Oxalobacteraceae* | *Unclassified_Oxalobacteraceae* |
| ASV666 | 0.006117797 | Autumn | *Bacteroidota* | *Bacteroidia* | *Sphingobacteriales* | *Sphingobacteriaceae* | *Pedobacter* |
| ASV667 | 0.048822028 | Autumn | *Proteobacteria* | *Alphaproteobacteria* | *Acetobacterales* | *Acetobacteraceae* | *Unclassified_Acetobacteraceae* |
| ASV668 | 0.031465125 | Autumn | *Proteobacteria* | *Gammaproteobacteria* | *Xanthomonadales* | *Rhodanobacteraceae* | *Mizugakiibacter* |
| ASV670 | 4.88E-07 | Autumn | *Bacteroidota* | *Bacteroidia* | *Sphingobacteriales* | *Sphingobacteriaceae* | *Mucilaginibacter* |
| ASV672 | 0.025530713 | Autumn | *Proteobacteria* | *Gammaproteobacteria* | *Burkholderiales* | *Comamonadaceae* | *Pelomonas* |
| ASV678 | 8.74E-82 | Autumn | *Verrucomicrobiota* | *Verrucomicrobiae* | *Chthoniobacterales* | *Chthoniobacteraceae* | *Candidatus Udaeobacter* |
| ASV683 | 9.65E-42 | Autumn | *Chloroflexi* | *Chloroflexia* | *Chloroflexales* | *Roseiflexaceae* | *Unclassified_Roseiflexaceae* |
| ASV687 | 0.006774096 | Autumn | *Myxococcota* | *Polyangia* | *Polyangiales* | *BIrii41* | *Unclassified_BIrii41* |
| ASV690 | 0.034858096 | Autumn | *Bacteroidota* | *Bacteroidia* | *Sphingobacteriales* | *Sphingobacteriaceae* | *Mucilaginibacter* |
| ASV702 | 0.000314629 | Autumn | *Bacteroidota* | *Bacteroidia* | *Chitinophagales* | *Chitinophagaceae* | *Unclassified_Chitinophagaceae* |
| ASV705 | 8.75E-05 | Autumn | *Proteobacteria* | *Alphaproteobacteria* | *Caulobacterales* | *Caulobacteraceae* | *Phenylobacterium* |
| ASV710 | 0.011572328 | Autumn | *Proteobacteria* | *Gammaproteobacteria* | *Burkholderiales* | *Methylophilaceae* | *Methylotenera* |
| ASV72 | 2.60E-04 | Autumn | *Firmicutes* | *Bacilli* | *Bacillales* | *Bacillaceae* | *Bacillus* |
| ASV721 | 1.20E-05 | Autumn | *Actinobacteriota* | *Actinobacteria* | *Corynebacteriales* | *Nocardiaceae* | *Nocardia* |
| ASV722 | 4.16E-05 | Autumn | *Actinobacteriota* | *Actinobacteria* | *Micrococcales* | *Microbacteriaceae* | *Lysinimonas* |
| ASV723 | 0.025904918 | Autumn | *Myxococcota* | *Polyangia* | *Polyangiales* | *BIrii41* | *Unclassified_BIrii41* |
| ASV73 | 0.016659714 | Autumn | *Bacteroidota* | *Bacteroidia* | *Sphingobacteriales* | *Sphingobacteriaceae* | *Pedobacter* |
| ASV747 | 9.23E-05 | Autumn | *Proteobacteria* | *Gammaproteobacteria* | *Burkholderiales* | *Comamonadaceae* | *Variovorax* |
| ASV749 | 0.006117797 | Autumn | *Actinobacteriota* | *Actinobacteria* | *Frankiales* | *Sporichthyaceae* | *hgcI clade* |
| ASV756 | 5.44E-08 | Autumn | *Proteobacteria* | *Alphaproteobacteria* | *Holosporales* | *Holosporaceae* | *Unclassified_Holosporaceae* |
| ASV773 | 1.29E-05 | Autumn | *Proteobacteria* | *Alphaproteobacteria* | *Rhizobiales* | *Devosiaceae* | *Devosia* |
| ASV777 | 4.39E-04 | Autumn | *Acidobacteriota* | *Acidobacteriae* | *Acidobacteriales* | *Acidobacteriaceae (Subgroup 1)* | *Acidipila-Silvibacterium* |
| ASV779 | 0.002238084 | Autumn | *Bacteroidota* | *Bacteroidia* | *Sphingobacteriales* | *Sphingobacteriaceae* | *Mucilaginibacter* |
| ASV78 | 0.016693863 | Autumn | *Bacteroidota* | *Bacteroidia* | *Sphingobacteriales* | *Sphingobacteriaceae* | *Pedobacter* |
| ASV783 | 6.03E-66 | Autumn | *Bacteroidota* | *Bacteroidia* | *Sphingobacteriales* | *Sphingobacteriaceae* | *Mucilaginibacter* |
| ASV793 | 0.04512089 | Autumn | *Proteobacteria* | *Alphaproteobacteria* | *Caulobacterales* | *Caulobacteraceae* | *Phenylobacterium* |
| ASV795 | 1.39E-05 | Autumn | *Proteobacteria* | *Alphaproteobacteria* | *Rhizobiales* | *Hyphomicrobiaceae* | *Hyphomicrobium* |
| ASV80 | 3.98E-05 | Autumn | *Proteobacteria* | *Gammaproteobacteria* | *Burkholderiales* | *Oxalobacteraceae* | *Massilia* |
| ASV801 | 0.001586111 | Autumn | *Proteobacteria* | *Gammaproteobacteria* | *Burkholderiales* | *Comamonadaceae* | *Variovorax* |
| ASV803 | 9.08E-06 | Autumn | *Bacteroidota* | *Bacteroidia* | *Sphingobacteriales* | *Sphingobacteriaceae* | *Mucilaginibacter* |
| ASV816 | 0.025393476 | Autumn | *Verrucomicrobiota* | *Verrucomicrobiae* | *Verrucomicrobiales* | *Rubritaleaceae* | *Luteolibacter* |
| ASV818 | 3.21E-07 | Autumn | *Chloroflexi* | *Chloroflexia* | *Chloroflexales* | *Roseiflexaceae* | *Unclassified_Roseiflexaceae* |
| ASV819 | 3.58E-07 | Autumn | *Proteobacteria* | *Gammaproteobacteria* | *Burkholderiales* | *Comamonadaceae* | *Rhizobacter* |
| ASV820 | 0.005175991 | Autumn | *Bacteroidota* | *Bacteroidia* | *Chitinophagales* | *Chitinophagaceae* | *Ferruginibacter* |
| ASV822 | 6.86E-05 | Autumn | *Proteobacteria* | *Gammaproteobacteria* | *Burkholderiales* | *Comamonadaceae* | *Rhodoferax* |
| ASV828 | 1.17E-04 | Autumn | *Proteobacteria* | *Gammaproteobacteria* | *Burkholderiales* | *Burkholderiaceae* | *Burkholderia-Caballeronia-Paraburkholderia* |
| ASV840 | 0.033457024 | Autumn | *Proteobacteria* | *Alphaproteobacteria* | *Rhizobiales* | *Xanthobacteraceae* | *Unclassified_Xanthobacteraceae* |
| ASV843 | 4.46E-07 | Autumn | *Verrucomicrobiota* | *Verrucomicrobiae* | *Verrucomicrobiales* | *Rubritaleaceae* | *Luteolibacter* |
| ASV848 | 0.008781156 | Autumn | *Proteobacteria* | *Gammaproteobacteria* | *Burkholderiales* | *Burkholderiaceae* | *Burkholderia-Caballeronia-Paraburkholderia* |
| ASV849 | 3.81E-05 | Autumn | *Proteobacteria* | *Alphaproteobacteria* | *Rhizobiales* | *Xanthobacteraceae* | *Unclassified_Xanthobacteraceae* |
| ASV850 | 0.008321079 | Autumn | *Proteobacteria* | *Alphaproteobacteria* | *Rhizobiales* | *Xanthobacteraceae* | *Pseudolabrys* |
| ASV853 | 5.64E-07 | Autumn | *Proteobacteria* | *Alphaproteobacteria* | *Azospirillales* | *Unclassified* | *Unclassified_Azospirillales* |
| ASV856 | 0.003936503 | Autumn | *Acidobacteriota* | *Blastocatellia* | *Blastocatellales* | *Blastocatellaceae* | *Unclassified_Blastocatellaceae* |
| ASV859 | 1.75E-04 | Autumn | *Proteobacteria* | *Alphaproteobacteria* | *Rhizobiales* | *Xanthobacteraceae* | *Bradyrhizobium* |
| ASV878 | 2.55E-04 | Autumn | *Chloroflexi* | *Ktedonobacteria* | *Ktedonobacterales* | *Ktedonobacteraceae* | *Unclassified_Ktedonobacteraceae* |
| ASV88 | 0.002322032 | Autumn | *Bacteroidota* | *Bacteroidia* | *Chitinophagales* | *Chitinophagaceae* | *Chitinophaga* |
| ASV892 | 5.65E-04 | Autumn | *Actinobacteriota* | *Actinobacteria* | *Propionibacteriales* | *Nocardioidaceae* | *Nocardioides* |
| ASV897 | 0.034832964 | Autumn | *Proteobacteria* | *Alphaproteobacteria* | *Rhizobiales* | *Rhizobiales Incertae Sedis* | *Nordella* |
| ASV898 | 0.047284559 | Autumn | *Proteobacteria* | *Alphaproteobacteria* | *Reyranellales* | *Reyranellaceae* | *Reyranella* |
| ASV905 | 2.92E-11 | Autumn | *Proteobacteria* | *Gammaproteobacteria* | *Burkholderiales* | *Oxalobacteraceae* | *Unclassified_Oxalobacteraceae* |
| ASV909 | 2.15E-20 | Autumn | *Proteobacteria* | *Alphaproteobacteria* | *Rhizobiales* | *Unclassified* | *Unclassified_Rhizobiales* |
| ASV916 | 0.007321997 | Autumn | *Proteobacteria* | *Alphaproteobacteria* | *Rhodobacterales* | *Rhodobacteraceae* | *Pseudorhodobacter* |
| ASV921 | 4.15E-07 | Autumn | *Bacteroidota* | *Bacteroidia* | *Sphingobacteriales* | *Sphingobacteriaceae* | *Mucilaginibacter* |
| ASV923 | 1.85E-10 | Autumn | *Proteobacteria* | *Alphaproteobacteria* | *Rhizobiales* | *Xanthobacteraceae* | *Bradyrhizobium* |
| ASV924 | 0.020875971 | Autumn | *Actinobacteriota* | *Actinobacteria* | *Micrococcales* | *Micrococcaceae* | *Citricoccus* |
| ASV927 | 0.010821502 | Autumn | *Actinobacteriota* | *Actinobacteria* | *Micrococcales* | *Intrasporangiaceae* | *Unclassified_Intrasporangiaceae* |
| ASV935 | 0.012213973 | Autumn | *Proteobacteria* | *Alphaproteobacteria* | *Rhizobiales* | *Labraceae* | *Labrys* |
| ASV94 | 0.002312219 | Autumn | *Proteobacteria* | *Alphaproteobacteria* | *Rhizobiales* | *Rhizobiaceae* | *Allorhizobium-Neorhizobium-Pararhizobium-Rhizobium* |
| ASV95 | 0.027706115 | Autumn | *Bacteroidota* | *Bacteroidia* | *Sphingobacteriales* | *Sphingobacteriaceae* | *Pedobacter* |
| ASV958 | 9.29E-06 | Autumn | *Proteobacteria* | *Gammaproteobacteria* | *Xanthomonadales* | *Xanthomonadaceae* | *Arenimonas* |
| ASV96 | 8.23E-04 | Autumn | *Proteobacteria* | *Alphaproteobacteria* | *Rhizobiales* | *Devosiaceae* | *Devosia* |
| ASV961 | 9.50E-06 | Autumn | *Chloroflexi* | *Ktedonobacteria* | *C0119* | *Unclassified* | *Unclassified_C0119* |
| ASV965 | 0.013871733 | Autumn | *Proteobacteria* | *Alphaproteobacteria* | *Rhizobiales* | *Beijerinckiaceae* | *Roseiarcus* |
| ASV986 | 0.025827208 | Autumn | *WPS-2* | *Unclassified* | *Unclassified* | *Unclassified* | *Unclassified_WPS-2* |
| ASV9932 | 0.038582173 | Autumn | *Bacteroidota* | *Bacteroidia* | *Chitinophagales* | *Chitinophagaceae* | *Rurimicrobium* |
| ASV994 | 8.26E-05 | Autumn | *Chloroflexi* | *Chloroflexia* | *Chloroflexales* | *Roseiflexaceae* | *Unclassified_Roseiflexaceae* |
| ASV1003 | 0.016207695 | Spring | *Acidobacteriota* | *Blastocatellia* | *Nov-24* | *Unclassified* | *Unclassified_11-24* |
| ASV1029 | 0.006669113 | Spring | *Bacteroidota* | *Bacteroidia* | *Cytophagales* | *Spirosomaceae* | *Larkinella* |
| ASV103 | 2.02E-08 | Spring | *Proteobacteria* | *Alphaproteobacteria* | *Sphingomonadales* | *Sphingomonadaceae* | *Sphingomonas* |
| ASV1040 | 0.045862608 | Spring | *Chloroflexi* | *Ktedonobacteria* | *B12-WMSP1* | *Unclassified* | *Unclassified_B12-WMSP1* |
| ASV106 | 0.003291568 | Spring | *Proteobacteria* | *Alphaproteobacteria* | *Rhizobiales* | *Stappiaceae* | *Stappia* |
| ASV1065 | 1.23E-07 | Spring | *Proteobacteria* | *Alphaproteobacteria* | *Rhizobiales* | *Xanthobacteraceae* | *Rhodoplanes* |
| ASV1069 | 0.00219023 | Spring | *Firmicutes* | *Bacilli* | *Paenibacillales* | *Paenibacillaceae* | *Paenibacillus* |
| ASV1078 | 0.001591449 | Spring | *Acidobacteriota* | *Blastocatellia* | *Blastocatellales* | *Blastocatellaceae* | *JGI 0001001-H03* |
| ASV10845 | 0.024838345 | Spring | *Verrucomicrobiota* | *Verrucomicrobiae* | *Chthoniobacterales* | *Chthoniobacteraceae* | *Chthoniobacter* |
| ASV111 | 0.016078486 | Spring | *Actinobacteriota* | *Actinobacteria* | *Streptomycetales* | *Streptomycetaceae* | *Streptomyces* |
| ASV1117 | 1.18E-05 | Spring | *Acidobacteriota* | *Acidobacteriae* | *Acidobacteriales* | *Unclassified* | *Unclassified_Acidobacteriales* |
| ASV1138 | 0.027972549 | Spring | *Firmicutes* | *Bacilli* | *Paenibacillales* | *Paenibacillaceae* | *Paenibacillus* |
| ASV1143 | 0.004184818 | Spring | *Proteobacteria* | *Alphaproteobacteria* | *Rhizobiales* | *Devosiaceae* | *Devosia* |
| ASV1152 | 0.020277842 | Spring | *Proteobacteria* | *Alphaproteobacteria* | *Rhizobiales* | *Rhizobiaceae* | *Aureimonas* |
| ASV1172 | 5.40E-06 | Spring | *Proteobacteria* | *Gammaproteobacteria* | *Gammaproteobacteria Incertae Sedis* | *Unknown Family* | *Candidatus Ovatusbacter* |
| ASV118 | 0.039327348 | Spring | *Actinobacteriota* | *Actinobacteria* | *Micrococcales* | *Micrococcaceae* | *Paenarthrobacter* |
| ASV1186 | 0.048603533 | Spring | *Actinobacteriota* | *Actinobacteria* | *Corynebacteriales* | *Nocardiaceae* | *Rhodococcus* |
| ASV121 | 2.08E-04 | Spring | *Firmicutes* | *Bacilli* | *Bacillales* | *Bacillaceae* | *Bacillus* |
| ASV1217 | 1.99E-05 | Spring | *Actinobacteriota* | *Actinobacteria* | *Micromonosporales* | *Micromonosporaceae* | *Asanoa* |
| ASV1222 | 0.007487741 | Spring | *Verrucomicrobiota* | *Verrucomicrobiae* | *Chthoniobacterales* | *Chthoniobacteraceae* | *Chthoniobacter* |
| ASV1228 | 1.11E-11 | Spring | *Proteobacteria* | *Alphaproteobacteria* | *Sphingomonadales* | *Sphingomonadaceae* | *Sphingomonas* |
| ASV1236 | 0.016543616 | Spring | *Proteobacteria* | *Gammaproteobacteria* | *Xanthomonadales* | *Rhodanobacteraceae* | *Luteibacter* |
| ASV1244 | 0.044658382 | Spring | *Verrucomicrobiota* | *Verrucomicrobiae* | *Verrucomicrobiales* | *Rubritaleaceae* | *Luteolibacter* |
| ASV1250 | 2.61E-04 | Spring | *Bacteroidota* | *Bacteroidia* | *Chitinophagales* | *Chitinophagaceae* | *Edaphobaculum* |
| ASV1273 | 2.03E-04 | Spring | *Proteobacteria* | *Alphaproteobacteria* | *Rhizobiales* | *Beijerinckiaceae* | *Microvirga* |
| ASV1282 | 0.002295362 | Spring | *Proteobacteria* | *Gammaproteobacteria* | *Burkholderiales* | *SC-I-84* | *Unclassified_SC-I-84* |
| ASV133 | 3.21E-04 | Spring | *Proteobacteria* | *Gammaproteobacteria* | *Burkholderiales* | *Oxalobacteraceae* | *Massilia* |
| ASV1331 | 4.87E-08 | Spring | *Gemmatimonadota* | *Gemmatimonadetes* | *Gemmatimonadales* | *Gemmatimonadaceae* | *Unclassified_Gemmatimonadaceae* |
| ASV1333 | 0.013132535 | Spring | *Proteobacteria* | *Gammaproteobacteria* | *Xanthomonadales* | *Xanthomonadaceae* | *Unclassified_Xanthomonadaceae* |
| ASV1353 | 3.16E-04 | Spring | *Bacteroidota* | *Bacteroidia* | *Chitinophagales* | *Chitinophagaceae* | *Unclassified_Chitinophagaceae* |
| ASV1356 | 0.013500321 | Spring | *Proteobacteria* | *Gammaproteobacteria* | *Burkholderiales* | *SC-I-84* | *Unclassified_SC-I-84* |
| ASV1362 | 0.001591449 | Spring | *Bacteroidota* | *Bacteroidia* | *Chitinophagales* | *Chitinophagaceae* | *Chitinophaga* |
| ASV1370 | 0.041035581 | Spring | *Bacteroidota* | *Bacteroidia* | *Chitinophagales* | *Chitinophagaceae* | *Aurantisolimonas* |
| ASV1373 | 0.042842898 | Spring | *Proteobacteria* | *Gammaproteobacteria* | *Burkholderiales* | *Oxalobacteraceae* | *Oxalobacter* |
| ASV1377 | 0.009560069 | Spring | *Acidobacteriota* | *Blastocatellia* | *Blastocatellales* | *Blastocatellaceae* | *Unclassified_Blastocatellaceae* |
| ASV1390 | 0.002004775 | Spring | *Acidobacteriota* | *Blastocatellia* | *Blastocatellales* | *Blastocatellaceae* | *JGI 0001001-H03* |
| ASV1420 | 0.002048279 | Spring | *Gemmatimonadota* | *Gemmatimonadetes* | *Gemmatimonadales* | *Gemmatimonadaceae* | *Unclassified_Gemmatimonadaceae* |
| ASV145 | 0.012008604 | Spring | *Bacteroidota* | *Bacteroidia* | *Sphingobacteriales* | *Sphingobacteriaceae* | *Pedobacter* |
| ASV1455 | 0.004626993 | Spring | *Proteobacteria* | *Alphaproteobacteria* | *Unclassified* | *Unclassified* | *Unclassified_Alphaproteobacteria* |
| ASV1461 | 0.046888652 | Spring | *Proteobacteria* | *Alphaproteobacteria* | *Sphingomonadales* | *Sphingomonadaceae* | *Erythrobacter* |
| ASV1467 | 0.001292565 | Spring | *Proteobacteria* | *Alphaproteobacteria* | *Caulobacterales* | *Caulobacteraceae* | *Brevundimonas* |
| ASV148 | 1.45E-15 | Spring | *Bacteroidota* | *Bacteroidia* | *Chitinophagales* | *Chitinophagaceae* | *Chitinophaga* |
| ASV1487 | 4.01E-06 | Spring | *Chloroflexi* | *Ktedonobacteria* | *C0119* | *Unclassified* | *Unclassified_C0119* |
| ASV1488 | 0.006689649 | Spring | *Chloroflexi* | *Chloroflexia* | *Thermomicrobiales* | *Thermomicrobiaceae* | *Nitrolancea* |
| ASV150 | 0.039327348 | Spring | *Proteobacteria* | *Gammaproteobacteria* | *Burkholderiales* | *Burkholderiaceae* | *Burkholderia-Caballeronia-Paraburkholderia* |
| ASV1504 | 0.002004775 | Spring | *Bacteroidota* | *Bacteroidia* | *Chitinophagales* | *Chitinophagaceae* | *Edaphobaculum* |
| ASV1519 | 0.014657002 | Spring | *Proteobacteria* | *Gammaproteobacteria* | *Xanthomonadales* | *Rhodanobacteraceae* | *Rhodanobacter* |
| ASV1530 | 0.032897658 | Spring | *Acidobacteriota* | *Acidobacteriae* | *Bryobacterales* | *Bryobacteraceae* | *Bryobacter* |
| ASV1542 | 2.14E-07 | Spring | *Proteobacteria* | *Gammaproteobacteria* | *Diplorickettsiales* | *Diplorickettsiaceae* | *Unclassified_Diplorickettsiaceae* |
| ASV1584 | 6.80E-21 | Spring | *Bacteroidota* | *Bacteroidia* | *Sphingobacteriales* | *Sphingobacteriaceae* | *Pedobacter* |
| ASV1586 | 0.016459296 | Spring | *Proteobacteria* | *Alphaproteobacteria* | *Micropepsales* | *Micropepsaceae* | *Unclassified_Micropepsaceae* |
| ASV1591 | 1.27E-04 | Spring | *Acidobacteriota* | *Blastocatellia* | *Pyrinomonadales* | *Pyrinomonadaceae* | *RB41* |
| ASV1592 | 1.27E-04 | Spring | *Actinobacteriota* | *Actinobacteria* | *Corynebacteriales* | *Mycobacteriaceae* | *Mycobacterium* |
| ASV1612 | 0.002501309 | Spring | *Proteobacteria* | *Gammaproteobacteria* | *Burkholderiales* | *SC-I-84* | *Unclassified_SC-I-84* |
| ASV1648 | 0.01037238 | Spring | *Actinobacteriota* | *Actinobacteria* | *Streptosporangiales* | *Streptosporangiaceae* | *Sphaerisporangium* |
| ASV1664 | 5.97E-69 | Spring | *Proteobacteria* | *Gammaproteobacteria* | *Burkholderiales* | *Oxalobacteraceae* | *Noviherbaspirillum* |
| ASV1694 | 0.006891075 | Spring | *Proteobacteria* | *Gammaproteobacteria* | *Burkholderiales* | *Oxalobacteraceae* | *Herbaspirillum* |
| ASV17 | 0.019704156 | Spring | *Proteobacteria* | *Alphaproteobacteria* | *Sphingomonadales* | *Sphingomonadaceae* | *Sphingomonas* |
| ASV1713 | 0.032917057 | Spring | *Proteobacteria* | *Alphaproteobacteria* | *Rhizobiales* | *Xanthobacteraceae* | *Rhodoplanes* |
| ASV1725 | 2.04E-04 | Spring | *Actinobacteriota* | *Actinobacteria* | *Propionibacteriales* | *Nocardioidaceae* | *Nocardioides* |
| ASV1737 | 0.005787548 | Spring | *Actinobacteriota* | *Actinobacteria* | *Streptosporangiales* | *Thermomonosporaceae* | *Actinocorallia* |
| ASV1743 | 0.003601378 | Spring | *WPS-2* | *Unclassified* | *Unclassified* | *Unclassified* | *Unclassified_WPS-2* |
| ASV1745 | 1.22E-04 | Spring | *Actinobacteriota* | *MB-A2-108* | *Unclassified* | *Unclassified* | *Unclassified_MB-A2-108* |
| ASV1754 | 0.001443251 | Spring | *Actinobacteriota* | *Actinobacteria* | *Frankiales* | *Sporichthyaceae* | *Unclassified_Sporichthyaceae* |
| ASV1759 | 0.00382139 | Spring | *Proteobacteria* | *Alphaproteobacteria* | *Rhizobiales* | *Xanthobacteraceae* | *Rhodoplanes* |
| ASV1809 | 0.018388407 | Spring | *Proteobacteria* | *Alphaproteobacteria* | *Rhizobiales* | *Rhizobiales Incertae Sedis* | *Unclassified_Rhizobiales Incertae Sedis* |
| ASV1856 | 8.39E-08 | Spring | *Nitrospirota* | *Nitrospiria* | *Nitrospirales* | *Nitrospiraceae* | *Nitrospira* |
| ASV1859 | 0.032598923 | Spring | *Firmicutes* | *Bacilli* | *Bacillales* | *Sporolactobacillaceae* | *Sporolactobacillus* |
| ASV1871 | 0.042350637 | Spring | *Actinobacteriota* | *Thermoleophilia* | *Gaiellales* | *Unclassified* | *Unclassified_Gaiellales* |
| ASV1885 | 0.015264274 | Spring | *Firmicutes* | *Thermacetogenia* | *Thermacetogeniales* | *Thermacetogeniaceae* | *Syntrophaceticus* |
| ASV1896 | 6.44E-05 | Spring | *Acidobacteriota* | *Acidobacteriae* | *Solibacterales* | *Solibacteraceae* | *Candidatus Solibacter* |
| ASV191 | 5.05E-05 | Spring | *Firmicutes* | *Bacilli* | *Paenibacillales* | *Paenibacillaceae* | *Paenibacillus* |
| ASV1932 | 0.042842898 | Spring | *Proteobacteria* | *Alphaproteobacteria* | *Elsterales* | *Unclassified* | *Unclassified_Elsterales* |
| ASV196 | 0.044165716 | Spring | *Proteobacteria* | *Alphaproteobacteria* | *SAR11 clade* | *Clade II* | *Unclassified_Clade II* |
| ASV2005 | 0.022520089 | Spring | *Proteobacteria* | *Alphaproteobacteria* | *Rhizobiales* | *Beijerinckiaceae* | *Microvirga* |
| ASV202 | 0.01037238 | Spring | *Proteobacteria* | *Alphaproteobacteria* | *Rhizobiales* | *Rhizobiales Incertae Sedis* | *Unclassified_Rhizobiales Incertae Sedis* |
| ASV2065 | 2.34E-04 | Spring | *Firmicutes* | *Bacilli* | *Paenibacillales* | *Paenibacillaceae* | *Paenibacillus* |
| ASV2099 | 2.83E-05 | Spring | *Acidobacteriota* | *Thermoanaerobaculia* | *Thermoanaerobaculales* | *Thermoanaerobaculaceae* | *Subgroup 10* |
| ASV2112 | 0.036797362 | Spring | *Firmicutes* | *Limnochordia* | *Hydrogenispora* | *Unclassified* | *Unclassified_Hydrogenispora* |
| ASV2129 | 0.021433745 | Spring | *Proteobacteria* | *Alphaproteobacteria* | *Caulobacterales* | *Caulobacteraceae* | *Brevundimonas* |
| ASV2130 | 2.57E-14 | Spring | *Actinobacteriota* | *Thermoleophilia* | *Gaiellales* | *Unclassified* | *Unclassified_Gaiellales* |
| ASV2131 | 0.010405438 | Spring | *Bacteroidota* | *Bacteroidia* | *Sphingobacteriales* | *Sphingobacteriaceae* | *Arcticibacter* |
| ASV2151 | 0.003884039 | Spring | *Proteobacteria* | *Gammaproteobacteria* | *Diplorickettsiales* | *Diplorickettsiaceae* | *Unclassified_Diplorickettsiaceae* |
| ASV2164 | 0.01037238 | Spring | *Acidobacteriota* | *Blastocatellia* | *Pyrinomonadales* | *Pyrinomonadaceae* | *RB41* |
| ASV2243 | 0.027108326 | Spring | *Nitrospirota* | *Nitrospiria* | *Nitrospirales* | *Nitrospiraceae* | *Nitrospira* |
| ASV2261 | 3.83E-17 | Spring | *Proteobacteria* | *Alphaproteobacteria* | *Reyranellales* | *Reyranellaceae* | *Reyranella* |
| ASV2302 | 0.013053392 | Spring | *Proteobacteria* | *Alphaproteobacteria* | *Sphingomonadales* | *Sphingomonadaceae* | *Sphingoaurantiacus* |
| ASV2312 | 0.002475884 | Spring | *Gemmatimonadota* | *Gemmatimonadetes* | *Gemmatimonadales* | *Gemmatimonadaceae* | *Gemmatimonas* |
| ASV2361 | 0.042842898 | Spring | *Proteobacteria* | *Alphaproteobacteria* | *Caulobacterales* | *Caulobacteraceae* | *Brevundimonas* |
| ASV2460 | 2.06E-10 | Spring | *Patescibacteria* | *Saccharimonadia* | *Saccharimonadales* | *Unclassified* | *Unclassified_Saccharimonadales* |
| ASV2470 | 5.12E-16 | Spring | *Chloroflexi* | *Chloroflexia* | *Thermomicrobiales* | *JG30-KF-CM45* | *Unclassified_JG30-KF-CM45* |
| ASV249 | 0.046130805 | Spring | *Bacteroidota* | *Bacteroidia* | *Cytophagales* | *Spirosomaceae* | *Dyadobacter* |
| ASV2493 | 0.011605985 | Spring | *Bacteroidota* | *Bacteroidia* | *Chitinophagales* | *Chitinophagaceae* | *Unclassified_Chitinophagaceae* |
| ASV2564 | 0.004691385 | Spring | *Proteobacteria* | *Gammaproteobacteria* | *Legionellales* | *Legionellaceae* | *Legionella* |
| ASV2618 | 0.005876933 | Spring | *Proteobacteria* | *Gammaproteobacteria* | *Gammaproteobacteria Incertae Sedis* | *Unknown Family* | *Acidibacter* |
| ASV263 | 1.86E-04 | Spring | *Gemmatimonadota* | *Gemmatimonadetes* | *Gemmatimonadales* | *Gemmatimonadaceae* | *Unclassified_Gemmatimonadaceae* |
| ASV265 | 0.011213079 | Spring | *Acidobacteriota* | *Acidobacteriae* | *Acidobacteriales* | *Acidobacteriaceae (Subgroup 1)* | *Acidipila-Silvibacterium* |
| ASV2650 | 0.01037238 | Spring | *Proteobacteria* | *Gammaproteobacteria* | *Burkholderiales* | *Comamonadaceae* | *Pelomonas* |
| ASV2664 | 0.002004775 | Spring | *Verrucomicrobiota* | *Verrucomicrobiae* | *Chthoniobacterales* | *Terrimicrobiaceae* | *Terrimicrobium* |
| ASV271 | 0.00219023 | Spring | *Proteobacteria* | *Alphaproteobacteria* | *Rhizobiales* | *Xanthobacteraceae* | *Bradyrhizobium* |
| ASV2746 | 0.042686264 | Spring | *Actinobacteriota* | *Actinobacteria* | *Propionibacteriales* | *Nocardioidaceae* | *Nocardioides* |
| ASV2753 | 0.016997062 | Spring | *Acidobacteriota* | *Thermoanaerobaculia* | *Thermoanaerobaculales* | *Thermoanaerobaculaceae* | *Subgroup 10* |
| ASV276 | 0.00141441 | Spring | *Proteobacteria* | *Gammaproteobacteria* | *Burkholderiales* | *Burkholderiaceae* | *Burkholderia-Caballeronia-Paraburkholderia* |
| ASV2780 | 2.31E-24 | Spring | *Bacteroidota* | *Bacteroidia* | *Cytophagales* | *Spirosomaceae* | *Fibrisoma* |
| ASV2824 | 0.0224981 | Spring | *Chloroflexi* | *KD4-96* | *Unclassified* | *Unclassified* | *Unclassified_KD4-96* |
| ASV283 | 0.007487741 | Spring | *Bacteroidota* | *Bacteroidia* | *Sphingobacteriales* | *Sphingobacteriaceae* | *Mucilaginibacter* |
| ASV2858 | 0.009131547 | Spring | *Actinobacteriota* | *Thermoleophilia* | *Solirubrobacterales* | *67-14* | *Unclassified_67-14* |
| ASV2896 | 0.014802627 | Spring | *Actinobacteriota* | *Actinobacteria* | *Micromonosporales* | *Micromonosporaceae* | *Micromonospora* |
| ASV2945 | 0.041035581 | Spring | *Proteobacteria* | *Gammaproteobacteria* | *Xanthomonadales* | *Rhodanobacteraceae* | *Mizugakiibacter* |
| ASV2957 | 0.024204249 | Spring | *Chloroflexi* | *Chloroflexia* | *Thermomicrobiales* | *JG30-KF-CM45* | *Unclassified_JG30-KF-CM45* |
| ASV298 | 0.016783744 | Spring | *Proteobacteria* | *Alphaproteobacteria* | *Sphingomonadales* | *Sphingomonadaceae* | *Sphingomonas* |
| ASV2995 | 0.021889673 | Spring | *Proteobacteria* | *Alphaproteobacteria* | *Sphingomonadales* | *Sphingomonadaceae* | *Sphingomonas* |
| ASV303 | 0.016543616 | Spring | *Actinobacteriota* | *Actinobacteria* | *Frankiales* | *Nakamurellaceae* | *Nakamurella* |
| ASV306 | 0.010394208 | Spring | *Proteobacteria* | *Gammaproteobacteria* | *Xanthomonadales* | *Xanthomonadaceae* | *Lysobacter* |
| ASV308 | 0.010358552 | Spring | *Proteobacteria* | *Gammaproteobacteria* | *Burkholderiales* | *SC-I-84* | *Unclassified_SC-I-84* |
| ASV309 | 0.036640676 | Spring | *Actinobacteriota* | *Acidimicrobiia* | *Microtrichales* | *Ilumatobacteraceae* | *CL500-29 marine group* |
| ASV31 | 6.90E-05 | Spring | *Bacteroidota* | *Bacteroidia* | *Sphingobacteriales* | *Sphingobacteriaceae* | *Pedobacter* |
| ASV321 | 0.025406482 | Spring | *Proteobacteria* | *Alphaproteobacteria* | *Rhizobiales* | *Xanthobacteraceae* | *Unclassified_Xanthobacteraceae* |
| ASV323 | 1.27E-05 | Spring | *Acidobacteriota* | *Blastocatellia* | *Blastocatellales* | *Blastocatellaceae* | *Unclassified_Blastocatellaceae* |
| ASV3265 | 0.032917057 | Spring | *Actinobacteriota* | *Actinobacteria* | *Propionibacteriales* | *Nocardioidaceae* | *Nocardioides* |
| ASV3270 | 0.020985741 | Spring | *Chloroflexi* | *Chloroflexia* | *Thermomicrobiales* | *JG30-KF-CM45* | *Unclassified_JG30-KF-CM45* |
| ASV331 | 8.56E-04 | Spring | *Actinobacteriota* | *Actinobacteria* | *Micrococcales* | *Microbacteriaceae* | *Microbacterium* |
| ASV339 | 1.46E-06 | Spring | *Actinobacteriota* | *Actinobacteria* | *Micrococcales* | *Micrococcaceae* | *Pseudarthrobacter* |
| ASV35 | 0.044658382 | Spring | *Proteobacteria* | *Alphaproteobacteria* | *Sphingomonadales* | *Sphingomonadaceae* | *Novosphingobium* |
| ASV3675 | 0.036797362 | Spring | *Proteobacteria* | *Gammaproteobacteria* | *Burkholderiales* | *Comamonadaceae* | *Tibeticola* |
| ASV375 | 0.013454137 | Spring | *Actinobacteriota* | *Actinobacteria* | *Glycomycetales* | *Glycomycetaceae* | *Glycomyces* |
| ASV377 | 3.24E-05 | Spring | *Actinobacteriota* | *Actinobacteria* | *Streptosporangiales* | *Thermomonosporaceae* | *Actinoallomurus* |
| ASV380 | 0.039486343 | Spring | *Verrucomicrobiota* | *Verrucomicrobiae* | *Verrucomicrobiales* | *Verrucomicrobiaceae* | *Unclassified_Verrucomicrobiaceae* |
| ASV386 | 7.83E-04 | Spring | *Bacteroidota* | *Bacteroidia* | *Sphingobacteriales* | *Sphingobacteriaceae* | *Mucilaginibacter* |
| ASV3861 | 0.009799375 | Spring | *Proteobacteria* | *Gammaproteobacteria* | *Burkholderiales* | *SC-I-84* | *Unclassified_SC-I-84* |
| ASV392 | 0.022069704 | Spring | *Acidobacteriota* | *Acidobacteriae* | *Acidobacteriales* | *Acidobacteriaceae (Subgroup 1)* | *Acidipila-Silvibacterium* |
| ASV3941 | 0.00289751 | Spring | *Bacteroidota* | *Bacteroidia* | *Cytophagales* | *Spirosomaceae* | *Spirosoma* |
| ASV395 | 0.00185429 | Spring | *Actinobacteriota* | *Actinobacteria* | *Propionibacteriales* | *Nocardioidaceae* | *Nocardioides* |
| ASV396 | 3.31E-07 | Spring | *Proteobacteria* | *Alphaproteobacteria* | *Sphingomonadales* | *Sphingomonadaceae* | *Sphingomonas* |
| ASV400 | 0.020277842 | Spring | *Bacteroidota* | *Bacteroidia* | *Sphingobacteriales* | *Sphingobacteriaceae* | *Pedobacter* |
| ASV4048 | 0.008126275 | Spring | *Proteobacteria* | *Gammaproteobacteria* | *Burkholderiales* | *Burkholderiaceae* | *Burkholderia-Caballeronia-Paraburkholderia* |
| ASV407 | 4.30E-05 | Spring | *Proteobacteria* | *Alphaproteobacteria* | *Rhizobiales* | *Hyphomicrobiaceae* | *Pedomicrobium* |
| ASV4138 | 0.037979723 | Spring | *Proteobacteria* | *Gammaproteobacteria* | *Burkholderiales* | *Oxalobacteraceae* | *Herminiimonas* |
| ASV415 | 0.010244693 | Spring | *Proteobacteria* | *Gammaproteobacteria* | *Burkholderiales* | *Oxalobacteraceae* | *Massilia* |
| ASV420 | 0.002525249 | Spring | *Proteobacteria* | *Alphaproteobacteria* | *Rhizobiales* | *Xanthobacteraceae* | *Rhodopseudomonas* |
| ASV440 | 0.011940738 | Spring | *Proteobacteria* | *Gammaproteobacteria* | *Burkholderiales* | *SC-I-84* | *agricultural soil bacterium SC-I-84* |
| ASV446 | 0.003875997 | Spring | *Actinobacteriota* | *Actinobacteria* | *Corynebacteriales* | *Mycobacteriaceae* | *Mycobacterium* |
| ASV448 | 4.18E-07 | Spring | *Proteobacteria* | *Alphaproteobacteria* | *Rhizobiales* | *Rhizobiaceae* | *Phyllobacterium* |
| ASV453 | 0.036989924 | Spring | *Actinobacteriota* | *Actinobacteria* | *Micrococcales* | *Micrococcaceae* | *Pseudarthrobacter* |
| ASV454 | 0.043110649 | Spring | *Proteobacteria* | *Alphaproteobacteria* | *Rhizobiales* | *Methyloligellaceae* | *Unclassified_Methyloligellaceae* |
| ASV464 | 0.043836459 | Spring | *Proteobacteria* | *Gammaproteobacteria* | *Burkholderiales* | *Oxalobacteraceae* | *Massilia* |
| ASV465 | 0.029054579 | Spring | *Verrucomicrobiota* | *Verrucomicrobiae* | *Verrucomicrobiales* | *Rubritaleaceae* | *Luteolibacter* |
| ASV466 | 0.042842898 | Spring | *Proteobacteria* | *Alphaproteobacteria* | *Sphingomonadales* | *Sphingomonadaceae* | *Sphingopyxis* |
| ASV474 | 0.046888652 | Spring | *Firmicutes* | *Bacilli* | *Paenibacillales* | *Paenibacillaceae* | *Paenibacillus* |
| ASV48 | 0.01313565 | Spring | *Proteobacteria* | *Gammaproteobacteria* | *Xanthomonadales* | *Rhodanobacteraceae* | *Luteibacter* |
| ASV482 | 6.44E-05 | Spring | *Bacteroidota* | *Bacteroidia* | *Chitinophagales* | *Chitinophagaceae* | *Unclassified_Chitinophagaceae* |
| ASV488 | 4.76E-33 | Spring | *Proteobacteria* | *Gammaproteobacteria* | *Xanthomonadales* | *Rhodanobacteraceae* | *Luteibacter* |
| ASV491 | 1.60E-04 | Spring | *Bacteroidota* | *Bacteroidia* | *Chitinophagales* | *Chitinophagaceae* | *Ferruginibacter* |
| ASV4964 | 0.046888652 | Spring | *Proteobacteria* | *Gammaproteobacteria* | *Burkholderiales* | *Burkholderiaceae* | *Lautropia* |
| ASV498 | 4.44E-06 | Spring | *Acidobacteriota* | *Blastocatellia* | *Blastocatellales* | *Blastocatellaceae* | *Unclassified_Blastocatellaceae* |
| ASV504 | 0.046888652 | Spring | *Actinobacteriota* | *Actinobacteria* | *Propionibacteriales* | *Nocardioidaceae* | *Nocardioides* |
| ASV511 | 1.95E-04 | Spring | *Actinobacteriota* | *Actinobacteria* | *Micrococcales* | *Micrococcaceae* | *Paeniglutamicibacter* |
| ASV514 | 0.002195818 | Spring | *Actinobacteriota* | *Actinobacteria* | *Micrococcales* | *Microbacteriaceae* | *Leifsonia* |
| ASV518 | 1.51E-06 | Spring | *Actinobacteriota* | *Actinobacteria* | *Micromonosporales* | *Micromonosporaceae* | *Catellatospora* |
| ASV521 | 0.012014266 | Spring | *Firmicutes* | *Clostridia* | *Clostridiales* | *Clostridiaceae* | *Clostridium sensu stricto 13* |
| ASV522 | 0.023674547 | Spring | *Chloroflexi* | *KD4-96* | *Unclassified* | *Unclassified* | *Unclassified_KD4-96* |
| ASV523 | 1.40E-09 | Spring | *Actinobacteriota* | *Actinobacteria* | *Streptomycetales* | *Streptomycetaceae* | *Streptomyces* |
| ASV526 | 2.93E-06 | Spring | *Proteobacteria* | *Alphaproteobacteria* | *Sphingomonadales* | *Sphingomonadaceae* | *Sphingomonas* |
| ASV532 | 0.024204249 | Spring | *Proteobacteria* | *Alphaproteobacteria* | *Rhizobiales* | *Xanthobacteraceae* | *Rhodopseudomonas* |
| ASV535 | 0.021889673 | Spring | *Actinobacteriota* | *Thermoleophilia* | *Solirubrobacterales* | *Solirubrobacteraceae* | *Solirubrobacter* |
| ASV536 | 0.036797362 | Spring | *Proteobacteria* | *Alphaproteobacteria* | *Rhizobiales* | *Rhizobiaceae* | *Mesorhizobium* |
| ASV554 | 5.51E-10 | Spring | *Proteobacteria* | *Gammaproteobacteria* | *Burkholderiales* | *Comamonadaceae* | *Rhizobacter* |
| ASV558 | 0.007884829 | Spring | *Actinobacteriota* | *Acidimicrobiia* | *Microtrichales* | *Ilumatobacteraceae* | *CL500-29 marine group* |
| ASV582 | 6.50E-08 | Spring | *Actinobacteriota* | *Actinobacteria* | *Corynebacteriales* | *Mycobacteriaceae* | *Mycobacterium* |
| ASV588 | 6.87E-08 | Spring | *Proteobacteria* | *Alphaproteobacteria* | *Rhizobiales* | *Hyphomicrobiaceae* | *Hyphomicrobium* |
| ASV594 | 0.01735029 | Spring | *Proteobacteria* | *Gammaproteobacteria* | *Xanthomonadales* | *Rhodanobacteraceae* | *Dyella* |
| ASV601 | 0.013454137 | Spring | *Chloroflexi* | *Gitt-GS-136* | *Unclassified* | *Unclassified* | *Unclassified_Gitt-GS-136* |
| ASV607 | 0.032598923 | Spring | *Proteobacteria* | *Alphaproteobacteria* | *Sphingomonadales* | *Sphingomonadaceae* | *Sphingomonas* |
| ASV609 | 1.51E-10 | Spring | *Proteobacteria* | *Gammaproteobacteria* | *Xanthomonadales* | *Rhodanobacteraceae* | *Tahibacter* |
| ASV61 | 0.036797362 | Spring | *Proteobacteria* | *Gammaproteobacteria* | *Xanthomonadales* | *Rhodanobacteraceae* | *Luteibacter* |
| ASV613 | 1.95E-07 | Spring | *Actinobacteriota* | *Actinobacteria* | *Propionibacteriales* | *Nocardioidaceae* | *Marmoricola* |
| ASV623 | 1.99E-05 | Spring | *Proteobacteria* | *Gammaproteobacteria* | *Xanthomonadales* | *Rhodanobacteraceae* | *Dokdonella* |
| ASV63 | 0.039327348 | Spring | *Actinobacteriota* | *Actinobacteria* | *Micrococcales* | *Microbacteriaceae* | *Unclassified_Microbacteriaceae* |
| ASV637 | 1.20E-06 | Spring | *Proteobacteria* | *Gammaproteobacteria* | *Burkholderiales* | *SC-I-84* | *Unclassified_SC-I-84* |
| ASV639 | 0.004718981 | Spring | *Bacteroidota* | *Bacteroidia* | *Chitinophagales* | *Chitinophagaceae* | *Flavisolibacter* |
| ASV654 | 1.52E-04 | Spring | *Bacteroidota* | *Bacteroidia* | *Chitinophagales* | *Chitinophagaceae* | *Chitinophaga* |
| ASV656 | 2.03E-04 | Spring | *Proteobacteria* | *Alphaproteobacteria* | *Rhizobiales* | *Xanthobacteraceae* | *Pseudolabrys* |
| ASV661 | 0.026930529 | Spring | *Proteobacteria* | *Alphaproteobacteria* | *Micavibrionales* | *Unclassified* | *Unclassified_Micavibrionales* |
| ASV668 | 2.70E-04 | Spring | *Proteobacteria* | *Gammaproteobacteria* | *Xanthomonadales* | *Rhodanobacteraceae* | *Mizugakiibacter* |
| ASV675 | 1.31E-04 | Spring | *Chloroflexi* | *KD4-96* | *Unclassified* | *Unclassified* | *Unclassified_KD4-96* |
| ASV677 | 0.024172288 | Spring | *Bacteroidota* | *Bacteroidia* | *Chitinophagales* | *Chitinophagaceae* | *Unclassified_Chitinophagaceae* |
| ASV68 | 0.024272518 | Spring | *Proteobacteria* | *Alphaproteobacteria* | *Rhizobiales* | *Devosiaceae* | *Devosia* |
| ASV686 | 0.001025133 | Spring | *Verrucomicrobiota* | *Verrucomicrobiae* | *Verrucomicrobiales* | *Rubritaleaceae* | *Luteolibacter* |
| ASV691 | 6.20E-09 | Spring | *Proteobacteria* | *Gammaproteobacteria* | *Xanthomonadales* | *Rhodanobacteraceae* | *Rhodanobacter* |
| ASV694 | 6.70E-05 | Spring | *Bacteroidota* | *Bacteroidia* | *Chitinophagales* | *Chitinophagaceae* | *Unclassified_Chitinophagaceae* |
| ASV696 | 1.76E-11 | Spring | *Bacteroidota* | *Bacteroidia* | *Chitinophagales* | *Chitinophagaceae* | *Ferruginibacter* |
| ASV706 | 0.004830297 | Spring | *Acidobacteriota* | *Blastocatellia* | *Blastocatellales* | *Blastocatellaceae* | *Unclassified_Blastocatellaceae* |
| ASV709 | 1.70E-45 | Spring | *Acidobacteriota* | *Blastocatellia* | *Blastocatellales* | *Blastocatellaceae* | *Unclassified_Blastocatellaceae* |
| ASV715 | 1.64E-04 | Spring | *Proteobacteria* | *Gammaproteobacteria* | *Xanthomonadales* | *Xanthomonadaceae* | *Pseudoxanthomonas* |
| ASV72 | 0.022069704 | Spring | *Firmicutes* | *Bacilli* | *Bacillales* | *Bacillaceae* | *Bacillus* |
| ASV721 | 0.016995574 | Spring | *Actinobacteriota* | *Actinobacteria* | *Corynebacteriales* | *Nocardiaceae* | *Nocardia* |
| ASV744 | 0.001257547 | Spring | *Proteobacteria* | *Alphaproteobacteria* | *Rhizobiales* | *Rhizobiaceae* | *Allorhizobium-Neorhizobium-Pararhizobium-Rhizobium* |
| ASV757 | 0.005543702 | Spring | *Proteobacteria* | *Gammaproteobacteria* | *Burkholderiales* | *Oxalobacteraceae* | *Massilia* |
| ASV758 | 0.015090662 | Spring | *Proteobacteria* | *Gammaproteobacteria* | *Pseudomonadales* | *Pseudomonadaceae* | *Pseudomonas* |
| ASV760 | 0.001691973 | Spring | *Proteobacteria* | *Alphaproteobacteria* | *Rhizobiales* | *Rhizobiales Incertae Sedis* | *Unclassified_Rhizobiales Incertae Sedis* |
| ASV767 | 2.49E-13 | Spring | *Proteobacteria* | *Gammaproteobacteria* | *Burkholderiales* | *Oxalobacteraceae* | *Actimicrobium* |
| ASV771 | 8.92E-04 | Spring | *Acidobacteriota* | *Blastocatellia* | *Pyrinomonadales* | *Pyrinomonadaceae* | *RB41* |
| ASV775 | 0.014657002 | Spring | *Proteobacteria* | *Gammaproteobacteria* | *Burkholderiales* | *Comamonadaceae* | *Rhodoferax* |
| ASV789 | 0.00601157 | Spring | *Proteobacteria* | *Alphaproteobacteria* | *Caulobacterales* | *Caulobacteraceae* | *Unclassified_Caulobacteraceae* |
| ASV806 | 8.42E-10 | Spring | *Proteobacteria* | *Alphaproteobacteria* | *Rhizobiales* | *Xanthobacteraceae* | *Bradyrhizobium* |
| ASV815 | 2.06E-10 | Spring | *Actinobacteriota* | *Thermoleophilia* | *Gaiellales* | *Gaiellaceae* | *Gaiella* |
| ASV822 | 0.049170061 | Spring | *Proteobacteria* | *Gammaproteobacteria* | *Burkholderiales* | *Comamonadaceae* | *Rhodoferax* |
| ASV823 | 1.20E-08 | Spring | *Actinobacteriota* | *Actinobacteria* | *Streptomycetales* | *Streptomycetaceae* | *Streptomyces* |
| ASV828 | 1.92E-07 | Spring | *Proteobacteria* | *Gammaproteobacteria* | *Burkholderiales* | *Burkholderiaceae* | *Burkholderia-Caballeronia-Paraburkholderia* |
| ASV842 | 4.87E-08 | Spring | *Verrucomicrobiota* | *Verrucomicrobiae* | *Chthoniobacterales* | *Chthoniobacteraceae* | *Candidatus Udaeobacter* |
| ASV848 | 0.033007729 | Spring | *Proteobacteria* | *Gammaproteobacteria* | *Burkholderiales* | *Burkholderiaceae* | *Burkholderia-Caballeronia-Paraburkholderia* |
| ASV852 | 0.046888652 | Spring | *Proteobacteria* | *Alphaproteobacteria* | *Micropepsales* | *Micropepsaceae* | *Unclassified_Micropepsaceae* |
| ASV853 | 1.16E-06 | Spring | *Proteobacteria* | *Alphaproteobacteria* | *Azospirillales* | *Unclassified* | *Unclassified_Azospirillales* |
| ASV855 | 0.049050533 | Spring | *Proteobacteria* | *Alphaproteobacteria* | *Rhizobiales* | *Xanthobacteraceae* | *Unclassified_Xanthobacteraceae* |
| ASV872 | 0.026766083 | Spring | *Firmicutes* | *Bacilli* | *Paenibacillales* | *Paenibacillaceae* | *Paenibacillus* |
| ASV876 | 0.001263106 | Spring | *Proteobacteria* | *Gammaproteobacteria* | *Burkholderiales* | *SC-I-84* | *Unclassified_SC-I-84* |
| ASV88 | 0.002296165 | Spring | *Bacteroidota* | *Bacteroidia* | *Chitinophagales* | *Chitinophagaceae* | *Chitinophaga* |
| ASV881 | 0.046335731 | Spring | *Verrucomicrobiota* | *Verrucomicrobiae* | *Verrucomicrobiales* | *Rubritaleaceae* | *Luteolibacter* |
| ASV891 | 0.004537175 | Spring | *Actinobacteriota* | *Actinobacteria* | *Frankiales* | *Acidothermaceae* | *Acidothermus* |
| ASV896 | 0.002820585 | Spring | *Proteobacteria* | *Gammaproteobacteria* | *Pseudomonadales* | *Pseudomonadaceae* | *Pseudomonas* |
| ASV897 | 1.99E-05 | Spring | *Proteobacteria* | *Alphaproteobacteria* | *Rhizobiales* | *Rhizobiales Incertae Sedis* | *Nordella* |
| ASV899 | 8.39E-05 | Spring | *Actinobacteriota* | *Actinobacteria* | *Propionibacteriales* | *Nocardioidaceae* | *Nocardioides* |
| ASV911 | 0.008718806 | Spring | *Proteobacteria* | *Alphaproteobacteria* | *Rhizobiales* | *Rhizobiales Incertae Sedis* | *Nordella* |
| ASV926 | 0.034934785 | Spring | *Actinobacteriota* | *Acidimicrobiia* | *Microtrichales* | *Unclassified* | *Unclassified_Microtrichales* |
| ASV942 | 0.003659431 | Spring | *Proteobacteria* | *Gammaproteobacteria* | *Xanthomonadales* | *Rhodanobacteraceae* | *Luteibacter* |
| ASV946 | 7.60E-04 | Spring | *Proteobacteria* | *Alphaproteobacteria* | *Caulobacterales* | *Caulobacteraceae* | *Unclassified_Caulobacteraceae* |
| ASV95 | 0.007504873 | Spring | *Bacteroidota* | *Bacteroidia* | *Sphingobacteriales* | *Sphingobacteriaceae* | *Pedobacter* |
| ASV952 | 0.004537175 | Spring | *Proteobacteria* | *Gammaproteobacteria* | *PLTA13* | *Unclassified* | *Unclassified_PLTA13* |
| ASV966 | 2.61E-04 | Spring | *Proteobacteria* | *Gammaproteobacteria* | *Xanthomonadales* | *Rhodanobacteraceae* | *Dokdonella* |
| ASV968 | 0.016768422 | Spring | *Cyanobacteria* | *Vampirivibrionia* | *Obscuribacterales* | *Obscuribacteraceae* | *Unclassified_Obscuribacteraceae* |
| ASV973 | 0.040550752 | Spring | *Proteobacteria* | *Alphaproteobacteria* | *Rhizobiales* | *Xanthobacteraceae* | *Unclassified_Xanthobacteraceae* |
| ASV974 | 3.62E-04 | Spring | *Actinobacteriota* | *Thermoleophilia* | *Gaiellales* | *Unclassified* | *Unclassified_Gaiellales* |
| ASV975 | 4.51E-05 | Spring | *Actinobacteriota* | *Actinobacteria* | *Propionibacteriales* | *Nocardioidaceae* | *Nocardioides* |
| ASV976 | 0.001843656 | Spring | *Proteobacteria* | *Alphaproteobacteria* | *Rhizobiales* | *Xanthobacteraceae* | *Bradyrhizobium* |
| ASV985 | 0.03594806 | Spring | *Firmicutes* | *Bacilli* | *Bacillales* | *Bacillaceae* | *Bacillus* |
| ASV991 | 0.036993475 | Spring | *Actinobacteriota* | *Actinobacteria* | *Streptomycetales* | *Streptomycetaceae* | *Streptacidiphilus* |
