# Supplementary material for: Beneficial microbial consortium improves winter rye performance by modulating bacterial communities in the rhizosphere and enhancing plant nutrient acquisition
Source: Front Plant Sci. 2023 Aug 28;14:1232288. doi: 10.3389/fpls.2023.1232288 (PMC10498285; doi:10.3389/fpls.2023.1232288)
Supplement: Supplementary file 9 [file Table_8.docx]

**Supplementary table 8.** ASVs that significantly differed in the rhizosphere of Control (Ctrl) or BMc inoculated Maize plants, under Organic or Conventional farming, in both sampling seasons. Differential abundance testing was performed via an ANOVA-like test implement in ANCOM-BC2 with Benjamini-Hochberg correction for each of the two sampling periods, as described in Supplementary table 7.

| **ASV** | **Phylum** | **Class** | **Order** | **Family** | **Genus** |
| --- | --- | --- | --- | --- | --- |
| ASV103 | *Proteobacteria* | *Alphaproteobacteria* | *Sphingomonadales* | *Sphingomonadaceae* | *Sphingomonas* |
| ASV1040 | *Chloroflexi* | *Ktedonobacteria* | *B12-WMSP1* | *Unclassified* | *Unclassified_B12-WMSP1* |
| ASV106 | *Proteobacteria* | *Alphaproteobacteria* | *Rhizobiales* | *Stappiaceae* | *Stappia* |
| ASV1152 | *Proteobacteria* | *Alphaproteobacteria* | *Rhizobiales* | *Rhizobiaceae* | *Aureimonas* |
| ASV133 | *Proteobacteria* | *Gammaproteobacteria* | *Burkholderiales* | *Oxalobacteraceae* | *Massilia* |
| ASV148 | *Bacteroidota* | *Bacteroidia* | *Chitinophagales* | *Chitinophagaceae* | *Chitinophaga* |
| ASV1519 | *Proteobacteria* | *Gammaproteobacteria* | *Xanthomonadales* | *Rhodanobacteraceae* | *Rhodanobacter* |
| ASV191 | *Firmicutes* | *Bacilli* | *Paenibacillales* | *Paenibacillaceae* | *Paenibacillus* |
| ASV265 | *Acidobacteriota* | *Acidobacteriae* | *Acidobacteriales* | *Acidobacteriaceae (Subgroup 1)* | *Acidipila-Silvibacterium* |
| ASV271 | *Proteobacteria* | *Alphaproteobacteria* | *Rhizobiales* | *Xanthobacteraceae* | *Bradyrhizobium* |
| ASV283 | *Bacteroidota* | *Bacteroidia* | *Sphingobacteriales* | *Sphingobacteriaceae* | *Mucilaginibacter* |
| ASV298 | *Proteobacteria* | *Alphaproteobacteria* | *Sphingomonadales* | *Sphingomonadaceae* | *Sphingomonas* |
| ASV323 | *Acidobacteriota* | *Blastocatellia* | *Blastocatellales* | *Blastocatellaceae* | *Unclassified_Blastocatellaceae* |
| ASV392 | *Acidobacteriota* | *Acidobacteriae* | *Acidobacteriales* | *Acidobacteriaceae (Subgroup 1)* | *Acidipila-Silvibacterium* |
| ASV400 | *Bacteroidota* | *Bacteroidia* | *Sphingobacteriales* | *Sphingobacteriaceae* | *Pedobacter* |
| ASV415 | *Proteobacteria* | *Gammaproteobacteria* | *Burkholderiales* | *Oxalobacteraceae* | *Massilia* |
| ASV420 | *Proteobacteria* | *Alphaproteobacteria* | *Rhizobiales* | *Xanthobacteraceae* | *Rhodopseudomonas* |
| ASV448 | *Proteobacteria* | *Alphaproteobacteria* | *Rhizobiales* | *Rhizobiaceae* | *Phyllobacterium* |
| ASV453 | *Actinobacteriota* | *Actinobacteria* | *Micrococcales* | *Micrococcaceae* | *Pseudarthrobacter* |
| ASV465 | *Verrucomicrobiota* | *Verrucomicrobiae* | *Verrucomicrobiales* | *Rubritaleaceae* | *Luteolibacter* |
| ASV466 | *Proteobacteria* | *Alphaproteobacteria* | *Sphingomonadales* | *Sphingomonadaceae* | *Sphingopyxis* |
| ASV482 | *Bacteroidota* | *Bacteroidia* | *Chitinophagales* | *Chitinophagaceae* | *Unclassified_Chitinophagaceae* |
| ASV498 | *Acidobacteriota* | *Blastocatellia* | *Blastocatellales* | *Blastocatellaceae* | *Unclassified_Blastocatellaceae* |
| ASV511 | *Actinobacteriota* | *Actinobacteria* | *Micrococcales* | *Micrococcaceae* | *Paeniglutamicibacter* |
| ASV523 | *Actinobacteriota* | *Actinobacteria* | *Streptomycetales* | *Streptomycetaceae* | *Streptomyces* |
| ASV532 | *Proteobacteria* | *Alphaproteobacteria* | *Rhizobiales* | *Xanthobacteraceae* | *Rhodopseudomonas* |
| ASV554 | *Proteobacteria* | *Gammaproteobacteria* | *Burkholderiales* | *Comamonadaceae* | *Rhizobacter* |
| ASV607 | *Proteobacteria* | *Alphaproteobacteria* | *Sphingomonadales* | *Sphingomonadaceae* | *Sphingomonas* |
| ASV609 | *Proteobacteria* | *Gammaproteobacteria* | *Xanthomonadales* | *Rhodanobacteraceae* | *Tahibacter* |
| ASV61 | *Proteobacteria* | *Gammaproteobacteria* | *Xanthomonadales* | *Rhodanobacteraceae* | *Luteibacter* |
| ASV63 | *Actinobacteriota* | *Actinobacteria* | *Micrococcales* | *Microbacteriaceae* | *Unclassified_Microbacteriaceae* |
| ASV656 | *Proteobacteria* | *Alphaproteobacteria* | *Rhizobiales* | *Xanthobacteraceae* | *Pseudolabrys* |
| ASV661 | *Proteobacteria* | *Alphaproteobacteria* | *Micavibrionales* | *Unclassified* | *Unclassified_Micavibrionales* |
| ASV668 | *Proteobacteria* | *Gammaproteobacteria* | *Xanthomonadales* | *Rhodanobacteraceae* | *Mizugakiibacter* |
| ASV72 | *Firmicutes* | *Bacilli* | *Bacillales* | *Bacillaceae* | *Bacillus* |
| ASV721 | *Actinobacteriota* | *Actinobacteria* | *Corynebacteriales* | *Nocardiaceae* | *Nocardia* |
| ASV822 | *Proteobacteria* | *Gammaproteobacteria* | *Burkholderiales* | *Comamonadaceae* | *Rhodoferax* |
| ASV828 | *Proteobacteria* | *Gammaproteobacteria* | *Burkholderiales* | *Burkholderiaceae* | *Burkholderia-Caballeronia-Paraburkholderia* |
| ASV848 | *Proteobacteria* | *Gammaproteobacteria* | *Burkholderiales* | *Burkholderiaceae* | *Burkholderia-Caballeronia-Paraburkholderia* |
| ASV853 | *Proteobacteria* | *Alphaproteobacteria* | *Azospirillales* | *Unclassified* | *Unclassified_Azospirillales* |
| ASV88 | *Bacteroidota* | *Bacteroidia* | *Chitinophagales* | *Chitinophagaceae* | *Chitinophaga* |
| ASV897 | *Proteobacteria* | *Alphaproteobacteria* | *Rhizobiales* | *Rhizobiales Incertae Sedis* | *Nordella* |
| ASV95 | *Bacteroidota* | *Bacteroidia* | *Sphingobacteriales* | *Sphingobacteriaceae* | *Pedobacter* |
