# Supplementary material for: Beneficial microbial consortium improves winter rye performance by modulating bacterial communities in the rhizosphere and enhancing plant nutrient acquisition
Source: Front Plant Sci. 2023 Aug 28;14:1232288. doi: 10.3389/fpls.2023.1232288 (PMC10498285; doi:10.3389/fpls.2023.1232288)
Supplement: Supplementary file 10 [file Table_9.docx]

**Supplementary table 9.** ASVs that significantly different in the rhizosphere of Maize plants and associated with either Control (Ctrl) or BMc inoculated Maize plants, under Organic or Conventional farming, in both sampling seasons. Differential abundance testing was performed via an ANOVA-like test implement in ANCOM-BC2 with Benjamini-Hochberg correction for each of the two sampling periods, as described in Supplementary table 7. Following the ANOVA-like test, we performed logistic regression models to identify ASVs that could be unique predictors for each combined treatment. The *p*-values of the logistic regression models were corrected via Benjamini-Hochberg correction.

| **ASV** | **p_value_adj** | **Model Coefficient** | **Associated Treatment** | **Season** | **Phylum** | **Class** | **Order** | **Family** | **Genus** | **Species** |
| --- | --- | --- | --- | --- | --- | --- | --- | --- | --- | --- |
| ASV1030 | 0.010197 | 0.49 | Integrated_BMs | Autumn | *Actinobacteriota* | *Actinobacteria* | *Frankiales* | *Geodermatophilaceae* | *Blastococcus* | *Unclassified* |
| ASV1123 | 0.049945 | -2.45 | Integrated_BMs | Autumn | *Proteobacteria* | *Gammaproteobacteria* | *Pseudomonadales* | *Pseudomonadaceae* | *Pseudomonas* | *Pseudomonas putida* |
| ASV1144 | 0.049945 | -2.45 | Integrated_BMs | Autumn | *Proteobacteria* | *Gammaproteobacteria* | *Xanthomonadales* | *Xanthomonadaceae* | *Lysobacter* | *Unclassified* |
| ASV1180 | 0.011798 | -5.03 | Integrated_BMs | Autumn | *Proteobacteria* | *Gammaproteobacteria* | *Xanthomonadales* | *Xanthomonadaceae* | *Lysobacter* | *Lysobacter sp. cf310* |
| ASV1183 | 0.010197 | 2.31 | Integrated_BMs | Autumn | *Proteobacteria* | *Alphaproteobacteria* | *Rhizobiales* | *Xanthobacteraceae* | *Unclassified_Xanthobacteraceae* | *Unclassified* |
| ASV1201 | 0.049945 | -2.11 | Integrated_BMs | Autumn | *Chloroflexi* | *Chloroflexia* | *Chloroflexales* | *Herpetosiphonaceae* | *Herpetosiphon* | *Unclassified* |
| ASV1213 | 0.010197 | 0.25 | Integrated_BMs | Autumn | *Proteobacteria* | *Gammaproteobacteria* | *Burkholderiales* | *Oxalobacteraceae* | *Herbaspirillum* | *Herbaspirillum lusitanum P6-12* |
| ASV1231 | 0.014062 | 0.16 | Integrated_BMs | Autumn | *Proteobacteria* | *Gammaproteobacteria* | *Burkholderiales* | *Oxalobacteraceae* | *Unclassified_Oxalobacteraceae* | *Unclassified* |
| ASV1315 | 0.010197 | 0.95 | Integrated_BMs | Autumn | *Proteobacteria* | *Gammaproteobacteria* | *Burkholderiales* | *Burkholderiaceae* | *Burkholderia-Caballeronia-Paraburkholderia* | *Unclassified* |
| ASV1320 | 0.033838 | 0.22 | Integrated_BMs | Autumn | *Bacteroidota* | *Bacteroidia* | *Sphingobacteriales* | *Sphingobacteriaceae* | *Mucilaginibacter* | *Mucilaginibacter dorajii* |
| ASV1360 | 0.010197 | 1.58 | Integrated_BMs | Autumn | *Bacteroidota* | *Bacteroidia* | *Chitinophagales* | *Chitinophagaceae* | *Taibaiella* | *Unclassified* |
| ASV1421 | 0.033838 | -5.39 | Integrated_BMs | Autumn | *Proteobacteria* | *Alphaproteobacteria* | *Sphingomonadales* | *Sphingomonadaceae* | *Sphingomonas* | *Sphingomonas hengshuiensis* |
| ASV1434 | 0.010786 | 0.65 | Integrated_BMs | Autumn | *Chloroflexi* | *Ktedonobacteria* | *C0119* | *Unclassified* | *Unclassified_C0119* | *Unclassified* |
| ASV1457 | 0.033838 | -3.87 | Integrated_BMs | Autumn | *Bacteroidota* | *Bacteroidia* | *Sphingobacteriales* | *Sphingobacteriaceae* | *Mucilaginibacter* | *Unclassified* |
| ASV1465 | 0.033838 | -2.61 | Integrated_BMs | Autumn | *Proteobacteria* | *Gammaproteobacteria* | *Burkholderiales* | *Comamonadaceae* | *Unclassified_Comamonadaceae* | *Unclassified* |
| ASV1558 | 0.021633 | 0.4 | Integrated_BMs | Autumn | *Proteobacteria* | *Alphaproteobacteria* | *Acetobacterales* | *Acetobacteraceae* | *Unclassified_Acetobacteraceae* | *Unclassified* |
| ASV1698 | 0.010197 | 3.22 | Integrated_BMs | Autumn | *Proteobacteria* | *Gammaproteobacteria* | *Burkholderiales* | *Oxalobacteraceae* | *Massilia* | *Massilia timonae CCUG 45783* |
| ASV1818 | 0.049945 | -2.72 | Integrated_BMs | Autumn | *Bacteroidota* | *Bacteroidia* | *Cytophagales* | *Spirosomaceae* | *Dyadobacter* | *Unclassified* |
| ASV2097 | 0.010197 | 3.16 | Integrated_BMs | Autumn | *Nitrospirota* | *Nitrospiria* | *Nitrospirales* | *Nitrospiraceae* | *Nitrospira* | *Unclassified* |
| ASV2173 | 9.29E-04 | 54.75 | Integrated_BMs | Autumn | *Proteobacteria* | *Gammaproteobacteria* | *Burkholderiales* | *Oxalobacteraceae* | *Massilia* | *Massilia sp.* |
| ASV2276 | 0.033838 | 0.56 | Integrated_BMs | Autumn | *Abditibacteriota* | *Abditibacteria* | *Abditibacteriales* | *Abditibacteriaceae* | *Abditibacterium* | *Unclassified* |
| ASV2380 | 0.011964 | 0.92 | Integrated_BMs | Autumn | *Proteobacteria* | *Gammaproteobacteria* | *Burkholderiales* | *Oxalobacteraceae* | *Massilia* | *Unclassified* |
| ASV310 | 0.037281 | -0.12 | Integrated_BMs | Autumn | *Proteobacteria* | *Alphaproteobacteria* | *Rhizobiales* | *Xanthobacteraceae* | *Afipia* | *Afipia sp. 9(2017)* |
| ASV3146 | 0.010197 | 3.22 | Integrated_BMs | Autumn | *Proteobacteria* | *Gammaproteobacteria* | *Burkholderiales* | *Burkholderiaceae* | *Burkholderia-Caballeronia-Paraburkholderia* | *Unclassified* |
| ASV317 | 0.049945 | -1.53 | Integrated_BMs | Autumn | *Proteobacteria* | *Alphaproteobacteria* | *Sphingomonadales* | *Sphingomonadaceae* | *Sphingomonas* | *Sphingomonas sp. TSK-1* |
| ASV3182 | 0.010197 | 2.3 | Integrated_BMs | Autumn | *Proteobacteria* | *Gammaproteobacteria* | *Burkholderiales* | *Burkholderiaceae* | *Burkholderia-Caballeronia-Paraburkholderia* | *Unclassified* |
| ASV325 | 0.049945 | -1.1 | Integrated_BMs | Autumn | *Proteobacteria* | *Alphaproteobacteria* | *Sphingomonadales* | *Sphingomonadaceae* | *Sphingomonas* | *Sphingomonas sp. JGI 0001002-C18* |
| ASV3414 | 0.010197 | 5.7 | Integrated_BMs | Autumn | *Proteobacteria* | *Gammaproteobacteria* | *Xanthomonadales* | *Xanthomonadaceae* | *Thermomonas* | *Unclassified* |
| ASV3661 | 0.015831 | 0.87 | Integrated_BMs | Autumn | *Bacteroidota* | *Bacteroidia* | *Flavobacteriales* | *Flavobacteriaceae* | *Flavobacterium* | *Unclassified* |
| ASV3811 | 0.010197 | 4.07 | Integrated_BMs | Autumn | *Proteobacteria* | *Gammaproteobacteria* | *Burkholderiales* | *Comamonadaceae* | *Unclassified_Comamonadaceae* | *Unclassified* |
| ASV3903 | 0.010197 | 9.78 | Integrated_BMs | Autumn | *Bacteroidota* | *Bacteroidia* | *Cytophagales* | *Spirosomaceae* | *Dyadobacter* | *Unclassified* |
| ASV400 | 0.021633 | -1.03 | Integrated_BMs | Autumn | *Bacteroidota* | *Bacteroidia* | *Sphingobacteriales* | *Sphingobacteriaceae* | *Pedobacter* | *Unclassified* |
| ASV409 | 0.0302 | 0.1 | Integrated_BMs | Autumn | *Proteobacteria* | *Gammaproteobacteria* | *Burkholderiales* | *Burkholderiaceae* | *Burkholderia-Caballeronia-Paraburkholderia* | *Burkholderia sp. LMG 21262* |
| ASV4172 | 0.010197 | 5.7 | Integrated_BMs | Autumn | *Proteobacteria* | *Gammaproteobacteria* | *Xanthomonadales* | *Rhodanobacteraceae* | *Luteibacter* | *Luteibacter yeojuensis* |
| ASV437 | 0.049945 | 0.11 | Integrated_BMs | Autumn | *Bacteroidota* | *Bacteroidia* | *Sphingobacteriales* | *Sphingobacteriaceae* | *Mucilaginibacter* | *Unclassified* |
| ASV451 | 0.010197 | 0.24 | Integrated_BMs | Autumn | *Verrucomicrobiota* | *Verrucomicrobiae* | *Chthoniobacterales* | *Terrimicrobiaceae* | *Terrimicrobium* | *Unclassified* |
| ASV453 | 9.29E-04 | 8.2 | Integrated_BMs | Autumn | *Actinobacteriota* | *Actinobacteria* | *Micrococcales* | *Micrococcaceae* | *Pseudarthrobacter* | *Arthrobacter sp. Soil761* |
| ASV478 | 9.29E-04 | 1.33 | Integrated_BMs | Autumn | *Bacteroidota* | *Bacteroidia* | *Sphingobacteriales* | *Sphingobacteriaceae* | *Pedobacter* | *Sphingobacteriaceae bacterium GW460-11-11-14-LB5* |
| ASV486 | 0.033838 | -4.18 | Integrated_BMs | Autumn | *Proteobacteria* | *Alphaproteobacteria* | *Rhizobiales* | *Devosiaceae* | *Devosia* | *Unclassified* |
| ASV498 | 0.033838 | -3.31 | Integrated_BMs | Autumn | *Acidobacteriota* | *Blastocatellia* | *Blastocatellales* | *Blastocatellaceae* | *Unclassified_Blastocatellaceae* | *Unclassified* |
| ASV511 | 0.033838 | -1.62 | Integrated_BMs | Autumn | *Actinobacteriota* | *Actinobacteria* | *Micrococcales* | *Micrococcaceae* | *Paeniglutamicibacter* | *Arthrobacter sp.* |
| ASV5349 | 0.016004 | 1.48 | Integrated_BMs | Autumn | *Proteobacteria* | *Gammaproteobacteria* | *Burkholderiales* | *Oxalobacteraceae* | *Massilia* | *Massilia sp. 9096* |
| ASV5363 | 0.010197 | 4.73 | Integrated_BMs | Autumn | *Bacteroidota* | *Bacteroidia* | *Sphingobacteriales* | *Sphingobacteriaceae* | *Mucilaginibacter* | *Mucilaginibacter sp. HL-25* |
| ASV555 | 0.012523 | 0.29 | Integrated_BMs | Autumn | *Proteobacteria* | *Alphaproteobacteria* | *Sphingomonadales* | *Sphingomonadaceae* | *Sphingomonas* | *Unclassified* |
| ASV561 | 0.049945 | -2.59 | Integrated_BMs | Autumn | *Proteobacteria* | *Alphaproteobacteria* | *Rhizobiales* | *Rhizobiaceae* | *Pseudochrobactrum* | *Pseudochrobactrum sp.* |
| ASV5772 | 0.010197 | 5.88 | Integrated_BMs | Autumn | *Actinobacteriota* | *Actinobacteria* | *Bifidobacteriales* | *Bifidobacteriaceae* | *Bifidobacterium* | *Bifidobacterium pseudocatenulatum* |
| ASV593 | 9.29E-04 | 11.04 | Integrated_BMs | Autumn | *Actinobacteriota* | *Actinobacteria* | *Streptomycetales* | *Streptomycetaceae* | *Kitasatospora* | *Kitasatospora gansuensis* |
| ASV606 | 0.019357 | 0.19 | Integrated_BMs | Autumn | *Bacteroidota* | *Bacteroidia* | *Flavobacteriales* | *Weeksellaceae* | *Chryseobacterium* | *Unclassified* |
| ASV640 | 0.033838 | -0.87 | Integrated_BMs | Autumn | *Proteobacteria* | *Gammaproteobacteria* | *Burkholderiales* | *Comamonadaceae* | *Rhizobacter* | *Unclassified* |
| ASV656 | 0.033838 | -1.9 | Integrated_BMs | Autumn | *Proteobacteria* | *Alphaproteobacteria* | *Rhizobiales* | *Xanthobacteraceae* | *Pseudolabrys* | *Unclassified* |
| ASV683 | 0.033838 | -1.46 | Integrated_BMs | Autumn | *Chloroflexi* | *Chloroflexia* | *Chloroflexales* | *Roseiflexaceae* | *Unclassified_Roseiflexaceae* | *Unclassified* |
| ASV722 | 0.049945 | -1.53 | Integrated_BMs | Autumn | *Actinobacteriota* | *Actinobacteria* | *Micrococcales* | *Microbacteriaceae* | *Lysinimonas* | *Unclassified* |
| ASV747 | 9.29E-04 | 65.98 | Integrated_BMs | Autumn | *Proteobacteria* | *Gammaproteobacteria* | *Burkholderiales* | *Comamonadaceae* | *Variovorax* | *Variovorax sp. PAMC 28711* |
| ASV801 | 0.011798 | 0.39 | Integrated_BMs | Autumn | *Proteobacteria* | *Gammaproteobacteria* | *Burkholderiales* | *Comamonadaceae* | *Variovorax* | *Variovorax sp. PAMC 28711* |
| ASV818 | 0.049945 | -2.6 | Integrated_BMs | Autumn | *Chloroflexi* | *Chloroflexia* | *Chloroflexales* | *Roseiflexaceae* | *Unclassified_Roseiflexaceae* | *Unclassified* |
| ASV819 | 0.049945 | -1 | Integrated_BMs | Autumn | *Proteobacteria* | *Gammaproteobacteria* | *Burkholderiales* | *Comamonadaceae* | *Rhizobacter* | *Unclassified* |
| ASV843 | 0.006037 | 0.59 | Integrated_BMs | Autumn | *Verrucomicrobiota* | *Verrucomicrobiae* | *Verrucomicrobiales* | *Rubritaleaceae* | *Luteolibacter* | *Unclassified* |
| ASV848 | 0.009945 | 0.34 | Integrated_BMs | Autumn | *Proteobacteria* | *Gammaproteobacteria* | *Burkholderiales* | *Burkholderiaceae* | *Burkholderia-Caballeronia-Paraburkholderia* | *Unclassified* |
| ASV849 | 0.049945 | -1.61 | Integrated_BMs | Autumn | *Proteobacteria* | *Alphaproteobacteria* | *Rhizobiales* | *Xanthobacteraceae* | *Unclassified_Xanthobacteraceae* | *Unclassified* |
| ASV853 | 0.049945 | -3.46 | Integrated_BMs | Autumn | *Proteobacteria* | *Alphaproteobacteria* | *Azospirillales* | *Unclassified* | *Unclassified_Azospirillales* | *Unclassified* |
| ASV892 | 0.033838 | 0.18 | Integrated_BMs | Autumn | *Actinobacteriota* | *Actinobacteria* | *Propionibacteriales* | *Nocardioidaceae* | *Nocardioides* | *Unclassified* |
| ASV909 | 0.033838 | -3.23 | Integrated_BMs | Autumn | *Proteobacteria* | *Alphaproteobacteria* | *Rhizobiales* | *Unclassified* | *Unclassified_Rhizobiales* | *Unclassified* |
| ASV921 | 0.006037 | 1 | Integrated_BMs | Autumn | *Bacteroidota* | *Bacteroidia* | *Sphingobacteriales* | *Sphingobacteriaceae* | *Mucilaginibacter* | *Mucilaginibacter dorajii* |
| ASV958 | 0.008459 | -4.5 | Integrated_BMs | Autumn | *Proteobacteria* | *Gammaproteobacteria* | *Xanthomonadales* | *Xanthomonadaceae* | *Arenimonas* | *Unclassified* |
| ASV1206 | 0.013915 | 0.47 | Integrated_BMs | Autumn | *Bacteroidota* | *Bacteroidia* | *Sphingobacteriales* | *Sphingobacteriaceae* | *Pedobacter* | *Unclassified* |
| ASV2331 | 0.027819 | 0.67 | Integrated_BMs | Autumn | *Actinobacteriota* | *Thermoleophilia* | *Solirubrobacterales* | *Solirubrobacteraceae* | *Conexibacter* | *Unclassified* |
| ASV235 | 0.008188 | 0.13 | Integrated_BMs | Autumn | *Bacteroidota* | *Bacteroidia* | *Sphingobacteriales* | *Sphingobacteriaceae* | *Pedobacter* | *Pedobacter panaciterrae* |
| ASV329 | 0.035916 | 0.09 | Integrated_BMs | Autumn | *Proteobacteria* | *Gammaproteobacteria* | *Burkholderiales* | *Burkholderiaceae* | *Burkholderia-Caballeronia-Paraburkholderia* | *Paraburkholderia hospita* |
| ASV436 | 0.049945 | -0.14 | Integrated_BMs | Autumn | *Proteobacteria* | *Gammaproteobacteria* | *Pseudomonadales* | *Pseudomonadaceae* | *Pseudomonas* | *Pseudomonas trivialis* |
| ASV489 | 0.010197 | 0.26 | Integrated_BMs | Autumn | *Bacteroidota* | *Bacteroidia* | *Sphingobacteriales* | *Sphingobacteriaceae* | *Pedobacter* | *Unclassified* |
| ASV562 | 0.033838 | -2.4 | Integrated_BMs | Autumn | *Actinobacteriota* | *MB-A2-108* | *Unclassified* | *Unclassified* | *Unclassified_MB-A2-108* | *Unclassified* |
| ASV565 | 0.014062 | 0.16 | Integrated_BMs | Autumn | *Bacteroidota* | *Bacteroidia* | *Chitinophagales* | *Chitinophagaceae* | *Taibaiella* | *Taibaiella sp.* |
| ASV571 | 0.021633 | 0.32 | Integrated_BMs | Autumn | *Verrucomicrobiota* | *Verrucomicrobiae* | *Verrucomicrobiales* | *Rubritaleaceae* | *Luteolibacter* | *Unclassified* |
| ASV575 | 0.017226 | 0.17 | Integrated_BMs | Autumn | *Proteobacteria* | *Alphaproteobacteria* | *Acetobacterales* | *Acetobacteraceae* | *Unclassified_Acetobacteraceae* | *Unclassified* |
| ASV1723 | 0.020886 | 0.46 | Integrated_BMs | Autumn | *Proteobacteria* | *Alphaproteobacteria* | *Rickettsiales* | *Candidatus Jidaibacter* | *Unclassified_Candidatus Jidaibacter* | *Unclassified* |
| ASV541 | 0.036751 | -0.2 | Integrated_BMs | Autumn | *Bacteroidota* | *Bacteroidia* | *Cytophagales* | *Spirosomaceae* | *Dyadobacter* | *Dyadobacter sp. THG-J3* |
| ASV723 | 0.019093 | -0.26 | Integrated_BMs | Autumn | *Myxococcota* | *Polyangia* | *Polyangiales* | *BIrii41* | *Unclassified_BIrii41* | *Unclassified* |
| ASV749 | 0.011798 | -2.89 | Integrated_BMs | Autumn | *Actinobacteriota* | *Actinobacteria* | *Frankiales* | *Sporichthyaceae* | *hgcI clade* | *Actinobacteria bacterium IMCC26103* |
| ASV1851 | 0.035364 | 0.32 | Integrated_BMs | Autumn | *Proteobacteria* | *Gammaproteobacteria* | *Xanthomonadales* | *Rhodanobacteraceae* | *Unclassified_Rhodanobacteraceae* | *Unclassified* |
| ASV163 | 0.049823 | -0.06 | Integrated_BMs | Autumn | *Proteobacteria* | *Alphaproteobacteria* | *Rhizobiales* | *Devosiaceae* | *Devosia* | *Unclassified* |
| ASV1794 | 0.049945 | -6.73 | Integrated_BMs | Autumn | *Proteobacteria* | *Gammaproteobacteria* | *Burkholderiales* | *Oxalobacteraceae* | *[Aquaspirillum] arcticum group* | *Unclassified* |
| ASV205 | 0.006037 | -0.32 | Integrated_BMs | Autumn | *Bacteroidota* | *Bacteroidia* | *Sphingobacteriales* | *Sphingobacteriaceae* | *Mucilaginibacter* | *Unclassified* |
| ASV333 | 0.015363 | -0.25 | Integrated_BMs | Autumn | *Proteobacteria* | *Gammaproteobacteria* | *Burkholderiales* | *Comamonadaceae* | *Rhizobacter* | *Rhizobacter gummiphilus* |
| ASV133 | 0.014932 | -0.06 | Integrated_BMs | Autumn | *Proteobacteria* | *Gammaproteobacteria* | *Burkholderiales* | *Oxalobacteraceae* | *Massilia* | *Unclassified* |
| ASV702 | 0.013968 | -0.47 | Integrated_BMs | Autumn | *Bacteroidota* | *Bacteroidia* | *Chitinophagales* | *Chitinophagaceae* | *Unclassified_Chitinophagaceae* | *Unclassified* |
| ASV159 | 0.018155 | 0.12 | Integrated_BMs | Autumn | *Actinobacteriota* | *Actinobacteria* | *Streptomycetales* | *Streptomycetaceae* | *Kitasatospora* | *Kitasatospora sp. MBT63* |
| ASV220 | 9.29E-04 | 3.59 | Integrated_BMs | Autumn | *Bacteroidota* | *Bacteroidia* | *Cytophagales* | *Spirosomaceae* | *Dyadobacter* | *Unclassified* |
| ASV595 | 0.027787 | 0.52 | Integrated_BMs | Autumn | *Acidobacteriota* | *Acidobacteriae* | *Bryobacterales* | *Bryobacteraceae* | *Bryobacter* | *Unclassified* |
| ASV609 | 0.020661 | 0.34 | Integrated_BMs | Autumn | *Proteobacteria* | *Gammaproteobacteria* | *Xanthomonadales* | *Rhodanobacteraceae* | *Tahibacter* | *Unclassified* |
| ASV678 | 0.013474 | 0.39 | Integrated_BMs | Autumn | *Verrucomicrobiota* | *Verrucomicrobiae* | *Chthoniobacterales* | *Chthoniobacteraceae* | *Candidatus Udaeobacter* | *Unclassified* |
| ASV299 | 0.049945 | 0.08 | Integrated_BMs | Autumn | *Firmicutes* | *Bacilli* | *Bacillales* | *Bacillaceae* | *Bacillus* | *Unclassified* |
| ASV482 | 0.033838 | 0.19 | Integrated_BMs | Autumn | *Bacteroidota* | *Bacteroidia* | *Chitinophagales* | *Chitinophagaceae* | *Unclassified_Chitinophagaceae* | *Unclassified* |
| ASV756 | 0.018099 | 0.18 | Integrated_BMs | Autumn | *Proteobacteria* | *Alphaproteobacteria* | *Holosporales* | *Holosporaceae* | *Unclassified_Holosporaceae* | *Unclassified* |
| ASV923 | 0.049945 | 0.19 | Integrated_BMs | Autumn | *Proteobacteria* | *Alphaproteobacteria* | *Rhizobiales* | *Xanthobacteraceae* | *Bradyrhizobium* | *Bradyrhizobium sp. ICMP 14754* |
| ASV40 | 0.016004 | 0.01 | Integrated_BMs | Autumn | *Bacteroidota* | *Bacteroidia* | *Sphingobacteriales* | *Sphingobacteriaceae* | *Pedobacter* | *Pedobacter sp. S8-2* |
| ASV420 | 0.010197 | 0.21 | Integrated_BMs | Autumn | *Proteobacteria* | *Alphaproteobacteria* | *Rhizobiales* | *Xanthobacteraceae* | *Rhodopseudomonas* | *alpha proteobacterium SK50-23* |
| ASV828 | 9.29E-04 | 35.1 | Integrated_BMs | Autumn | *Proteobacteria* | *Gammaproteobacteria* | *Burkholderiales* | *Burkholderiaceae* | *Burkholderia-Caballeronia-Paraburkholderia* | *Burkholderia sp. DCY113* |
| ASV517 | 0.033838 | 0.26 | Integrated_BMs | Autumn | *Actinobacteriota* | *Actinobacteria* | *Micrococcales* | *Micrococcaceae* | *Arthrobacter* | *Arthrobacter sp. Hiyo1* |
| ASV298 | 0.033838 | 0.1 | Integrated_BMs | Autumn | *Proteobacteria* | *Alphaproteobacteria* | *Sphingomonadales* | *Sphingomonadaceae* | *Sphingomonas* | *Unclassified* |
| ASV218 | 0.033838 | 0.05 | Integrated_BMs | Autumn | *Bacteroidota* | *Bacteroidia* | *Sphingobacteriales* | *Sphingobacteriaceae* | *Pedobacter* | *Beta vulgaris subsp. vulgaris* |
| ASV239 | 0.010197 | 0.08 | Integrated_BMs | Autumn | *Proteobacteria* | *Gammaproteobacteria* | *Pseudomonadales* | *Pseudomonadaceae* | *Pseudomonas* | *Unclassified* |
| ASV243 | 0.033527 | -0.16 | Integrated_BMs | Autumn | *Bacteroidota* | *Bacteroidia* | *Chitinophagales* | *Chitinophagaceae* | *Ferruginibacter* | *Unclassified* |
| ASV156 | 9.29E-04 | 19.88 | Integrated_BMs | Autumn | *Bacteroidota* | *Bacteroidia* | *Cytophagales* | *Spirosomaceae* | *Dyadobacter* | *Dyadobacter fermentans* |
| ASV191 | 0.010197 | 0.14 | Integrated_BMs | Autumn | *Firmicutes* | *Bacilli* | *Paenibacillales* | *Paenibacillaceae* | *Paenibacillus* | *Unclassified* |
| ASV128 | 0.047171 | -0.12 | Integrated_BMs | Autumn | *Verrucomicrobiota* | *Verrucomicrobiae* | *Verrucomicrobiales* | *Rubritaleaceae* | *Luteolibacter* | *Unclassified* |
| ASV94 | 0.049945 | -0.04 | Integrated_BMs | Autumn | *Proteobacteria* | *Alphaproteobacteria* | *Rhizobiales* | *Rhizobiaceae* | *Allorhizobium-Neorhizobium-Pararhizobium-Rhizobium* | *Pararhizobium giardinii* |
| ASV52 | 0.033838 | -0.03 | Integrated_BMs | Autumn | *Proteobacteria* | *Alphaproteobacteria* | *Sphingomonadales* | *Sphingomonadaceae* | *Sphingobium* | *Sphingobium sp. PDD-35b-9* |
| ASV80 | 0.030903 | -0.04 | Integrated_BMs | Autumn | *Proteobacteria* | *Gammaproteobacteria* | *Burkholderiales* | *Oxalobacteraceae* | *Massilia* | *Unclassified* |
| ASV30 | 0.0302 | -0.04 | Integrated_BMs | Autumn | *Bacteroidota* | *Bacteroidia* | *Flavobacteriales* | *Flavobacteriaceae* | *Flavobacterium* | *Flavobacterium sp. Amb3* |
| ASV72 | 0.010197 | 0.06 | Integrated_BMs | Autumn | *Firmicutes* | *Bacilli* | *Bacillales* | *Bacillaceae* | *Bacillus* | *Unclassified* |
| ASV60 | 0.033838 | -0.03 | Integrated_BMs | Autumn | *Proteobacteria* | *Alphaproteobacteria* | *Rhizobiales* | *Rhizobiaceae* | *Allorhizobium-Neorhizobium-Pararhizobium-Rhizobium* | *Unclassified* |
| ASV73 | 0.02832 | 0.02 | Integrated_BMs | Autumn | *Bacteroidota* | *Bacteroidia* | *Sphingobacteriales* | *Sphingobacteriaceae* | *Pedobacter* | *Pedobacter sp. S8-2* |
| ASV59 | 0.049823 | -0.03 | Integrated_BMs | Autumn | *Proteobacteria* | *Alphaproteobacteria* | *Sphingomonadales* | *Sphingomonadaceae* | *Sphingopyxis* | *Unclassified* |
| ASV49 | 9.29E-04 | 4.37 | Integrated_BMs | Autumn | *Proteobacteria* | *Alphaproteobacteria* | *Rhizobiales* | *Rhizobiaceae* | *Allorhizobium-Neorhizobium-Pararhizobium-Rhizobium* | *Unclassified* |
| ASV1123 | 0.049945 | -2.45 | Integrated_Ctrl | Autumn | *Proteobacteria* | *Gammaproteobacteria* | *Pseudomonadales* | *Pseudomonadaceae* | *Pseudomonas* | *Pseudomonas putida* |
| ASV1144 | 0.049945 | -2.45 | Integrated_Ctrl | Autumn | *Proteobacteria* | *Gammaproteobacteria* | *Xanthomonadales* | *Xanthomonadaceae* | *Lysobacter* | *Unclassified* |
| ASV1201 | 0.049945 | -2.11 | Integrated_Ctrl | Autumn | *Chloroflexi* | *Chloroflexia* | *Chloroflexales* | *Herpetosiphonaceae* | *Herpetosiphon* | *Unclassified* |
| ASV1421 | 0.033838 | -5.39 | Integrated_Ctrl | Autumn | *Proteobacteria* | *Alphaproteobacteria* | *Sphingomonadales* | *Sphingomonadaceae* | *Sphingomonas* | *Sphingomonas hengshuiensis* |
| ASV1457 | 0.033838 | -3.87 | Integrated_Ctrl | Autumn | *Bacteroidota* | *Bacteroidia* | *Sphingobacteriales* | *Sphingobacteriaceae* | *Mucilaginibacter* | *Unclassified* |
| ASV1465 | 0.033838 | -2.61 | Integrated_Ctrl | Autumn | *Proteobacteria* | *Gammaproteobacteria* | *Burkholderiales* | *Comamonadaceae* | *Unclassified_Comamonadaceae* | *Unclassified* |
| ASV1497 | 0.033838 | -5.86 | Integrated_Ctrl | Autumn | *Chloroflexi* | *Ktedonobacteria* | *C0119* | *Unclassified* | *Unclassified_C0119* | *Unclassified* |
| ASV1818 | 0.049945 | -2.72 | Integrated_Ctrl | Autumn | *Bacteroidota* | *Bacteroidia* | *Cytophagales* | *Spirosomaceae* | *Dyadobacter* | *Unclassified* |
| ASV2204 | 0.010197 | 1.34 | Integrated_Ctrl | Autumn | *Actinobacteriota* | *Actinobacteria* | *Streptomycetales* | *Streptomycetaceae* | *Streptomyces* | *Streptomyces sp.* |
| ASV280 | 0.021633 | -2.01 | Integrated_Ctrl | Autumn | *Actinobacteriota* | *Actinobacteria* | *Micrococcales* | *Microbacteriaceae* | *Frigoribacterium* | *Unclassified* |
| ASV3047 | 0.010197 | 2.76 | Integrated_Ctrl | Autumn | *Acidobacteriota* | *Acidobacteriae* | *Acidobacteriales* | *Unclassified* | *Unclassified_Acidobacteriales* | *Unclassified* |
| ASV312 | 0.011798 | -2.38 | Integrated_Ctrl | Autumn | *Bacteroidota* | *Bacteroidia* | *Chitinophagales* | *Chitinophagaceae* | *Taibaiella* | *Unclassified* |
| ASV317 | 0.049945 | -1.53 | Integrated_Ctrl | Autumn | *Proteobacteria* | *Alphaproteobacteria* | *Sphingomonadales* | *Sphingomonadaceae* | *Sphingomonas* | *Sphingomonas sp. TSK-1* |
| ASV325 | 0.049945 | -1.1 | Integrated_Ctrl | Autumn | *Proteobacteria* | *Alphaproteobacteria* | *Sphingomonadales* | *Sphingomonadaceae* | *Sphingomonas* | *Sphingomonas sp. JGI 0001002-C18* |
| ASV366 | 0.011798 | -3.02 | Integrated_Ctrl | Autumn | *Actinobacteriota* | *Actinobacteria* | *Micrococcales* | *Microbacteriaceae* | *Schumannella* | *Unclassified* |
| ASV387 | 0.008459 | -1.68 | Integrated_Ctrl | Autumn | *Bacteroidota* | *Bacteroidia* | *Chitinophagales* | *Chitinophagaceae* | *Ferruginibacter* | *Unclassified* |
| ASV409 | 0.033838 | -0.52 | Integrated_Ctrl | Autumn | *Proteobacteria* | *Gammaproteobacteria* | *Burkholderiales* | *Burkholderiaceae* | *Burkholderia-Caballeronia-Paraburkholderia* | *Burkholderia sp. LMG 21262* |
| ASV423 | 0.021633 | -2.16 | Integrated_Ctrl | Autumn | *Proteobacteria* | *Gammaproteobacteria* | *Xanthomonadales* | *Xanthomonadaceae* | *Pseudoxanthomonas* | *Pseudoxanthomonas yeongjuensis* |
| ASV437 | 0.011798 | -4.27 | Integrated_Ctrl | Autumn | *Bacteroidota* | *Bacteroidia* | *Sphingobacteriales* | *Sphingobacteriaceae* | *Mucilaginibacter* | *Unclassified* |
| ASV463 | 0.033838 | -8.1 | Integrated_Ctrl | Autumn | *Proteobacteria* | *Gammaproteobacteria* | *Pseudomonadales* | *Pseudomonadaceae* | *Pseudomonas* | *Pseudomonas putida* |
| ASV466 | 0.049945 | -0.77 | Integrated_Ctrl | Autumn | *Proteobacteria* | *Alphaproteobacteria* | *Sphingomonadales* | *Sphingomonadaceae* | *Sphingopyxis* | *Sphingopyxis sp. QXT-31* |
| ASV468 | 0.010197 | -0.46 | Integrated_Ctrl | Autumn | *Bacteroidota* | *Bacteroidia* | *Chitinophagales* | *Chitinophagaceae* | *Terrimonas* | *Unclassified* |
| ASV511 | 0.033838 | -1.62 | Integrated_Ctrl | Autumn | *Actinobacteriota* | *Actinobacteria* | *Micrococcales* | *Micrococcaceae* | *Paeniglutamicibacter* | *Arthrobacter sp.* |
| ASV512 | 0.033838 | -2.31 | Integrated_Ctrl | Autumn | *Proteobacteria* | *Alphaproteobacteria* | *Sphingomonadales* | *Sphingomonadaceae* | *Unclassified_Sphingomonadaceae* | *Unclassified* |
| ASV539 | 0.049945 | -0.2 | Integrated_Ctrl | Autumn | *Bacteroidota* | *Bacteroidia* | *Chitinophagales* | *Chitinophagaceae* | *Taibaiella* | *Unclassified* |
| ASV561 | 0.049945 | -2.59 | Integrated_Ctrl | Autumn | *Proteobacteria* | *Alphaproteobacteria* | *Rhizobiales* | *Rhizobiaceae* | *Pseudochrobactrum* | *Pseudochrobactrum sp.* |
| ASV598 | 0.011798 | -3.22 | Integrated_Ctrl | Autumn | *Proteobacteria* | *Gammaproteobacteria* | *Xanthomonadales* | *Rhodanobacteraceae* | *Tahibacter* | *Unclassified* |
| ASV606 | 0.049945 | -2.17 | Integrated_Ctrl | Autumn | *Bacteroidota* | *Bacteroidia* | *Flavobacteriales* | *Weeksellaceae* | *Chryseobacterium* | *Unclassified* |
| ASV607 | 0.033838 | -2.86 | Integrated_Ctrl | Autumn | *Proteobacteria* | *Alphaproteobacteria* | *Sphingomonadales* | *Sphingomonadaceae* | *Sphingomonas* | *Unclassified* |
| ASV640 | 0.033838 | -0.87 | Integrated_Ctrl | Autumn | *Proteobacteria* | *Gammaproteobacteria* | *Burkholderiales* | *Comamonadaceae* | *Rhizobacter* | *Unclassified* |
| ASV657 | 0.011798 | -2.42 | Integrated_Ctrl | Autumn | *Actinobacteriota* | *Actinobacteria* | *Propionibacteriales* | *Propionibacteriaceae* | *Microlunatus* | *Microlunatus sp.* |
| ASV683 | 0.033838 | -1.46 | Integrated_Ctrl | Autumn | *Chloroflexi* | *Chloroflexia* | *Chloroflexales* | *Roseiflexaceae* | *Unclassified_Roseiflexaceae* | *Unclassified* |
| ASV705 | 0.011798 | -1.84 | Integrated_Ctrl | Autumn | *Proteobacteria* | *Alphaproteobacteria* | *Caulobacterales* | *Caulobacteraceae* | *Phenylobacterium* | *Unclassified* |
| ASV722 | 0.049945 | -1.53 | Integrated_Ctrl | Autumn | *Actinobacteriota* | *Actinobacteria* | *Micrococcales* | *Microbacteriaceae* | *Lysinimonas* | *Unclassified* |
| ASV747 | 0.049945 | -2.88 | Integrated_Ctrl | Autumn | *Proteobacteria* | *Gammaproteobacteria* | *Burkholderiales* | *Comamonadaceae* | *Variovorax* | *Variovorax sp. PAMC 28711* |
| ASV801 | 0.049945 | -6.19 | Integrated_Ctrl | Autumn | *Proteobacteria* | *Gammaproteobacteria* | *Burkholderiales* | *Comamonadaceae* | *Variovorax* | *Variovorax sp. PAMC 28711* |
| ASV818 | 0.049945 | -2.6 | Integrated_Ctrl | Autumn | *Chloroflexi* | *Chloroflexia* | *Chloroflexales* | *Roseiflexaceae* | *Unclassified_Roseiflexaceae* | *Unclassified* |
| ASV819 | 0.049945 | -1 | Integrated_Ctrl | Autumn | *Proteobacteria* | *Gammaproteobacteria* | *Burkholderiales* | *Comamonadaceae* | *Rhizobacter* | *Unclassified* |
| ASV848 | 0.033838 | -2.65 | Integrated_Ctrl | Autumn | *Proteobacteria* | *Gammaproteobacteria* | *Burkholderiales* | *Burkholderiaceae* | *Burkholderia-Caballeronia-Paraburkholderia* | *Unclassified* |
| ASV849 | 0.049945 | -1.61 | Integrated_Ctrl | Autumn | *Proteobacteria* | *Alphaproteobacteria* | *Rhizobiales* | *Xanthobacteraceae* | *Unclassified_Xanthobacteraceae* | *Unclassified* |
| ASV853 | 0.049945 | -3.46 | Integrated_Ctrl | Autumn | *Proteobacteria* | *Alphaproteobacteria* | *Azospirillales* | *Unclassified* | *Unclassified_Azospirillales* | *Unclassified* |
| ASV897 | 0.033838 | -4.2 | Integrated_Ctrl | Autumn | *Proteobacteria* | *Alphaproteobacteria* | *Rhizobiales* | *Rhizobiales Incertae Sedis* | *Nordella* | *Unclassified* |
| ASV909 | 0.033838 | -3.23 | Integrated_Ctrl | Autumn | *Proteobacteria* | *Alphaproteobacteria* | *Rhizobiales* | *Unclassified* | *Unclassified_Rhizobiales* | *Unclassified* |
| ASV259 | 0.015043 | -0.13 | Integrated_Ctrl | Autumn | *Proteobacteria* | *Alphaproteobacteria* | *Rhizobiales* | *Devosiaceae* | *Devosia* | *Devosia neptuniae* |
| ASV329 | 0.011533 | -0.39 | Integrated_Ctrl | Autumn | *Proteobacteria* | *Gammaproteobacteria* | *Burkholderiales* | *Burkholderiaceae* | *Burkholderia-Caballeronia-Paraburkholderia* | *Paraburkholderia hospita* |
| ASV591 | 0.010228 | -0.75 | Integrated_Ctrl | Autumn | *Proteobacteria* | *Alphaproteobacteria* | *Rhizobiales* | *Beijerinckiaceae* | *Bosea* | *Unclassified* |
| ASV631 | 0.03385 | -0.34 | Integrated_Ctrl | Autumn | *Proteobacteria* | *Gammaproteobacteria* | *Burkholderiales* | *Oxalobacteraceae* | *Massilia* | *Unclassified* |
| ASV1319 | 0.010197 | 2.11 | Integrated_Ctrl | Autumn | *Actinobacteriota* | *Thermoleophilia* | *Gaiellales* | *Unclassified* | *Unclassified_Gaiellales* | *Unclassified* |
| ASV672 | 0.033838 | -0.2 | Integrated_Ctrl | Autumn | *Proteobacteria* | *Gammaproteobacteria* | *Burkholderiales* | *Comamonadaceae* | *Pelomonas* | *Unclassified* |
| ASV1803 | 0.010197 | 1.09 | Integrated_Ctrl | Autumn | *Proteobacteria* | *Gammaproteobacteria* | *Burkholderiales* | *Comamonadaceae* | *Giesbergeria* | *Unclassified* |
| ASV291 | 0.035364 | -0.32 | Integrated_Ctrl | Autumn | *Proteobacteria* | *Gammaproteobacteria* | *Legionellales* | *Legionellaceae* | *Legionella* | *Unclassified* |
| ASV323 | 0.02768 | -0.28 | Integrated_Ctrl | Autumn | *Acidobacteriota* | *Blastocatellia* | *Blastocatellales* | *Blastocatellaceae* | *Unclassified_Blastocatellaceae* | *Unclassified* |
| ASV3716 | 0.010197 | 4.6 | Integrated_Ctrl | Autumn | *Chloroflexi* | *Ktedonobacteria* | *Ktedonobacterales* | *Ktedonobacteraceae* | *Unclassified_Ktedonobacteraceae* | *Unclassified* |
| ASV1372 | 9.29E-04 | 16.77 | Integrated_Ctrl | Autumn | *Proteobacteria* | *Gammaproteobacteria* | *Burkholderiales* | *Oxalobacteraceae* | *Unclassified_Oxalobacteraceae* | *Unclassified* |
| ASV1381 | 9.29E-04 | 13.3 | Integrated_Ctrl | Autumn | *Chloroflexi* | *Ktedonobacteria* | *C0119* | *Unclassified* | *Unclassified_C0119* | *Unclassified* |
| ASV1659 | 9.29E-04 | 23.88 | Integrated_Ctrl | Autumn | *Chloroflexi* | *JG30-KF-CM66* | *Unclassified* | *Unclassified* | *Unclassified_JG30-KF-CM66* | *Unclassified* |
| ASV1915 | 0.010197 | 0.68 | Integrated_Ctrl | Autumn | *Proteobacteria* | *Gammaproteobacteria* | *Xanthomonadales* | *Xanthomonadaceae* | *Lysobacter* | *Unclassified* |
| ASV2447 | 0.015556 | 0.6 | Integrated_Ctrl | Autumn | *Proteobacteria* | *Gammaproteobacteria* | *Burkholderiales* | *Oxalobacteraceae* | *Massilia* | *Unclassified* |
| ASV2600 | 0.006625 | 1.1 | Integrated_Ctrl | Autumn | *Actinobacteriota* | *Thermoleophilia* | *Solirubrobacterales* | *Solirubrobacteraceae* | *Conexibacter* | *Unclassified* |
| ASV552 | 0.033117 | 0.08 | Integrated_Ctrl | Autumn | *Actinobacteriota* | *Actinobacteria* | *Micrococcales* | *Microbacteriaceae* | *Lysinimonas* | *Lysinimonas kribbensis* |
| ASV644 | 0.049945 | 0.07 | Integrated_Ctrl | Autumn | *Proteobacteria* | *Gammaproteobacteria* | *Burkholderiales* | *Oxalobacteraceae* | *Massilia* | *Unclassified* |
| ASV820 | 0.006625 | 0.32 | Integrated_Ctrl | Autumn | *Bacteroidota* | *Bacteroidia* | *Chitinophagales* | *Chitinophagaceae* | *Ferruginibacter* | *Unclassified* |
| ASV859 | 0.017226 | 0.13 | Integrated_Ctrl | Autumn | *Proteobacteria* | *Alphaproteobacteria* | *Rhizobiales* | *Xanthobacteraceae* | *Bradyrhizobium* | *Bradyrhizobium sp. CCBAU 43142* |
| ASV986 | 0.010197 | 0.39 | Integrated_Ctrl | Autumn | *WPS-2* | *Unclassified* | *Unclassified* | *Unclassified* | *Unclassified_WPS-2* | *Unclassified* |
| ASV1185 | 9.29E-04 | 38.41 | Integrated_Ctrl | Autumn | *Proteobacteria* | *Gammaproteobacteria* | *Xanthomonadales* | *Xanthomonadaceae* | *Lysobacter* | *Lysobacter sp.* |
| ASV570 | 9.29E-04 | 5257.2 | Integrated_Ctrl | Autumn | *Proteobacteria* | *Alphaproteobacteria* | *Rhizobiales* | *Xanthobacteraceae* | *Rhodopseudomonas* | *Rhodopseudomonas sp.* |
| ASV577 | 0.049945 | 0.08 | Integrated_Ctrl | Autumn | *Acidobacteriota* | *Acidobacteriae* | *Acidobacteriales* | *Acidobacteriaceae (Subgroup 1)* | *Unclassified_Acidobacteriaceae (Subgroup 1)* | *Unclassified* |
| ASV597 | 9.29E-04 | 7.2 | Integrated_Ctrl | Autumn | *Gemmatimonadota* | *Gemmatimonadetes* | *Gemmatimonadales* | *Gemmatimonadaceae* | *Unclassified_Gemmatimonadaceae* | *Unclassified* |
| ASV779 | 0.022859 | 0.22 | Integrated_Ctrl | Autumn | *Bacteroidota* | *Bacteroidia* | *Sphingobacteriales* | *Sphingobacteriaceae* | *Mucilaginibacter* | *Mucilaginibacter sp. OK098* |
| ASV793 | 0.033838 | 0.13 | Integrated_Ctrl | Autumn | *Proteobacteria* | *Alphaproteobacteria* | *Caulobacterales* | *Caulobacteraceae* | *Phenylobacterium* | *Unclassified* |
| ASV850 | 0.007621 | 0.49 | Integrated_Ctrl | Autumn | *Proteobacteria* | *Alphaproteobacteria* | *Rhizobiales* | *Xanthobacteraceae* | *Pseudolabrys* | *Unclassified* |
| ASV856 | 0.014062 | 0.2 | Integrated_Ctrl | Autumn | *Acidobacteriota* | *Blastocatellia* | *Blastocatellales* | *Blastocatellaceae* | *Unclassified_Blastocatellaceae* | *Unclassified* |
| ASV1218 | 0.015941 | 0.23 | Integrated_Ctrl | Autumn | *Proteobacteria* | *Gammaproteobacteria* | *Burkholderiales* | *SC-I-84* | *Unclassified_SC-I-84* | *Unclassified* |
| ASV1274 | 9.29E-04 | 7.88 | Integrated_Ctrl | Autumn | *Proteobacteria* | *Alphaproteobacteria* | *Caulobacterales* | *Caulobacteraceae* | *Asticcacaulis* | *Asticcacaulis biprosthecum C19* |
| ASV1399 | 0.049945 | 0.26 | Integrated_Ctrl | Autumn | *Proteobacteria* | *Alphaproteobacteria* | *Rhizobiales* | *Rhizobiales Incertae Sedis* | *Bauldia* | *Unclassified* |
| ASV365 | 0.006625 | 0.23 | Integrated_Ctrl | Autumn | *Acidobacteriota* | *Acidobacteriae* | *Acidobacteriales* | *Unclassified* | *Unclassified_Acidobacteriales* | *Unclassified* |
| ASV668 | 9.29E-04 | 13.95 | Integrated_Ctrl | Autumn | *Proteobacteria* | *Gammaproteobacteria* | *Xanthomonadales* | *Rhodanobacteraceae* | *Mizugakiibacter* | *Unclassified* |
| ASV1011 | 0.026974 | 0.28 | Integrated_Ctrl | Autumn | *Gemmatimonadota* | *Gemmatimonadetes* | *Gemmatimonadales* | *Gemmatimonadaceae* | *Gemmatimonas* | *Unclassified* |
| ASV1646 | 9.29E-04 | 42.53 | Integrated_Ctrl | Autumn | *Bacteroidota* | *Bacteroidia* | *Cytophagales* | *Microscillaceae* | *Unclassified_Microscillaceae* | *Unclassified* |
| ASV816 | 0.010197 | 0.39 | Integrated_Ctrl | Autumn | *Verrucomicrobiota* | *Verrucomicrobiae* | *Verrucomicrobiales* | *Rubritaleaceae* | *Luteolibacter* | *Unclassified* |
| ASV1040 | 0.010197 | 0.54 | Integrated_Ctrl | Autumn | *Chloroflexi* | *Ktedonobacteria* | *B12-WMSP1* | *Unclassified* | *Unclassified_B12-WMSP1* | *Unclassified* |
| ASV1105 | 0.022723 | 0.24 | Integrated_Ctrl | Autumn | *Acidobacteriota* | *Acidobacteriae* | *Acidobacteriales* | *Unclassified* | *Unclassified_Acidobacteriales* | *Unclassified* |
| ASV215 | 0.010197 | 0.1 | Integrated_Ctrl | Autumn | *Gemmatimonadota* | *Gemmatimonadetes* | *Gemmatimonadales* | *Gemmatimonadaceae* | *Gemmatimonas* | *Unclassified* |
| ASV271 | 0.030863 | 0.07 | Integrated_Ctrl | Autumn | *Proteobacteria* | *Alphaproteobacteria* | *Rhizobiales* | *Xanthobacteraceae* | *Bradyrhizobium* | *Unclassified* |
| ASV283 | 0.035364 | 0.07 | Integrated_Ctrl | Autumn | *Bacteroidota* | *Bacteroidia* | *Sphingobacteriales* | *Sphingobacteriaceae* | *Mucilaginibacter* | *Unclassified* |
| ASV290 | 0.010197 | 0.34 | Integrated_Ctrl | Autumn | *Verrucomicrobiota* | *Verrucomicrobiae* | *Verrucomicrobiales* | *Rubritaleaceae* | *Luteolibacter* | *Unclassified* |
| ASV382 | 9.29E-04 | 6.82 | Integrated_Ctrl | Autumn | *Bacteroidota* | *Bacteroidia* | *Sphingobacteriales* | *Sphingobacteriaceae* | *Mucilaginibacter* | *Unclassified* |
| ASV384 | 0.015556 | 0.1 | Integrated_Ctrl | Autumn | *Proteobacteria* | *Gammaproteobacteria* | *Burkholderiales* | *Oxalobacteraceae* | *Massilia* | *Unclassified* |
| ASV433 | 0.010197 | 0.13 | Integrated_Ctrl | Autumn | *Acidobacteriota* | *Acidobacteriae* | *Acidobacteriales* | *Acidobacteriaceae (Subgroup 1)* | *Granulicella* | *Unclassified* |
| ASV599 | 0.043405 | 0.2 | Integrated_Ctrl | Autumn | *Proteobacteria* | *Gammaproteobacteria* | *Xanthomonadales* | *Rhodanobacteraceae* | *Dokdonella* | *Unclassified* |
| ASV670 | 0.021859 | 0.19 | Integrated_Ctrl | Autumn | *Bacteroidota* | *Bacteroidia* | *Sphingobacteriales* | *Sphingobacteriaceae* | *Mucilaginibacter* | *Mucilaginibacter boryungensis* |
| ASV905 | 0.030999 | 0.2 | Integrated_Ctrl | Autumn | *Proteobacteria* | *Gammaproteobacteria* | *Burkholderiales* | *Oxalobacteraceae* | *Unclassified_Oxalobacteraceae* | *Unclassified* |
| ASV961 | 0.014932 | 0.98 | Integrated_Ctrl | Autumn | *Chloroflexi* | *Ktedonobacteria* | *C0119* | *Unclassified* | *Unclassified_C0119* | *Unclassified* |
| ASV299 | 0.039853 | 0.09 | Integrated_Ctrl | Autumn | *Firmicutes* | *Bacilli* | *Bacillales* | *Bacillaceae* | *Bacillus* | *Unclassified* |
| ASV392 | 0.010762 | 0.18 | Integrated_Ctrl | Autumn | *Acidobacteriota* | *Acidobacteriae* | *Acidobacteriales* | *Acidobacteriaceae (Subgroup 1)* | *Acidipila-Silvibacterium* | *Unclassified* |
| ASV532 | 0.049945 | 0.11 | Integrated_Ctrl | Autumn | *Proteobacteria* | *Alphaproteobacteria* | *Rhizobiales* | *Xanthobacteraceae* | *Rhodopseudomonas* | *Unclassified* |
| ASV687 | 9.29E-04 | 3.46 | Integrated_Ctrl | Autumn | *Myxococcota* | *Polyangia* | *Polyangiales* | *BIrii41* | *Unclassified_BIrii41* | *Unclassified* |
| ASV777 | 0.045614 | 0.22 | Integrated_Ctrl | Autumn | *Acidobacteriota* | *Acidobacteriae* | *Acidobacteriales* | *Acidobacteriaceae (Subgroup 1)* | *Acidipila-Silvibacterium* | *Unclassified* |
| ASV328 | 0.049945 | 0.1 | Integrated_Ctrl | Autumn | *Acidobacteriota* | *Acidobacteriae* | *Subgroup 2* | *Unclassified* | *Unclassified_Subgroup 2* | *Unclassified* |
| ASV721 | 0.021633 | 0.35 | Integrated_Ctrl | Autumn | *Actinobacteriota* | *Actinobacteria* | *Corynebacteriales* | *Nocardiaceae* | *Nocardia* | *Nocardia nova* |
| ASV442 | 0.012523 | 0.15 | Integrated_Ctrl | Autumn | *Bacteroidota* | *Bacteroidia* | *Sphingobacteriales* | *Sphingobacteriaceae* | *Mucilaginibacter* | *Unclassified* |
| ASV148 | 0.011938 | 0.05 | Integrated_Ctrl | Autumn | *Bacteroidota* | *Bacteroidia* | *Chitinophagales* | *Chitinophagaceae* | *Chitinophaga* | *Unclassified* |
| ASV193 | 0.016004 | 0.05 | Integrated_Ctrl | Autumn | *Proteobacteria* | *Gammaproteobacteria* | *Burkholderiales* | *Oxalobacteraceae* | *Duganella* | *Burkholderia sp. Era35* |
| ASV228 | 0.033838 | 0.04 | Integrated_Ctrl | Autumn | *Proteobacteria* | *Gammaproteobacteria* | *Burkholderiales* | *Oxalobacteraceae* | *Duganella* | *Duganella sp.* |
| ASV265 | 0.010197 | 0.12 | Integrated_Ctrl | Autumn | *Acidobacteriota* | *Acidobacteriae* | *Acidobacteriales* | *Acidobacteriaceae (Subgroup 1)* | *Acidipila-Silvibacterium* | *Unclassified* |
| ASV379 | 0.027787 | 0.16 | Integrated_Ctrl | Autumn | *Proteobacteria* | *Alphaproteobacteria* | *Reyranellales* | *Reyranellaceae* | *Reyranella* | *Unclassified* |
| ASV335 | 0.006625 | 0.41 | Integrated_Ctrl | Autumn | *Bacteroidota* | *Bacteroidia* | *Sphingobacteriales* | *Sphingobacteriaceae* | *Mucilaginibacter* | *Unclassified* |
| ASV250 | 0.033838 | -0.19 | Integrated_Ctrl | Autumn | *Proteobacteria* | *Gammaproteobacteria* | *Xanthomonadales* | *Xanthomonadaceae* | *Arenimonas* | *Arenimonas oryziterrae DSM 21050 = YC6267* |
| ASV170 | 0.028463 | -0.11 | Integrated_Ctrl | Autumn | *Bacteroidota* | *Bacteroidia* | *Cytophagales* | *Spirosomaceae* | *Dyadobacter* | *Dyadobacter fermentans* |
| ASV211 | 0.022598 | -0.19 | Integrated_Ctrl | Autumn | *Proteobacteria* | *Gammaproteobacteria* | *Burkholderiales* | *Oxalobacteraceae* | *Massilia* | *Unclassified* |
| ASV169 | 0.040476 | -0.12 | Integrated_Ctrl | Autumn | *Proteobacteria* | *Gammaproteobacteria* | *Burkholderiales* | *Comamonadaceae* | *Acidovorax* | *Acidovorax sp. 1608163* |
| ASV187 | 0.012863 | 0.11 | Integrated_Ctrl | Autumn | *Acidobacteriota* | *Acidobacteriae* | *Acidobacteriales* | *Unclassified* | *Unclassified_Acidobacteriales* | *Unclassified* |
| ASV88 | 0.0092 | 0.05 | Integrated_Ctrl | Autumn | *Bacteroidota* | *Bacteroidia* | *Chitinophagales* | *Chitinophagaceae* | *Chitinophaga* | *Unclassified* |
| ASV146 | 0.034085 | 0.04 | Integrated_Ctrl | Autumn | *Firmicutes* | *Bacilli* | *Paenibacillales* | *Paenibacillaceae* | *Paenibacillus* | *Unclassified* |
| ASV73 | 0.049945 | -0.03 | Integrated_Ctrl | Autumn | *Bacteroidota* | *Bacteroidia* | *Sphingobacteriales* | *Sphingobacteriaceae* | *Pedobacter* | *Pedobacter sp. S8-2* |
| ASV33 | 0.033838 | -0.02 | Integrated_Ctrl | Autumn | *Proteobacteria* | *Alphaproteobacteria* | *Rhizobiales* | *Devosiaceae* | *Devosia* | *Unclassified* |
| ASV44 | 0.036209 | -0.02 | Integrated_Ctrl | Autumn | *Proteobacteria* | *Alphaproteobacteria* | *Rhizobiales* | *Rhizobiaceae* | *Allorhizobium-Neorhizobium-Pararhizobium-Rhizobium* | *Unclassified* |
| ASV24 | 0.009945 | 0.01 | Integrated_Ctrl | Autumn | *Proteobacteria* | *Alphaproteobacteria* | *Caulobacterales* | *Caulobacteraceae* | *Brevundimonas* | *Unclassified* |
| ASV1063 | 0.028388 | 0.47 | Organic_BMs | Autumn | *Proteobacteria* | *Alphaproteobacteria* | *Tistrellales* | *Geminicoccaceae* | *Candidatus Alysiosphaera* | *Unclassified* |
| ASV1118 | 0.010197 | 0.67 | Organic_BMs | Autumn | *Bacteroidota* | *Bacteroidia* | *Flavobacteriales* | *Flavobacteriaceae* | *Flavobacterium* | *Flavobacterium oncorhynchi* |
| ASV1123 | 0.026974 | 0.25 | Organic_BMs | Autumn | *Proteobacteria* | *Gammaproteobacteria* | *Pseudomonadales* | *Pseudomonadaceae* | *Pseudomonas* | *Pseudomonas putida* |
| ASV1159 | 0.020661 | 0.25 | Organic_BMs | Autumn | *Proteobacteria* | *Gammaproteobacteria* | *Xanthomonadales* | *Xanthomonadaceae* | *Stenotrophomonas* | *Unclassified* |
| ASV1457 | 0.049823 | 0.28 | Organic_BMs | Autumn | *Bacteroidota* | *Bacteroidia* | *Sphingobacteriales* | *Sphingobacteriaceae* | *Mucilaginibacter* | *Unclassified* |
| ASV1568 | 0.010197 | 4.49 | Organic_BMs | Autumn | *Bacteroidota* | *Bacteroidia* | *Sphingobacteriales* | *KD3-93* | *Unclassified_KD3-93* | *Unclassified* |
| ASV1576 | 0.011798 | 0.31 | Organic_BMs | Autumn | *Proteobacteria* | *Gammaproteobacteria* | *Pseudomonadales* | *Pseudomonadaceae* | *Pseudomonas* | *Pseudomonas sp. NC02* |
| ASV1985 | 0.007621 | 1.43 | Organic_BMs | Autumn | *Actinobacteriota* | *Actinobacteria* | *Micrococcales* | *Microbacteriaceae* | *Microbacterium* | *Microbacterium kitamiense* |
| ASV2328 | 0.010197 | 2.68 | Organic_BMs | Autumn | *Myxococcota* | *Myxococcia* | *Myxococcales* | *Myxococcaceae* | *Cystobacter* | *Cystobacter ferrugineus* |
| ASV2722 | 0.010197 | 1.99 | Organic_BMs | Autumn | *Bacteroidota* | *Bacteroidia* | *Sphingobacteriales* | *Sphingobacteriaceae* | *Pedobacter* | *Unclassified* |
| ASV312 | 0.008459 | 0.23 | Organic_BMs | Autumn | *Bacteroidota* | *Bacteroidia* | *Chitinophagales* | *Chitinophagaceae* | *Taibaiella* | *Unclassified* |
| ASV350 | 9.29E-04 | 5.06 | Organic_BMs | Autumn | *Proteobacteria* | *Alphaproteobacteria* | *Sphingomonadales* | *Sphingomonadaceae* | *Sphingobium* | *Unclassified* |
| ASV366 | 0.049945 | 0.15 | Organic_BMs | Autumn | *Actinobacteriota* | *Actinobacteria* | *Micrococcales* | *Microbacteriaceae* | *Schumannella* | *Unclassified* |
| ASV3755 | 0.010197 | 4.08 | Organic_BMs | Autumn | *Proteobacteria* | *Alphaproteobacteria* | *Sphingomonadales* | *Sphingomonadaceae* | *Sphingopyxis* | *Sphingopyxis terrae subsp. ummariensis* |
| ASV3934 | 0.010197 | 3.68 | Organic_BMs | Autumn | *Bacteroidota* | *Bacteroidia* | *Flavobacteriales* | *Flavobacteriaceae* | *Flavobacterium* | *Flavobacterium sp. W1.09-205* |
| ASV423 | 9.29E-04 | 32.6 | Organic_BMs | Autumn | *Proteobacteria* | *Gammaproteobacteria* | *Xanthomonadales* | *Xanthomonadaceae* | *Pseudoxanthomonas* | *Pseudoxanthomonas yeongjuensis* |
| ASV431 | 0.006037 | 0.25 | Organic_BMs | Autumn | *Proteobacteria* | *Alphaproteobacteria* | *Rhizobiales* | *Labraceae* | *Labrys* | *Labrys methylaminiphilus* |
| ASV448 | 0.027755 | 0.12 | Organic_BMs | Autumn | *Proteobacteria* | *Alphaproteobacteria* | *Rhizobiales* | *Rhizobiaceae* | *Phyllobacterium* | *Phyllobacterium myrsinacearum* |
| ASV511 | 0.015556 | 0.15 | Organic_BMs | Autumn | *Actinobacteriota* | *Actinobacteria* | *Micrococcales* | *Micrococcaceae* | *Paeniglutamicibacter* | *Arthrobacter sp.* |
| ASV605 | 0.040554 | 0.06 | Organic_BMs | Autumn | *Bacteroidota* | *Bacteroidia* | *Sphingobacteriales* | *Sphingobacteriaceae* | *Pedobacter* | *Unclassified* |
| ASV657 | 0.021633 | 0.43 | Organic_BMs | Autumn | *Actinobacteriota* | *Actinobacteria* | *Propionibacteriales* | *Propionibacteriaceae* | *Microlunatus* | *Microlunatus sp.* |
| ASV658 | 0.011757 | 0.29 | Organic_BMs | Autumn | *Proteobacteria* | *Alphaproteobacteria* | *Rhizobiales* | *Beijerinckiaceae* | *Bosea* | *Bosea sp. TM19_7* |
| ASV666 | 0.010197 | 0.99 | Organic_BMs | Autumn | *Bacteroidota* | *Bacteroidia* | *Sphingobacteriales* | *Sphingobacteriaceae* | *Pedobacter* | *Unclassified* |
| ASV683 | 0.032876 | 0.16 | Organic_BMs | Autumn | *Chloroflexi* | *Chloroflexia* | *Chloroflexales* | *Roseiflexaceae* | *Unclassified_Roseiflexaceae* | *Unclassified* |
| ASV167 | 9.29E-04 | 1.98 | Organic_BMs | Autumn | *Bacteroidota* | *Bacteroidia* | *Chitinophagales* | *Chitinophagaceae* | *Chitinophaga* | *actinobacterium kmd_222* |
| ASV2331 | 0.049945 | -4.49 | Organic_BMs | Autumn | *Actinobacteriota* | *Thermoleophilia* | *Solirubrobacterales* | *Solirubrobacteraceae* | *Conexibacter* | *Unclassified* |
| ASV304 | 0.021633 | 0.09 | Organic_BMs | Autumn | *Bacteroidota* | *Bacteroidia* | *Sphingobacteriales* | *Sphingobacteriaceae* | *Pedobacter* | *Unclassified* |
| ASV354 | 0.016097 | 0.14 | Organic_BMs | Autumn | *Proteobacteria* | *Alphaproteobacteria* | *Sphingomonadales* | *Sphingomonadaceae* | *Sphingopyxis* | *Sphingopyxis taejonensis* |
| ASV575 | 0.021633 | -1.56 | Organic_BMs | Autumn | *Proteobacteria* | *Alphaproteobacteria* | *Acetobacterales* | *Acetobacteraceae* | *Unclassified_Acetobacteraceae* | *Unclassified* |
| ASV878 | 0.049945 | -2.33 | Organic_BMs | Autumn | *Chloroflexi* | *Ktedonobacteria* | *Ktedonobacterales* | *Ktedonobacteraceae* | *Unclassified_Ktedonobacteraceae* | *Unclassified* |
| ASV1169 | 0.049945 | -1.96 | Organic_BMs | Autumn | *Bacteroidota* | *Bacteroidia* | *Chitinophagales* | *Chitinophagaceae* | *Unclassified_Chitinophagaceae* | *Unclassified* |
| ASV415 | 0.033838 | -0.78 | Organic_BMs | Autumn | *Proteobacteria* | *Gammaproteobacteria* | *Burkholderiales* | *Oxalobacteraceae* | *Massilia* | *Unclassified* |
| ASV1915 | 0.049945 | -8.24 | Organic_BMs | Autumn | *Proteobacteria* | *Gammaproteobacteria* | *Xanthomonadales* | *Xanthomonadaceae* | *Lysobacter* | *Unclassified* |
| ASV552 | 0.049945 | -0.74 | Organic_BMs | Autumn | *Actinobacteriota* | *Actinobacteria* | *Micrococcales* | *Microbacteriaceae* | *Lysinimonas* | *Lysinimonas kribbensis* |
| ASV820 | 0.049945 | -1.31 | Organic_BMs | Autumn | *Bacteroidota* | *Bacteroidia* | *Chitinophagales* | *Chitinophagaceae* | *Ferruginibacter* | *Unclassified* |
| ASV1185 | 0.049945 | -2.67 | Organic_BMs | Autumn | *Proteobacteria* | *Gammaproteobacteria* | *Xanthomonadales* | *Xanthomonadaceae* | *Lysobacter* | *Lysobacter sp.* |
| ASV452 | 0.033838 | -1.02 | Organic_BMs | Autumn | *Proteobacteria* | *Alphaproteobacteria* | *Rhizobiales* | *Hyphomicrobiaceae* | *Hyphomicrobium* | *Unclassified* |
| ASV570 | 0.049945 | -0.91 | Organic_BMs | Autumn | *Proteobacteria* | *Alphaproteobacteria* | *Rhizobiales* | *Xanthobacteraceae* | *Rhodopseudomonas* | *Rhodopseudomonas sp.* |
| ASV663 | 0.049945 | -1.13 | Organic_BMs | Autumn | *Proteobacteria* | *Gammaproteobacteria* | *Burkholderiales* | *Oxalobacteraceae* | *Unclassified_Oxalobacteraceae* | *Unclassified* |
| ASV667 | 0.021633 | -2 | Organic_BMs | Autumn | *Proteobacteria* | *Alphaproteobacteria* | *Acetobacterales* | *Acetobacteraceae* | *Unclassified_Acetobacteraceae* | *Unclassified* |
| ASV773 | 0.049945 | -1.27 | Organic_BMs | Autumn | *Proteobacteria* | *Alphaproteobacteria* | *Rhizobiales* | *Devosiaceae* | *Devosia* | *Unclassified* |
| ASV793 | 0.049945 | -1.29 | Organic_BMs | Autumn | *Proteobacteria* | *Alphaproteobacteria* | *Caulobacterales* | *Caulobacteraceae* | *Phenylobacterium* | *Unclassified* |
| ASV935 | 0.033838 | -2.12 | Organic_BMs | Autumn | *Proteobacteria* | *Alphaproteobacteria* | *Rhizobiales* | *Labraceae* | *Labrys* | *Unclassified* |
| ASV965 | 0.033838 | -1.91 | Organic_BMs | Autumn | *Proteobacteria* | *Alphaproteobacteria* | *Rhizobiales* | *Beijerinckiaceae* | *Roseiarcus* | *Unclassified* |
| ASV1274 | 0.033838 | -0.79 | Organic_BMs | Autumn | *Proteobacteria* | *Alphaproteobacteria* | *Caulobacterales* | *Caulobacteraceae* | *Asticcacaulis* | *Asticcacaulis biprosthecum C19* |
| ASV1399 | 0.033838 | -4.15 | Organic_BMs | Autumn | *Proteobacteria* | *Alphaproteobacteria* | *Rhizobiales* | *Rhizobiales Incertae Sedis* | *Bauldia* | *Unclassified* |
| ASV287 | 0.011798 | -0.47 | Organic_BMs | Autumn | *Proteobacteria* | *Gammaproteobacteria* | *Burkholderiales* | *Oxalobacteraceae* | *Massilia* | *Janthinobacterium sp. 286* |
| ASV1646 | 0.039437 | -0.49 | Organic_BMs | Autumn | *Bacteroidota* | *Bacteroidia* | *Cytophagales* | *Microscillaceae* | *Unclassified_Microscillaceae* | *Unclassified* |
| ASV816 | 0.021633 | -3.89 | Organic_BMs | Autumn | *Verrucomicrobiota* | *Verrucomicrobiae* | *Verrucomicrobiales* | *Rubritaleaceae* | *Luteolibacter* | *Unclassified* |
| ASV1040 | 0.021633 | -4.9 | Organic_BMs | Autumn | *Chloroflexi* | *Ktedonobacteria* | *B12-WMSP1* | *Unclassified* | *Unclassified_B12-WMSP1* | *Unclassified* |
| ASV1105 | 0.033838 | -1.65 | Organic_BMs | Autumn | *Acidobacteriota* | *Acidobacteriae* | *Acidobacteriales* | *Unclassified* | *Unclassified_Acidobacteriales* | *Unclassified* |
| ASV271 | 0.033838 | -0.42 | Organic_BMs | Autumn | *Proteobacteria* | *Alphaproteobacteria* | *Rhizobiales* | *Xanthobacteraceae* | *Bradyrhizobium* | *Unclassified* |
| ASV359 | 0.037406 | -0.11 | Organic_BMs | Autumn | *Verrucomicrobiota* | *Verrucomicrobiae* | *Chthoniobacterales* | *Chthoniobacteraceae* | *Chthoniobacter* | *Unclassified* |
| ASV384 | 0.011798 | -8.04 | Organic_BMs | Autumn | *Proteobacteria* | *Gammaproteobacteria* | *Burkholderiales* | *Oxalobacteraceae* | *Massilia* | *Unclassified* |
| ASV433 | 0.026774 | -0.2 | Organic_BMs | Autumn | *Acidobacteriota* | *Acidobacteriae* | *Acidobacteriales* | *Acidobacteriaceae (Subgroup 1)* | *Granulicella* | *Unclassified* |
| ASV627 | 0.033838 | -1.36 | Organic_BMs | Autumn | *Gemmatimonadota* | *Gemmatimonadetes* | *Gemmatimonadales* | *Gemmatimonadaceae* | *Gemmatimonas* | *Unclassified* |
| ASV678 | 0.033838 | -1.14 | Organic_BMs | Autumn | *Verrucomicrobiota* | *Verrucomicrobiae* | *Chthoniobacterales* | *Chthoniobacteraceae* | *Candidatus Udaeobacter* | *Unclassified* |
| ASV795 | 0.021633 | -2.64 | Organic_BMs | Autumn | *Proteobacteria* | *Alphaproteobacteria* | *Rhizobiales* | *Hyphomicrobiaceae* | *Hyphomicrobium* | *Hyphomicrobium denitrificans 1NES1* |
| ASV803 | 0.011798 | -2.94 | Organic_BMs | Autumn | *Bacteroidota* | *Bacteroidia* | *Sphingobacteriales* | *Sphingobacteriaceae* | *Mucilaginibacter* | *Unclassified* |
| ASV905 | 0.021633 | -2.53 | Organic_BMs | Autumn | *Proteobacteria* | *Gammaproteobacteria* | *Burkholderiales* | *Oxalobacteraceae* | *Unclassified_Oxalobacteraceae* | *Unclassified* |
| ASV927 | 0.021633 | -24.15 | Organic_BMs | Autumn | *Actinobacteriota* | *Actinobacteria* | *Micrococcales* | *Intrasporangiaceae* | *Unclassified_Intrasporangiaceae* | *Unclassified* |
| ASV961 | 0.035364 | -0.49 | Organic_BMs | Autumn | *Chloroflexi* | *Ktedonobacteria* | *C0119* | *Unclassified* | *Unclassified_C0119* | *Unclassified* |
| ASV299 | 0.021633 | -1.53 | Organic_BMs | Autumn | *Firmicutes* | *Bacilli* | *Bacillales* | *Bacillaceae* | *Bacillus* | *Unclassified* |
| ASV392 | 0.049945 | -0.13 | Organic_BMs | Autumn | *Acidobacteriota* | *Acidobacteriae* | *Acidobacteriales* | *Acidobacteriaceae (Subgroup 1)* | *Acidipila-Silvibacterium* | *Unclassified* |
| ASV482 | 0.049945 | -0.2 | Organic_BMs | Autumn | *Bacteroidota* | *Bacteroidia* | *Chitinophagales* | *Chitinophagaceae* | *Unclassified_Chitinophagaceae* | *Unclassified* |
| ASV532 | 0.022598 | -0.3 | Organic_BMs | Autumn | *Proteobacteria* | *Alphaproteobacteria* | *Rhizobiales* | *Xanthobacteraceae* | *Rhodopseudomonas* | *Unclassified* |
| ASV687 | 0.036337 | -0.22 | Organic_BMs | Autumn | *Myxococcota* | *Polyangia* | *Polyangiales* | *BIrii41* | *Unclassified_BIrii41* | *Unclassified* |
| ASV756 | 0.021633 | -1.09 | Organic_BMs | Autumn | *Proteobacteria* | *Alphaproteobacteria* | *Holosporales* | *Holosporaceae* | *Unclassified_Holosporaceae* | *Unclassified* |
| ASV777 | 0.011798 | -3.76 | Organic_BMs | Autumn | *Acidobacteriota* | *Acidobacteriae* | *Acidobacteriales* | *Acidobacteriaceae (Subgroup 1)* | *Acidipila-Silvibacterium* | *Unclassified* |
| ASV923 | 0.021633 | -3.13 | Organic_BMs | Autumn | *Proteobacteria* | *Alphaproteobacteria* | *Rhizobiales* | *Xanthobacteraceae* | *Bradyrhizobium* | *Bradyrhizobium sp. ICMP 14754* |
| ASV403 | 0.017919 | -0.24 | Organic_BMs | Autumn | *Proteobacteria* | *Alphaproteobacteria* | *Sphingomonadales* | *Sphingomonadaceae* | *Sphingomonas* | *Unclassified* |
| ASV721 | 0.021859 | -0.52 | Organic_BMs | Autumn | *Actinobacteriota* | *Actinobacteria* | *Corynebacteriales* | *Nocardiaceae* | *Nocardia* | *Nocardia nova* |
| ASV442 | 0.029568 | -0.15 | Organic_BMs | Autumn | *Bacteroidota* | *Bacteroidia* | *Sphingobacteriales* | *Sphingobacteriaceae* | *Mucilaginibacter* | *Unclassified* |
| ASV148 | 0.015784 | -0.11 | Organic_BMs | Autumn | *Bacteroidota* | *Bacteroidia* | *Chitinophagales* | *Chitinophagaceae* | *Chitinophaga* | *Unclassified* |
| ASV193 | 0.015334 | -0.1 | Organic_BMs | Autumn | *Proteobacteria* | *Gammaproteobacteria* | *Burkholderiales* | *Oxalobacteraceae* | *Duganella* | *Burkholderia sp. Era35* |
| ASV228 | 0.018099 | -0.1 | Organic_BMs | Autumn | *Proteobacteria* | *Gammaproteobacteria* | *Burkholderiales* | *Oxalobacteraceae* | *Duganella* | *Duganella sp.* |
| ASV265 | 0.009665 | -0.85 | Organic_BMs | Autumn | *Acidobacteriota* | *Acidobacteriae* | *Acidobacteriales* | *Acidobacteriaceae (Subgroup 1)* | *Acidipila-Silvibacterium* | *Unclassified* |
| ASV173 | 0.033838 | -0.06 | Organic_BMs | Autumn | *Proteobacteria* | *Gammaproteobacteria* | *Burkholderiales* | *Oxalobacteraceae* | *Duganella* | *Burkholderia sp. Era35* |
| ASV335 | 0.042275 | -0.11 | Organic_BMs | Autumn | *Bacteroidota* | *Bacteroidia* | *Sphingobacteriales* | *Sphingobacteriaceae* | *Mucilaginibacter* | *Unclassified* |
| ASV95 | 0.007621 | 0.19 | Organic_BMs | Autumn | *Bacteroidota* | *Bacteroidia* | *Sphingobacteriales* | *Sphingobacteriaceae* | *Pedobacter* | *Unclassified* |
| ASV170 | 0.04154 | 0.03 | Organic_BMs | Autumn | *Bacteroidota* | *Bacteroidia* | *Cytophagales* | *Spirosomaceae* | *Dyadobacter* | *Dyadobacter fermentans* |
| ASV187 | 0.049945 | -0.09 | Organic_BMs | Autumn | *Acidobacteriota* | *Acidobacteriae* | *Acidobacteriales* | *Unclassified* | *Unclassified_Acidobacteriales* | *Unclassified* |
| ASV88 | 0.014938 | -0.08 | Organic_BMs | Autumn | *Bacteroidota* | *Bacteroidia* | *Chitinophagales* | *Chitinophagaceae* | *Chitinophaga* | *Unclassified* |
| ASV146 | 0.009079 | -0.13 | Organic_BMs | Autumn | *Firmicutes* | *Bacilli* | *Paenibacillales* | *Paenibacillaceae* | *Paenibacillus* | *Unclassified* |
| ASV52 | 0.048393 | 0.01 | Organic_BMs | Autumn | *Proteobacteria* | *Alphaproteobacteria* | *Sphingomonadales* | *Sphingomonadaceae* | *Sphingobium* | *Sphingobium sp. PDD-35b-9* |
| ASV63 | 0.045838 | -0.04 | Organic_BMs | Autumn | *Actinobacteriota* | *Actinobacteria* | *Micrococcales* | *Microbacteriaceae* | *Unclassified_Microbacteriaceae* | *Unclassified* |
| ASV24 | 0.049945 | -0.01 | Organic_BMs | Autumn | *Proteobacteria* | *Alphaproteobacteria* | *Caulobacterales* | *Caulobacteraceae* | *Brevundimonas* | *Unclassified* |
| ASV1063 | 0.021633 | -4.65 | Organic_Ctrl | Autumn | *Proteobacteria* | *Alphaproteobacteria* | *Tistrellales* | *Geminicoccaceae* | *Candidatus Alysiosphaera* | *Unclassified* |
| ASV1082 | 0.035364 | 0.11 | Organic_Ctrl | Autumn | *Proteobacteria* | *Gammaproteobacteria* | *Burkholderiales* | *Comamonadaceae* | *Rhizobacter* | *Unclassified* |
| ASV1144 | 0.020661 | 0.34 | Organic_Ctrl | Autumn | *Proteobacteria* | *Gammaproteobacteria* | *Xanthomonadales* | *Xanthomonadaceae* | *Lysobacter* | *Unclassified* |
| ASV1180 | 0.049945 | 0.24 | Organic_Ctrl | Autumn | *Proteobacteria* | *Gammaproteobacteria* | *Xanthomonadales* | *Xanthomonadaceae* | *Lysobacter* | *Lysobacter sp. cf310* |
| ASV11877 | 0.039437 | 26.64 | Organic_Ctrl | Autumn | *Proteobacteria* | *Gammaproteobacteria* | *Steroidobacterales* | *Steroidobacteraceae* | *Steroidobacter* | *Unclassified* |
| ASV1201 | 0.010197 | 0.39 | Organic_Ctrl | Autumn | *Chloroflexi* | *Chloroflexia* | *Chloroflexales* | *Herpetosiphonaceae* | *Herpetosiphon* | *Unclassified* |
| ASV1316 | 0.049945 | 0.25 | Organic_Ctrl | Autumn | *Bacteroidota* | *Bacteroidia* | *Chitinophagales* | *Chitinophagaceae* | *Pseudoflavitalea* | *Unclassified* |
| ASV1344 | 0.049945 | 0.12 | Organic_Ctrl | Autumn | *Proteobacteria* | *Gammaproteobacteria* | *Burkholderiales* | *Oxalobacteraceae* | *Massilia* | *Massilia sp. OS322* |
| ASV1378 | 0.010197 | 2.66 | Organic_Ctrl | Autumn | *Proteobacteria* | *Alphaproteobacteria* | *Elsterales* | *Unclassified* | *Unclassified_Elsterales* | *Unclassified* |
| ASV1402 | 0.010197 | 3.2 | Organic_Ctrl | Autumn | *Gemmatimonadota* | *Gemmatimonadetes* | *Gemmatimonadales* | *Gemmatimonadaceae* | *Unclassified_Gemmatimonadaceae* | *Unclassified* |
| ASV1465 | 0.043035 | 0.22 | Organic_Ctrl | Autumn | *Proteobacteria* | *Gammaproteobacteria* | *Burkholderiales* | *Comamonadaceae* | *Unclassified_Comamonadaceae* | *Unclassified* |
| ASV1466 | 0.010197 | 0.66 | Organic_Ctrl | Autumn | *Actinobacteriota* | *Actinobacteria* | *Pseudonocardiales* | *Pseudonocardiaceae* | *Pseudonocardia* | *Unclassified* |
| ASV1497 | 0.014091 | 0.36 | Organic_Ctrl | Autumn | *Chloroflexi* | *Ktedonobacteria* | *C0119* | *Unclassified* | *Unclassified_C0119* | *Unclassified* |
| ASV1672 | 0.043837 | 0.2 | Organic_Ctrl | Autumn | *Proteobacteria* | *Gammaproteobacteria* | *Burkholderiales* | *Oxalobacteraceae* | *Actimicrobium* | *Unclassified* |
| ASV1711 | 0.010197 | 2.38 | Organic_Ctrl | Autumn | *Chloroflexi* | *Chloroflexia* | *Thermomicrobiales* | *JG30-KF-CM45* | *Unclassified_JG30-KF-CM45* | *Unclassified* |
| ASV1818 | 0.037406 | 0.3 | Organic_Ctrl | Autumn | *Bacteroidota* | *Bacteroidia* | *Cytophagales* | *Spirosomaceae* | *Dyadobacter* | *Unclassified* |
| ASV2194 | 0.010197 | 1.46 | Organic_Ctrl | Autumn | *Proteobacteria* | *Gammaproteobacteria* | *Burkholderiales* | *Comamonadaceae* | *Variovorax* | *Variovorax sp.* |
| ASV2234 | 0.024382 | 0.53 | Organic_Ctrl | Autumn | *Proteobacteria* | *Gammaproteobacteria* | *Xanthomonadales* | *Rhodanobacteraceae* | *Dokdonella* | *Unclassified* |
| ASV2256 | 0.010197 | 2.01 | Organic_Ctrl | Autumn | *Bacteroidota* | *Bacteroidia* | *Cytophagales* | *Microscillaceae* | *Unclassified_Microscillaceae* | *Unclassified* |
| ASV2276 | 0.049945 | -5.68 | Organic_Ctrl | Autumn | *Abditibacteriota* | *Abditibacteria* | *Abditibacteriales* | *Abditibacteriaceae* | *Abditibacterium* | *Unclassified* |
| ASV2715 | 0.010184 | 1.18 | Organic_Ctrl | Autumn | *Proteobacteria* | *Gammaproteobacteria* | *Burkholderiales* | *Oxalobacteraceae* | *Massilia* | *Unclassified* |
| ASV2813 | 0.026974 | 0.61 | Organic_Ctrl | Autumn | *Cyanobacteria* | *Sericytochromatia* | *Unclassified* | *Unclassified* | *Unclassified_Sericytochromatia* | *Unclassified* |
| ASV2817 | 0.010197 | 3.62 | Organic_Ctrl | Autumn | *Proteobacteria* | *Gammaproteobacteria* | *Burkholderiales* | *Oxalobacteraceae* | *Noviherbaspirillum* | *Unclassified* |
| ASV300 | 0.033838 | -1.95 | Organic_Ctrl | Autumn | *Bacteroidota* | *Bacteroidia* | *Chitinophagales* | *Chitinophagaceae* | *Chitinophaga* | *actinobacterium kmd_222* |
| ASV310 | 0.049945 | 0.08 | Organic_Ctrl | Autumn | *Proteobacteria* | *Alphaproteobacteria* | *Rhizobiales* | *Xanthobacteraceae* | *Afipia* | *Afipia sp. 9(2017)* |
| ASV317 | 0.037782 | 0.04 | Organic_Ctrl | Autumn | *Proteobacteria* | *Alphaproteobacteria* | *Sphingomonadales* | *Sphingomonadaceae* | *Sphingomonas* | *Sphingomonas sp. TSK-1* |
| ASV400 | 0.032875 | 0.08 | Organic_Ctrl | Autumn | *Bacteroidota* | *Bacteroidia* | *Sphingobacteriales* | *Sphingobacteriaceae* | *Pedobacter* | *Unclassified* |
| ASV4095 | 0.010197 | 3.5 | Organic_Ctrl | Autumn | *Bacteroidota* | *Bacteroidia* | *Sphingobacteriales* | *Sphingobacteriaceae* | *Pedobacter* | *Unclassified* |
| ASV466 | 0.049945 | -0.77 | Organic_Ctrl | Autumn | *Proteobacteria* | *Alphaproteobacteria* | *Sphingomonadales* | *Sphingomonadaceae* | *Sphingopyxis* | *Sphingopyxis sp. QXT-31* |
| ASV468 | 0.026131 | 0.12 | Organic_Ctrl | Autumn | *Bacteroidota* | *Bacteroidia* | *Chitinophagales* | *Chitinophagaceae* | *Terrimonas* | *Unclassified* |
| ASV486 | 0.035233 | 0.06 | Organic_Ctrl | Autumn | *Proteobacteria* | *Alphaproteobacteria* | *Rhizobiales* | *Devosiaceae* | *Devosia* | *Unclassified* |
| ASV523 | 0.010197 | 0.89 | Organic_Ctrl | Autumn | *Actinobacteriota* | *Actinobacteria* | *Streptomycetales* | *Streptomycetaceae* | *Streptomyces* | *Streptomyces albus* |
| ASV554 | 0.004646 | 1.14 | Organic_Ctrl | Autumn | *Proteobacteria* | *Gammaproteobacteria* | *Burkholderiales* | *Comamonadaceae* | *Rhizobacter* | *Unclassified* |
| ASV593 | 0.049945 | -1.82 | Organic_Ctrl | Autumn | *Actinobacteriota* | *Actinobacteria* | *Streptomycetales* | *Streptomycetaceae* | *Kitasatospora* | *Kitasatospora gansuensis* |
| ASV598 | 0.010197 | 0.3 | Organic_Ctrl | Autumn | *Proteobacteria* | *Gammaproteobacteria* | *Xanthomonadales* | *Rhodanobacteraceae* | *Tahibacter* | *Unclassified* |
| ASV640 | 9.29E-04 | 4.77 | Organic_Ctrl | Autumn | *Proteobacteria* | *Gammaproteobacteria* | *Burkholderiales* | *Comamonadaceae* | *Rhizobacter* | *Unclassified* |
| ASV656 | 0.010197 | 0.37 | Organic_Ctrl | Autumn | *Proteobacteria* | *Alphaproteobacteria* | *Rhizobiales* | *Xanthobacteraceae* | *Pseudolabrys* | *Unclassified* |
| ASV783 | 9.29E-04 | 1.32 | Organic_Ctrl | Autumn | *Bacteroidota* | *Bacteroidia* | *Sphingobacteriales* | *Sphingobacteriaceae* | *Mucilaginibacter* | *Mucilaginibacter puniceus* |
| ASV818 | 0.010197 | 0.18 | Organic_Ctrl | Autumn | *Chloroflexi* | *Chloroflexia* | *Chloroflexales* | *Roseiflexaceae* | *Unclassified_Roseiflexaceae* | *Unclassified* |
| ASV819 | 0.022598 | 0.12 | Organic_Ctrl | Autumn | *Proteobacteria* | *Gammaproteobacteria* | *Burkholderiales* | *Comamonadaceae* | *Rhizobacter* | *Unclassified* |
| ASV822 | 0.009945 | 0.5 | Organic_Ctrl | Autumn | *Proteobacteria* | *Gammaproteobacteria* | *Burkholderiales* | *Comamonadaceae* | *Rhodoferax* | *beta proteobacterium HTCC541* |
| ASV853 | 0.009945 | 0.62 | Organic_Ctrl | Autumn | *Proteobacteria* | *Alphaproteobacteria* | *Azospirillales* | *Unclassified* | *Unclassified_Azospirillales* | *Unclassified* |
| ASV909 | 0.015831 | 0.34 | Organic_Ctrl | Autumn | *Proteobacteria* | *Alphaproteobacteria* | *Rhizobiales* | *Unclassified* | *Unclassified_Rhizobiales* | *Unclassified* |
| ASV916 | 0.010197 | 1.46 | Organic_Ctrl | Autumn | *Proteobacteria* | *Alphaproteobacteria* | *Rhodobacterales* | *Rhodobacteraceae* | *Pseudorhodobacter* | *Unclassified* |
| ASV958 | 0.010464 | 1.97 | Organic_Ctrl | Autumn | *Proteobacteria* | *Gammaproteobacteria* | *Xanthomonadales* | *Xanthomonadaceae* | *Arenimonas* | *Unclassified* |
| ASV994 | 0.010197 | 0.2 | Organic_Ctrl | Autumn | *Chloroflexi* | *Chloroflexia* | *Chloroflexales* | *Roseiflexaceae* | *Unclassified_Roseiflexaceae* | *Unclassified* |
| ASV167 | 0.037476 | -0.13 | Organic_Ctrl | Autumn | *Bacteroidota* | *Bacteroidia* | *Chitinophagales* | *Chitinophagaceae* | *Chitinophaga* | *actinobacterium kmd_222* |
| ASV235 | 0.018788 | -0.26 | Organic_Ctrl | Autumn | *Bacteroidota* | *Bacteroidia* | *Sphingobacteriales* | *Sphingobacteriaceae* | *Pedobacter* | *Pedobacter panaciterrae* |
| ASV354 | 0.049945 | -2.23 | Organic_Ctrl | Autumn | *Proteobacteria* | *Alphaproteobacteria* | *Sphingomonadales* | *Sphingomonadaceae* | *Sphingopyxis* | *Sphingopyxis taejonensis* |
| ASV489 | 0.049945 | -1.54 | Organic_Ctrl | Autumn | *Bacteroidota* | *Bacteroidia* | *Sphingobacteriales* | *Sphingobacteriaceae* | *Pedobacter* | *Unclassified* |
| ASV878 | 0.049945 | -2.33 | Organic_Ctrl | Autumn | *Chloroflexi* | *Ktedonobacteria* | *Ktedonobacterales* | *Ktedonobacteraceae* | *Unclassified_Ktedonobacteraceae* | *Unclassified* |
| ASV1169 | 0.049945 | -1.96 | Organic_Ctrl | Autumn | *Bacteroidota* | *Bacteroidia* | *Chitinophagales* | *Chitinophagaceae* | *Unclassified_Chitinophagaceae* | *Unclassified* |
| ASV1723 | 0.033838 | -6.86 | Organic_Ctrl | Autumn | *Proteobacteria* | *Alphaproteobacteria* | *Rickettsiales* | *Candidatus Jidaibacter* | *Unclassified_Candidatus Jidaibacter* | *Unclassified* |
| ASV672 | 9.29E-04 | 3.75 | Organic_Ctrl | Autumn | *Proteobacteria* | *Gammaproteobacteria* | *Burkholderiales* | *Comamonadaceae* | *Pelomonas* | *Unclassified* |
| ASV723 | 0.022332 | 0.18 | Organic_Ctrl | Autumn | *Myxococcota* | *Polyangia* | *Polyangiales* | *BIrii41* | *Unclassified_BIrii41* | *Unclassified* |
| ASV221 | 9.29E-04 | 3.42 | Organic_Ctrl | Autumn | *Proteobacteria* | *Gammaproteobacteria* | *Burkholderiales* | *Comamonadaceae* | *Rhodoferax* | *Unclassified* |
| ASV291 | 9.29E-04 | 9.56 | Organic_Ctrl | Autumn | *Proteobacteria* | *Gammaproteobacteria* | *Legionellales* | *Legionellaceae* | *Legionella* | *Unclassified* |
| ASV323 | 0.03332 | 0.1 | Organic_Ctrl | Autumn | *Acidobacteriota* | *Blastocatellia* | *Blastocatellales* | *Blastocatellaceae* | *Unclassified_Blastocatellaceae* | *Unclassified* |
| ASV163 | 0.028634 | 0.03 | Organic_Ctrl | Autumn | *Proteobacteria* | *Alphaproteobacteria* | *Rhizobiales* | *Devosiaceae* | *Devosia* | *Unclassified* |
| ASV2447 | 0.049945 | -17.87 | Organic_Ctrl | Autumn | *Proteobacteria* | *Gammaproteobacteria* | *Burkholderiales* | *Oxalobacteraceae* | *Massilia* | *Unclassified* |
| ASV552 | 0.049945 | -0.74 | Organic_Ctrl | Autumn | *Actinobacteriota* | *Actinobacteria* | *Micrococcales* | *Microbacteriaceae* | *Lysinimonas* | *Lysinimonas kribbensis* |
| ASV644 | 0.033838 | -7.06 | Organic_Ctrl | Autumn | *Proteobacteria* | *Gammaproteobacteria* | *Burkholderiales* | *Oxalobacteraceae* | *Massilia* | *Unclassified* |
| ASV1185 | 0.049945 | -2.67 | Organic_Ctrl | Autumn | *Proteobacteria* | *Gammaproteobacteria* | *Xanthomonadales* | *Xanthomonadaceae* | *Lysobacter* | *Lysobacter sp.* |
| ASV570 | 0.049945 | -0.91 | Organic_Ctrl | Autumn | *Proteobacteria* | *Alphaproteobacteria* | *Rhizobiales* | *Xanthobacteraceae* | *Rhodopseudomonas* | *Rhodopseudomonas sp.* |
| ASV577 | 0.033838 | -2.02 | Organic_Ctrl | Autumn | *Acidobacteriota* | *Acidobacteriae* | *Acidobacteriales* | *Acidobacteriaceae (Subgroup 1)* | *Unclassified_Acidobacteriaceae (Subgroup 1)* | *Unclassified* |
| ASV597 | 0.049945 | -1.06 | Organic_Ctrl | Autumn | *Gemmatimonadota* | *Gemmatimonadetes* | *Gemmatimonadales* | *Gemmatimonadaceae* | *Unclassified_Gemmatimonadaceae* | *Unclassified* |
| ASV663 | 0.049945 | -1.13 | Organic_Ctrl | Autumn | *Proteobacteria* | *Gammaproteobacteria* | *Burkholderiales* | *Oxalobacteraceae* | *Unclassified_Oxalobacteraceae* | *Unclassified* |
| ASV773 | 0.049945 | -1.27 | Organic_Ctrl | Autumn | *Proteobacteria* | *Alphaproteobacteria* | *Rhizobiales* | *Devosiaceae* | *Devosia* | *Unclassified* |
| ASV779 | 0.033838 | -2.46 | Organic_Ctrl | Autumn | *Bacteroidota* | *Bacteroidia* | *Sphingobacteriales* | *Sphingobacteriaceae* | *Mucilaginibacter* | *Mucilaginibacter sp. OK098* |
| ASV856 | 0.049945 | -5.58 | Organic_Ctrl | Autumn | *Acidobacteriota* | *Blastocatellia* | *Blastocatellales* | *Blastocatellaceae* | *Unclassified_Blastocatellaceae* | *Unclassified* |
| ASV133 | 0.010197 | 0.05 | Organic_Ctrl | Autumn | *Proteobacteria* | *Gammaproteobacteria* | *Burkholderiales* | *Oxalobacteraceae* | *Massilia* | *Unclassified* |
| ASV365 | 0.021633 | -1.63 | Organic_Ctrl | Autumn | *Acidobacteriota* | *Acidobacteriae* | *Acidobacteriales* | *Unclassified* | *Unclassified_Acidobacteriales* | *Unclassified* |
| ASV702 | 0.010197 | 0.55 | Organic_Ctrl | Autumn | *Bacteroidota* | *Bacteroidia* | *Chitinophagales* | *Chitinophagaceae* | *Unclassified_Chitinophagaceae* | *Unclassified* |
| ASV1011 | 0.033838 | -3.04 | Organic_Ctrl | Autumn | *Gemmatimonadota* | *Gemmatimonadetes* | *Gemmatimonadales* | *Gemmatimonadaceae* | *Gemmatimonas* | *Unclassified* |
| ASV1105 | 0.033838 | -1.65 | Organic_Ctrl | Autumn | *Acidobacteriota* | *Acidobacteriae* | *Acidobacteriales* | *Unclassified* | *Unclassified_Acidobacteriales* | *Unclassified* |
| ASV1519 | 0.021633 | -5.4 | Organic_Ctrl | Autumn | *Proteobacteria* | *Gammaproteobacteria* | *Xanthomonadales* | *Rhodanobacteraceae* | *Rhodanobacter* | *Unclassified* |
| ASV159 | 9.29E-04 | -5.22 | Organic_Ctrl | Autumn | *Actinobacteriota* | *Actinobacteria* | *Streptomycetales* | *Streptomycetaceae* | *Kitasatospora* | *Kitasatospora sp. MBT63* |
| ASV215 | 0.020661 | -0.11 | Organic_Ctrl | Autumn | *Gemmatimonadota* | *Gemmatimonadetes* | *Gemmatimonadales* | *Gemmatimonadaceae* | *Gemmatimonas* | *Unclassified* |
| ASV220 | 0.018565 | -0.24 | Organic_Ctrl | Autumn | *Bacteroidota* | *Bacteroidia* | *Cytophagales* | *Spirosomaceae* | *Dyadobacter* | *Unclassified* |
| ASV271 | 0.033838 | -0.42 | Organic_Ctrl | Autumn | *Proteobacteria* | *Alphaproteobacteria* | *Rhizobiales* | *Xanthobacteraceae* | *Bradyrhizobium* | *Unclassified* |
| ASV384 | 0.049945 | -0.08 | Organic_Ctrl | Autumn | *Proteobacteria* | *Gammaproteobacteria* | *Burkholderiales* | *Oxalobacteraceae* | *Massilia* | *Unclassified* |
| ASV425 | 0.018155 | -0.25 | Organic_Ctrl | Autumn | *Proteobacteria* | *Gammaproteobacteria* | *Burkholderiales* | *Oxalobacteraceae* | *Herbaspirillum* | *Herbaspirillum hiltneri* |
| ASV595 | 0.028388 | -0.28 | Organic_Ctrl | Autumn | *Acidobacteriota* | *Acidobacteriae* | *Bryobacterales* | *Bryobacteraceae* | *Bryobacter* | *Unclassified* |
| ASV599 | 0.043035 | -0.23 | Organic_Ctrl | Autumn | *Proteobacteria* | *Gammaproteobacteria* | *Xanthomonadales* | *Rhodanobacteraceae* | *Dokdonella* | *Unclassified* |
| ASV627 | 0.033838 | -1.36 | Organic_Ctrl | Autumn | *Gemmatimonadota* | *Gemmatimonadetes* | *Gemmatimonadales* | *Gemmatimonadaceae* | *Gemmatimonas* | *Unclassified* |
| ASV670 | 0.030874 | -0.25 | Organic_Ctrl | Autumn | *Bacteroidota* | *Bacteroidia* | *Sphingobacteriales* | *Sphingobacteriaceae* | *Mucilaginibacter* | *Mucilaginibacter boryungensis* |
| ASV678 | 0.033838 | -1.14 | Organic_Ctrl | Autumn | *Verrucomicrobiota* | *Verrucomicrobiae* | *Chthoniobacterales* | *Chthoniobacteraceae* | *Candidatus Udaeobacter* | *Unclassified* |
| ASV328 | 0.033838 | -0.17 | Organic_Ctrl | Autumn | *Acidobacteriota* | *Acidobacteriae* | *Subgroup 2* | *Unclassified* | *Unclassified_Subgroup 2* | *Unclassified* |
| ASV40 | 0.016004 | -0.03 | Organic_Ctrl | Autumn | *Bacteroidota* | *Bacteroidia* | *Sphingobacteriales* | *Sphingobacteriaceae* | *Pedobacter* | *Pedobacter sp. S8-2* |
| ASV298 | 0.010197 | -0.52 | Organic_Ctrl | Autumn | *Proteobacteria* | *Alphaproteobacteria* | *Sphingomonadales* | *Sphingomonadaceae* | *Sphingomonas* | *Unclassified* |
| ASV218 | 0.021633 | -0.22 | Organic_Ctrl | Autumn | *Bacteroidota* | *Bacteroidia* | *Sphingobacteriales* | *Sphingobacteriaceae* | *Pedobacter* | *Beta vulgaris subsp. vulgaris* |
| ASV243 | 0.012274 | 0.12 | Organic_Ctrl | Autumn | *Bacteroidota* | *Bacteroidia* | *Chitinophagales* | *Chitinophagaceae* | *Ferruginibacter* | *Unclassified* |
| ASV156 | 0.03407 | -0.06 | Organic_Ctrl | Autumn | *Bacteroidota* | *Bacteroidia* | *Cytophagales* | *Spirosomaceae* | *Dyadobacter* | *Dyadobacter fermentans* |
| ASV187 | 0.033838 | -0.13 | Organic_Ctrl | Autumn | *Acidobacteriota* | *Acidobacteriae* | *Acidobacteriales* | *Unclassified* | *Unclassified_Acidobacteriales* | *Unclassified* |
| ASV157 | 0.021633 | 0.04 | Organic_Ctrl | Autumn | *Proteobacteria* | *Gammaproteobacteria* | *Burkholderiales* | *Comamonadaceae* | *Limnohabitans* | *Unclassified* |
| ASV191 | 0.021974 | -0.18 | Organic_Ctrl | Autumn | *Firmicutes* | *Bacilli* | *Paenibacillales* | *Paenibacillaceae* | *Paenibacillus* | *Unclassified* |
| ASV178 | 0.016497 | -0.39 | Organic_Ctrl | Autumn | *Proteobacteria* | *Alphaproteobacteria* | *Caulobacterales* | *Caulobacteraceae* | *Asticcacaulis* | *Unclassified* |
| ASV106 | 0.028634 | 0.02 | Organic_Ctrl | Autumn | *Proteobacteria* | *Alphaproteobacteria* | *Rhizobiales* | *Stappiaceae* | *Stappia* | *Unclassified* |
| ASV103 | 0.049945 | 0.03 | Organic_Ctrl | Autumn | *Proteobacteria* | *Alphaproteobacteria* | *Sphingomonadales* | *Sphingomonadaceae* | *Sphingomonas* | *Unclassified* |
| ASV94 | 0.033838 | 0.02 | Organic_Ctrl | Autumn | *Proteobacteria* | *Alphaproteobacteria* | *Rhizobiales* | *Rhizobiaceae* | *Allorhizobium-Neorhizobium-Pararhizobium-Rhizobium* | *Pararhizobium giardinii* |
| ASV78 | 0.018565 | -0.18 | Organic_Ctrl | Autumn | *Bacteroidota* | *Bacteroidia* | *Sphingobacteriales* | *Sphingobacteriaceae* | *Pedobacter* | *Pedobacter insulae* |
| ASV30 | 0.049823 | 0.01 | Organic_Ctrl | Autumn | *Bacteroidota* | *Bacteroidia* | *Flavobacteriales* | *Flavobacteriaceae* | *Flavobacterium* | *Flavobacterium sp. Amb3* |
| ASV72 | 0.013156 | -0.12 | Organic_Ctrl | Autumn | *Firmicutes* | *Bacilli* | *Bacillales* | *Bacillaceae* | *Bacillus* | *Unclassified* |
| ASV96 | 0.03875 | -0.06 | Organic_Ctrl | Autumn | *Proteobacteria* | *Alphaproteobacteria* | *Rhizobiales* | *Devosiaceae* | *Devosia* | *bacterium enrichment culture clone heteroA15_4W* |
| ASV60 | 0.049945 | 0.01 | Organic_Ctrl | Autumn | *Proteobacteria* | *Alphaproteobacteria* | *Rhizobiales* | *Rhizobiaceae* | *Allorhizobium-Neorhizobium-Pararhizobium-Rhizobium* | *Unclassified* |
| ASV61 | 0.016222 | -0.19 | Organic_Ctrl | Autumn | *Proteobacteria* | *Gammaproteobacteria* | *Xanthomonadales* | *Rhodanobacteraceae* | *Luteibacter* | *Luteibacter sp. 329MFSha* |
| ASV37 | 0.038221 | -0.03 | Organic_Ctrl | Autumn | *Bacteroidota* | *Bacteroidia* | *Sphingobacteriales* | *Sphingobacteriaceae* | *Mucilaginibacter* | *Unclassified* |
| ASV63 | 0.049945 | -0.03 | Organic_Ctrl | Autumn | *Actinobacteriota* | *Actinobacteria* | *Micrococcales* | *Microbacteriaceae* | *Unclassified_Microbacteriaceae* | *Unclassified* |
| ASV33 | 0.023963 | 0.01 | Organic_Ctrl | Autumn | *Proteobacteria* | *Alphaproteobacteria* | *Rhizobiales* | *Devosiaceae* | *Devosia* | *Unclassified* |
| ASV29 | 0.049945 | -0.01 | Organic_Ctrl | Autumn | *Bacteroidota* | *Bacteroidia* | *Cytophagales* | *Spirosomaceae* | *Dyadobacter* | *Unclassified* |
| ASV24 | 0.049528 | -0.01 | Organic_Ctrl | Autumn | *Proteobacteria* | *Alphaproteobacteria* | *Caulobacterales* | *Caulobacteraceae* | *Brevundimonas* | *Unclassified* |
| ASV1228 | 0.018792 | -3.42 | Integrated_BMs | Spring | *Proteobacteria* | *Alphaproteobacteria* | *Sphingomonadales* | *Sphingomonadaceae* | *Sphingomonas* | *Unclassified* |
| ASV1333 | 0.008444 | 2.43 | Integrated_BMs | Spring | *Proteobacteria* | *Gammaproteobacteria* | *Xanthomonadales* | *Xanthomonadaceae* | *Unclassified_Xanthomonadaceae* | *Unclassified* |
| ASV1362 | 0.008444 | 0.8 | Integrated_BMs | Spring | *Bacteroidota* | *Bacteroidia* | *Chitinophagales* | *Chitinophagaceae* | *Chitinophaga* | *Chitinophaga ginsengisegetis* |
| ASV1664 | 6.97E-04 | 5.05 | Integrated_BMs | Spring | *Proteobacteria* | *Gammaproteobacteria* | *Burkholderiales* | *Oxalobacteraceae* | *Noviherbaspirillum* | *Unclassified* |
| ASV1694 | 0.008444 | 3.12 | Integrated_BMs | Spring | *Proteobacteria* | *Gammaproteobacteria* | *Burkholderiales* | *Oxalobacteraceae* | *Herbaspirillum* | *Herbaspirillum autotrophicum* |
| ASV1743 | 0.032468 | -2.88 | Integrated_BMs | Spring | *WPS-2* | *Unclassified* | *Unclassified* | *Unclassified* | *Unclassified_WPS-2* | *Unclassified* |
| ASV2312 | 0.008444 | 1.97 | Integrated_BMs | Spring | *Gemmatimonadota* | *Gemmatimonadetes* | *Gemmatimonadales* | *Gemmatimonadaceae* | *Gemmatimonas* | *Unclassified* |
| ASV2650 | 0.008444 | 4.16 | Integrated_BMs | Spring | *Proteobacteria* | *Gammaproteobacteria* | *Burkholderiales* | *Comamonadaceae* | *Pelomonas* | *Burkholderia sp. kmd_430* |
| ASV2664 | 0.008444 | 1.65 | Integrated_BMs | Spring | *Verrucomicrobiota* | *Verrucomicrobiae* | *Chthoniobacterales* | *Terrimicrobiaceae* | *Terrimicrobium* | *Unclassified* |
| ASV2780 | 6.97E-04 | 10.01 | Integrated_BMs | Spring | *Bacteroidota* | *Bacteroidia* | *Cytophagales* | *Spirosomaceae* | *Fibrisoma* | *Cenchrus americanus* |
| ASV2858 | 0.008444 | 3.75 | Integrated_BMs | Spring | *Actinobacteriota* | *Thermoleophilia* | *Solirubrobacterales* | *67-14* | *Unclassified_67-14* | *Unclassified* |
| ASV339 | 6.97E-04 | 1.54 | Integrated_BMs | Spring | *Actinobacteriota* | *Actinobacteria* | *Micrococcales* | *Micrococcaceae* | *Pseudarthrobacter* | *Arthrobacter sp. XBGRY2* |
| ASV386 | 0.020225 | 0.12 | Integrated_BMs | Spring | *Bacteroidota* | *Bacteroidia* | *Sphingobacteriales* | *Sphingobacteriaceae* | *Mucilaginibacter* | *Mucilaginibacter aquaedulcis* |
| ASV3861 | 0.008444 | 3.69 | Integrated_BMs | Spring | *Proteobacteria* | *Gammaproteobacteria* | *Burkholderiales* | *SC-I-84* | *Unclassified_SC-I-84* | *Unclassified* |
| ASV3941 | 0.008444 | 2.62 | Integrated_BMs | Spring | *Bacteroidota* | *Bacteroidia* | *Cytophagales* | *Spirosomaceae* | *Spirosoma* | *Unclassified* |
| ASV396 | 0.045709 | 0.06 | Integrated_BMs | Spring | *Proteobacteria* | *Alphaproteobacteria* | *Sphingomonadales* | *Sphingomonadaceae* | *Sphingomonas* | *Unclassified* |
| ASV4048 | 0.008444 | 4.65 | Integrated_BMs | Spring | *Proteobacteria* | *Gammaproteobacteria* | *Burkholderiales* | *Burkholderiaceae* | *Burkholderia-Caballeronia-Paraburkholderia* | *Caballeronia telluris* |
| ASV464 | 0.01116 | 0.14 | Integrated_BMs | Spring | *Proteobacteria* | *Gammaproteobacteria* | *Burkholderiales* | *Oxalobacteraceae* | *Massilia* | *Unclassified* |
| ASV4964 | 0.008444 | 5.91 | Integrated_BMs | Spring | *Proteobacteria* | *Gammaproteobacteria* | *Burkholderiales* | *Burkholderiaceae* | *Lautropia* | *Unclassified* |
| ASV523 | 0.018792 | -0.99 | Integrated_BMs | Spring | *Actinobacteriota* | *Actinobacteria* | *Streptomycetales* | *Streptomycetaceae* | *Streptomyces* | *Streptomyces albus* |
| ASV536 | 0.010791 | 0.18 | Integrated_BMs | Spring | *Proteobacteria* | *Alphaproteobacteria* | *Rhizobiales* | *Rhizobiaceae* | *Mesorhizobium* | *Mesorhizobium huakuii* |
| ASV709 | 0.032468 | -1.76 | Integrated_BMs | Spring | *Acidobacteriota* | *Blastocatellia* | *Blastocatellales* | *Blastocatellaceae* | *Unclassified_Blastocatellaceae* | *Unclassified* |
| ASV744 | 0.008444 | 0.63 | Integrated_BMs | Spring | *Proteobacteria* | *Alphaproteobacteria* | *Rhizobiales* | *Rhizobiaceae* | *Allorhizobium-Neorhizobium-Pararhizobium-Rhizobium* | *Unclassified* |
| ASV757 | 0.008944 | 0.18 | Integrated_BMs | Spring | *Proteobacteria* | *Gammaproteobacteria* | *Burkholderiales* | *Oxalobacteraceae* | *Massilia* | *Unclassified* |
| ASV767 | 0.018792 | -1.91 | Integrated_BMs | Spring | *Proteobacteria* | *Gammaproteobacteria* | *Burkholderiales* | *Oxalobacteraceae* | *Actimicrobium* | *Unclassified* |
| ASV789 | 0.008444 | 0.2 | Integrated_BMs | Spring | *Proteobacteria* | *Alphaproteobacteria* | *Caulobacterales* | *Caulobacteraceae* | *Unclassified_Caulobacteraceae* | *Unclassified* |
| ASV848 | 0.006782 | 2.53 | Integrated_BMs | Spring | *Proteobacteria* | *Gammaproteobacteria* | *Burkholderiales* | *Burkholderiaceae* | *Burkholderia-Caballeronia-Paraburkholderia* | *Unclassified* |
| ASV896 | 0.008444 | 1.28 | Integrated_BMs | Spring | *Proteobacteria* | *Gammaproteobacteria* | *Pseudomonadales* | *Pseudomonadaceae* | *Pseudomonas* | *bacterium endosymbiont of Curculio koreanus* |
| ASV974 | 0.012212 | -0.41 | Integrated_BMs | Spring | *Actinobacteriota* | *Thermoleophilia* | *Gaiellales* | *Unclassified* | *Unclassified_Gaiellales* | *Unclassified* |
| ASV1065 | 0.018792 | -1.95 | Integrated_BMs | Spring | *Proteobacteria* | *Alphaproteobacteria* | *Rhizobiales* | *Xanthobacteraceae* | *Rhodoplanes* | *Unclassified* |
| ASV1078 | 0.032468 | -2.4 | Integrated_BMs | Spring | *Acidobacteriota* | *Blastocatellia* | *Blastocatellales* | *Blastocatellaceae* | *JGI 0001001-H03* | *Unclassified* |
| ASV1117 | 0.018792 | -2.98 | Integrated_BMs | Spring | *Acidobacteriota* | *Acidobacteriae* | *Acidobacteriales* | *Unclassified* | *Unclassified_Acidobacteriales* | *Unclassified* |
| ASV1138 | 0.009743 | -11.12 | Integrated_BMs | Spring | *Firmicutes* | *Bacilli* | *Paenibacillales* | *Paenibacillaceae* | *Paenibacillus* | *Paenibacillus sacheonensis* |
| ASV395 | 0.009743 | -4.48 | Integrated_BMs | Spring | *Actinobacteriota* | *Actinobacteria* | *Propionibacteriales* | *Nocardioidaceae* | *Nocardioides* | *Unclassified* |
| ASV526 | 0.009743 | -1.24 | Integrated_BMs | Spring | *Proteobacteria* | *Alphaproteobacteria* | *Sphingomonadales* | *Sphingomonadaceae* | *Sphingomonas* | *Unclassified* |
| ASV554 | 0.009743 | -2.42 | Integrated_BMs | Spring | *Proteobacteria* | *Gammaproteobacteria* | *Burkholderiales* | *Comamonadaceae* | *Rhizobacter* | *Unclassified* |
| ASV588 | 0.009743 | -2.76 | Integrated_BMs | Spring | *Proteobacteria* | *Alphaproteobacteria* | *Rhizobiales* | *Hyphomicrobiaceae* | *Hyphomicrobium* | *Unclassified* |
| ASV639 | 0.044138 | -0.21 | Integrated_BMs | Spring | *Bacteroidota* | *Bacteroidia* | *Chitinophagales* | *Chitinophagaceae* | *Flavisolibacter* | *Unclassified* |
| ASV694 | 0.026345 | -0.28 | Integrated_BMs | Spring | *Bacteroidota* | *Bacteroidia* | *Chitinophagales* | *Chitinophagaceae* | *Unclassified_Chitinophagaceae* | *Unclassified* |
| ASV696 | 0.018792 | -1.97 | Integrated_BMs | Spring | *Bacteroidota* | *Bacteroidia* | *Chitinophagales* | *Chitinophagaceae* | *Ferruginibacter* | *Unclassified* |
| ASV806 | 0.009743 | -7.67 | Integrated_BMs | Spring | *Proteobacteria* | *Alphaproteobacteria* | *Rhizobiales* | *Xanthobacteraceae* | *Bradyrhizobium* | *Unclassified* |
| ASV815 | 0.018792 | -1.61 | Integrated_BMs | Spring | *Actinobacteriota* | *Thermoleophilia* | *Gaiellales* | *Gaiellaceae* | *Gaiella* | *Unclassified* |
| ASV853 | 0.02657 | -0.35 | Integrated_BMs | Spring | *Proteobacteria* | *Alphaproteobacteria* | *Azospirillales* | *Unclassified* | *Unclassified_Azospirillales* | *Unclassified* |
| ASV855 | 0.018792 | -1.92 | Integrated_BMs | Spring | *Proteobacteria* | *Alphaproteobacteria* | *Rhizobiales* | *Xanthobacteraceae* | *Unclassified_Xanthobacteraceae* | *Unclassified* |
| ASV897 | 0.040403 | -0.3 | Integrated_BMs | Spring | *Proteobacteria* | *Alphaproteobacteria* | *Rhizobiales* | *Rhizobiales Incertae Sedis* | *Nordella* | *Unclassified* |
| ASV899 | 0.009743 | -4.87 | Integrated_BMs | Spring | *Actinobacteriota* | *Actinobacteria* | *Propionibacteriales* | *Nocardioidaceae* | *Nocardioides* | *Nocardioides sp.* |
| ASV911 | 0.032468 | -1.74 | Integrated_BMs | Spring | *Proteobacteria* | *Alphaproteobacteria* | *Rhizobiales* | *Rhizobiales Incertae Sedis* | *Nordella* | *Unclassified* |
| ASV1356 | 0.018792 | -4.67 | Integrated_BMs | Spring | *Proteobacteria* | *Gammaproteobacteria* | *Burkholderiales* | *SC-I-84* | *Unclassified_SC-I-84* | *Unclassified* |
| ASV498 | 0.027226 | -0.19 | Integrated_BMs | Spring | *Acidobacteriota* | *Blastocatellia* | *Blastocatellales* | *Blastocatellaceae* | *Unclassified_Blastocatellaceae* | *Unclassified* |
| ASV511 | 0.03917 | -0.17 | Integrated_BMs | Spring | *Actinobacteriota* | *Actinobacteria* | *Micrococcales* | *Micrococcaceae* | *Paeniglutamicibacter* | *Arthrobacter sp.* |
| ASV1152 | 0.032468 | -4.32 | Integrated_BMs | Spring | *Proteobacteria* | *Alphaproteobacteria* | *Rhizobiales* | *Rhizobiaceae* | *Aureimonas* | *Unclassified* |
| ASV306 | 0.018792 | -0.15 | Integrated_BMs | Spring | *Proteobacteria* | *Gammaproteobacteria* | *Xanthomonadales* | *Xanthomonadaceae* | *Lysobacter* | *Lysobacter sp.* |
| ASV407 | 0.010182 | -0.42 | Integrated_BMs | Spring | *Proteobacteria* | *Alphaproteobacteria* | *Rhizobiales* | *Hyphomicrobiaceae* | *Pedomicrobium* | *Unclassified* |
| ASV775 | 0.032468 | -0.27 | Integrated_BMs | Spring | *Proteobacteria* | *Gammaproteobacteria* | *Burkholderiales* | *Comamonadaceae* | *Rhodoferax* | *Unclassified* |
| ASV309 | 0.040375 | -0.11 | Integrated_BMs | Spring | *Actinobacteriota* | *Acidimicrobiia* | *Microtrichales* | *Ilumatobacteraceae* | *CL500-29 marine group* | *Unclassified* |
| ASV323 | 0.010652 | -0.35 | Integrated_BMs | Spring | *Acidobacteriota* | *Blastocatellia* | *Blastocatellales* | *Blastocatellaceae* | *Unclassified_Blastocatellaceae* | *Unclassified* |
| ASV1282 | 0.032468 | 0.37 | Integrated_BMs | Spring | *Proteobacteria* | *Gammaproteobacteria* | *Burkholderiales* | *SC-I-84* | *Unclassified_SC-I-84* | *Unclassified* |
| ASV271 | 0.021651 | 0.13 | Integrated_BMs | Spring | *Proteobacteria* | *Alphaproteobacteria* | *Rhizobiales* | *Xanthobacteraceae* | *Bradyrhizobium* | *Unclassified* |
| ASV298 | 0.032194 | 0.08 | Integrated_BMs | Spring | *Proteobacteria* | *Alphaproteobacteria* | *Sphingomonadales* | *Sphingomonadaceae* | *Sphingomonas* | *Unclassified* |
| ASV828 | 6.97E-04 | 4.46 | Integrated_BMs | Spring | *Proteobacteria* | *Gammaproteobacteria* | *Burkholderiales* | *Burkholderiaceae* | *Burkholderia-Caballeronia-Paraburkholderia* | *Burkholderia sp. DCY113* |
| ASV145 | 0.027336 | 0.02 | Integrated_BMs | Spring | *Bacteroidota* | *Bacteroidia* | *Sphingobacteriales* | *Sphingobacteriaceae* | *Pedobacter* | *Pedobacter koreensis* |
| ASV609 | 0.008444 | 0.23 | Integrated_BMs | Spring | *Proteobacteria* | *Gammaproteobacteria* | *Xanthomonadales* | *Rhodanobacteraceae* | *Tahibacter* | *Unclassified* |
| ASV392 | 0.044364 | 0.08 | Integrated_BMs | Spring | *Acidobacteriota* | *Acidobacteriae* | *Acidobacteriales* | *Acidobacteriaceae (Subgroup 1)* | *Acidipila-Silvibacterium* | *Unclassified* |
| ASV276 | 0.016746 | 0.13 | Integrated_BMs | Spring | *Proteobacteria* | *Gammaproteobacteria* | *Burkholderiales* | *Burkholderiaceae* | *Burkholderia-Caballeronia-Paraburkholderia* | *Paraburkholderia phytofirmans* |
| ASV375 | 0.016041 | -0.23 | Integrated_BMs | Spring | *Actinobacteriota* | *Actinobacteria* | *Glycomycetales* | *Glycomycetaceae* | *Glycomyces* | *Glycomyces sp.* |
| ASV121 | 0.044364 | -0.05 | Integrated_BMs | Spring | *Firmicutes* | *Bacilli* | *Bacillales* | *Bacillaceae* | *Bacillus* | *Bacillus butanolivorans* |
| ASV191 | 0.005876 | 0.22 | Integrated_BMs | Spring | *Firmicutes* | *Bacilli* | *Paenibacillales* | *Paenibacillaceae* | *Paenibacillus* | *Unclassified* |
| ASV263 | 0.022244 | -0.19 | Integrated_BMs | Spring | *Gemmatimonadota* | *Gemmatimonadetes* | *Gemmatimonadales* | *Gemmatimonadaceae* | *Unclassified_Gemmatimonadaceae* | *Unclassified* |
| ASV150 | 0.008444 | 0.1 | Integrated_BMs | Spring | *Proteobacteria* | *Gammaproteobacteria* | *Burkholderiales* | *Burkholderiaceae* | *Burkholderia-Caballeronia-Paraburkholderia* | *Burkholderia sp. HB1* |
| ASV103 | 0.013126 | -0.13 | Integrated_BMs | Spring | *Proteobacteria* | *Alphaproteobacteria* | *Sphingomonadales* | *Sphingomonadaceae* | *Sphingomonas* | *Unclassified* |
| ASV106 | 0.005685 | -0.62 | Integrated_BMs | Spring | *Proteobacteria* | *Alphaproteobacteria* | *Rhizobiales* | *Stappiaceae* | *Stappia* | *Unclassified* |
| ASV111 | 0.02657 | -0.06 | Integrated_BMs | Spring | *Actinobacteriota* | *Actinobacteria* | *Streptomycetales* | *Streptomycetaceae* | *Streptomyces* | *Streptomyces sp. N01-1637* |
| ASV1069 | 0.020538 | -0.84 | Integrated_Ctrl | Spring | *Firmicutes* | *Bacilli* | *Paenibacillales* | *Paenibacillaceae* | *Paenibacillus* | *Paenibacillus sp. 1ZS3-15* |
| ASV10845 | 0.039065 | 26.34 | Integrated_Ctrl | Spring | *Verrucomicrobiota* | *Verrucomicrobiae* | *Chthoniobacterales* | *Chthoniobacteraceae* | *Chthoniobacter* | *Unclassified* |
| ASV1172 | 0.018792 | -3.17 | Integrated_Ctrl | Spring | *Proteobacteria* | *Gammaproteobacteria* | *Gammaproteobacteria Incertae Sedis* | *Unknown Family* | *Candidatus Ovatusbacter* | *Unclassified* |
| ASV1222 | 0.032468 | -2.51 | Integrated_Ctrl | Spring | *Verrucomicrobiota* | *Verrucomicrobiae* | *Chthoniobacterales* | *Chthoniobacteraceae* | *Chthoniobacter* | *Unclassified* |
| ASV1244 | 0.032468 | -12.26 | Integrated_Ctrl | Spring | *Verrucomicrobiota* | *Verrucomicrobiae* | *Verrucomicrobiales* | *Rubritaleaceae* | *Luteolibacter* | *Unclassified* |
| ASV1586 | 0.008444 | 1.95 | Integrated_Ctrl | Spring | *Proteobacteria* | *Alphaproteobacteria* | *Micropepsales* | *Micropepsaceae* | *Unclassified_Micropepsaceae* | *Unclassified* |
| ASV2945 | 0.008444 | 2.76 | Integrated_Ctrl | Spring | *Proteobacteria* | *Gammaproteobacteria* | *Xanthomonadales* | *Rhodanobacteraceae* | *Mizugakiibacter* | *Unclassified* |
| ASV396 | 0.018792 | -1 | Integrated_Ctrl | Spring | *Proteobacteria* | *Alphaproteobacteria* | *Sphingomonadales* | *Sphingomonadaceae* | *Sphingomonas* | *Unclassified* |
| ASV415 | 0.008444 | 1.27 | Integrated_Ctrl | Spring | *Proteobacteria* | *Gammaproteobacteria* | *Burkholderiales* | *Oxalobacteraceae* | *Massilia* | *Unclassified* |
| ASV465 | 0.032468 | -0.75 | Integrated_Ctrl | Spring | *Verrucomicrobiota* | *Verrucomicrobiae* | *Verrucomicrobiales* | *Rubritaleaceae* | *Luteolibacter* | *Unclassified* |
| ASV474 | 0.020416 | -0.17 | Integrated_Ctrl | Spring | *Firmicutes* | *Bacilli* | *Paenibacillales* | *Paenibacillaceae* | *Paenibacillus* | *Unclassified* |
| ASV518 | 0.009743 | -1.51 | Integrated_Ctrl | Spring | *Actinobacteriota* | *Actinobacteria* | *Micromonosporales* | *Micromonosporaceae* | *Catellatospora* | *Catellatospora citrea* |
| ASV522 | 0.032468 | -0.66 | Integrated_Ctrl | Spring | *Chloroflexi* | *KD4-96* | *Unclassified* | *Unclassified* | *Unclassified_KD4-96* | *Unclassified* |
| ASV535 | 0.032468 | -0.74 | Integrated_Ctrl | Spring | *Actinobacteriota* | *Thermoleophilia* | *Solirubrobacterales* | *Solirubrobacteraceae* | *Solirubrobacter* | *Unclassified* |
| ASV594 | 0.008444 | -0.63 | Integrated_Ctrl | Spring | *Proteobacteria* | *Gammaproteobacteria* | *Xanthomonadales* | *Rhodanobacteraceae* | *Dyella* | *Dyella kyungheensis* |
| ASV601 | 0.04222 | -0.18 | Integrated_Ctrl | Spring | *Chloroflexi* | *Gitt-GS-136* | *Unclassified* | *Unclassified* | *Unclassified_Gitt-GS-136* | *Unclassified* |
| ASV613 | 0.018792 | -0.78 | Integrated_Ctrl | Spring | *Actinobacteriota* | *Actinobacteria* | *Propionibacteriales* | *Nocardioidaceae* | *Marmoricola* | *Nocardioides sp. MTD22* |
| ASV654 | 0.005362 | -0.84 | Integrated_Ctrl | Spring | *Bacteroidota* | *Bacteroidia* | *Chitinophagales* | *Chitinophagaceae* | *Chitinophaga* | *Unclassified* |
| ASV677 | 0.009743 | -0.4 | Integrated_Ctrl | Spring | *Bacteroidota* | *Bacteroidia* | *Chitinophagales* | *Chitinophagaceae* | *Unclassified_Chitinophagaceae* | *Unclassified* |
| ASV706 | 0.032468 | -1.16 | Integrated_Ctrl | Spring | *Acidobacteriota* | *Blastocatellia* | *Blastocatellales* | *Blastocatellaceae* | *Unclassified_Blastocatellaceae* | *Unclassified* |
| ASV709 | 0.032468 | -1.76 | Integrated_Ctrl | Spring | *Acidobacteriota* | *Blastocatellia* | *Blastocatellales* | *Blastocatellaceae* | *Unclassified_Blastocatellaceae* | *Unclassified* |
| ASV715 | 0.006716 | -4.74 | Integrated_Ctrl | Spring | *Proteobacteria* | *Gammaproteobacteria* | *Xanthomonadales* | *Xanthomonadaceae* | *Pseudoxanthomonas* | *Unclassified* |
| ASV823 | 0.009743 | -3.29 | Integrated_Ctrl | Spring | *Actinobacteriota* | *Actinobacteria* | *Streptomycetales* | *Streptomycetaceae* | *Streptomyces* | *Streptomyces exfoliatus* |
| ASV848 | 0.032468 | -3.07 | Integrated_Ctrl | Spring | *Proteobacteria* | *Gammaproteobacteria* | *Burkholderiales* | *Burkholderiaceae* | *Burkholderia-Caballeronia-Paraburkholderia* | *Unclassified* |
| ASV966 | 0.018792 | -2.82 | Integrated_Ctrl | Spring | *Proteobacteria* | *Gammaproteobacteria* | *Xanthomonadales* | *Rhodanobacteraceae* | *Dokdonella* | *Unclassified* |
| ASV973 | 0.032468 | -3.29 | Integrated_Ctrl | Spring | *Proteobacteria* | *Alphaproteobacteria* | *Rhizobiales* | *Xanthobacteraceae* | *Unclassified_Xanthobacteraceae* | *Unclassified* |
| ASV976 | 0.032468 | -4.26 | Integrated_Ctrl | Spring | *Proteobacteria* | *Alphaproteobacteria* | *Rhizobiales* | *Xanthobacteraceae* | *Bradyrhizobium* | *Unclassified* |
| ASV1370 | 0.009743 | 0.42 | Integrated_Ctrl | Spring | *Bacteroidota* | *Bacteroidia* | *Chitinophagales* | *Chitinophagaceae* | *Aurantisolimonas* | *Chitinophagaceae bacterium BLB-2F4-1* |
| ASV1530 | 0.008444 | 3.06 | Integrated_Ctrl | Spring | *Acidobacteriota* | *Acidobacteriae* | *Bryobacterales* | *Bryobacteraceae* | *Bryobacter* | *Unclassified* |
| ASV1871 | 0.008444 | 2.98 | Integrated_Ctrl | Spring | *Actinobacteriota* | *Thermoleophilia* | *Gaiellales* | *Unclassified* | *Unclassified_Gaiellales* | *Unclassified* |
| ASV321 | 0.03423 | -0.09 | Integrated_Ctrl | Spring | *Proteobacteria* | *Alphaproteobacteria* | *Rhizobiales* | *Xanthobacteraceae* | *Unclassified_Xanthobacteraceae* | *Unclassified* |
| ASV454 | 0.028049 | -0.2 | Integrated_Ctrl | Spring | *Proteobacteria* | *Alphaproteobacteria* | *Rhizobiales* | *Methyloligellaceae* | *Unclassified_Methyloligellaceae* | *Unclassified* |
| ASV249 | 0.009659 | -0.38 | Integrated_Ctrl | Spring | *Bacteroidota* | *Bacteroidia* | *Cytophagales* | *Spirosomaceae* | *Dyadobacter* | *Unclassified* |
| ASV1859 | 0.008444 | 3.01 | Integrated_Ctrl | Spring | *Firmicutes* | *Bacilli* | *Bacillales* | *Sporolactobacillaceae* | *Sporolactobacillus* | *Unclassified* |
| ASV1932 | 0.008444 | 2.98 | Integrated_Ctrl | Spring | *Proteobacteria* | *Alphaproteobacteria* | *Elsterales* | *Unclassified* | *Unclassified_Elsterales* | *Unclassified* |
| ASV1152 | 0.033876 | 0.16 | Integrated_Ctrl | Spring | *Proteobacteria* | *Alphaproteobacteria* | *Rhizobiales* | *Rhizobiaceae* | *Aureimonas* | *Unclassified* |
| ASV1467 | 0.004152 | 1.79 | Integrated_Ctrl | Spring | *Proteobacteria* | *Alphaproteobacteria* | *Caulobacterales* | *Caulobacteraceae* | *Brevundimonas* | *Unclassified* |
| ASV1488 | 0.046595 | 0.32 | Integrated_Ctrl | Spring | *Chloroflexi* | *Chloroflexia* | *Thermomicrobiales* | *Thermomicrobiaceae* | *Nitrolancea* | *Unclassified* |
| ASV1584 | 6.97E-04 | 4.19 | Integrated_Ctrl | Spring | *Bacteroidota* | *Bacteroidia* | *Sphingobacteriales* | *Sphingobacteriaceae* | *Pedobacter* | *Unclassified* |
| ASV2460 | 6.97E-04 | 13.55 | Integrated_Ctrl | Spring | *Patescibacteria* | *Saccharimonadia* | *Saccharimonadales* | *Unclassified* | *Unclassified_Saccharimonadales* | *Unclassified* |
| ASV946 | 0.032468 | 0.11 | Integrated_Ctrl | Spring | *Proteobacteria* | *Alphaproteobacteria* | *Caulobacterales* | *Caulobacteraceae* | *Unclassified_Caulobacteraceae* | *Unclassified* |
| ASV1504 | 0.008444 | 0.49 | Integrated_Ctrl | Spring | *Bacteroidota* | *Bacteroidia* | *Chitinophagales* | *Chitinophagaceae* | *Edaphobaculum* | *Unclassified* |
| ASV1519 | 0.009006 | 1.1 | Integrated_Ctrl | Spring | *Proteobacteria* | *Gammaproteobacteria* | *Xanthomonadales* | *Rhodanobacteraceae* | *Rhodanobacter* | *Unclassified* |
| ASV2129 | 0.004458 | 1.95 | Integrated_Ctrl | Spring | *Proteobacteria* | *Alphaproteobacteria* | *Caulobacterales* | *Caulobacteraceae* | *Brevundimonas* | *Unclassified* |
| ASV1487 | 0.038124 | 0.44 | Integrated_Ctrl | Spring | *Chloroflexi* | *Ktedonobacteria* | *C0119* | *Unclassified* | *Unclassified_C0119* | *Unclassified* |
| ASV1542 | 0.016041 | 0.37 | Integrated_Ctrl | Spring | *Proteobacteria* | *Gammaproteobacteria* | *Diplorickettsiales* | *Diplorickettsiaceae* | *Unclassified_Diplorickettsiaceae* | *Unclassified* |
| ASV265 | 0.029104 | 0.07 | Integrated_Ctrl | Spring | *Acidobacteriota* | *Acidobacteriae* | *Acidobacteriales* | *Acidobacteriaceae (Subgroup 1)* | *Acidipila-Silvibacterium* | *Unclassified* |
| ASV308 | 0.045472 | 0.08 | Integrated_Ctrl | Spring | *Proteobacteria* | *Gammaproteobacteria* | *Burkholderiales* | *SC-I-84* | *Unclassified_SC-I-84* | *Unclassified* |
| ASV377 | 0.009743 | 0.13 | Integrated_Ctrl | Spring | *Actinobacteriota* | *Actinobacteria* | *Streptosporangiales* | *Thermomonosporaceae* | *Actinoallomurus* | *Actinoallomurus sp.* |
| ASV668 | 0.017653 | 0.25 | Integrated_Ctrl | Spring | *Proteobacteria* | *Gammaproteobacteria* | *Xanthomonadales* | *Rhodanobacteraceae* | *Mizugakiibacter* | *Unclassified* |
| ASV691 | 0.049401 | 0.2 | Integrated_Ctrl | Spring | *Proteobacteria* | *Gammaproteobacteria* | *Xanthomonadales* | *Rhodanobacteraceae* | *Rhodanobacter* | *Unclassified* |
| ASV721 | 0.007885 | 0.79 | Integrated_Ctrl | Spring | *Actinobacteriota* | *Actinobacteria* | *Corynebacteriales* | *Nocardiaceae* | *Nocardia* | *Nocardia nova* |
| ASV661 | 0.011841 | 0.45 | Integrated_Ctrl | Spring | *Proteobacteria* | *Alphaproteobacteria* | *Micavibrionales* | *Unclassified* | *Unclassified_Micavibrionales* | *Unclassified* |
| ASV148 | 6.97E-04 | 7.53 | Integrated_Ctrl | Spring | *Bacteroidota* | *Bacteroidia* | *Chitinophagales* | *Chitinophagaceae* | *Chitinophaga* | *Unclassified* |
| ASV88 | 0.008444 | 0.12 | Integrated_Ctrl | Spring | *Bacteroidota* | *Bacteroidia* | *Chitinophagales* | *Chitinophagaceae* | *Chitinophaga* | *Unclassified* |
| ASV968 | 0.009715 | 0.82 | Integrated_Ctrl | Spring | *Cyanobacteria* | *Vampirivibrionia* | *Obscuribacterales* | *Obscuribacteraceae* | *Unclassified_Obscuribacteraceae* | *Unclassified* |
| ASV758 | 0.030966 | -1.48 | Integrated_Ctrl | Spring | *Proteobacteria* | *Gammaproteobacteria* | *Pseudomonadales* | *Pseudomonadaceae* | *Pseudomonas* | *Pseudomonas sp.* |
| ASV440 | 0.048857 | -0.17 | Integrated_Ctrl | Spring | *Proteobacteria* | *Gammaproteobacteria* | *Burkholderiales* | *SC-I-84* | *agricultural soil bacterium SC-I-84* | *Unclassified* |
| ASV191 | 0.045658 | -0.12 | Integrated_Ctrl | Spring | *Firmicutes* | *Bacilli* | *Paenibacillales* | *Paenibacillaceae* | *Paenibacillus* | *Unclassified* |
| ASV202 | 0.032468 | -0.14 | Integrated_Ctrl | Spring | *Proteobacteria* | *Alphaproteobacteria* | *Rhizobiales* | *Rhizobiales Incertae Sedis* | *Unclassified_Rhizobiales Incertae Sedis* | *Unclassified* |
| ASV72 | 0.005685 | -0.18 | Integrated_Ctrl | Spring | *Firmicutes* | *Bacilli* | *Bacillales* | *Bacillaceae* | *Bacillus* | *Unclassified* |
| ASV118 | 0.040344 | -0.09 | Integrated_Ctrl | Spring | *Actinobacteriota* | *Actinobacteria* | *Micrococcales* | *Micrococcaceae* | *Paenarthrobacter* | *Arthrobacter sp. P2* |
| ASV61 | 0.032468 | -0.03 | Integrated_Ctrl | Spring | *Proteobacteria* | *Gammaproteobacteria* | *Xanthomonadales* | *Rhodanobacteraceae* | *Luteibacter* | *Luteibacter sp. 329MFSha* |
| ASV48 | 0.015148 | -0.05 | Integrated_Ctrl | Spring | *Proteobacteria* | *Gammaproteobacteria* | *Xanthomonadales* | *Rhodanobacteraceae* | *Luteibacter* | *Unclassified* |
| ASV63 | 0.01512 | 0.05 | Integrated_Ctrl | Spring | *Actinobacteriota* | *Actinobacteria* | *Micrococcales* | *Microbacteriaceae* | *Unclassified_Microbacteriaceae* | *Unclassified* |
| ASV31 | 0.006782 | 0.01 | Integrated_Ctrl | Spring | *Bacteroidota* | *Bacteroidia* | *Sphingobacteriales* | *Sphingobacteriaceae* | *Pedobacter* | *Unclassified* |
| ASV35 | 6.97E-04 | 0.47 | Integrated_Ctrl | Spring | *Proteobacteria* | *Alphaproteobacteria* | *Sphingomonadales* | *Sphingomonadaceae* | *Novosphingobium* | *Unclassified* |
| ASV17 | 0.005282 | 0.01 | Integrated_Ctrl | Spring | *Proteobacteria* | *Alphaproteobacteria* | *Sphingomonadales* | *Sphingomonadaceae* | *Sphingomonas* | *Unclassified* |
| ASV1029 | 6.97E-04 | 9.29 | Organic_BMs | Spring | *Bacteroidota* | *Bacteroidia* | *Cytophagales* | *Spirosomaceae* | *Larkinella* | *Unclassified* |
| ASV1069 | 6.97E-04 | 10.96 | Organic_BMs | Spring | *Firmicutes* | *Bacilli* | *Paenibacillales* | *Paenibacillaceae* | *Paenibacillus* | *Paenibacillus sp. 1ZS3-15* |
| ASV1172 | 0.016041 | 0.28 | Organic_BMs | Spring | *Proteobacteria* | *Gammaproteobacteria* | *Gammaproteobacteria Incertae Sedis* | *Unknown Family* | *Candidatus Ovatusbacter* | *Unclassified* |
| ASV1222 | 0.032468 | 0.22 | Organic_BMs | Spring | *Verrucomicrobiota* | *Verrucomicrobiae* | *Chthoniobacterales* | *Chthoniobacteraceae* | *Chthoniobacter* | *Unclassified* |
| ASV1228 | 0.041625 | 0.19 | Organic_BMs | Spring | *Proteobacteria* | *Alphaproteobacteria* | *Sphingomonadales* | *Sphingomonadaceae* | *Sphingomonas* | *Unclassified* |
| ASV1236 | 6.97E-04 | 545.85 | Organic_BMs | Spring | *Proteobacteria* | *Gammaproteobacteria* | *Xanthomonadales* | *Rhodanobacteraceae* | *Luteibacter* | *Luteibacter yeojuensis* |
| ASV1244 | 0.031829 | 0.29 | Organic_BMs | Spring | *Verrucomicrobiota* | *Verrucomicrobiae* | *Verrucomicrobiales* | *Rubritaleaceae* | *Luteolibacter* | *Unclassified* |
| ASV1353 | 0.008444 | 0.46 | Organic_BMs | Spring | *Bacteroidota* | *Bacteroidia* | *Chitinophagales* | *Chitinophagaceae* | *Unclassified_Chitinophagaceae* | *Unclassified* |
| ASV1725 | 0.006716 | 0.68 | Organic_BMs | Spring | *Actinobacteriota* | *Actinobacteria* | *Propionibacteriales* | *Nocardioidaceae* | *Nocardioides* | *Unclassified* |
| ASV1743 | 6.97E-04 | 29.83 | Organic_BMs | Spring | *WPS-2* | *Unclassified* | *Unclassified* | *Unclassified* | *Unclassified_WPS-2* | *Unclassified* |
| ASV1745 | 0.045687 | 0.32 | Organic_BMs | Spring | *Actinobacteriota* | *MB-A2-108* | *Unclassified* | *Unclassified* | *Unclassified_MB-A2-108* | *Unclassified* |
| ASV1754 | 0.027482 | 0.23 | Organic_BMs | Spring | *Actinobacteriota* | *Actinobacteria* | *Frankiales* | *Sporichthyaceae* | *Unclassified_Sporichthyaceae* | *Unclassified* |
| ASV2005 | 0.008444 | 2.04 | Organic_BMs | Spring | *Proteobacteria* | *Alphaproteobacteria* | *Rhizobiales* | *Beijerinckiaceae* | *Microvirga* | *Unclassified* |
| ASV2065 | 0.008444 | 0.47 | Organic_BMs | Spring | *Firmicutes* | *Bacilli* | *Paenibacillales* | *Paenibacillaceae* | *Paenibacillus* | *Unclassified* |
| ASV2112 | 0.008444 | 3.14 | Organic_BMs | Spring | *Firmicutes* | *Limnochordia* | *Hydrogenispora* | *Unclassified* | *Unclassified_Hydrogenispora* | *Unclassified* |
| ASV2151 | 0.008998 | 0.53 | Organic_BMs | Spring | *Proteobacteria* | *Gammaproteobacteria* | *Diplorickettsiales* | *Diplorickettsiaceae* | *Unclassified_Diplorickettsiaceae* | *Unclassified* |
| ASV2564 | 0.013708 | 0.43 | Organic_BMs | Spring | *Proteobacteria* | *Gammaproteobacteria* | *Legionellales* | *Legionellaceae* | *Legionella* | *Unclassified* |
| ASV2618 | 0.01116 | 0.56 | Organic_BMs | Spring | *Proteobacteria* | *Gammaproteobacteria* | *Gammaproteobacteria Incertae Sedis* | *Unknown Family* | *Acidibacter* | *Unclassified* |
| ASV2957 | 0.008444 | 2.08 | Organic_BMs | Spring | *Chloroflexi* | *Chloroflexia* | *Thermomicrobiales* | *JG30-KF-CM45* | *Unclassified_JG30-KF-CM45* | *Unclassified* |
| ASV2995 | 0.008444 | 2.05 | Organic_BMs | Spring | *Proteobacteria* | *Alphaproteobacteria* | *Sphingomonadales* | *Sphingomonadaceae* | *Sphingomonas* | *Unclassified* |
| ASV3270 | 0.008444 | 2.04 | Organic_BMs | Spring | *Chloroflexi* | *Chloroflexia* | *Thermomicrobiales* | *JG30-KF-CM45* | *Unclassified_JG30-KF-CM45* | *Unclassified* |
| ASV4138 | 0.008444 | 2.61 | Organic_BMs | Spring | *Proteobacteria* | *Gammaproteobacteria* | *Burkholderiales* | *Oxalobacteraceae* | *Herminiimonas* | *Herminiimonas sp. THG-DN7.15* |
| ASV448 | 0.02355 | 0.07 | Organic_BMs | Spring | *Proteobacteria* | *Alphaproteobacteria* | *Rhizobiales* | *Rhizobiaceae* | *Phyllobacterium* | *Phyllobacterium myrsinacearum* |
| ASV488 | 6.97E-04 | 1.76 | Organic_BMs | Spring | *Proteobacteria* | *Gammaproteobacteria* | *Xanthomonadales* | *Rhodanobacteraceae* | *Luteibacter* | *Luteibacter sp. 329MFSha* |
| ASV491 | 0.025683 | 0.05 | Organic_BMs | Spring | *Bacteroidota* | *Bacteroidia* | *Chitinophagales* | *Chitinophagaceae* | *Ferruginibacter* | *Unclassified* |
| ASV558 | 0.008444 | 0.81 | Organic_BMs | Spring | *Actinobacteriota* | *Acidimicrobiia* | *Microtrichales* | *Ilumatobacteraceae* | *CL500-29 marine group* | *Unclassified* |
| ASV594 | 0.014012 | 0.16 | Organic_BMs | Spring | *Proteobacteria* | *Gammaproteobacteria* | *Xanthomonadales* | *Rhodanobacteraceae* | *Dyella* | *Dyella kyungheensis* |
| ASV654 | 0.011918 | 0.24 | Organic_BMs | Spring | *Bacteroidota* | *Bacteroidia* | *Chitinophagales* | *Chitinophagaceae* | *Chitinophaga* | *Unclassified* |
| ASV677 | 0.021521 | 0.21 | Organic_BMs | Spring | *Bacteroidota* | *Bacteroidia* | *Chitinophagales* | *Chitinophagaceae* | *Unclassified_Chitinophagaceae* | *Unclassified* |
| ASV686 | 0.008444 | 0.16 | Organic_BMs | Spring | *Verrucomicrobiota* | *Verrucomicrobiae* | *Verrucomicrobiales* | *Rubritaleaceae* | *Luteolibacter* | *Unclassified* |
| ASV706 | 0.026826 | 0.13 | Organic_BMs | Spring | *Acidobacteriota* | *Blastocatellia* | *Blastocatellales* | *Blastocatellaceae* | *Unclassified_Blastocatellaceae* | *Unclassified* |
| ASV715 | 0.016744 | 0.22 | Organic_BMs | Spring | *Proteobacteria* | *Gammaproteobacteria* | *Xanthomonadales* | *Xanthomonadaceae* | *Pseudoxanthomonas* | *Unclassified* |
| ASV942 | 6.97E-04 | 23.41 | Organic_BMs | Spring | *Proteobacteria* | *Gammaproteobacteria* | *Xanthomonadales* | *Rhodanobacteraceae* | *Luteibacter* | *Unclassified* |
| ASV974 | 0.034457 | 0.36 | Organic_BMs | Spring | *Actinobacteriota* | *Thermoleophilia* | *Gaiellales* | *Unclassified* | *Unclassified_Gaiellales* | *Unclassified* |
| ASV985 | 0.007975 | 0.26 | Organic_BMs | Spring | *Firmicutes* | *Bacilli* | *Bacillales* | *Bacillaceae* | *Bacillus* | *Bacillus sp. (in: Bacteria)* |
| ASV1078 | 0.026826 | 0.18 | Organic_BMs | Spring | *Acidobacteriota* | *Blastocatellia* | *Blastocatellales* | *Blastocatellaceae* | *JGI 0001001-H03* | *Unclassified* |
| ASV395 | 6.97E-04 | 7.07 | Organic_BMs | Spring | *Actinobacteriota* | *Actinobacteria* | *Propionibacteriales* | *Nocardioidaceae* | *Nocardioides* | *Unclassified* |
| ASV806 | 0.032468 | 0.24 | Organic_BMs | Spring | *Proteobacteria* | *Alphaproteobacteria* | *Rhizobiales* | *Xanthobacteraceae* | *Bradyrhizobium* | *Unclassified* |
| ASV852 | 0.022127 | 0.17 | Organic_BMs | Spring | *Proteobacteria* | *Alphaproteobacteria* | *Micropepsales* | *Micropepsaceae* | *Unclassified_Micropepsaceae* | *Unclassified* |
| ASV899 | 6.97E-04 | 10.15 | Organic_BMs | Spring | *Actinobacteriota* | *Actinobacteria* | *Propionibacteriales* | *Nocardioidaceae* | *Nocardioides* | *Nocardioides sp.* |
| ASV511 | 0.013674 | 0.15 | Organic_BMs | Spring | *Actinobacteriota* | *Actinobacteria* | *Micrococcales* | *Micrococcaceae* | *Paeniglutamicibacter* | *Arthrobacter sp.* |
| ASV1488 | 0.032468 | -7.69 | Organic_BMs | Spring | *Chloroflexi* | *Chloroflexia* | *Thermomicrobiales* | *Thermomicrobiaceae* | *Nitrolancea* | *Unclassified* |
| ASV1040 | 0.032468 | -3.47 | Organic_BMs | Spring | *Chloroflexi* | *Ktedonobacteria* | *B12-WMSP1* | *Unclassified* | *Unclassified_B12-WMSP1* | *Unclassified* |
| ASV891 | 0.032468 | -3.6 | Organic_BMs | Spring | *Actinobacteriota* | *Actinobacteria* | *Frankiales* | *Acidothermaceae* | *Acidothermus* | *Unclassified* |
| ASV1487 | 0.018792 | -7.07 | Organic_BMs | Spring | *Chloroflexi* | *Ktedonobacteria* | *C0119* | *Unclassified* | *Unclassified_C0119* | *Unclassified* |
| ASV1542 | 0.032468 | -5.83 | Organic_BMs | Spring | *Proteobacteria* | *Gammaproteobacteria* | *Diplorickettsiales* | *Diplorickettsiaceae* | *Unclassified_Diplorickettsiaceae* | *Unclassified* |
| ASV265 | 0.038452 | -0.12 | Organic_BMs | Spring | *Acidobacteriota* | *Acidobacteriae* | *Acidobacteriales* | *Acidobacteriaceae (Subgroup 1)* | *Acidipila-Silvibacterium* | *Unclassified* |
| ASV377 | 0.018792 | -1.21 | Organic_BMs | Spring | *Actinobacteriota* | *Actinobacteria* | *Streptosporangiales* | *Thermomonosporaceae* | *Actinoallomurus* | *Actinoallomurus sp.* |
| ASV532 | 0.032468 | -0.28 | Organic_BMs | Spring | *Proteobacteria* | *Alphaproteobacteria* | *Rhizobiales* | *Xanthobacteraceae* | *Rhodopseudomonas* | *Unclassified* |
| ASV637 | 0.018792 | -1.6 | Organic_BMs | Spring | *Proteobacteria* | *Gammaproteobacteria* | *Burkholderiales* | *SC-I-84* | *Unclassified_SC-I-84* | *Unclassified* |
| ASV668 | 0.032468 | -0.34 | Organic_BMs | Spring | *Proteobacteria* | *Gammaproteobacteria* | *Xanthomonadales* | *Rhodanobacteraceae* | *Mizugakiibacter* | *Unclassified* |
| ASV691 | 0.009743 | -4.71 | Organic_BMs | Spring | *Proteobacteria* | *Gammaproteobacteria* | *Xanthomonadales* | *Rhodanobacteraceae* | *Rhodanobacter* | *Unclassified* |
| ASV283 | 0.008444 | -0.27 | Organic_BMs | Spring | *Bacteroidota* | *Bacteroidia* | *Sphingobacteriales* | *Sphingobacteriaceae* | *Mucilaginibacter* | *Unclassified* |
| ASV392 | 0.042719 | -0.16 | Organic_BMs | Spring | *Acidobacteriota* | *Acidobacteriae* | *Acidobacteriales* | *Acidobacteriaceae (Subgroup 1)* | *Acidipila-Silvibacterium* | *Unclassified* |
| ASV661 | 0.011108 | -0.81 | Organic_BMs | Spring | *Proteobacteria* | *Alphaproteobacteria* | *Micavibrionales* | *Unclassified* | *Unclassified_Micavibrionales* | *Unclassified* |
| ASV148 | 6.97E-04 | -8.14 | Organic_BMs | Spring | *Bacteroidota* | *Bacteroidia* | *Chitinophagales* | *Chitinophagaceae* | *Chitinophaga* | *Unclassified* |
| ASV88 | 0.02355 | -0.08 | Organic_BMs | Spring | *Bacteroidota* | *Bacteroidia* | *Chitinophagales* | *Chitinophagaceae* | *Chitinophaga* | *Unclassified* |
| ASV968 | 0.012212 | -0.77 | Organic_BMs | Spring | *Cyanobacteria* | *Vampirivibrionia* | *Obscuribacterales* | *Obscuribacteraceae* | *Unclassified_Obscuribacteraceae* | *Unclassified* |
| ASV758 | 6.97E-04 | 9.51 | Organic_BMs | Spring | *Proteobacteria* | *Gammaproteobacteria* | *Pseudomonadales* | *Pseudomonadaceae* | *Pseudomonas* | *Pseudomonas sp.* |
| ASV121 | 0.02355 | 0.02 | Organic_BMs | Spring | *Firmicutes* | *Bacilli* | *Bacillales* | *Bacillaceae* | *Bacillus* | *Bacillus butanolivorans* |
| ASV103 | 0.032468 | 0.04 | Organic_BMs | Spring | *Proteobacteria* | *Alphaproteobacteria* | *Sphingomonadales* | *Sphingomonadaceae* | *Sphingomonas* | *Unclassified* |
| ASV118 | 0.008444 | 0.11 | Organic_BMs | Spring | *Actinobacteriota* | *Actinobacteria* | *Micrococcales* | *Micrococcaceae* | *Paenarthrobacter* | *Arthrobacter sp. P2* |
| ASV61 | 0.008444 | 0.03 | Organic_BMs | Spring | *Proteobacteria* | *Gammaproteobacteria* | *Xanthomonadales* | *Rhodanobacteraceae* | *Luteibacter* | *Luteibacter sp. 329MFSha* |
| ASV63 | 0.045318 | -0.04 | Organic_BMs | Spring | *Actinobacteriota* | *Actinobacteria* | *Micrococcales* | *Microbacteriaceae* | *Unclassified_Microbacteriaceae* | *Unclassified* |
| ASV17 | 0.012212 | -0.03 | Organic_BMs | Spring | *Proteobacteria* | *Alphaproteobacteria* | *Sphingomonadales* | *Sphingomonadaceae* | *Sphingomonas* | *Unclassified* |
| ASV1003 | 0.011165 | 0.25 | Organic_Ctrl | Spring | *Acidobacteriota* | *Blastocatellia* | *Nov-24* | *Unclassified* | *Unclassified_11-24* | *Unclassified* |
| ASV1143 | 0.045008 | 0.16 | Organic_Ctrl | Spring | *Proteobacteria* | *Alphaproteobacteria* | *Rhizobiales* | *Devosiaceae* | *Devosia* | *Unclassified* |
| ASV1217 | 6.97E-04 | 15.57 | Organic_Ctrl | Spring | *Actinobacteriota* | *Actinobacteria* | *Micromonosporales* | *Micromonosporaceae* | *Asanoa* | *Unclassified* |
| ASV1331 | 6.97E-04 | 6.78 | Organic_Ctrl | Spring | *Gemmatimonadota* | *Gemmatimonadetes* | *Gemmatimonadales* | *Gemmatimonadaceae* | *Unclassified_Gemmatimonadaceae* | *Unclassified* |
| ASV1390 | 0.007975 | 0.42 | Organic_Ctrl | Spring | *Acidobacteriota* | *Blastocatellia* | *Blastocatellales* | *Blastocatellaceae* | *JGI 0001001-H03* | *Unclassified* |
| ASV1420 | 0.018408 | 0.35 | Organic_Ctrl | Spring | *Gemmatimonadota* | *Gemmatimonadetes* | *Gemmatimonadales* | *Gemmatimonadaceae* | *Unclassified_Gemmatimonadaceae* | *Unclassified* |
| ASV1455 | 0.008437 | 0.6 | Organic_Ctrl | Spring | *Proteobacteria* | *Alphaproteobacteria* | *Unclassified* | *Unclassified* | *Unclassified_Alphaproteobacteria* | *Unclassified* |
| ASV1461 | 0.008444 | 4.07 | Organic_Ctrl | Spring | *Proteobacteria* | *Alphaproteobacteria* | *Sphingomonadales* | *Sphingomonadaceae* | *Erythrobacter* | *Unclassified* |
| ASV1592 | 0.005878 | 0.72 | Organic_Ctrl | Spring | *Actinobacteriota* | *Actinobacteria* | *Corynebacteriales* | *Mycobacteriaceae* | *Mycobacterium* | *Unclassified* |
| ASV1612 | 0.010791 | 0.63 | Organic_Ctrl | Spring | *Proteobacteria* | *Gammaproteobacteria* | *Burkholderiales* | *SC-I-84* | *Unclassified_SC-I-84* | *Unclassified* |
| ASV1759 | 0.022244 | 0.41 | Organic_Ctrl | Spring | *Proteobacteria* | *Alphaproteobacteria* | *Rhizobiales* | *Xanthobacteraceae* | *Rhodoplanes* | *Unclassified* |
| ASV1885 | 0.008444 | 1.23 | Organic_Ctrl | Spring | *Firmicutes* | *Thermacetogenia* | *Thermacetogeniales* | *Thermacetogeniaceae* | *Syntrophaceticus* | *Unclassified* |
| ASV1896 | 6.97E-04 | 31.43 | Organic_Ctrl | Spring | *Acidobacteriota* | *Acidobacteriae* | *Solibacterales* | *Solibacteraceae* | *Candidatus Solibacter* | *Unclassified* |
| ASV2099 | 6.97E-04 | 21.52 | Organic_Ctrl | Spring | *Acidobacteriota* | *Thermoanaerobaculia* | *Thermoanaerobaculales* | *Thermoanaerobaculaceae* | *Subgroup 10* | *Unclassified* |
| ASV2130 | 6.97E-04 | 8.28 | Organic_Ctrl | Spring | *Actinobacteriota* | *Thermoleophilia* | *Gaiellales* | *Unclassified* | *Unclassified_Gaiellales* | *Unclassified* |
| ASV2131 | 0.046932 | 0.35 | Organic_Ctrl | Spring | *Bacteroidota* | *Bacteroidia* | *Sphingobacteriales* | *Sphingobacteriaceae* | *Arcticibacter* | *Unclassified* |
| ASV2164 | 0.008444 | 1.35 | Organic_Ctrl | Spring | *Acidobacteriota* | *Blastocatellia* | *Pyrinomonadales* | *Pyrinomonadaceae* | *RB41* | *Unclassified* |
| ASV2243 | 0.008444 | 2.47 | Organic_Ctrl | Spring | *Nitrospirota* | *Nitrospiria* | *Nitrospirales* | *Nitrospiraceae* | *Nitrospira* | *Unclassified* |
| ASV2261 | 6.97E-04 | 5.8 | Organic_Ctrl | Spring | *Proteobacteria* | *Alphaproteobacteria* | *Reyranellales* | *Reyranellaceae* | *Reyranella* | *Unclassified* |
| ASV2302 | 0.042489 | 0.63 | Organic_Ctrl | Spring | *Proteobacteria* | *Alphaproteobacteria* | *Sphingomonadales* | *Sphingomonadaceae* | *Sphingoaurantiacus* | *Unclassified* |
| ASV2361 | 0.008444 | 3.42 | Organic_Ctrl | Spring | *Proteobacteria* | *Alphaproteobacteria* | *Caulobacterales* | *Caulobacteraceae* | *Brevundimonas* | *Unclassified* |
| ASV2470 | 6.97E-04 | 7.21 | Organic_Ctrl | Spring | *Chloroflexi* | *Chloroflexia* | *Thermomicrobiales* | *JG30-KF-CM45* | *Unclassified_JG30-KF-CM45* | *Unclassified* |
| ASV2493 | 0.005379 | 2.18 | Organic_Ctrl | Spring | *Bacteroidota* | *Bacteroidia* | *Chitinophagales* | *Chitinophagaceae* | *Unclassified_Chitinophagaceae* | *Unclassified* |
| ASV2746 | 0.008444 | 3.42 | Organic_Ctrl | Spring | *Actinobacteriota* | *Actinobacteria* | *Propionibacteriales* | *Nocardioidaceae* | *Nocardioides* | *Nocardioides sp. MSL 21* |
| ASV2753 | 0.008444 | 2.02 | Organic_Ctrl | Spring | *Acidobacteriota* | *Thermoanaerobaculia* | *Thermoanaerobaculales* | *Thermoanaerobaculaceae* | *Subgroup 10* | *Unclassified* |
| ASV2824 | 0.008444 | 2.73 | Organic_Ctrl | Spring | *Chloroflexi* | *KD4-96* | *Unclassified* | *Unclassified* | *Unclassified_KD4-96* | *Unclassified* |
| ASV2896 | 0.008444 | 1.7 | Organic_Ctrl | Spring | *Actinobacteriota* | *Actinobacteria* | *Micromonosporales* | *Micromonosporaceae* | *Micromonospora* | *Micromonospora sp. B006* |
| ASV3265 | 0.010182 | 0.82 | Organic_Ctrl | Spring | *Actinobacteriota* | *Actinobacteria* | *Propionibacteriales* | *Nocardioidaceae* | *Nocardioides* | *Nocardioides sp.* |
| ASV331 | 0.027444 | 0.11 | Organic_Ctrl | Spring | *Actinobacteriota* | *Actinobacteria* | *Micrococcales* | *Microbacteriaceae* | *Microbacterium* | *Microbacterium sp. B-3029* |
| ASV400 | 0.008717 | 0.16 | Organic_Ctrl | Spring | *Bacteroidota* | *Bacteroidia* | *Sphingobacteriales* | *Sphingobacteriaceae* | *Pedobacter* | *Unclassified* |
| ASV504 | 0.032468 | 0.08 | Organic_Ctrl | Spring | *Actinobacteriota* | *Actinobacteria* | *Propionibacteriales* | *Nocardioidaceae* | *Nocardioides* | *Unclassified* |
| ASV518 | 0.011121 | 0.14 | Organic_Ctrl | Spring | *Actinobacteriota* | *Actinobacteria* | *Micromonosporales* | *Micromonosporaceae* | *Catellatospora* | *Catellatospora citrea* |
| ASV582 | 6.97E-04 | 15.35 | Organic_Ctrl | Spring | *Actinobacteriota* | *Actinobacteria* | *Corynebacteriales* | *Mycobacteriaceae* | *Mycobacterium* | *Unclassified* |
| ASV601 | 6.97E-04 | 4.87 | Organic_Ctrl | Spring | *Chloroflexi* | *Gitt-GS-136* | *Unclassified* | *Unclassified* | *Unclassified_Gitt-GS-136* | *Unclassified* |
| ASV613 | 0.040438 | 0.1 | Organic_Ctrl | Spring | *Actinobacteriota* | *Actinobacteria* | *Propionibacteriales* | *Nocardioidaceae* | *Marmoricola* | *Nocardioides sp. MTD22* |
| ASV675 | 0.008862 | 0.43 | Organic_Ctrl | Spring | *Chloroflexi* | *KD4-96* | *Unclassified* | *Unclassified* | *Unclassified_KD4-96* | *Unclassified* |
| ASV709 | 0.007975 | 0.56 | Organic_Ctrl | Spring | *Acidobacteriota* | *Blastocatellia* | *Blastocatellales* | *Blastocatellaceae* | *Unclassified_Blastocatellaceae* | *Unclassified* |
| ASV771 | 0.028049 | 0.1 | Organic_Ctrl | Spring | *Acidobacteriota* | *Blastocatellia* | *Pyrinomonadales* | *Pyrinomonadaceae* | *RB41* | *Unclassified* |
| ASV842 | 6.97E-04 | 7.93 | Organic_Ctrl | Spring | *Verrucomicrobiota* | *Verrucomicrobiae* | *Chthoniobacterales* | *Chthoniobacteraceae* | *Candidatus Udaeobacter* | *Unclassified* |
| ASV872 | 0.011169 | 0.2 | Organic_Ctrl | Spring | *Firmicutes* | *Bacilli* | *Paenibacillales* | *Paenibacillaceae* | *Paenibacillus* | *Unclassified* |
| ASV876 | 0.026345 | 0.16 | Organic_Ctrl | Spring | *Proteobacteria* | *Gammaproteobacteria* | *Burkholderiales* | *SC-I-84* | *Unclassified_SC-I-84* | *Unclassified* |
| ASV975 | 0.008444 | 0.22 | Organic_Ctrl | Spring | *Actinobacteriota* | *Actinobacteria* | *Propionibacteriales* | *Nocardioidaceae* | *Nocardioides* | *Unclassified* |
| ASV1065 | 0.033317 | 0.29 | Organic_Ctrl | Spring | *Proteobacteria* | *Alphaproteobacteria* | *Rhizobiales* | *Xanthobacteraceae* | *Rhodoplanes* | *Unclassified* |
| ASV1737 | 6.97E-04 | 18.83 | Organic_Ctrl | Spring | *Actinobacteriota* | *Actinobacteria* | *Streptosporangiales* | *Thermomonosporaceae* | *Actinocorallia* | *Unclassified* |
| ASV1856 | 6.97E-04 | 8.79 | Organic_Ctrl | Spring | *Nitrospirota* | *Nitrospiria* | *Nitrospirales* | *Nitrospiraceae* | *Nitrospira* | *Unclassified* |
| ASV321 | 0.016041 | 0.12 | Organic_Ctrl | Spring | *Proteobacteria* | *Alphaproteobacteria* | *Rhizobiales* | *Xanthobacteraceae* | *Unclassified_Xanthobacteraceae* | *Unclassified* |
| ASV554 | 0.01512 | 0.1 | Organic_Ctrl | Spring | *Proteobacteria* | *Gammaproteobacteria* | *Burkholderiales* | *Comamonadaceae* | *Rhizobacter* | *Unclassified* |
| ASV656 | 0.027444 | 0.18 | Organic_Ctrl | Spring | *Proteobacteria* | *Alphaproteobacteria* | *Rhizobiales* | *Xanthobacteraceae* | *Pseudolabrys* | *Unclassified* |
| ASV696 | 0.021521 | 0.15 | Organic_Ctrl | Spring | *Bacteroidota* | *Bacteroidia* | *Chitinophagales* | *Chitinophagaceae* | *Ferruginibacter* | *Unclassified* |
| ASV815 | 0.010087 | 0.25 | Organic_Ctrl | Spring | *Actinobacteriota* | *Thermoleophilia* | *Gaiellales* | *Gaiellaceae* | *Gaiella* | *Unclassified* |
| ASV853 | 0.043337 | 0.17 | Organic_Ctrl | Spring | *Proteobacteria* | *Alphaproteobacteria* | *Azospirillales* | *Unclassified* | *Unclassified_Azospirillales* | *Unclassified* |
| ASV911 | 0.03423 | 0.19 | Organic_Ctrl | Spring | *Proteobacteria* | *Alphaproteobacteria* | *Rhizobiales* | *Rhizobiales Incertae Sedis* | *Nordella* | *Unclassified* |
| ASV1356 | 0.01791 | 0.39 | Organic_Ctrl | Spring | *Proteobacteria* | *Gammaproteobacteria* | *Burkholderiales* | *SC-I-84* | *Unclassified_SC-I-84* | *Unclassified* |
| ASV498 | 0.005379 | 0.59 | Organic_Ctrl | Spring | *Acidobacteriota* | *Blastocatellia* | *Blastocatellales* | *Blastocatellaceae* | *Unclassified_Blastocatellaceae* | *Unclassified* |
| ASV607 | 0.013674 | 0.34 | Organic_Ctrl | Spring | *Proteobacteria* | *Alphaproteobacteria* | *Sphingomonadales* | *Sphingomonadaceae* | *Sphingomonas* | *Unclassified* |
| ASV306 | 0.018792 | 0.08 | Organic_Ctrl | Spring | *Proteobacteria* | *Gammaproteobacteria* | *Xanthomonadales* | *Xanthomonadaceae* | *Lysobacter* | *Lysobacter sp.* |
| ASV407 | 0.006716 | 1.11 | Organic_Ctrl | Spring | *Proteobacteria* | *Alphaproteobacteria* | *Rhizobiales* | *Hyphomicrobiaceae* | *Pedomicrobium* | *Unclassified* |
| ASV775 | 0.025683 | 0.21 | Organic_Ctrl | Spring | *Proteobacteria* | *Gammaproteobacteria* | *Burkholderiales* | *Comamonadaceae* | *Rhodoferax* | *Unclassified* |
| ASV991 | 0.032468 | -4.95 | Organic_Ctrl | Spring | *Actinobacteriota* | *Actinobacteria* | *Streptomycetales* | *Streptomycetaceae* | *Streptacidiphilus* | *Streptacidiphilus carbonis* |
| ASV323 | 0.045687 | 0.08 | Organic_Ctrl | Spring | *Acidobacteriota* | *Blastocatellia* | *Blastocatellales* | *Blastocatellaceae* | *Unclassified_Blastocatellaceae* | *Unclassified* |
| ASV1282 | 0.018792 | -11.6 | Organic_Ctrl | Spring | *Proteobacteria* | *Gammaproteobacteria* | *Burkholderiales* | *SC-I-84* | *Unclassified_SC-I-84* | *Unclassified* |
| ASV1542 | 0.032468 | -5.83 | Organic_Ctrl | Spring | *Proteobacteria* | *Gammaproteobacteria* | *Diplorickettsiales* | *Diplorickettsiaceae* | *Unclassified_Diplorickettsiaceae* | *Unclassified* |
| ASV298 | 0.016666 | -0.19 | Organic_Ctrl | Spring | *Proteobacteria* | *Alphaproteobacteria* | *Sphingomonadales* | *Sphingomonadaceae* | *Sphingomonas* | *Unclassified* |
| ASV420 | 0.018792 | -0.31 | Organic_Ctrl | Spring | *Proteobacteria* | *Alphaproteobacteria* | *Rhizobiales* | *Xanthobacteraceae* | *Rhodopseudomonas* | *alpha proteobacterium SK50-23* |
| ASV482 | 0.006716 | -3.12 | Organic_Ctrl | Spring | *Bacteroidota* | *Bacteroidia* | *Chitinophagales* | *Chitinophagaceae* | *Unclassified_Chitinophagaceae* | *Unclassified* |
| ASV721 | 0.03423 | -0.35 | Organic_Ctrl | Spring | *Actinobacteriota* | *Actinobacteria* | *Corynebacteriales* | *Nocardiaceae* | *Nocardia* | *Nocardia nova* |
| ASV828 | 0.010471 | -1.8 | Organic_Ctrl | Spring | *Proteobacteria* | *Gammaproteobacteria* | *Burkholderiales* | *Burkholderiaceae* | *Burkholderia-Caballeronia-Paraburkholderia* | *Burkholderia sp. DCY113* |
| ASV145 | 0.007975 | -0.7 | Organic_Ctrl | Spring | *Bacteroidota* | *Bacteroidia* | *Sphingobacteriales* | *Sphingobacteriaceae* | *Pedobacter* | *Pedobacter koreensis* |
| ASV609 | 0.01241 | -0.6 | Organic_Ctrl | Spring | *Proteobacteria* | *Gammaproteobacteria* | *Xanthomonadales* | *Rhodanobacteraceae* | *Tahibacter* | *Unclassified* |
| ASV95 | 0.007975 | -0.51 | Organic_Ctrl | Spring | *Bacteroidota* | *Bacteroidia* | *Sphingobacteriales* | *Sphingobacteriaceae* | *Pedobacter* | *Unclassified* |
| ASV276 | 0.007975 | -0.83 | Organic_Ctrl | Spring | *Proteobacteria* | *Gammaproteobacteria* | *Burkholderiales* | *Burkholderiaceae* | *Burkholderia-Caballeronia-Paraburkholderia* | *Paraburkholderia phytofirmans* |
| ASV375 | 0.030863 | 0.12 | Organic_Ctrl | Spring | *Actinobacteriota* | *Actinobacteria* | *Glycomycetales* | *Glycomycetaceae* | *Glycomyces* | *Glycomyces sp.* |
| ASV440 | 0.017992 | 0.29 | Organic_Ctrl | Spring | *Proteobacteria* | *Gammaproteobacteria* | *Burkholderiales* | *SC-I-84* | *agricultural soil bacterium SC-I-84* | *Unclassified* |
| ASV263 | 0.01116 | 0.12 | Organic_Ctrl | Spring | *Gemmatimonadota* | *Gemmatimonadetes* | *Gemmatimonadales* | *Gemmatimonadaceae* | *Unclassified_Gemmatimonadaceae* | *Unclassified* |
| ASV202 | 0.010182 | 0.09 | Organic_Ctrl | Spring | *Proteobacteria* | *Alphaproteobacteria* | *Rhizobiales* | *Rhizobiales Incertae Sedis* | *Unclassified_Rhizobiales Incertae Sedis* | *Unclassified* |
| ASV150 | 0.029104 | -0.16 | Organic_Ctrl | Spring | *Proteobacteria* | *Gammaproteobacteria* | *Burkholderiales* | *Burkholderiaceae* | *Burkholderia-Caballeronia-Paraburkholderia* | *Burkholderia sp. HB1* |
| ASV106 | 0.008444 | 0.11 | Organic_Ctrl | Spring | *Proteobacteria* | *Alphaproteobacteria* | *Rhizobiales* | *Stappiaceae* | *Stappia* | *Unclassified* |
| ASV111 | 0.008444 | 0.07 | Organic_Ctrl | Spring | *Actinobacteriota* | *Actinobacteria* | *Streptomycetales* | *Streptomycetaceae* | *Streptomyces* | *Streptomyces sp. N01-1637* |
