# Supplementary material for: Beneficial microbial consortium improves winter rye performance by modulating bacterial communities in the rhizosphere and enhancing plant nutrient acquisition
Source: Front Plant Sci. 2023 Aug 28;14:1232288. doi: 10.3389/fpls.2023.1232288 (PMC10498285; doi:10.3389/fpls.2023.1232288)
Supplement: Supplementary file 11 [file Table_10.docx]

**Supplementary table 10.** ASVs that significantly differed in the rhizosphere of Maize plants and associated with BMc inoculated Maize plants under Organic or Conventional farming in the two sampling seasons. Differential testing was performed as described in Supplementary table 9.

| **ASV** | **p_value_adj** | **Model Coefficient** | **Season** | **Phylum** | **Class** | **Order** | **Family** | **Genus** | **Species** |
| --- | --- | --- | --- | --- | --- | --- | --- | --- | --- |
| ASV511 | 0.02 | 0.15 | Autumn | Actinobacteriota | Actinobacteria | Micrococcales | Micrococcaceae | Paeniglutamicibacter | Arthrobacter sp. |
| ASV511 | 0.01 | 0.15 | Spring | Actinobacteriota | Actinobacteria | Micrococcales | Micrococcaceae | Paeniglutamicibacter | Arthrobacter sp. |
| ASV63 | 0.045 | -0.04 | Autumn | Actinobacteriota | Actinobacteria | Micrococcales | Microbacteriaceae | Unclassified_Microbacteriaceae | Unclassified |
| ASV63 | 0.045 | -0.04 | Spring | Actinobacteriota | Actinobacteria | Micrococcales | Microbacteriaceae | Unclassified_Microbacteriaceae | Unclassified |
| ASV88 | 0.015 | -0.08 | Autumn | Bacteroidota | Bacteroidia | Chitinophagales | Chitinophagaceae | Chitinophaga | Unclassified |
| ASV88 | 0.024 | -0.08 | Spring | Bacteroidota | Bacteroidia | Chitinophagales | Chitinophagaceae | Chitinophaga | Unclassified |
